# Proton-Sensitive Free-Radical Dimer Evolution Is a Critical Control Point for the Synthesis of Δ^2,2^′^^-Bibenzothiazines

**DOI:** 10.1021/acs.joc.0c01520

**Published:** 2020-08-26

**Authors:** Luca Valgimigli, Maria Laura Alfieri, Riccardo Amorati, Andrea Baschieri, Orlando Crescenzi, Alessandra Napolitano, Marco d’Ischia

**Affiliations:** † Department of Chemistry “Giacomo Ciamician”, University of Bologna, Bologna I-40126, Italy; ‡ Department of Chemical Sciences, University of Naples Federico II, Naples I-80126, Italy

## Abstract

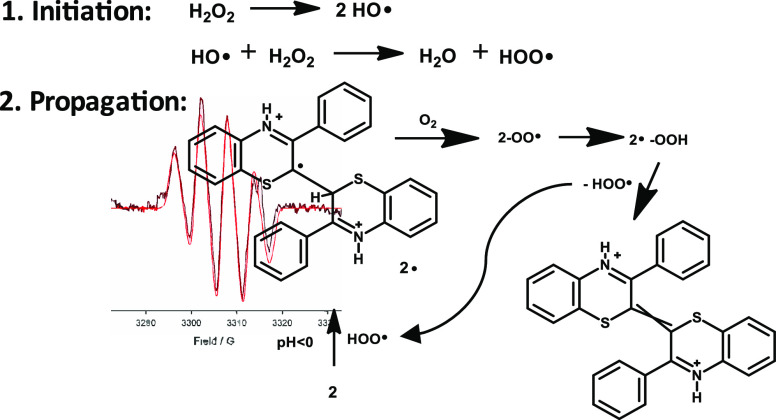

The mechanism of the acid-dependent interring dehydrogenation in the conversion of the single-bonded 3-phenyl-2*H*-1,4-benzothiazine dimer **2** to the Δ^2,2^′^^-bi(2*H*-1,4-benzothiazine) scaffold of red hair pigments is disclosed herein. Integrated chemical oxidation and oxygen consumption experiments, coupled with electron paramagnetic resonance (EPR) analyses and DFT calculations, allowed the identification of a key diprotonated free-radical intermediate, which was implicated in a remarkable oxygen-dependent chain process via peroxyl radical formation and evolution to give the Δ^2,2^′^^-bi(2*H*-1,4-benzothiazine) dimer **3** by interring dehydrogenation. The critical requirement for strongly acidic conditions was rationalized for the first time by the differential evolution channels of isomeric peroxyl radical intermediates at the 2- versus 3-positions. These results offer for the first time a rationale to expand the synthetic scope of the double interring dehydrogenation pathway for the preparation of novel symmetric double-bond bridged captodative heterocycles.

## Introduction

**Figure 1:**
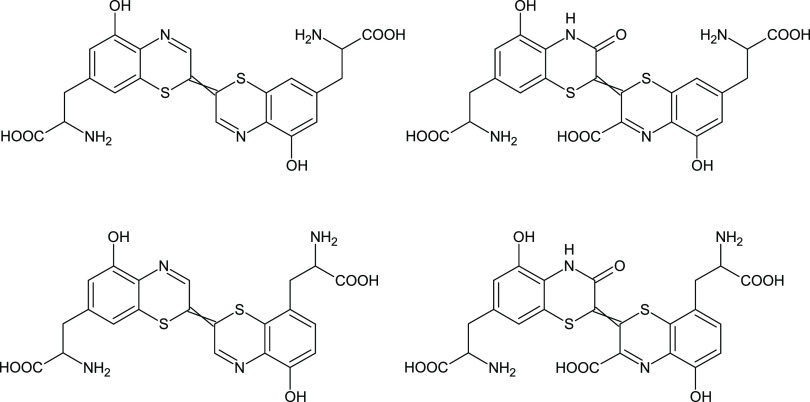
Structures of red hair pigment trichochromes.

The Δ^2,2^′^^-bibenzothiazine system, the core structure of trichochromes and related pigments found in red hair and feathers, is characterized by an interring double bond, which allows efficient push–pull interactions and π-electron conjugation across the S–C=C–C=N– systems (Figure 1).^1−4^ Because of the indigoid nature of the chromophore and the inherent structural rigidity, Δ^2,2^′^^-bibenzothiazines display redox properties and an intense absorption in the visible region with a peculiar acidichromic behavior finely tunable by substituents, which attract potential interest for various applications.^5^

**Scheme 1:**
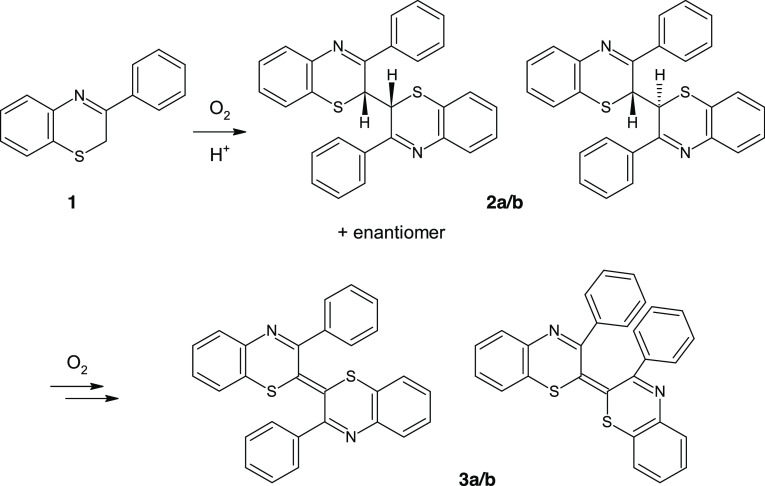
Oxidative Coupling of 3-Phenyl-1,4-benzothiazine to Δ^2,2^′^^-Bibenzothiazine **3** via 2,2′-Bi(2*H*-1,4-benzothiazine) **2**

From the synthetic point of view, Δ^2,2^′^^-bibenzothiazines can be easily produced by the doubly dehydrogenative dimerization of 2*H*-1,4-benzothiazine derivatives. This process is spontaneous and remarkably facile in strongly acidic media and in the presence of oxygen, but it is completely inhibited under mild or nonacidic conditions (Scheme 1).^1,4^

The factors accounting for the tendency of two sp^3^ C–H bonds in the benzothiazine ring to undergo doubly dehydrogenative coupling are of mechanistic interest in the broad general context of the C–H bond activation strategies.^6−9^ Recent evidence^10^ suggested that the marked activating effect of acids on the dehydrogenative coupling of 3-phenyl-1,4-benzothiazine (**1**) to the corresponding Δ^2,2^′^^-bibenzothiazine (Scheme 1) is due to a decrease in the energy of the initial H-atom abstraction step caused by N-protonation.

The process evolves via captodatively stabilized^10,11^ free-radical intermediates, which appear to dimerize rather than to couple with oxygen, based on the lack of detectable oxygenated products or intermediates.

The most puzzling issue in this process concerns the role of acids and oxygen in the desaturation step. Current evidence indicates that the single-bonded dimer can be detected as an intermediate or isolated at neutral pH.^10^ Its conversion to the final double-bonded bibenzothiazine is promoted by hydrogen peroxide and is critically dependent on (a) strong acids and (b) the presence of oxygen. So far, the role of acids and the involvement and fate of oxygen in the conversion of the interring single bond in **2** to the double bond in **3** have remained little understood.

Herein, we report electron paramagnetic resonance spectroscopy (EPR), oxygen uptake experiments, and DFT calculations on the mechanism of desaturation of **2** to **3** by H_2_O_2_. Specific aims of the study were (a) to elucidate the role of oxygen and strong acids in the dehydrogenative conversion of the interring single bond in **2** to the double bond in **3** and (b) to identify and characterize free-radical intermediates in the process, as yet still elusive.

## Results and Discussion

### Chemical Oxidation Experiments

Initial experiments were directed to reassess the mechanism of formation of the unsaturated Δ^2,2^′^^-bibenzothiazine system on monomer **1** as a probe substrate under various oxidation conditions.

It was thus confirmed that under strongly acidic conditions, i.e., methanol/36% HCl 3:1, **1** reacts rapidly with H_2_O_2_ in the presence of oxygen to give dehydrogenated dimer **3**,^10^ whereas in neutral organic solvents, e.g., methanol, no reaction occurred with H_2_O_2_ even over prolonged periods of time.

Reaction of **1** with free-radical species such as 2,2-diphenyl-1-picrylhydrazyl (DPPH) or 4-hydroxy-2,2,6,6-tetramethylpiperidin-1-oxyl (TEMPO) induced a slow conversion to the single-bonded dimer **2a**/**b**, without detectable **3a**/**b**. Under mildly acidic conditions, such as picric acid in ethanol, **1** was converted to dimers **2a**/**b**, which accumulated, and were not further oxidized to **3**. When purified dimers **2a**/**b** were exposed to oxidants like 2,3-dichloro-5,6-dicyanobenzoquinone or chloranil or to an excess of DPPH or TEMPO in organic solvents, no significant reaction occurred. On the other hand, peroxides and hydroperoxides were shown to allow the conversion of **1** into **3**(10) and **2** to **3** (Figure S1) though hydrogen peroxide was found to be the most efficient and synthetically convenient oxidant to promote the conversion.

The conversion of dimers **2a**/**b** to **3** in methanol under acidic conditions in the presence of excess H_2_O_2_ was next investigated by spectrophotometrically monitoring the development of the green-blue chromophore of **3** at 598 nm after 30 min in methanol/aq. HCl 3:1, using different concentrations of the acid (Figure S2). Formation of **3** was apparent with 3 M or 2 M HCl, whereas at acid concentration below 1 M, no chromophore was observed. Formation of double-bonded dimer **3** was confirmed by HPLC analysis.

### EPR Spectroscopy

In our previous study in which the reaction of 12.5–25 mM **1** in air-equilibrated MeOH containing 3 M HCl was performed in the cavity of an electron paramagnetic resonance (EPR) spectrometer, we observed that addition of H_2_O_2_ (final concentration, 0.2–0.4 equiv) to the solution led to the slow buildup of a weak EPR signal centered at *g* = 2.0051 with a characteristic hyperfine structure,^10^ while no signal was observed in control experiments in the absence of H_2_O_2_. The hyperfine structure of the signal showed large coupling constants (ca. 6 Gauss) due to two different protons (spin = ^1^/_2_), one relatively large coupling constant (ca. 3 Gauss) with nitrogen (spin = 1) and two smaller coupling constants with nonequivalent protons. Both the measured *g*-factor and the hyperfine structure were not compatible with peroxyl radicals (**1**-OO^·^) produced by coupling of oxygen with a carbon-centered radical (expected *g* ≈ 2.015)^12^ but suggested rather a C/N-centered conjugated radical with spin delocalization on heavier atoms like sulfur. In this connection, the radical cations of protonated/methylated 1,4-diazines (lacking sulfur) exhibit *g*-factors in the range 2.0030–2.0033,^13^ similar to *N*,*N*-diphenylaminyl radical, *g* = 2.0032, while the structurally related aminyl radical of phenothiazine exhibited *g* = 2.0046.^14^

**Figure 2:**
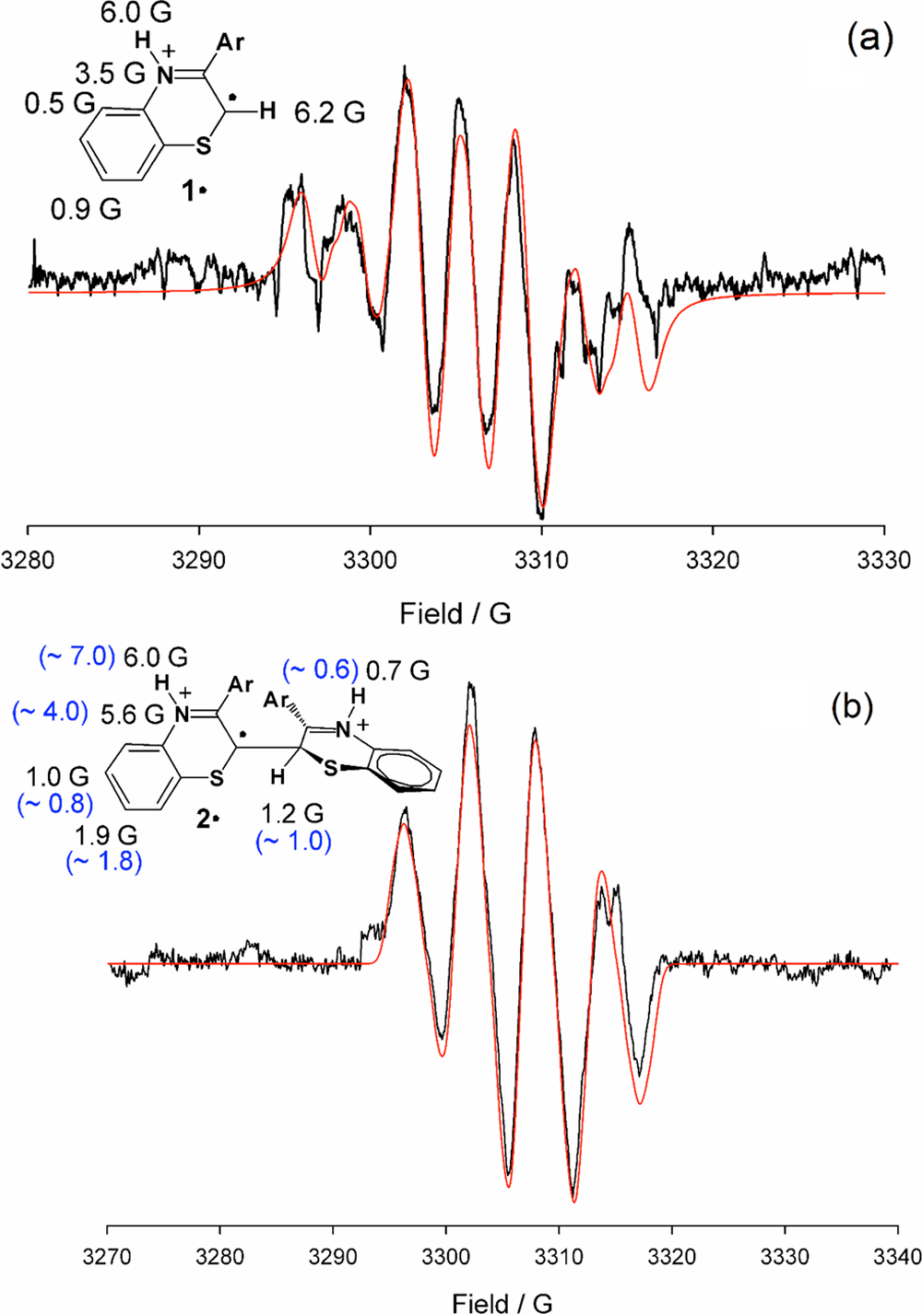
Experimental EPR spectrum obtained with (a) 12.5 mM monomer **1** or (b) dimer **2** in MeOH/36% HCl 3:1 upon reaction with 4 mM H_2_O_2_ (black) and its computer simulation (red) using the coupling constants (*hccs*) displayed on the structure. Simulation of the spectrum shown for monomer **1** (panel a) was obtained with a radical mixture of **1**^·^ and **2**^·^ at a 10:6 ratio matched with the experimental spectrum. For radical **2**^·^, calculated *hccs* (see the Supporting Information) are reported in parentheses (blue).

DFT calculations of the expected spin distribution and corresponding EPR coupling constants for neutral and protonated radical **1**^·^ (Table S1) allowed to rule out neutral species, unequivocally assigning the spectrum to the protonated radical. Simulation of the EPR spectra and interactive fitting according to the Monte Carlo method,^15^ using the DFT-calculated coupling constants as an initial input, allowed to reproduce reasonably well the experimental spectra despite the modest signal/noise ratio. Match of simulated and experimental spectra was, however, not fully satisfactory, in that the central portion of the experimental spectrum (of the highest quality) showed three lines of approximately equal intensity (Figure 2a), at variance with simulation predicting a less intense central line (Figure S3). We hypothesized that this was due to superimposition of the spectrum of radical **1**^·^ to that of other radical species formed in the reaction mixture. The most reasonable candidate was the dimeric radical **2**^·^, formed upon oxidation of **2** (see Scheme 1).

To confirm this hypothesis and shed more light on the mechanism of conversion of **1** to **3**, we performed matched EPR experiments using dimer **2** in place of phenylbenzothiazine **1**. Upon addition of H_2_O_2_, an intense spectrum was observed (Figure 2b). The measured *g*-factor was 2.0052, just slightly higher than that of radical **1**^·^, indicating a similar structure with increased spin delocalization on heavy atoms (e.g., two sulfur atoms of the two benzothiazine moieties). Spectral analysis and interactive simulation afforded coupling constants in excellent agreement with those calculated for the diprotonated radical **2**^·^ (Figure 2b and Table S2), while the agreement with those calculated for the monoprotonated species were less satisfactory. Interestingly, no EPR signal attributable to the corresponding peroxyl radical **2**-OO^·^, expected at a much lower field, could be observed although experiments were performed in air-saturated solutions, confirming a general instability of peroxyl radical intermediates.^12^ This is in keeping with the work of Pratt and Porter who showed that β-fragmentation of an alkylperoxyl radical is facilitated by electron-withdrawing substituents (like the iminium function in **2**-OO^·^) that would destabilize it while stabilizing the C-centered radical (e.g., **2**^·^) due to hyperconjugative effects.^9^

With both sets of spectral parameters available, simulations of spectra due to the superimposition of those of protonated radical **1**^·^ and diprotonated radical **2**^·^ were fitted to the experimental EPR spectrum previously assigned to radical **1**^·^ alone (see above). The quality of matching in the central portion of the spectrum was significantly improved (Figure 2a), confirming our hypothesis. This finding fully supports a free-radical formation pathway for dimer **3**.

### Oxygen Uptake Measurements

Our previous investigation showed that oxygen is necessary to the overall process of conversion of phenylbenzothiazine **1** to colored dimer **3**, as no color development is observed in the presence of various oxidizing species in the absence of oxygen.^10^ On the other hand, from analysis of the products, it clearly appears that oxygen is not incorporated in the reaction products, suggesting that any oxygen consumed in the process must be eliminated, e.g., in the form of water or as hydrogen peroxide. EPR studies did not reveal the formation of oxygen-centered radicals such as peroxyl radicals. The role of oxygen in the conversion of **2a**/**b** to **3** was then investigated by monitoring oxygen consumption in a differential oxygen uptake apparatus.^16,17^

**Figure 3:**
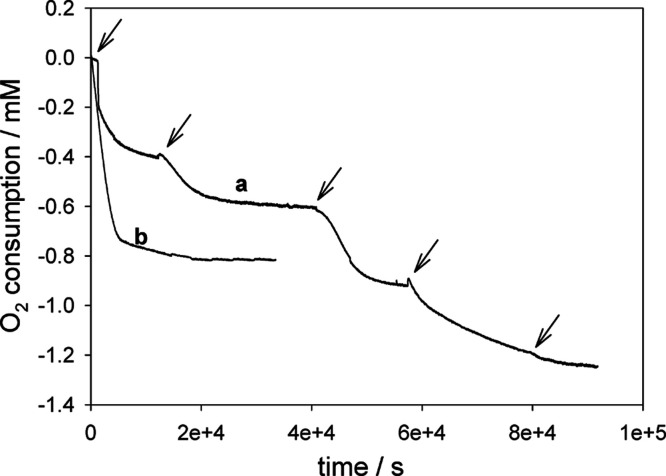
Oxygen uptake measured by incubating 5 μmol of dimer **2** in 4 mL of MeOH containing 3 M H_2_SO_4_ (final conc., 1.25 mM) at 303 K with (a) addition of aliquots of 1 μmol of H_2_O_2_ to the system at time points indicated by an arrow or (b) by a single addition of 10 μmol of H_2_O_2_.

When dimer **2** was incubated in MeOH containing 3 M H_2_SO_4_ at 303 K, oxygen consumption was poor until 1 μmol of H_2_O_2_ was added to the system, causing a rapid oxygen consumption, which stopped after approximately 0.2–0.3 equiv (with respect to the starting compound) had been consumed. Reinjection of a second aliquot of H_2_O_2_ caused oxygen consumption to restart, and the phenomenon was observed for subsequent additions of H_2_O_2_ until the reaction was complete (Figure 3a, the fifth addition causes no further reaction). The exact stoichiometry of oxygen uptake depended on the initial concentration of H_2_O_2_, ranging from 0.6 to 1.0 with respect to the starting dimer **2**. Indeed, addition of equimolar H_2_O_2_ as a single aliquot resulted in a lower oxygen consumption with respect to that obtained by repeated addition of substoichiometric amounts, and a large molar excess brought the reaction close to completion with apparent lower overall O_2_ consumption (Figure 3b), likely as a result of acid-catalyzed dismutation of H_2_O_2_ that partly restores the oxygen consumed by the reaction. Indeed, control experiments where a similar amount (10 μmol) of H_2_O_2_ was added to 3 M H_2_SO_4_ in MeOH, in the oxygen uptake apparatus, in the absence of dimer **2** showed oxygen evolution at a rate compatible with the “missing” oxygen consumption recorded in the presence of dimer **2** (Figure S4). Incubating monomer **1** in place of the dimer under similar settings, a similar behavior was observed, with a higher oxygen consumption following repeated addition of H_2_O_2_ than that observed by adding the same overall amount in a single bolus (Figure S5). On the basis of these data, it can be concluded that in the present experiment, hydrogen peroxide plays a key role in the generation of the free-radical intermediates, while oxygen is involved in propagation steps. It may also be noted that the need for an amount of H_2_O_2_ comparable to that of **1** or **2**, albeit substoichiometric, and the consumption of 0.6–1.0 equiv of oxygen suggest that the chain reaction is poorly efficient (short chain), and other mechanistic possibilities are likely coinvolved.

### DFT Calculations

The results reported above indicated that conversion of **2** to **3** requires strong acids, to allow the generation of an EPR-detectable diprotonated free-radical dimer, and is activated by addition of hydrogen peroxide triggering oxygen consumption. However, the precise step underlying the critical requirement for strong acids and the actual role of oxygen in promoting desaturation of the diprotonated dimer remained unclear.

To settle these issues, the influence of protonation on the various critical steps of the most plausible reaction pathways was assessed by DFT calculations. The PBE0^18^ functional in combination with a reasonably large basis set [6-31+G(d,p)] was used for extensive structural explorations and for computation of vibrational–rotational contributions to the free energy. The M06-2X^19^ functional with a much larger basis set [6-311++G(2d,2p)] was adopted for single-point energy evaluations. Geometry optimizations were performed either in vacuo or by adoption of a polarizable continuum medium (PCM)^20^ to account for the influence of the solution environment. The M06-2X single-point calculations also included nonelectrostatic contributions to the solvation free energy, employing radii and nonelectrostatic terms of the SMD solvation model.^21^

The pH value in the methanol-containing medium adopted in the experiments of this study was estimated by making a reference to substituted anilines, for which p*K*_a_ values in methanol were reported.^22^ The extent of protonation of the selected anilines was evaluated based on the shifts of the absorption maxima of the protonated or free base forms in the 3:1 methanol/HCl with the acid at the concentrations used (Figure S6). On this basis, the pH of the methanol/3 M HCl medium was estimated to be below 0, whereas for the methanol/1 M HCl medium, the estimated pH was higher than 1.

**Scheme 2:**
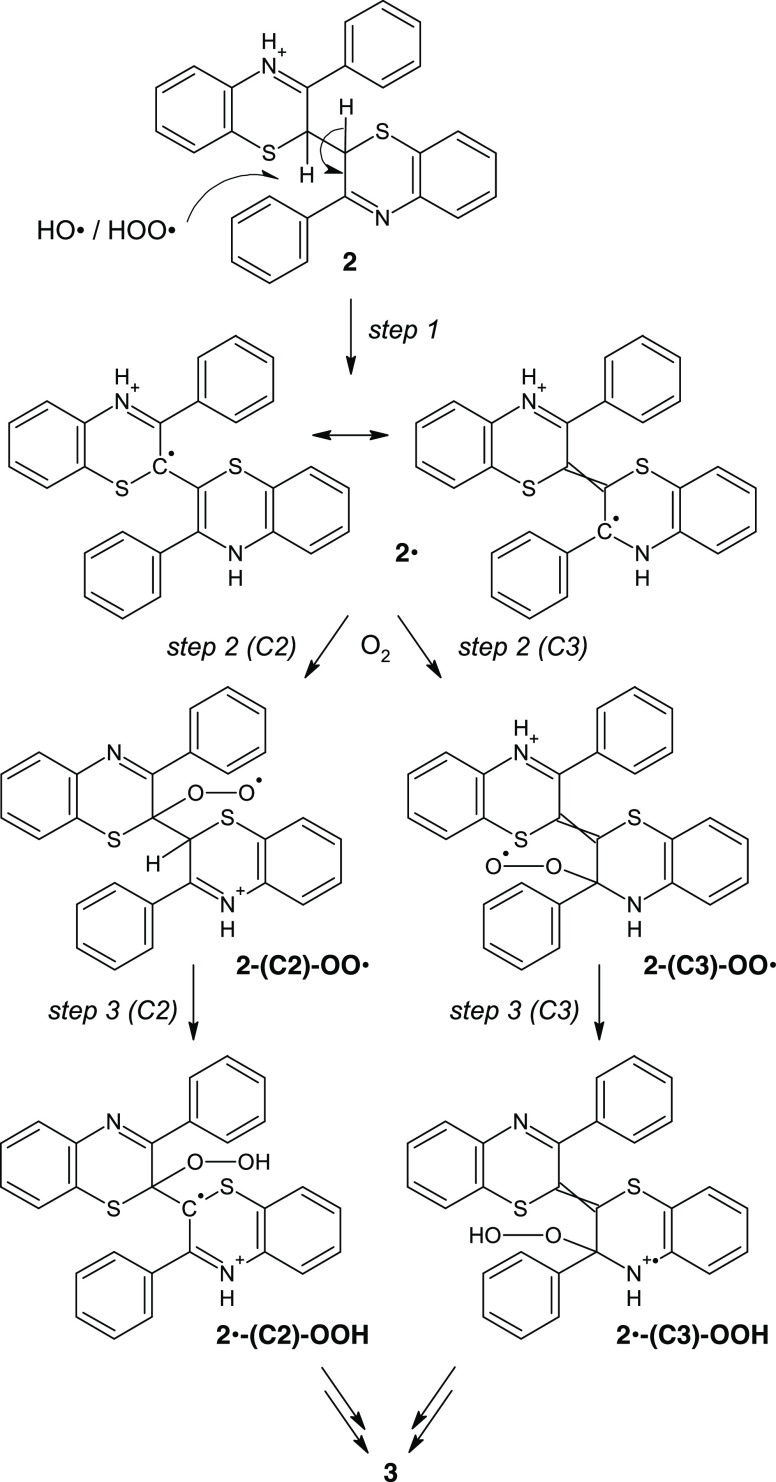
Proposed Formation and Evolution Pathways of the Free-Radical Dimer **2**· in Acidic Methanol For each species in the reaction path, only the most stable tautomer of the monoprotonated form is represented; however, depending on the specific pH of the reaction medium, each species will populate several tautomers of both the mono- and diprotonated forms. A detailed DFT characterization of such protonation microstates is provided as Supporting Information.

The reaction pathways considered for this study are illustrated in Scheme 2 for the case of monoprotonated species. They involve the following key steps.

(*Step 1*) H-atom abstraction from the single-bonded dimer **2** either by a hydroxyl radical (HO^·^), produced from hydrogen peroxide, or by the hydroperoxyl radical (HOO^·^), produced from hydrogen peroxide and HO^·^ (eq 1, initiation) or during propagation steps (*vide infra*), to give dimer radical **2**^·^ existing mainly as a resonance-stabilized captodative form as an enamine tautomer.
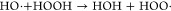
1

(*Step 2 (C2)/2 (C3)*) Free-radical coupling of the dimer radical **2**^·^ with oxygen to give isomeric peroxyl radical intermediates at C2/C3 (**2-(C2)-OO^·^** and **2-(C3)-OO^·^**, respectively).

(*Step 3 (C2)/3 (C3)*) Intramolecular H-atom abstraction to generate the corresponding β-hydroperoxyalkyl/aminyl radicals, **2^·^-(C2)-OOH** and **2^·^-(C3)-OOH**.

The reaction proceeds with identical ease and outcomes either in the presence of 3 M HCl or of 3 M H_2_SO_4_, suggesting that it is not promoted by a specific acid, but it requires a strongly acidic medium.

**Scheme 3:**
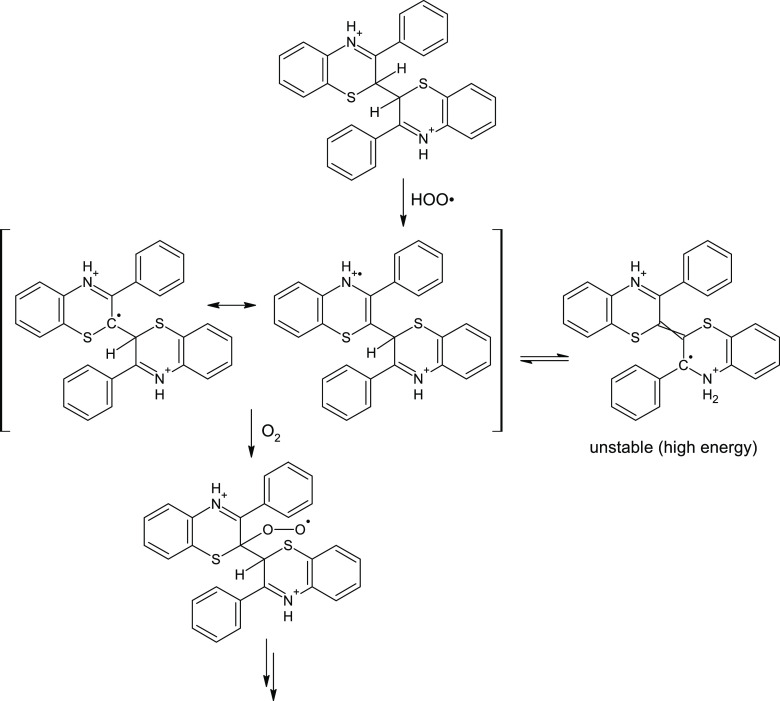
Main Structures for the Diprotonated Form of the Free-Radical Dimer **2**· in Methanol

For the present study, steps 1–3 were investigated in methanol as a solvent. All species involved were fully characterized in all possible protonation states, including consideration of the different tautomers and of conformational equilibria; p*K*_a_ values for the two protonation steps were estimated by comparison of the computed free-energy changes with those obtained at the same theory level for a series of nitrogen bases for which experimental data in methanol were available.^22^ Only the most stable species are represented in the following schemes. Further details of the computational aspects are provided in the Supporting Information (Tables S3–S8), including energy data at different theory levels for the most stable conformer of each species examined and computed p*K*_a_ data. Representative formulae for the free-radical intermediates in the diprotonated forms and relevant equilibria are provided in Scheme 3.

Simple inspection of Scheme 3 reveals an important mechanistic clue: H atom abstraction from the C2 position of the diprotonated form of **2** generates a resonance-stabilized free radical localized on a single thiazine ring due to disruption of captodative interring resonance effects, with higher spin density on the 2- and N-positions. Oxygen coupling at C3 is prevented unless a tautomerization step is considered, which, however, leads to an unstable (ca. 8 kcal/mol) >NH_2_^+^ species.

**Figure 4:**
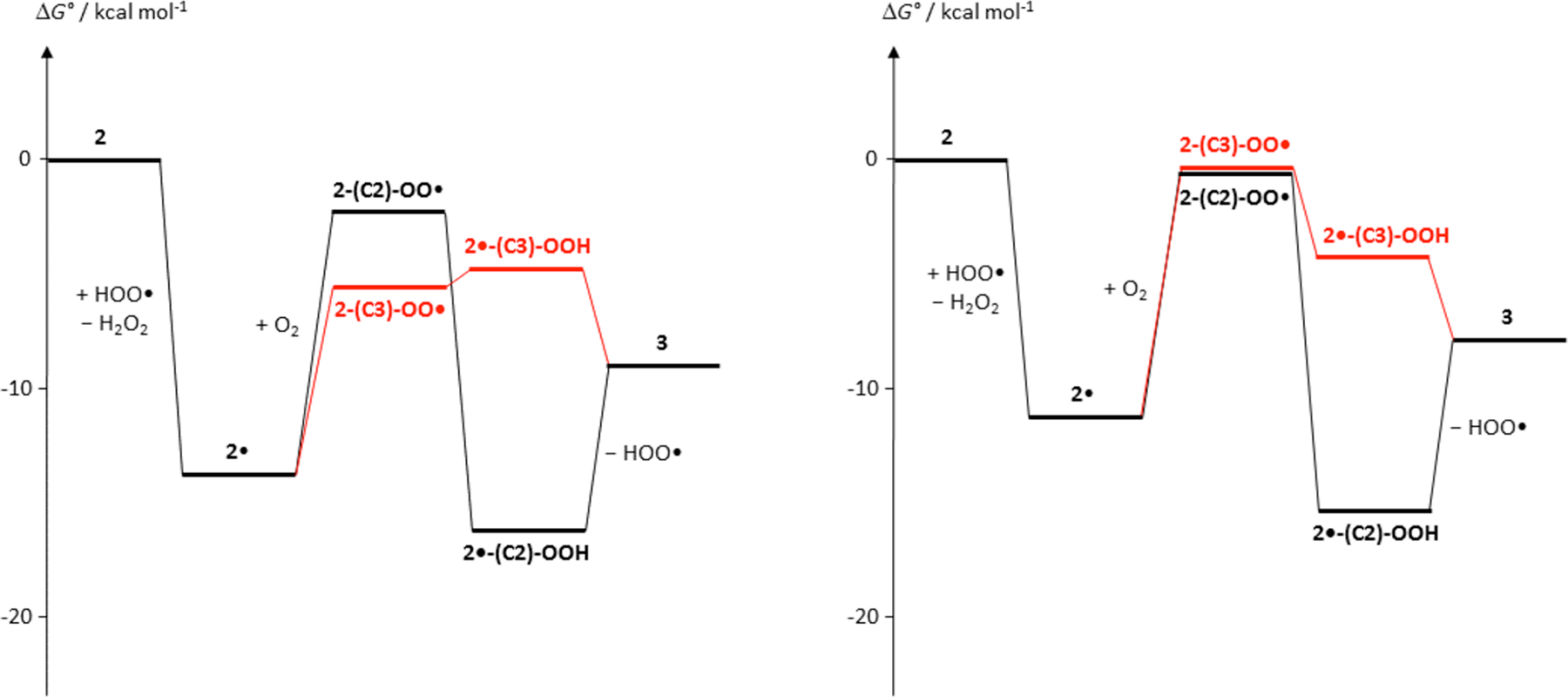
Computed free-energy diagram for reagents, products, and putative intermediates in the reaction pathway leading from **2** to **3**, under different pH conditions. (left panel) pH 3.0; (right panel) pH −1.5.

Consistent with this view, computational analysis of the regioisomeric free-radical intermediates in Scheme 2 revealed the most noticeable difference in the relative energies as a function of the protonation state. Whereas under neutral or weakly acidic conditions, oxygen coupling proved to be more favorable at C3 than at C2, as inferred by the greater stability of **2-(C3)-OO^·^** over **2-(C2)-OO^·^** (2.9 kcal/mol for the neutral forms and 4.3 for the monoprotonated forms), the situation is reversed in the case of the diprotonated forms, with **2-(C2)-OO^·^** more stable than **2-(C3)-OO^·^** by 6.2 kcal/mol. At acidic pH, moreover, step 1 proved to be more exergonic than under neutral conditions (by 8.8 kcal/mol for the monoprotonated forms and by 3.2 kcal/mol for the diprotonated forms). The pH effects (selected pH 3 and −1.5) are summarized graphically in Figure 4, in which the free energy of each species has been corrected to account for the coexistence of different protonation forms, based on the computed p*K*_a_ values (Table S7).

It follows from the points above that only a strongly acidic medium can efficiently direct the reaction pathway toward the C2 coupling route, which appears to be the privileged channel to interring dehydrogenation and product formation compared to the C3 route. Conversion of peroxyl radicals at C2 to the final Δ^2,2^′^^ dimeric product **3** may take different nonexclusive pathways, namely, (a) intramolecular H-atom abstraction by −OO^·^ from C–H in 2′ to form a hydroperoxide intermediate bearing a C-centered radical that may cleave to release the hydroperoxyl radical HOO^·^ (the two steps, intramolecular H-abstraction and loss of HOO^·^ could be concerted) (Figure 4 for selected pH 3 and −1.5 and Figure S7 for other pH conditions explored) or (b) intermolecular H-atom abstraction (from hydrogen peroxide generating HOO^·^ as a chain transporter) to give a hydroperoxide intermediate, which would then undergo loss of H_2_O_2_ (Figure S8).

In a highly acidic medium, the reaction path (a) resembles the chemistry recently described by Pratt and coworkers to explain the release of HOO^·^ during the autoxidation of unsaturated hydrocarbons and is likely to benefit from accelerated kinetics due to quantum tunneling of the activation barrier.^23,24^ In a highly acidic medium, release of HOO^·^ as the last step also justifies the radical-chain nature of this reaction, which requires less than stoichiometric amounts of initiating reactants (e.g., H_2_O_2_) to proceed to completion.

**Figure 5:**
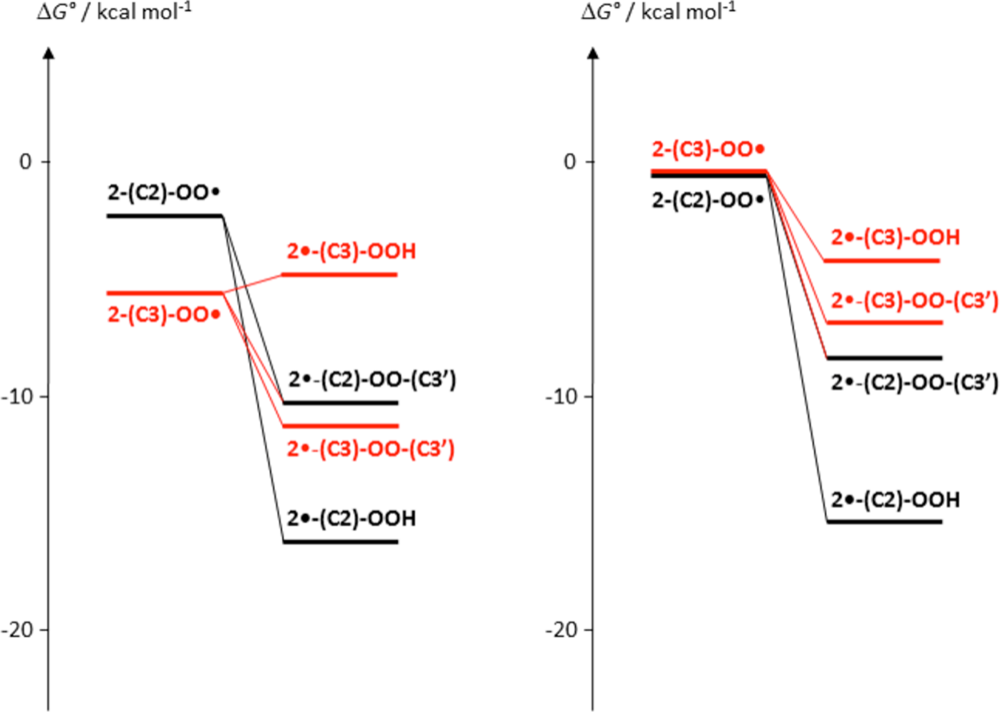
Computed free-energy diagram for alternative evolution pathways of the peroxyl radicals from **2**. The energy scale is the same as in Figure 4. (left panel) pH 3.0; (right panel) pH −1.5.

**Scheme 4:**
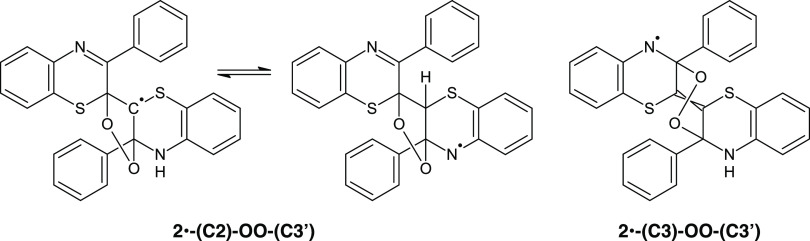
Structures of Possible Endoperoxidic Products from the Isomeric **2**-(C2)-OO·/**2**-(C3)-OO· Radicals

On the other hand, isomeric peroxyl radicals at C3 may take convenient reaction channels based, e.g., on cyclization leading to endoperoxides (Scheme 4), as predicted by DFT calculations, which would hardly evolve toward interring dehydrogenation and formation of **3** (Figure 5).

ESI-MS analysis of the crude product mixture arising from oxidation of **2** to **3** did not reveal the formation of signals attributable to endoperoxides although the ionization conditions might have caused decomposition of such moderately stable products. Although experimental data do not allow to unambiguously demonstrate the unproductive outcome of coupling reactions at C3 and the putative endoperoxy intermediates thereof, it is worth noting that the analogous cyclization routes of peroxyl radicals on the 2-position are not favored over intramolecular H-abstraction (Figure 5), whereby elimination/fragmentation with loss of oxygen appears to be by far the best option possible for peroxy species at C2. Additionally, elimination/fragmentation is in line with the lack of recovery of oxygenated products and with the observations by EPR spectroscopy.

A more detailed investigation of the reaction pathway at the transition state level was hindered by the highly demanding computational effort, especially on account of the remarkable conformational freedom of most species. Nonetheless, the clear pH-dependent trend of the energy order for the matching series of intermediates along the competing oxygenation pathways at C2 vs. C3 justifies reliance on Hammond’s postulate to put the main mechanistic conclusions of this study on solid ground.

## Conclusions

Altogether, chemical, EPR, and oxygen consumption data coupled with DFT calculations allowed to propose for the first time a consistent mechanism accounting for the intriguing acid-promoted interring dehydrogenation of single-bonded dimer **2** leading to the central bibenzothiazine core of red hair pigments. In addition, it has been possible to identify and characterize the transient free-radical dimer **2**^·^ in its diprotonated form by careful EPR experiments coupled with computational analysis.

Besides shedding new light on the chemistry of captodative free radicals with oxygen, these results offer an improved rationale to expand the synthetic scope of the double interring dehydrogenation pathway for the preparation of novel symmetric double-bond bridged captodative heterocycles.

## Experimental Section

### General Information

All solvents and reagents were obtained from commercial sources and used without further purification. UV–vis absorption spectra were registered at room temperature on a V-560 JASCO spectrophotometer using calibrated 2 mL quartz cuvettes. LC–MS analyses were performed on an HPLC instrument Agilent 1100 Series MSD equipped with a UV–vis detector and an electrospray ionization source in positive ion mode (ESI^+^). Detection wavelength was set at 254 nm. The spray voltage was set at 3.5 kV. Nitrogen was employed as both drying and nebulizer gas. Mass spectra were registered with the cone and fragmentator voltage set at 4 kV and 80 V, respectively. An octyl column (15 cm × 4.6 mm, 3 μm particle size) was used. An acetonitrile/water gradient was used as follows: 0–50 min, 50–70% acetonitrile and 50–60 min, 70% acetonitrile. The flow rate was set at of 0.7 mL/min.

2,2-Diphenyl-1-picrylhydrazyl (DPPH) or 4-hydroxy-2,2,6,6-tetramethylpiperidin-1-oxyl (TEMPO), 2,3-dichloro-5,6-dicyanobenzoquinone, sodium persulfate, *m*-chloroperbenzoic acid, iron(II) chloride, and chloranil were purchased from Sigma-Aldrich. 3-Phenyl-1,4-benzothiazine (**1**) and 3,3′-diphenyl-2,2′-bi(1,4-benzothiazine) (**2**) were prepared as previously described.^10^

### Oxidation Reaction of **1** or **2**

(a) Picric acid: Compound **1** at 13 mM in ethanol was treated with equimolar picric acid, and the mixture was left at reflux under vigorous stirring in air. The reaction course was followed by HPLC analysis. After 30 min at complete consumption of the starting material, two main products at *R*_T_ of 45.2 and 47.6 min at a 1:2 ratio identified as the single-bonded dimers **2a**/**b** (meso/dl pair diastereoisomers) were formed. The solid that separates from the mixture was shown to consist of a single compound (*R*_T_ = 47.6 min) by HPLC analysis. Under the same conditions, dimers **2a**/**b** were not appreciably consumed as evidenced by HPLC analysis.
(b) DDQ or Chloranil: The reaction was carried out on **1** or **2** at rt as in (a) using dioxane as the solvent and the oxidant at equimolar concentration. No significant consumption of the starting compound was observed in either case over at least 2 h.
(c) TEMPO/DPPH: Compound **1** at 2 mM in methanol was treated with equimolar TEMPO or DPPH, and the mixture was left under vigorous stirring in air. The reaction course was followed by HPLC showing the complete consumption of the starting compound after 1 h with formation of dimers **2a**/**b**. Treatment of the mixture with an additional molar equivalent of TEMPO or DPPH did not result in any significant consumption of **2a**/**b**. Compound **2a**/**b** was treated separately with either reagent under the conditions described for **1**. No appreciable consumption was observed in either case by HPLC analysis.
(d) HCl/H_2_O_2_: Compound **2a**/**b** at 50 μM in methanol/HCl at a 3:1 v/v ratio up to different concentrations of the acid in the range 0.25–3 M was treated with 10 molar equivalents of H_2_O_2_ according to the protocol already developed.^10^ Development of the absorbance at 598 nm for dimer **3** was monitored over 30 min.
(e) *m*-Chloroperbenzoic acid or persulfate/iron: Compound **2a**/**b** at 50 μM in methanol/HCl at a 3:1 v/v ratio was treated with *m*-chloroperbenzoic acid (0.8 equiv) or sodium persulfate/Fe(II) at a 1:1 molar ratio (1.5 equiv). Development of the absorbance at 598 nm for dimer **3** was monitored over 30 min.

### Computational Studies

All calculations were performed with the Gaussian package of programs.^25^ Structures were geometry-optimized at the DFT level, with a hybrid functional (PBE0)^18^ and a reasonably large basis set, 6-31+G(d,p). For radical species, the unrestricted formulation was adopted. For each chemical species, all significant tautomers in the neutral, monoprotonated, and diprotonated state were examined. Extensive conformational explorations were carried out, separately for each of the above conditions, based essentially on relaxed grid searches in torsion angle space. In those cases where conformational enantiomers exist, a single enantiomeric series has been examined. Computations were performed either in vacuo (neutral forms only) or by adoption of a polarizable continuum medium (PCM)^20^ (all neutral, monoprotonated, and diprotonated forms) to account for the influence of the solution environment. In view of the faster convergence, a scaled van der Waals cavity based on universal force field (UFF) radii^26^ was used, and polarization charges were modeled by spherical Gaussian functions;^27^ vibrational–rotational contributions to the free energy were also computed (at 298.15 K, in the rigid rotor-harmonic oscillator approximation). Nonelectrostatic contributions to the solvation free energy were disregarded at this stage; these terms were accounted for in single-point PCM calculations at the M06-2X^19^/6-311++G(2d,2p) level, employing radii and nonelectrostatic terms of the SMD solvation model.^21^

For computation of EPR parameters, geometry optimizations were carried out at the unrestricted DFT level, with the B3LYP functional^28^ and the N07D basis set, as optimized for B3LYP,^29^ either in vacuo or by adoption of a polarizable continuum medium. Single-point calculations were then carried out with the B3LYP functional and specifically tailored basis sets, namely, EPR-II or EPR-III;^30^ the sets were completed for the sulfur center with a 6-31+G(d) or 6-311++G(2d) basis, respectively.

### EPR Spectroscopy

X-Band EPR spectra were collected at 298 K in a CW spectrometer equipped with a variable temperature unit, after mixing a solution (12–25 mM) of dimer **2** in methanol containing 3 M HCl with H_2_O_2_ (0.1–0.4 equiv) in an open (presence of atmospheric oxygen) suprasil quartz tube with 1 mm i.d. To increase the S/N ratio, up to eight spectra were accumulated and digitally averaged. Blank experiments in the absence of H_2_O_2_ did not produce any detectable EPR signal even under continuous photolysis of the mixture in the cavity of the spectrometer with a 500 W Hg lamp. The measured *g*-factor was corrected with respect to that of 2,4,6-tri-*tert*-butylphenoxyl radical (*g* = 2.0046). Optimized hyperfine constants were obtained by interactive fitting of the experimental spectrum with simulated ones, using the Monte Carlo method.^15^ Simulations were performed with WINESR software developed by Prof. Marco Lucarini (University of Bologna). As an initial input for computer simulations, calculated (B3LYP, see Tables S1 and S2) values were used along with literature data for similar structures.

### Oxygen Uptake Measurements

Oxygen consumption measurements were performed in a two-channel oxygen uptake apparatus, based on a Validyne DP 15 differential pressure transducer built in the laboratory.^17^ The oxygen consumption in the sample was measured after calibration of the apparatus from the differential pressure recorded with time between the two channels. Monomer **1** or dimer **2** was incubated in MeOH containing 3 M H_2_SO_4_ (4 mL) at 1–2 mM at 303 K; H_2_O_2_ was added either as a single addition up to 8 mM or in aliquots (0.25 mM equivalent each) at time intervals during the course of oxidation. The reaction was monitored up to 24 h following addition of H_2_O_2_; the extent of conversion to **3** was judged by color changes during the reaction and by spectrophotometry at the end of the reaction.

## Supporting Information Available

UV–vis analysis of the oxidation of dimer **2** with peroxides and hydroperoxides followed by development of the chromophore of dimer **3**, EPR spectrum and oxygen uptake measurement of 3-phenyl-1,4-benzothiazine, UV–vis analysis of substituted anilines, computed free-energy diagram for reagents, products, and putative intermediates in the reaction pathway leading from **2** to **3**; DFT calculations and EPR coupling constants for neutral and protonated radical **1**^·^; computed p*K*_a_ values (PDF)
