# Supplementary material for: Proton-Sensitive Free-Radical Dimer Evolution Is a Critical Control Point for the Synthesis of Δ2,2′-Bibenzothiazines
Source: J Org Chem. 2020 Aug 26;85(17):11440–8. doi: 10.1021/acs.joc.0c01520 (PMC8011920; doi:10.1021/acs.joc.0c01520)
Supplement: Supplementary file 1 — jo0c01520_si_001.pdf [file jo0c01520_si_001.pdf]

Proton-Sensitive Free Radical Dimer Evolution Is a Critical Control Point for the Synthesis of $\Delta^{2,2'}$-Bibenzothiazines.

Luca Valgimigli,[†*] Maria Laura Alfieri,[‡] Riccardo Amorati,[†] Andrea Baschieri,[†] Orlando Crescenzi,[‡] Alessandra Napolitano,[‡*] and Marco d'Ischia[‡]

[†]Department of Chemistry "Giacomo Ciamician", University of Bologna, I-40126 Bologna, Italy
[‡]Department of Chemical Sciences, University of Naples Federico II, I-80126, Naples, Italy

Supporting Information

Content	Page
**Figure S1.** UV-vis analysis of the oxidation of dimer **2** with peroxides and hydroperoxides.	S2
**Figure S2.** Development of the chromophore of dimers **3** at 598 nm from **2**.	S3
**Figure S3.** EPR spectrum of a solution of **1** in acidic MeOH and simulated spectra.	S4
**Figure S4.** Control experiment of oxygen evolution from H_2O_2 in MeOH 3 M H_2SO_4 at 303 K.	S5
**Figure S5.** Oxygen uptake measured by incubating 6.5 μmoles of monomer **1** in 4 mL MeOH 3 M H_2SO_4.	S6
**Figure S6**. UV-vis absorption spectra of substituted anilines in 3:1 methanol/36%HCl.	S7
**Figure S7.** Computed free energy diagram for reagents, products and putative intermediates in the reaction pathway leading from **2** to **3** (reaction path a: see text), under different pH conditions.	S9
**Figure S8.** Computed free energy diagram for reagents, products and putative intermediates in the reaction pathway leading from **2** to **3** (reaction path b: see text) under different pH conditions.	S10
**Table S1.** EPR parameters computed for the predominant forms of *N*-monoprotonated *C*-radicals from single-bond dimers in MeOH.	S11
**Table S2.** EPR parameters computed for the predominant forms of *N*,*N'*-diprotonated *C*-radicals from single-bond dimers in MeOH.	S15
**Table S3.** Calculations on neutral forms in vacuo.	S19
**Table S4.** Calculations on neutral forms in methanol.	S57
**Table S5.** Calculations on monoprotonated forms in methanol.	S95
**Table S6.** Calculations on diprotonated forms in methanol.	S157
**Table S7.** pK_a values computed in methanol at different theory levels for all bibenzothiazine derivatives examined.	S191
**Table S8.** pK_a values computed in methanol at different theory levels for some reference nitrogen bases and corresponding experimental values.	S192

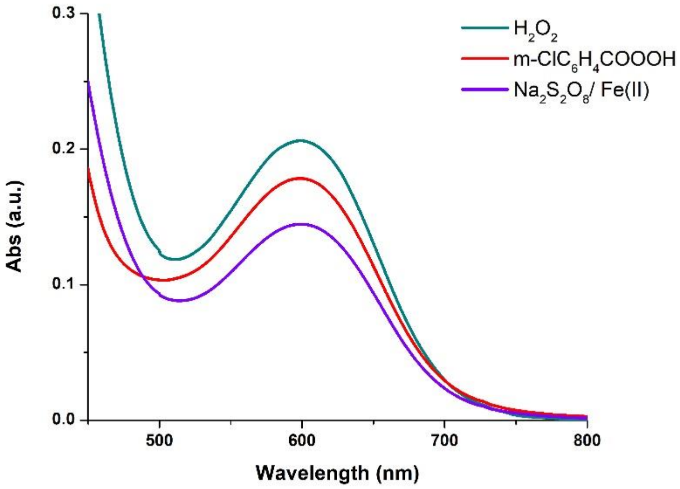

Figure S1. UV-vis analysis of the oxidation of dimer **2** at 50 µM in MeOH:36% HCl 3:1 with hydrogen peroxide (0.4 equiv.), m-chloroperbenzoic acid (m-ClC_6H_4COOOH) (0.8 equiv.) and sodium persulfate/Fe(II) (1.5 equiv) at 5 min reaction time.

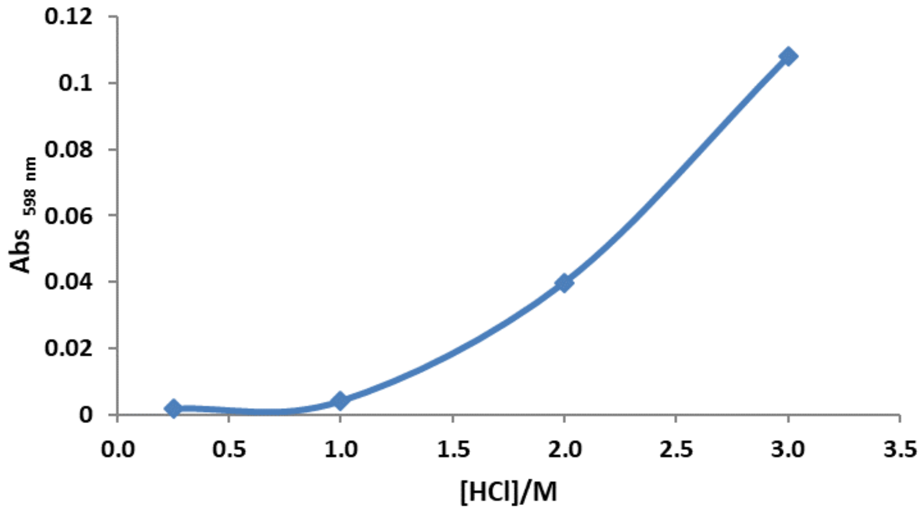

Figure S2. Development of the chromophore of dimers **3** at 598 nm from **2** (50 μM) in methanol / HCl 3:1 at different final HCl concentrations in the presence of H_2O_2 (500 μM) at 30 min.

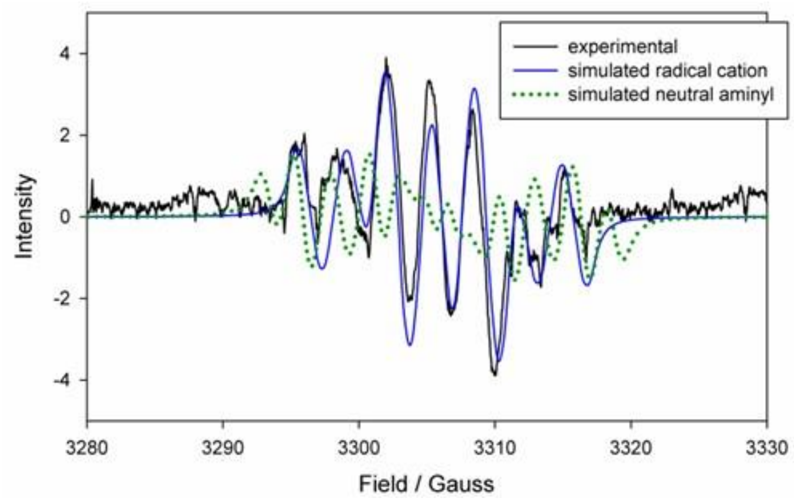

Figure S3. EPR spectrum of a solution of **1** in acidic MeOH and simulated spectra of the neutral and protonated free radical **1·**.

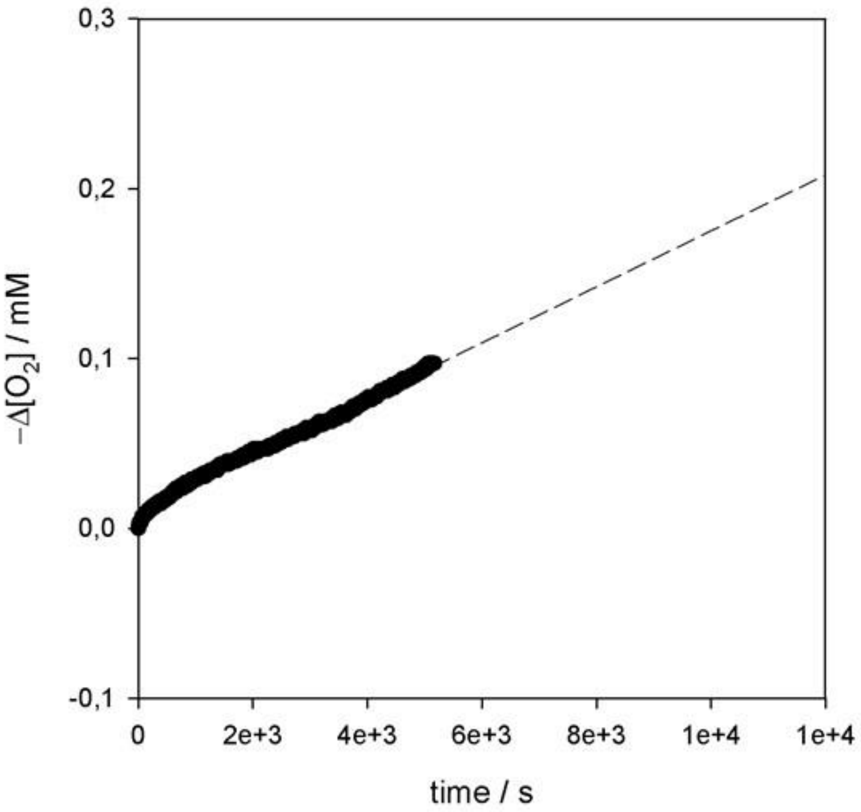

Figure S4. Oxygen evolution measured by adding 10 μmoles of H_2O_2 in 4 mL MeOH 3 M H_2SO_4 at 303 K in the absence of monomer **1** or dimer **2**.

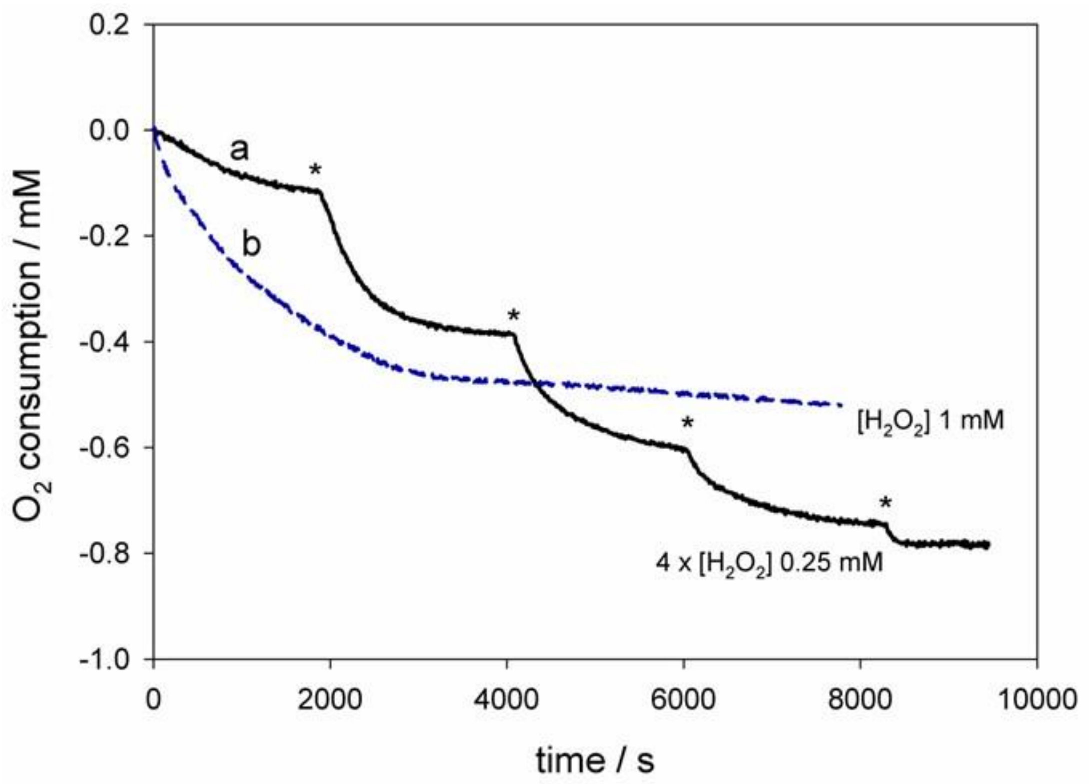

Figure S5. Oxygen uptake measured by incubating 6.5 μmoles of monomer **1** in 4 mL MeOH 3 M H_2SO_4 (final conc. 1.6 mM) at 303 K with (a) addition of aliquots of 1 μmole H_2O_2 to the system at time points indicated by an asterisk, or (b) by single addition of 4 molar equivalents of H_2O_2 (1 mM).

4-nitroaniline	
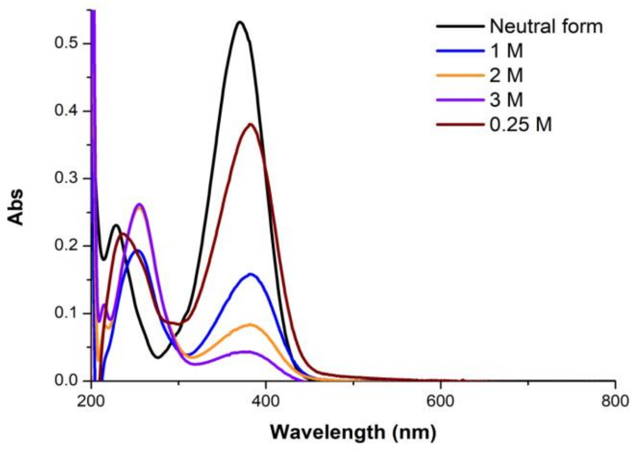	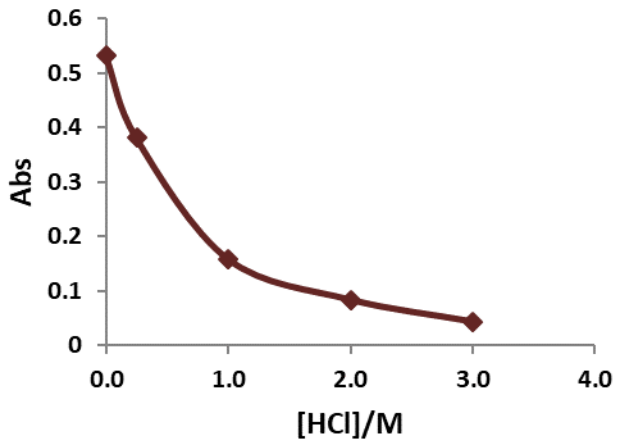
**2-nitroaniline**	
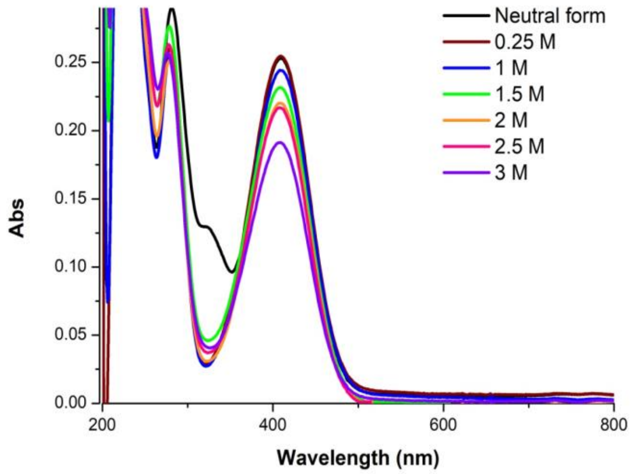	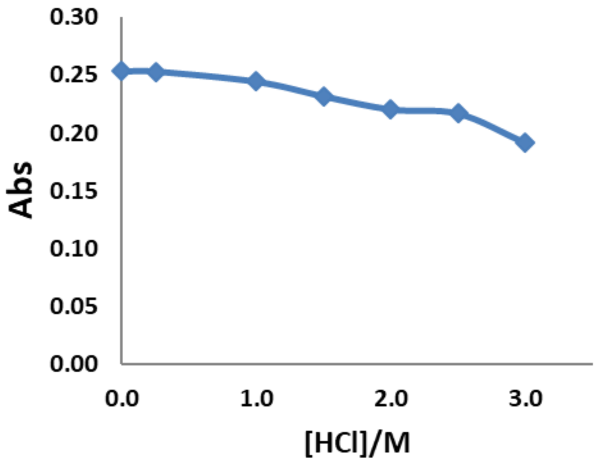

2-nitro-4-chloroaniline

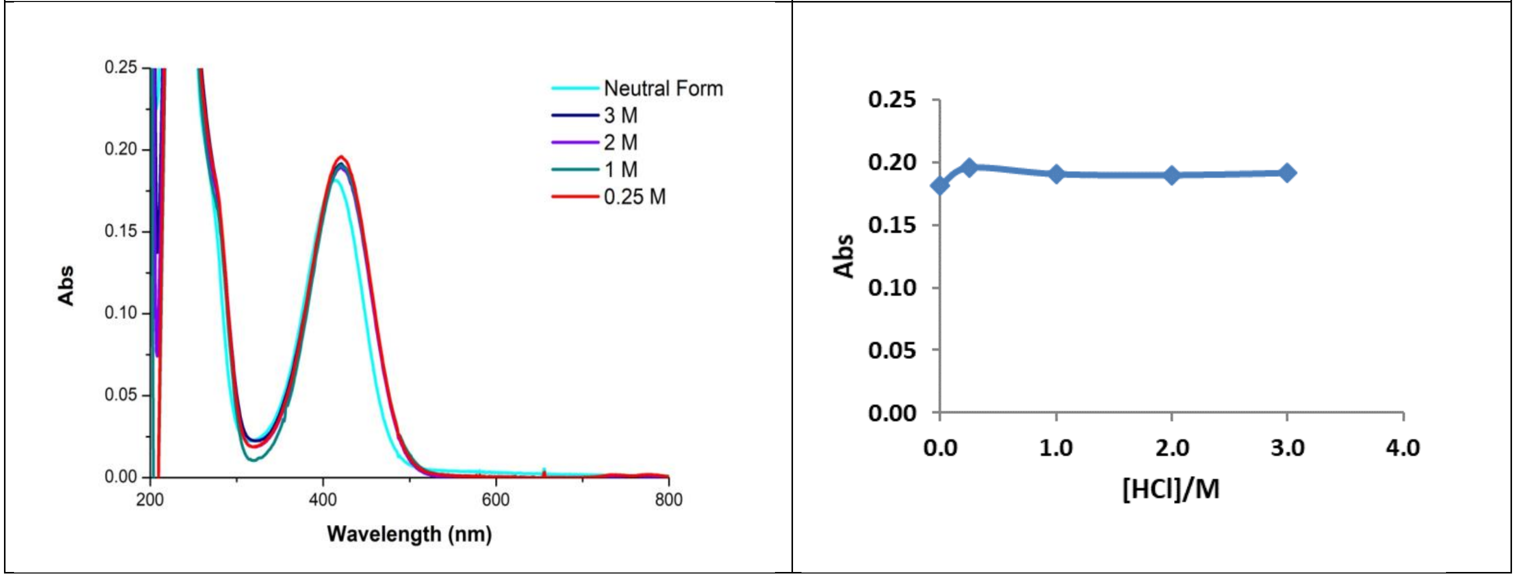

Figure S6. Left: Absorption spectra of substituted anilines in 3:1 methanol/HCl at the concentrations shown. Right: Absorbance at maximum *vs* HCl concentration. Choice of anilines was based on their pK_as in methanol as reported by Rived, F.; Roses, M.; Bosch, E. *Anal. Chim. Acta* **1998**, *374*, 309-324.

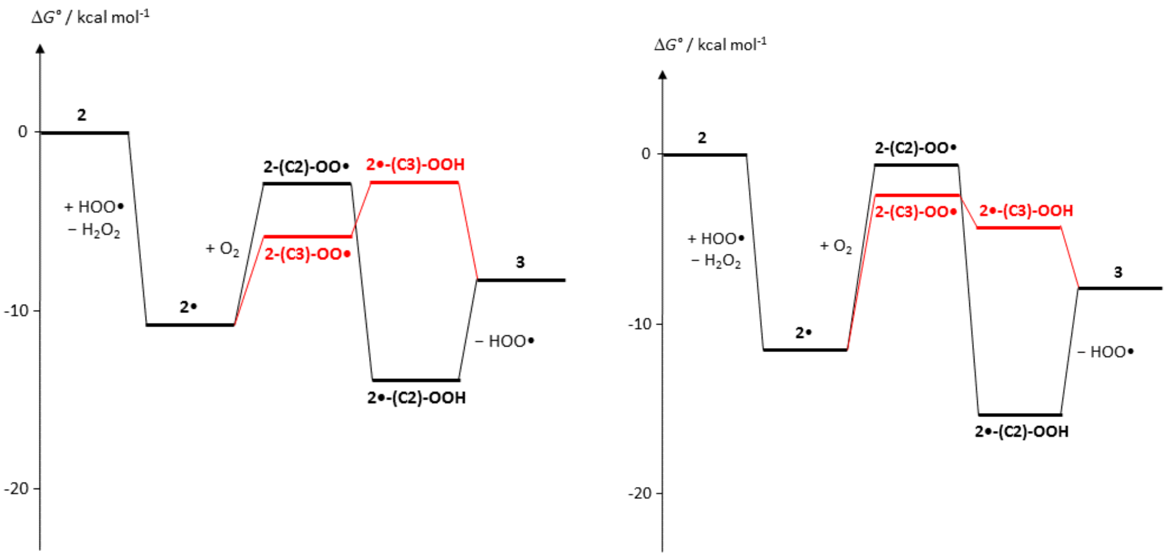

Figure S7. Computed free energy diagram for reagents, products and putative intermediates in the reaction pathway leading from **2** to **3** (reaction path a: see text), under different pH conditions. Left panel, pH 6.0; right panel, pH 0.0. The graphs at pH 3.0 and −1.5 are shown in the main text.

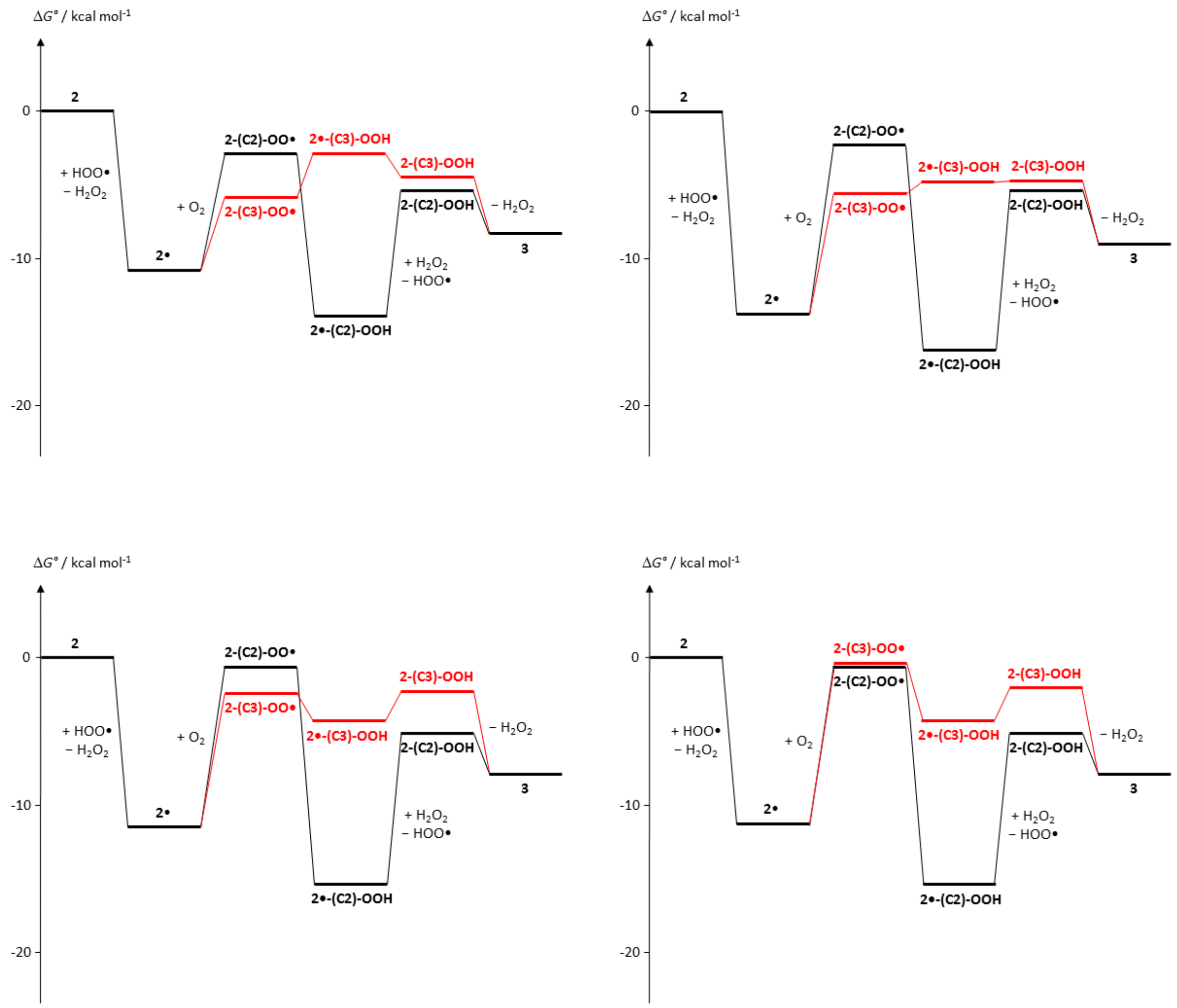

Figure S8. Computed free energy diagram for reagents, products and putative intermediates in the reaction pathway leading from **2** to **3** (reaction path b: see text) under different pH conditions. Top left panel, pH 6.0; top right panel, pH 3.0; bottom left panel, pH 0.0; bottom right panel, pH −1.5.

Table S1. EPR parameters computed for the predominant forms of *N*-monoprotonated *C*-radicals from single-bond dimers in MeOH.

**SOMO**[a]	**Spin density**[b]	**Statistical weight**	***hcc* / Gauss**[c] **B3LYP/EPR-II**	***hcc* / Gauss**[c] **B3LYP/EPR-III**
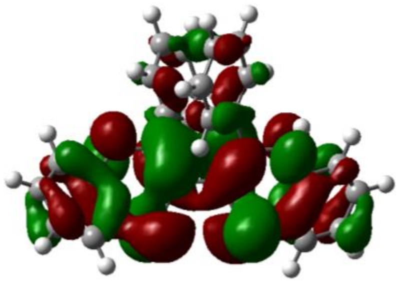	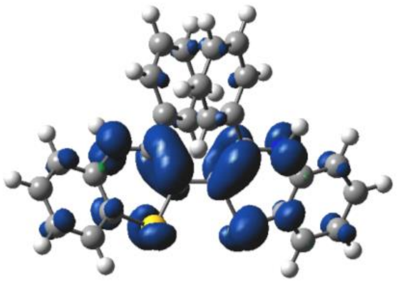	0.36	S1 5.17<br>C2 0.25<br>C3 4.33<br>N4 1.87<br>C5 1.46<br>C6 -0.91<br>C7 1.51<br>C8 0.76<br>C8a 4.67<br>C4a -2.04<br>C-Ph,*ipso* -1.67<br>C-Ph,*ortho* 0.69<br>C-Ph,*meta* -0.62<br>C-Ph,*para* 0.67<br>C-Ph,*meta* -0.45<br>C-Ph,*ortho* 0.96<br>H5 -0.76<br>H6 0.31<br>H7 -0.76<br>H8 0.33<br>H-Ph,*ortho* -0.21<br>H-Ph,*para* -0.57<br>H-Ph,*meta* 0.35<br>H-Ph,*ortho* -0.49<br>S1’ 5.17<br>C2’ 0.25<br>C3’ 4.33<br>N4’ 1.87<br>C5’ 1.46<br>C6’ -0.91<br>C7’ 1.51<br>C8’ 0.76<br>C8a’ 4.67<br>C4a’ -2.04<br>C-Ph’,*ipso* -1.67<br>C-Ph’,*ortho* 0.69	S1 5.52<br>C2 0.21<br>C3 4.45<br>N4 1.88<br>C5 1.39<br>C6 -0.83<br>C7 1.45<br>C8 0.87<br>C8a 4.52<br>C4a -1.85<br>C-Ph,*ipso* -1.56<br>C-Ph,*ortho* 0.66<br>C-Ph,*meta* -0.57<br>C-Ph,*para* 0.64<br>C-Ph,*meta* -0.43<br>C-Ph,*ortho* 0.96<br>H5 -0.72<br>H6 0.30<br>H7 -0.73<br>H8 0.32<br>H-Ph,*para* -0.56<br>H-Ph,*meta* 0.35<br>H-Ph,*ortho* -0.48<br>S1’ 5.52<br>C2’ 0.21<br>C3’ 4.45<br>N4’ 1.88<br>C5’ 1.39<br>C6’ -0.83<br>C7’ 1.45<br>C8’ 0.87<br>C8a’ 4.52<br>C4a’ -1.85<br>C-Ph’,*ipso* -1.56<br>C-Ph’,*ortho* 0.66<br>C-Ph’,*meta* -0.57

			C-Ph’,*meta* -0.62<br>C-Ph’,*para* 0.67<br>C-Ph’,*meta* -0.45<br>C-Ph’,*ortho* 0.96<br>H5’ -0.76<br>H6’ 0.31<br>H7’ -0.76<br>H8’ 0.33<br>H-Ph’,*ortho* -0.21<br>H-Ph’,*para* -0.57<br>H-Ph’,*meta* 0.35<br>H-Ph’,*ortho* -0.49<br>H4’ -3.83<br>H4 -3.83	C-Ph’,*para* 0.64<br>C-Ph’,*meta* -0.43<br>C-Ph’,*ortho* 0.96<br>H5’ -0.72<br>H6’ 0.30<br>H7’ -0.73<br>H8’ 0.32<br>H-Ph’,*para* -0.56<br>H-Ph’,*meta* 0.35<br>H-Ph’,*ortho* -0.48<br>H4’ -3.75<br>H4 -3.75
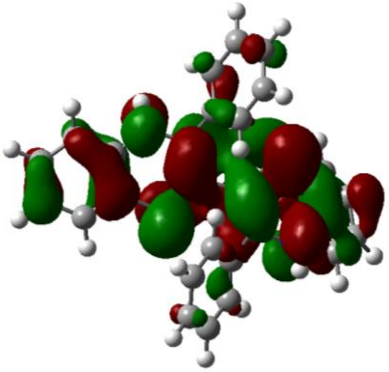	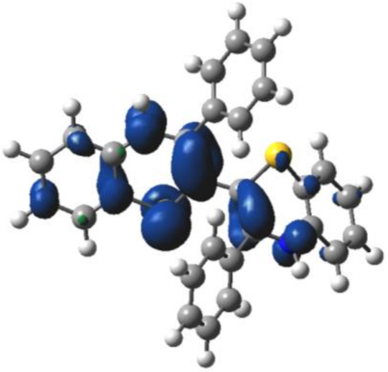	0.30	S1 3.87<br>C2 2.70<br>C3 -1.90<br>N4 3.60<br>C5 0.88<br>C6 -0.93<br>C7 1.55<br>C8 -1.94<br>C8a 1.26<br>C4a -2.63<br>C-Ph,*ipso* -1.57<br>C-Ph,*ortho* 0.70<br>C-Ph,*meta* -0.41<br>C-Ph,*para* 0.35<br>C-Ph,*ortho* 1.46<br>H5 -0.95<br>H7 -1.51<br>H8 0.32<br>H-Ph,*ortho* -0.21<br>H-Ph,*meta* 0.23<br>H-Ph,*para* -0.26<br>H-Ph,*meta* 0.21<br>H-Ph,*ortho* -0.21<br>S1’ 2.49<br>C2’ -1.79<br>C3’ 2.85	S1 2.79<br>C2 2.61<br>C3 -1.64<br>N4 3.64<br>C5 0.81<br>C6 -0.81<br>C7 1.42<br>C8 -1.77<br>C8a 1.13<br>C4a -2.38<br>C-Ph,*ipso* -1.51<br>C-Ph,*ortho* 0.71<br>C-Ph,*meta* -0.40<br>C-Ph,*para* 0.34<br>C-Ph,*ortho* 1.48<br>H5 -0.90<br>H7 -1.48<br>H8 0.30<br>H-Ph,*meta* 0.24<br>H-Ph,*para* -0.26<br>H-Ph,*meta* 0.21<br>H-Ph,*ortho* -0.21<br>S1’ 2.70<br>C2’ -1.82<br>C3’ 2.86<br>N4’ 1.12

			N4’ 1.15<br>C5’ 1.09<br>C6’ -0.51<br>C7’ 0.83<br>C8’ 0.27<br>C8a’ 2.56<br>C4a’ -0.49<br>C-Ph’,*ortho* 0.49<br>C-Ph’,*ortho* 0.46<br>H5’ -0.47<br>H6’ 0.23<br>H7’ -0.43<br>H-Ph’,*meta* 0.21<br>H4’ -2.14<br>H4 -6.19	C5’ 1.00<br>C6’ -0.44<br>C7’ 0.76<br>C8’ 0.30<br>C8a’ 2.38<br>C4a’ -0.38<br>C-Ph’,*ortho* 0.49<br>C-Ph’,*ortho* 0.45<br>H5’ -0.44<br>H6’ 0.22<br>H7’ -0.40<br>H-Ph’,*meta* 0.21<br>H4’ -2.03<br>H4 -6.11
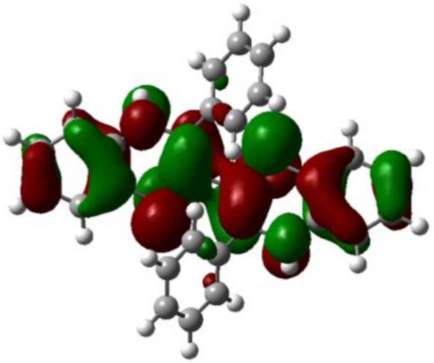	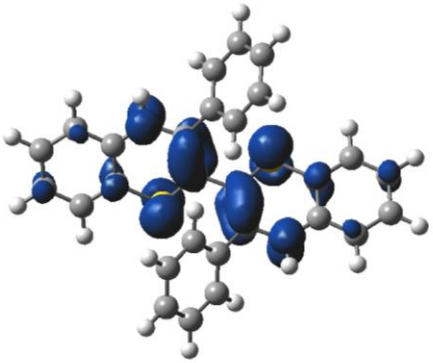	0.34	S1 2.42<br>C3 -0.81<br>N4 2.47<br>C5 0.76<br>C6 -0.67<br>C7 0.98<br>C8 -1.30<br>C8a 0.43<br>C4a -1.33<br>C-Ph,*ipso* -1.19<br>C-Ph,*ortho* 0.83<br>H5 -0.67<br>H7 -0.99<br>S1’ 2.42<br>C3’ -0.81<br>N4’ 2.47<br>C5’ 0.76<br>C6’ -0.67<br>C7’ 0.98<br>C8’ -1.30<br>C8a’ 0.43<br>C4a’ -1.33<br>C-Ph’,*ipso* -1.19<br>C-Ph’,*ortho* 0.83<br>H5’ -0.67	S1 1.59<br>C3 -0.62<br>N4 2.47<br>C5 0.70<br>C6 -0.59<br>C7 0.89<br>C8 -1.18<br>C8a 0.35<br>C4a -1.14<br>C-Ph,*ipso* -1.14<br>C-Ph,*ortho* 0.22<br>C-Ph,*ortho* 0.84<br>H5 -0.63<br>H7 -0.96<br>S1’ 1.59<br>C3’ -0.62<br>N4’ 2.47<br>C5’ 0.70<br>C6’ -0.59<br>C7’ 0.89<br>C8’ -1.18<br>C8a’ 0.35<br>C4a’ -1.14<br>C-Ph’,*ipso* -1.14<br>C-Ph’,*ortho* 0.84

			H7’ -0.99<br>H4’ -4.19<br>H4 -4.19	C-Ph’,*ortho* 0.22<br>H5’ -0.63<br>H7’ -0.96<br>H4’ -4.10<br>H4 -4.10

[a] Isovalue 0.02 a.u.

[b] Isodensity 0.002 a.u.

[c] Isotopes: ^{1}H, ^{13}C, ^{14}N, ^{33}S. Only couplings whose absolute value exceeds 0.2 Gauss are listed. The numbering system is shown in the following scheme:

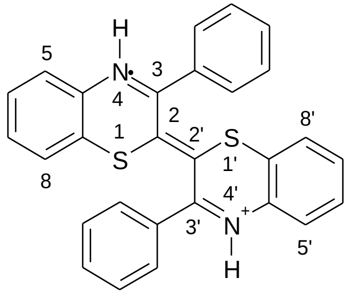

Table S2. EPR parameters computed for the predominant forms of *N*,*N'*-diprotonated *C*-radicals from single-bond dimers in MeOH.

SOMO[a]	Spin density[b]	Statistical weight	*hcc* / Gauss[c] B3LYP/EPR-II	*hcc* / Gauss[c] B3LYP/EPR-III
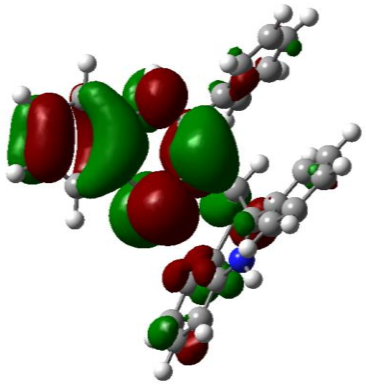	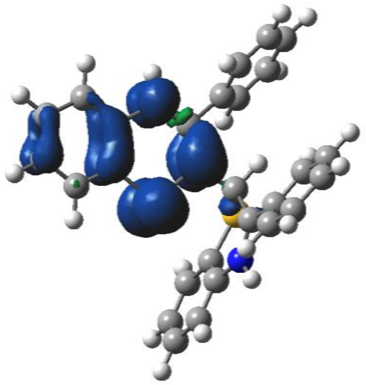	0.54	S1 6.41<br>C2 7.78<br>C3 -5.54<br>N4 4.11<br>C5 -0.41<br>C7 1.46<br>C8 -2.13<br>C8a 1.32<br>C4a -2.73<br>C-Ph *ortho* 0.34<br>C-Ph *ortho* -0.29<br>H4 -6.99<br>H5 -0.65<br>H6 -0.81<br>H7 -1.74<br>S1' 1.36<br>C2' -4.17<br>C3' 2.41<br>C5' -0.26<br>C6' 0.30<br>C7' -0.30<br>C8' 0.46<br>C8a' -0.29<br>C4a' 0.32<br>C-Ph',*ipso* -0.29<br>C-Ph',*ortho* 0.22<br>H2' 0.57<br>H4' 0.51<br>H6' -0.22<br>H8' -0.27	S1 4.99<br>C2 7.31<br>C3 -5.04<br>N4 4.10<br>C5 -0.42<br>C7 1.30<br>C8 -1.88<br>C8a 1.20<br>C4a -2.47<br>C-Ph *ipso* -0.23<br>C-Ph *ortho* 0.41<br>C-Ph *ortho* -0.22<br>H4 -6.83<br>H5 -0.59<br>H6 -0.85<br>H7 -1.67<br>S1' 1.14<br>C2' -3.86<br>C3' 2.31<br>C5' -0.26<br>C6' 0.29<br>C7' -0.28<br>C8' 0.43<br>C8a' -0.26<br>C4a' 0.31<br>C-Ph',*ipso* -0.27<br>C-Ph',*ortho* 0.21<br>H2' 0.54<br>H4' 0.51<br>H6' -0.23<br>H8' -0.27

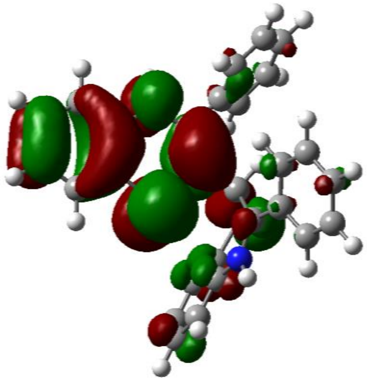	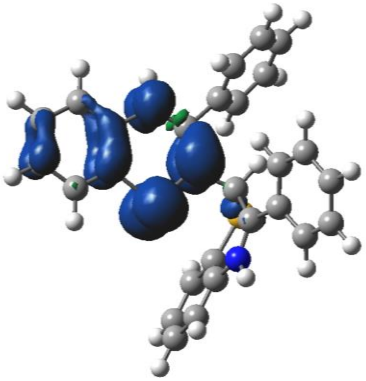	0.43	S1 6.38<br>C2 7.40<br>C3 -5.34<br>N4 4.09<br>C5 -0.39<br>C7 1.46<br>C8 -2.14<br>C8a 1.27<br>C4a -2.74<br>C-Ph *ipso* -0.25<br>C-Ph *ortho* 0.32<br>C-Ph *ortho* -0.27<br>H4 -7.00<br>H5 -0.66<br>H6 -0.81<br>H7 -1.74<br>S1’ 1.71<br>C2’ -4.18<br>C3’ 1.61<br>C5’ -0.32<br>C6’ 0.32<br>C7’ -0.31<br>C8’ 0.46<br>C8a’ -0.35<br>C4a’ 0.41<br>C-Ph’,*ipso* -0.26<br>C-Ph’,*ortho* 0.47<br>C-Ph’,*para* 0.21<br>H2’ 1.10<br>H4’ 0.59<br>H6’ -0.22<br>H8’ -0.26	S1 5.00<br>C2 6.97<br>C3 -4.85<br>N4 4.09<br>C5 -0.40<br>C7 1.30<br>C8 -1.89<br>C8a 1.15<br>C4a -2.49<br>C-Ph *ipso* -0.29<br>C-Ph *ortho* 0.39<br>H4 -6.85<br>H5 -0.60<br>H6 -0.84<br>H7 -1.67<br>S1’ 1.44<br>C2’ -3.88<br>C3’ 1.54<br>C5’ -0.31<br>C6’ 0.31<br>C7’ -0.29<br>C8’ 0.44<br>C8a’ -0.32<br>C4a’ 0.39<br>C-Ph’,*ipso* -0.23<br>C-Ph’,*ortho* 0.47<br>H4’ 0.58<br>H2’ 1.06<br>H6’ -0.22<br>H8’ -0.26

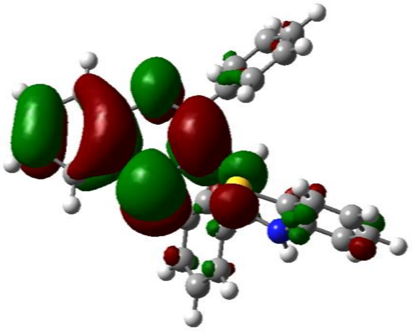	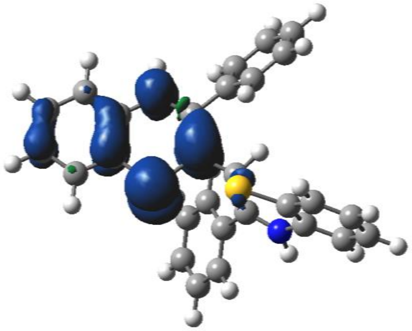	0.02	S1 6.53<br>C2 6.75<br>C3 -5.39<br>N4 4.09<br>C5 -0.47<br>C7 1.46<br>C8 -2.10<br>C8a 1.31<br>C4a -2.66<br>C-Ph *ortho* -0.30<br>C-Ph *ortho* 0.31<br>H4 -7.01<br>H5 -0.62<br>H6 -0.85<br>H7 -1.76<br>S1’ 0.79<br>C2’ -3.95<br>C3’ 2.19<br>C8’ 0.37<br>C8a’ -0.56<br>C-Ph’,*ortho* 0.60<br>C-Ph’,*para* 0.21<br>C-Ph’,*ortho* 0.22<br>H2’ 0.84<br>H4’ 0.64	S1 4.88<br>C2 6.31<br>C3 -4.86<br>N4 4.09<br>C5 -0.46<br>C7 1.32<br>C8 -1.86<br>C8a 1.19<br>C4a -2.42<br>C-Ph *ortho* -0.23<br>C-Ph *ortho* 0.38<br>H4 -6.85<br>H5 -0.58<br>H6 -0.88<br>H7 -1.69<br>S1’ 0.37<br>C2’ -3.68<br>C3’ 2.05<br>C8’ 0.35<br>C8a’ -0.55<br>C-Ph’,*ortho* 0.59<br>C-Ph’,*para* 0.21<br>C-Ph’,*ortho* 0.22<br>H2’ 0.83<br>H4’ 0.64
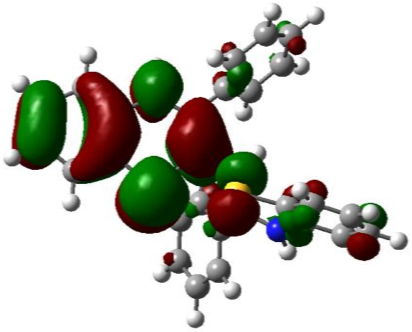	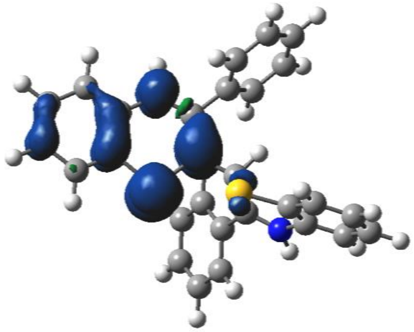	0.01	S1 6.53<br>C2 6.83<br>C3 -5.57<br>N4 4.06<br>C5 -0.47<br>C7 1.48<br>C8 -2.19<br>C8a 1.22<br>C4a -2.66<br>C-Ph *ortho* -0.37<br>C-Ph *ortho* 0.28<br>H4 -7.00<br>H5 -0.63<br>H6 -0.85<br>H7 -1.78	S1 4.95<br>C2 6.38<br>C3 -5.05<br>N4 4.05<br>C5 -0.47<br>C7 1.33<br>C8 -1.95<br>C8a 1.10<br>C4a -2.41<br>C-Ph *ipso* -0.24<br>C-Ph *ortho* -0.30<br>C-Ph *ortho* 0.35<br>H4 -6.84<br>H5 -0.58<br>H6 -0.88

			H8 0.21<br>S1’ 0.64<br>C2’ -3.09<br>C3’ 2.00<br>C5’ -0.21<br>C6’ 0.25<br>C7’ -0.24<br>C8’ 0.38<br>C8a’ -0.71<br>C4a’ 0.27<br>C-Ph’,*ortho* 0.46<br>H2’ 0.47<br>H4’ 0.60	H7 -1.71<br>C2’ -2.85<br>C3’ 1.88<br>C5’ -0.21<br>C6’ 0.25<br>C7’ -0.24<br>C8’ 0.36<br>C8a’ -0.70<br>C4a’ 0.27<br>C-Ph’,*ortho* 0.43<br>H2’ 0.44<br>H4’ 0.59

[a] Isovalue 0.02 a.u.

[b] Isodensity 0.002 a.u.

[c] Isotopes: ^{1}H, ^{13}C, ^{14}N, ^{33}S. Only couplings whose absolute value exceeds 0.2 Gauss are listed. The numbering system is shown in the following scheme:

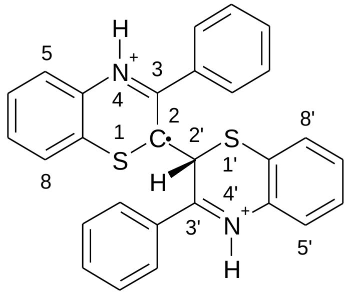

Table S3. Neutral forms in vacuo. For each chemical species, values are reported for the most stable conformer / geometric isomer identified. $G_{\mathrm{RRHO,calc}}$ [M062X / 6-311++G(2d,2p)] = E_{el} [M062X / 6-311++G(2d,2p) // PBE0 / 6-31+G(d,p)] - E_{el} [PBE0 / 6-31+G(d,p)] + G_{RRHO} [PBE0 / 6-31+G(d,p)]; and likewise for $G_{\mathrm{RRHO,calc}}$ [M062X / 6-31+G(d,p)]. In this and in the following tables, G values are referred to a 1 atm standard state; however, they were converted to a 1 M standard state in order to compute the reaction free energies discussed in the paper (see *e.g.* Cramer, C. J. *Essentials of Computational Chemistry: Theories and Models*, 2nd edition, John Wiley & Sons, Chichester, 2004, pp 378-379).

**Species**	**PBE0 / 6-31+G(d,p)**			**M062X / 6-31+G(d,p)**		**M062X / 6-311++G(2d,2p)**		
**Cartesian coordinates / Å**	**E_{el} / Ha**	**H_{RRHO} / Ha**	**G_{RRHO} / Ha**	**E_{el} / Ha**	**$G_{\mathrm{RRHO,calc}}$ / Ha**	**E_{el} / Ha**	**$G_{\mathrm{RRHO,calc}}$ / Ha**	**$\Delta G_{\mathrm{RRHO,calc}}$ / kcal mol^{-1}**
**Single-bond dimer**								
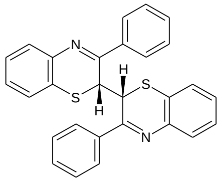<br>S -1.00031 -2.46028 1.24651<br>C -0.10319 -0.97711 0.71763<br>C -0.80252 0.23685 1.30911<br>N -2.07630 0.40335 1.22142<br>C -4.19650 -0.18781 0.28865<br>C -5.13026 -1.12711 -0.12414<br>C -4.80999 -2.48689 -0.09351<br>C -3.54723 -2.89635 0.31990<br>C -2.59379 -1.95261 0.71077<br>C -2.91690 -0.57776 0.71628<br>C -0.00880 1.30619 1.95755<br>C -0.59399 2.57071 2.13524<br>C 0.10810 3.59810 2.74886<br>C 1.40889 3.38449 3.20787<br>C 1.99907 2.13502 3.04208<br>C 1.30022 1.10602 2.41606<br>H 0.89390 -1.09589 1.14423<br>H -4.43314 0.87212 0.31034<br>H -6.11371 -0.80523 -0.45354<br>H -5.54141 -3.22879 -0.40015<br>H -3.28692 -3.95129 0.32490<br>H -1.60597 2.72380 1.77603<br>H -0.35824 4.57200 2.86814<br>H 1.95799 4.18824 3.69027<br>H 3.00899 1.95545 3.39941<br>H 1.79088 0.14413 2.30793<br>S 0.89007 -2.29413 -1.55185<br>C 0.04076 -0.86660 -0.82290<br>C 0.80768 0.37981 -1.24277<br>N 2.08276 0.47322 -1.08572	-1983.28470	-1982.85166	-1982.93600	-1984.41474	-1984.06604	-1984.76490	-1984.41619	1.1

C 4.12949 -0.33778 -0.15019<br>C 5.00671 -1.36905 0.15161<br>C 4.63928 -2.69373 -0.10147<br>C 3.38228 -2.98089 -0.62153<br>C 2.48309 -1.94775 -0.89902<br>C 2.85885 -0.60279 -0.68618<br>C 0.09495 1.53755 -1.83178<br>C 0.84521 2.63946 -2.27646<br>C 0.22178 3.74425 -2.83744<br>C -1.16788 3.77610 -2.97020<br>C -1.92320 2.69229 -2.53340<br>C -1.29899 1.58212 -1.96931<br>H -0.95166 -0.88687 -1.27626<br>H 4.40384 0.70176 0.00369<br>H 5.98462 -1.14434 0.56707<br>H 5.32824 -3.50445 0.11649<br>H 3.08291 -4.01068 -0.79661<br>H 1.92392 2.60199 -2.16888<br>H 0.82025 4.58535 -3.17609<br>H -1.65605 4.64015 -3.41224<br>H -3.00514 2.70555 -2.62905<br>H -1.92341 0.76338 -1.62805								
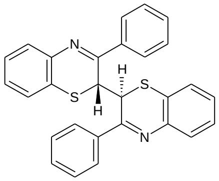<br><br>S 0.97930 -0.84050 -1.81970<br>C -0.29150 -0.38777 -0.60122<br>C -0.99478 -1.66771 -0.17601<br>N -0.32942 -2.69391 0.22759<br>C 1.72257 -3.72171 0.90883<br>C 3.07836 -3.96049 0.74071<br>C 3.79024 -3.27461 -0.24813<br>C 3.14999 -2.32358 -1.03375<br>C 1.79178 -2.05440 -0.84480<br>C 1.05021 -2.77602 0.11711<br>C -2.47275 -1.76654 -0.20009<br>C -3.08309 -2.91637 0.32966<br>C -4.46257 -3.06001 0.31659<br>C -5.26900 -2.06229 -0.23522<br>C -4.67814 -0.92081 -0.76753<br>C -3.29314 -0.77124 -0.74757<br>H -0.96247 0.27143 -1.15333<br>H 1.13963 -4.27119 1.64214<br>H 3.57977 -4.69513 1.36380<br>H 4.84716 -3.47349 -0.39976<br>H 3.70559 -1.76500 -1.78193	-1983.28475	-1982.85171	-1982.93580	-1984.41618	-1984.06723	-1984.76686	-1984.41791	0.0

H -2.44632 -3.68741 0.74977<br>H -4.91405 -3.95467 0.73620<br>H -6.34927 -2.17663 -0.24929<br>H -5.29259 -0.13674 -1.20052<br>H -2.87279 0.13704 -1.16533<br>S -0.97930 0.84050 1.81970<br>C 0.29150 0.38777 0.60122<br>C 0.99478 1.66771 0.17601<br>N 0.32942 2.69391 -0.22759<br>C -1.72257 3.72171 -0.90883<br>C -3.07836 3.96049 -0.74071<br>C -3.79024 3.27461 0.24813<br>C -3.14999 2.32358 1.03375<br>C -1.79178 2.05440 0.84480<br>C -1.05021 2.77602 -0.11711<br>C 2.47275 1.76654 0.20009<br>C 3.08309 2.91637 -0.32966<br>C 4.46257 3.06001 -0.31659<br>C 5.26900 2.06229 0.23522<br>C 4.67814 0.92081 0.76753<br>C 3.29314 0.77124 0.74757<br>H 0.96247 -0.27143 1.15333<br>H -1.13963 4.27119 -1.64214<br>H -3.57977 4.69513 -1.36380<br>H -4.84716 3.47349 0.39976<br>H -3.70559 1.76500 1.78193<br>H 2.44632 3.68741 -0.74977<br>H 4.91405 3.95467 -0.73620<br>H 6.34927 2.17663 0.24929<br>H 5.29259 0.13674 1.20052<br>H 2.87279 -0.13704 1.16533								
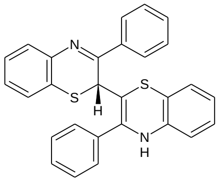<br>S 1.62853 1.63209 -1.72647<br>C 0.46911 1.02214 -0.44100<br>C 1.16862 1.07328 0.91527<br>N 2.39692 0.75033 1.10706<br>C 4.42611 -0.30501 0.43460<br>C 5.37176 -0.64842 -0.52094<br>C 5.17321 -0.28852 -1.85591<br>C 4.01950 0.39191 -2.22947<br>C 3.05834 0.72289 -1.27189<br>C 3.25781 0.38596 0.08181<br>C 0.37750 1.49666 2.09772<br>C 1.03039 1.67220 3.32859	-1983.27699	-1982.84361	-1982.92760	-1984.40550	-1984.05610	-1984.75711	-1984.40772	6.4

C 0.32704 2.06989 4.45697<br>C -1.04817 2.29917 4.38509<br>C -1.70861 2.12476 3.17256<br>C -1.00351 1.72851 2.03844<br>H -0.30414 1.79267 -0.45408<br>H 4.56279 -0.55781 1.48178<br>H 6.26793 -1.18731 -0.22800<br>H 5.91283 -0.54523 -2.60873<br>H 3.85135 0.65832 -3.26940<br>H 2.09892 1.48993 3.37129<br>H 0.85257 2.20467 5.39833<br>H -1.59853 2.61174 5.26812<br>H -2.77912 2.29683 3.10384<br>H -1.55204 1.59020 1.11229<br>S 0.77951 -1.78109 -0.54999<br>C -0.18429 -0.29543 -0.74794<br>C -1.47375 -0.41509 -1.15100<br>N -2.07079 -1.67216 -1.26947<br>C -2.76389 -3.61792 0.02634<br>C -2.44187 -4.64738 0.90817<br>C -1.15168 -4.75472 1.42111<br>C -0.17542 -3.83663 1.03487<br>C -0.47877 -2.83810 0.11325<br>C -1.78212 -2.71329 -0.38081<br>C -2.37473 0.72162 -1.46021<br>C -3.62862 0.81863 -0.83776<br>C -4.49098 1.86787 -1.14199<br>C -4.11807 2.83059 -2.07936<br>C -2.88051 2.73599 -2.71260<br>C -2.01541 1.68750 -2.40925<br>H -3.77784 -3.51960 -0.35536<br>H -3.20867 -5.35757 1.20313<br>H -0.90175 -5.54840 2.11829<br>H 0.83471 -3.90566 1.42819<br>H -3.91476 0.08006 -0.09225<br>H -5.45306 1.93744 -0.64217<br>H -4.79177 3.64854 -2.31766<br>H -2.58922 3.47506 -3.45338<br>H -1.06207 1.59805 -2.92306<br>H -3.01351 -1.65951 -1.62883								
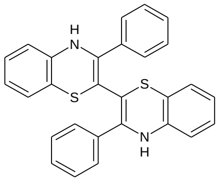<br>S -0.75790 -2.44628 -0.90918<br>C -0.39521 -0.68772 -0.90759<br>C -1.38326 0.17930 -1.23713	-1983.27662	-1982.84278	-1982.92647	-1984.40714	-1984.05699	-1984.76031	-1984.41017	4.9

N -2.69468 -0.27251 -1.39516<br>C -4.56123 -1.29323 -0.20854<br>C -5.06618 -2.33516 0.56585<br>C -4.23367 -3.37595 0.97065<br>C -2.89484 -3.38058 0.57968<br>C -2.39709 -2.36969 -0.23992<br>C -3.22637 -1.30497 -0.61853<br>C -1.19810 1.63802 -1.40766<br>C -2.10409 2.53713 -0.82773<br>C -1.96100 3.90970 -1.01293<br>C -0.91165 4.40479 -1.78354<br>C -0.00545 3.51783 -2.36535<br>C -0.14774 2.14641 -2.18365<br>H -5.20529 -0.46728 -0.50245<br>H -6.11072 -2.32265 0.86303<br>H -4.62124 -4.18401 1.58343<br>H -2.23455 -4.18780 0.88436<br>H -2.90346 2.16141 -0.19325<br>H -2.66563 4.59226 -0.54600<br>H -0.80096 5.47511 -1.93325<br>H 0.81144 3.89494 -2.97423<br>H 0.55346 1.46103 -2.65035<br>S 2.21676 -0.86849 -1.67287<br>C 0.96485 -0.30855 -0.53289<br>C 1.31349 0.44074 0.53668<br>N 2.65499 0.78763 0.77789<br>C 4.83082 -0.15013 1.31949<br>C 5.86603 -1.03990 1.04411<br>C 5.76036 -1.93033 -0.02063<br>C 4.61597 -1.91585 -0.81699<br>C 3.59889 -0.99652 -0.57398<br>C 3.69388 -0.11358 0.50903<br>C 0.35635 0.97013 1.53438<br>C -0.59990 0.14077 2.13377<br>C -1.47207 0.64956 3.09102<br>C -1.39801 1.98966 3.47108<br>C -0.44644 2.82033 2.88413<br>C 0.42545 2.31533 1.92221<br>H 4.90506 0.52892 2.16614<br>H 6.74995 -1.04243 1.67503<br>H 6.55840 -2.63515 -0.23192<br>H 4.51408 -2.61033 -1.64617<br>H -0.64971 -0.90650 1.85168<br>H -2.20656 -0.00791 3.54781<br>H -2.07746 2.38282 4.22212<br>H -0.38701 3.86789 3.16613<br>H 1.13951 2.97954 1.44092<br>H 2.77666 1.27434 1.65501<br>H -3.35268 0.43108 -1.69456								
**Single-bond dimer, radical**								

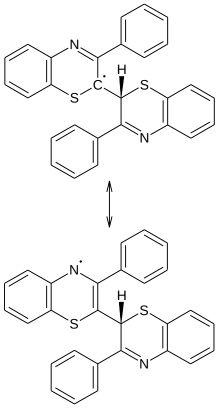<br>S 0.81531 1.55945 -0.49776<br>C -0.29816 0.46535 0.27051<br>C -1.64585 0.81120 0.47341<br>N -2.25695 1.91317 0.04335<br>C -2.34973 4.02498 -1.02549<br>C -1.76603 5.09304 -1.68161<br>C -0.39273 5.08231 -1.95709<br>C 0.38143 3.99495 -1.57650<br>C -0.20808 2.91229 -0.91708<br>C -1.59021 2.90583 -0.62068<br>C -2.56351 -0.11994 1.19863<br>C -2.28781 -0.57410 2.49288<br>C -3.18908 -1.40397 3.15691<br>C -4.37264 -1.79313 2.53369<br>C -4.66050 -1.33364 1.24892<br>C -3.76711 -0.49456 0.58999<br>H -3.40974 4.00981 -0.79132<br>H -2.37306 5.94262 -1.98015<br>H 0.07103 5.91996 -2.46909<br>H 1.44677 3.97800 -1.79204<br>H -1.37796 -0.25348 2.99372<br>H -2.96686 -1.74057 4.16557<br>H -5.07242 -2.44394 3.05023<br>H -5.58752 -1.62339 0.76189<br>H -3.99593 -0.11011 -0.39971<br>S 1.27268 -0.86380 2.20445<br>C 0.28408 -0.86524 0.65524<br>C 1.09786 -1.54157 -0.44905<br>N 2.36088 -1.37692 -0.61997<br>C 4.40729 -0.21233 -0.24746<br>C 5.28995 0.48784 0.56242<br>C 4.94261 0.77258 1.88536<br>C 3.70622 0.37583 2.38214<br>C 2.80879 -0.31450 1.56373	-1982.65422	-1982.23431	-1982.31843	-1983.77875	-1983.44296	-1984.12973	-1983.79395	1.6

C 3.15769 -0.62840 0.23482<br>C 0.37847 -2.41827 -1.40336<br>C -1.01928 -2.52416 -1.42040<br>C -1.65768 -3.35513 -2.33790<br>C -0.91287 -4.09752 -3.24946<br>C 0.47995 -4.00009 -3.24174<br>C 1.11804 -3.16928 -2.33185<br>H -0.54531 -1.52313 0.91714<br>H 4.65844 -0.46320 -1.27375<br>H 6.25222 0.80322 0.17027<br>H 5.63192 1.31125 2.52900<br>H 3.42416 0.61095 3.40480<br>H -1.63269 -1.95457 -0.72923<br>H -2.74196 -3.41988 -2.33557<br>H -1.41195 -4.74910 -3.96121<br>H 1.06963 -4.57745 -3.94833<br>H 2.19931 -3.08318 -2.31664								
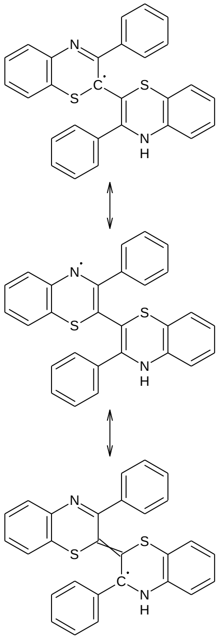<br>S -2.02152 -1.75477 -1.03426<br>C -0.87950 -0.72579 -0.20480<br>C -1.28497 0.13734 0.83058<br>N -2.53795 0.34567 1.23117<br>C -4.86966 -0.06272 1.27967<br>C -6.00371 -0.74849 0.88393<br>C -5.91045 -1.76767 -0.07181<br>C -4.67808 -2.08196 -0.62932	-1982.65389	-1982.23380	-1982.31803	-1983.78060	-1983.44474	-1984.13235	-1983.79650	0.0

C -3.53339 -1.38219 -0.23866<br>C -3.60021 -0.35910 0.73623<br>C -0.27507 0.92668 1.59381<br>C 0.91433 0.35557 2.05983<br>C 1.81005 1.10840 2.81482<br>C 1.53073 2.44164 3.11171<br>C 0.34113 3.01297 2.66199<br>C -0.55909 2.25805 1.91652<br>H -4.91059 0.72059 2.03031<br>H -6.96648 -0.50019 1.32088<br>H -6.79627 -2.31455 -0.38040<br>H -4.59895 -2.86892 -1.37513<br>H 1.13261 -0.68710 1.84999<br>H 2.72435 0.64688 3.17773<br>H 2.23127 3.02822 3.70001<br>H 0.11042 4.04856 2.89697<br>H -1.49570 2.68916 1.57739<br>S 1.21124 -2.51779 -0.60589<br>C 0.47769 -0.87857 -0.72406<br>C 1.22184 0.12230 -1.25950<br>N 2.57414 -0.06679 -1.52617<br>C 4.72714 -0.54371 -0.49313<br>C 5.52238 -1.36633 0.30143<br>C 4.98130 -2.50329 0.89651<br>C 3.64202 -2.82591 0.67841<br>C 2.85750 -2.03669 -0.15937<br>C 3.39274 -0.87617 -0.73223<br>C 0.70313 1.47277 -1.56734<br>C -0.49084 1.63302 -2.28265<br>C -0.95392 2.90478 -2.60313<br>C -0.23091 4.03372 -2.21870<br>C 0.95828 3.88417 -1.50832<br>C 1.42406 2.61285 -1.18632<br>H 5.14228 0.35977 -0.93419<br>H 6.56398 -1.10626 0.46495<br>H 5.59538 -3.14022 1.52559<br>H 3.20634 -3.70996 1.13535<br>H -1.04472 0.75390 -2.59843<br>H -1.87801 3.01455 -3.16349<br>H -0.59369 5.02577 -2.47222<br>H 1.51914 4.75911 -1.19218<br>H 2.33048 2.50438 -0.59561<br>H 3.03228 0.71123 -1.97643								
**Single-bond dimer, C2-peroxyl radical**								

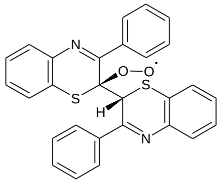<br>S 0.84900 -1.17925 -1.03435<br>C 0.21651 0.41704 -0.50428<br>C 1.29778 1.40208 -0.04408<br>N 2.54635 1.33177 -0.31885<br>C 4.49251 0.52500 -1.41256<br>C 5.18049 -0.42145 -2.15663<br>C 4.52604 -1.58539 -2.56756<br>C 3.19108 -1.79284 -2.24084<br>C 2.49945 -0.83561 -1.49481<br>C 3.14638 0.33320 -1.06878<br>C 0.87412 2.59208 0.74932<br>C 1.64600 2.96346 1.85732<br>C 1.31998 4.09095 2.60230<br>C 0.22399 4.87510 2.24437<br>C -0.53876 4.52545 1.13280<br>C -0.22072 3.39026 0.39024<br>H 4.96769 1.44148 -1.07611<br>H 6.22153 -0.25807 -2.41753<br>H 5.05513 -2.33351 -3.15063<br>H 2.68024 -2.69456 -2.56787<br>H 2.50194 2.35178 2.12411<br>H 1.92309 4.35845 3.46532<br>H -0.02971 5.75756 2.82475<br>H -1.37953 5.14414 0.83181<br>H -0.80273 3.15226 -0.49542<br>S -0.27547 -0.17781 2.20850<br>C -0.90441 0.21140 0.55181<br>C -1.99632 -0.77481 0.14204<br>N -1.98399 -2.02735 0.42592<br>C -0.94610 -4.03047 1.19364<br>C -0.06262 -4.71705 2.01513<br>C 0.75063 -4.00962 2.90226<br>C 0.69369 -2.62001 2.94526<br>C -0.18019 -1.92863 2.10602<br>C -1.02167 -2.63140 1.22402<br>C -3.14791 -0.27167 -0.64727<br>C -3.57959 1.06021 -0.59841<br>C -4.69056 1.47493 -1.32770<br>C -5.37893 0.57094 -2.13177<br>C -4.95487 -0.75696 -2.19322<br>C -3.85651 -1.17503 -1.45451<br>H -1.35682 1.19384 0.68854<br>H -1.60699 -4.55497 0.51007	-2132.82908	-2132.39965	-2132.48817	-2134.05455	-2133.71365	-2134.45224	-2134.11133	0.4

H -0.01519 -5.80110 1.97548<br>H 1.43659 -4.53821 3.55768<br>H 1.33903 -2.06629 3.62187<br>H -3.07272 1.78892 0.02673<br>H -5.01671 2.50913 -1.26611<br>H -6.23935 0.89819 -2.70845<br>H -5.48220 -1.46835 -2.82244<br>H -3.52203 -2.20634 -1.48908<br>O -0.51416 1.07875 -1.62985<br>O 0.24025 1.24886 -2.67681								
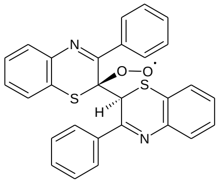<br>S 1.16871 -1.01713 1.62206<br>C -0.19524 -0.46133 0.53357<br>C -0.94306 -1.71841 0.05564<br>N -0.29666 -2.68365 -0.49633<br>C 1.69711 -3.61312 -1.44249<br>C 3.06463 -3.83976 -1.40569<br>C 3.84635 -3.23303 -0.41778<br>C 3.26322 -2.36999 0.50237<br>C 1.89048 -2.10970 0.44762<br>C 1.08623 -2.75527 -0.51411<br>C -2.40004 -1.90643 0.25713<br>C -2.85544 -3.22388 0.42896<br>C -4.20735 -3.49379 0.59467<br>C -5.13425 -2.45158 0.58664<br>C -4.69484 -1.14184 0.41243<br>C -3.33971 -0.86605 0.25434<br>H 1.06076 -4.10413 -2.17262<br>H 3.52302 -4.50440 -2.13186<br>H 4.91469 -3.42298 -0.37235<br>H 3.87267 -1.87107 1.25060<br>H -2.12669 -4.02766 0.42997<br>H -4.53842 -4.51872 0.73559<br>H -6.19236 -2.65981 0.71881<br>H -5.40850 -0.32299 0.40086<br>H -3.02889 0.16080 0.11266<br>S -0.91167 0.75394 -1.93163<br>C 0.32506 0.40212 -0.65144<br>C 0.98337 1.70450 -0.21404<br>N 0.31860 2.77789 0.02394<br>C -1.71789 3.97355 0.37135<br>C -3.04221 4.24421 0.06093<br>C -3.70725 3.46643 -0.89023	-2132.83010	-2132.40039	-2132.48746	-2134.05705	-2133.71441	-2134.45456	-2134.11192	0.0

C -3.05507 2.39821 -1.49657<br>C -1.73165 2.10308 -1.15965<br>C -1.03645 2.90813 -0.23519<br>C 2.45453 1.76378 -0.04337<br>C 2.99647 2.73350 0.81487<br>C 4.36998 2.84910 0.97632<br>C 5.23415 2.00942 0.27125<br>C 4.70881 1.05143 -0.59098<br>C 3.33008 0.92310 -0.74173<br>H 1.05072 -0.23025 -1.16777<br>H -1.16837 4.58285 1.08240<br>H -3.55398 5.07082 0.54441<br>H -4.73713 3.68684 -1.15550<br>H -3.57483 1.77721 -2.22156<br>H 2.31555 3.38539 1.35158<br>H 4.77073 3.59689 1.65481<br>H 6.30953 2.10379 0.39326<br>H 5.37076 0.39858 -1.15276<br>H 2.95492 0.17143 -1.42936<br>O -1.07501 0.35006 1.34372<br>O -0.57755 0.67499 2.50626								
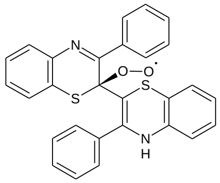<br>S -2.11964 -0.80479 -1.30554<br>C -0.92046 -0.30472 -0.04453<br>C -1.23281 1.07975 0.55529<br>N -2.39361 1.62130 0.59569<br>C -4.75335 1.74917 0.36905<br>C -5.96395 1.31506 -0.15078<br>C -5.99238 0.21990 -1.01707<br>C -4.81467 -0.44120 -1.34822<br>C -3.59932 -0.00680 -0.81647<br>C -3.55285 1.10066 0.04409<br>C -0.13435 1.81084 1.23952<br>C 0.88919 1.15812 1.93806<br>C 1.86374 1.89377 2.60880<br>C 1.83759 3.28523 2.58378<br>C 0.81904 3.94272 1.89274<br>C -0.16095 3.21222 1.23298<br>H -4.69507 2.59940 1.04185<br>H -6.88418 1.82726 0.11303<br>H -6.93477 -0.12591 -1.43182<br>H -4.83678 -1.30057 -2.01313<br>H 0.91196 0.07507 1.98868	-2132.82692	-2132.39712	-2132.48573	-2134.05325	-2133.71206	-2134.45160	-2134.11041	0.9

H 2.64431 1.37342 3.15671<br>H 2.60347 3.85545 3.10207<br>H 0.78835 5.02864 1.86989<br>H -0.96332 3.71252 0.70038<br>C 0.42277 -0.39909 -0.71741<br>S 0.73906 0.95669 -1.83358<br>C 2.45304 1.19697 -1.45761<br>C 3.23423 0.05662 -1.24072<br>N 2.60939 -1.19440 -1.18705<br>C 1.35096 -1.38151 -0.61482<br>C 3.03978 2.45910 -1.45936<br>C 4.41983 2.58644 -1.30828<br>C 5.20308 1.44948 -1.12232<br>C 4.61306 0.18895 -1.06817<br>C 1.20904 -2.68818 0.07575<br>C 0.22510 -3.60207 -0.31524<br>C 0.13820 -4.84628 0.30179<br>C 1.03283 -5.19249 1.31239<br>C 2.02236 -4.29104 1.70161<br>C 2.11608 -3.04920 1.08045<br>H 2.41464 3.33607 -1.59889<br>H 4.87822 3.57005 -1.33692<br>H 6.27884 1.54062 -1.00462<br>H 5.22231 -0.69567 -0.89672<br>H -0.47097 -3.33314 -1.10404<br>H -0.63090 -5.54741 -0.00875<br>H 0.96015 -6.16262 1.79549<br>H 2.71830 -4.55265 2.49355<br>H 2.87936 -2.34071 1.39427<br>O -0.91317 -1.24063 1.10583<br>O -2.10211 -1.42260 1.60316<br>H 3.22797 -1.98703 -1.09923								
**Single-bond dimer, C3-peroxyl radical**								
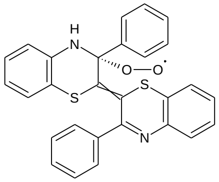<br>S 1.78894 0.21387 1.46006<br>C 0.47250 0.63235 0.35766<br>C 0.46443 2.09048 0.05188<br>N 1.53697 2.71333 -0.30258<br>C 3.81536 2.67296 -1.03781<br>C 5.10981 2.18253 -0.95629<br>C 5.39854 1.11305 -0.10419<br>C 4.38381 0.51695 0.63589<br>C 3.07357 0.98730 0.52872	-2132.83405	-2132.40399	-2132.49250	-2134.05900	-2133.71744	-2134.45809	-2134.11654	0.0

C 2.77473 2.09184 -0.29633<br>C -0.77134 2.88768 0.23919<br>C -1.78423 2.46289 1.10743<br>C -2.90904 3.25408 1.31744<br>C -3.04201 4.47328 0.65520<br>C -2.03600 4.90233 -0.21131<br>C -0.90496 4.12026 -0.41238<br>H 3.57315 3.52718 -1.66304<br>H 5.90093 2.64266 -1.54078<br>H 6.41376 0.73597 -0.02291<br>H 4.59859 -0.33168 1.27947<br>H -1.68727 1.51433 1.62774<br>H -3.68274 2.91645 2.00116<br>H -3.92344 5.08801 0.81530<br>H -2.13338 5.85158 -0.73083<br>H -0.11025 4.44737 -1.07489<br>S -1.51076 0.22077 -1.42950<br>C -0.38312 -0.27639 -0.16641<br>C -0.38737 -1.74376 0.21946<br>N -1.44451 -2.47714 -0.36190<br>C -3.85998 -2.74384 -0.07444<br>C -5.14228 -2.22987 -0.24438<br>C -5.32774 -0.93701 -0.73265<br>C -4.22239 -0.15448 -1.05826<br>C -2.93988 -0.67971 -0.91822<br>C -2.74897 -1.97417 -0.42213<br>C 0.91963 -2.47773 -0.06051<br>C 1.55282 -3.28483 0.88495<br>C 2.70305 -3.99222 0.53775<br>C 3.22845 -3.89670 -0.74779<br>C 2.59388 -3.09343 -1.69431<br>C 1.44165 -2.39273 -1.35446<br>H -3.71378 -3.74263 0.32858<br>H -6.00077 -2.83919 0.02219<br>H -6.32860 -0.53337 -0.85020<br>H -4.34823 0.85969 -1.42613<br>H 1.16606 -3.35550 1.89540<br>H 3.19075 -4.61537 1.28205<br>H 4.12800 -4.44527 -1.01237<br>H 2.99459 -3.01307 -2.70063<br>H 0.93915 -1.77730 -2.09557<br>H -1.38815 -3.45709 -0.11724<br>O -0.57200 -1.66211 1.74870<br>O -1.11849 -2.73128 2.23662								
**Single-bond dimer, dioxolane radical**								

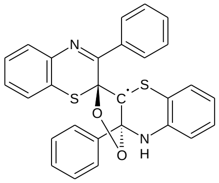<br>S 1.73114 -1.86805 0.37896<br>C 1.02178 -0.25027 -0.15171<br>C 1.92377 0.86398 0.39511<br>N 3.20484 0.81108 0.31545<br>C 5.23817 -0.09985 -0.53506<br>C 6.03237 -1.17276 -0.90784<br>C 5.51348 -2.47065 -0.86510<br>C 4.19658 -2.68460 -0.47881<br>C 3.38373 -1.60198 -0.12821<br>C 3.90580 -0.29351 -0.13833<br>C 1.32803 2.06325 1.02752<br>C 1.97281 2.62757 2.13678<br>C 1.46477 3.77191 2.74083<br>C 0.31423 4.37775 2.23572<br>C -0.32218 3.83313 1.12254<br>C 0.17640 2.67959 0.52379<br>H 5.62140 0.91611 -0.53279<br>H 7.05903 -1.00562 -1.21946<br>H 6.13427 -3.31703 -1.14431<br>H 3.78434 -3.68972 -0.46484<br>H 2.87330 2.15231 2.51329<br>H 1.96718 4.19217 3.60755<br>H -0.08202 5.27299 2.70693<br>H -1.20905 4.30698 0.71168<br>H -0.31850 2.28128 -0.35503<br>S -1.08425 -0.56455 1.72551<br>C -0.43368 -0.29032 0.17501<br>C -1.16868 -0.53668 -1.11699<br>N -2.01004 -1.70400 -1.05041<br>C -3.92580 -2.82108 -0.11637<br>C -4.72695 -3.18190 0.95635<br>C -4.42833 -2.72851 2.24216<br>C -3.31584 -1.92051 2.43059<br>C -2.49210 -1.56972 1.35670<br>C -2.79274 -2.00972 0.05599<br>C -1.91451 0.68876 -1.64573<br>C -1.54019 1.29815 -2.84477<br>C -2.24545 2.40394 -3.31889<br>C -3.32460 2.91123 -2.60001<br>C -3.69933 2.30633 -1.39995<br>C -2.99900 1.20261 -0.92390<br>H -4.16734 -3.17152 -1.11732<br>H -5.59338 -3.81366 0.78520	-2132.83935	-2132.40913	-2132.49602	-2134.06269	-2133.71937	-2134.46111	-2134.11778	0.4

H -5.05344 -2.99965 3.08661<br>H -3.06823 -1.55608 3.42456<br>H -0.69379 0.90648 -3.39848<br>H -1.94479 2.87061 -4.25275<br>H -3.87127 3.77390 -2.97024<br>H -4.53979 2.69505 -0.83171<br>H -3.29828 0.74361 0.01436<br>O 1.00825 -0.08851 -1.56350<br>O -0.09490 -0.89596 -2.00208<br>H -2.47562 -1.88741 -1.92996								
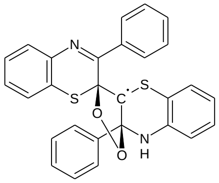<br>S 1.40605 -2.05973 1.44582<br>C 0.93100 -0.33422 0.91842<br>C 2.17247 0.30805 0.29855<br>N 2.88500 -0.31112 -0.57233<br>C 3.21913 -2.06751 -2.15287<br>C 3.14778 -3.39931 -2.53426<br>C 2.55542 -4.33609 -1.68351<br>C 2.01132 -3.93062 -0.47019<br>C 2.05709 -2.58544 -0.09418<br>C 2.68257 -1.63752 -0.92983<br>C 2.56276 1.69103 0.65298<br>C 3.92689 2.01576 0.62333<br>C 4.35128 3.31010 0.89497<br>C 3.41761 4.30482 1.18630<br>C 2.06017 3.99322 1.20582<br>C 1.62978 2.69428 0.95010<br>H 3.69972 -1.32336 -2.78082<br>H 3.56486 -3.71355 -3.48633<br>H 2.50728 -5.38256 -1.97013<br>H 1.53325 -4.65282 0.18580<br>H 4.64149 1.23475 0.38420<br>H 5.41200 3.54420 0.88047<br>H 3.74782 5.31825 1.39718<br>H 1.32765 4.76578 1.42210<br>H 0.56946 2.46721 0.96630<br>S -0.39882 -0.00779 -1.60358<br>C -0.30406 -0.27925 0.07946<br>C -1.43801 0.08450 1.00220<br>N -1.79109 1.46886 0.75277<br>C -3.03985 2.95352 -0.69574<br>C -3.41905 3.38138 -1.96022<br>C -2.93175 2.73469 -3.09681	-2132.83913	-2132.40897	-2132.49469	-2134.06320	-2133.71875	-2134.46116	-2134.11672	1.1

C -2.04630 1.67597 -2.94773<br>C -1.63876 1.25545 -1.67860<br>C -2.15459 1.87820 -0.52676<br>C -2.61153 -0.88958 1.02187<br>C -2.37037 -2.23503 1.31982<br>C -3.42021 -3.14497 1.35369<br>C -4.72305 -2.72441 1.08454<br>C -4.96678 -1.38861 0.78243<br>C -3.91511 -0.47277 0.75199<br>H -3.43427 3.44983 0.18863<br>H -4.10670 4.21620 -2.05708<br>H -3.23366 3.05471 -4.08877<br>H -1.64682 1.17152 -3.82392<br>H -1.35506 -2.56062 1.52746<br>H -3.22151 -4.18616 1.59137<br>H -5.54274 -3.43691 1.10848<br>H -5.97673 -1.05230 0.56628<br>H -4.12280 0.56229 0.50143<br>O 0.55323 0.33447 2.10189<br>O -0.83737 -0.02327 2.28719<br>H -2.33826 1.87625 1.50111								
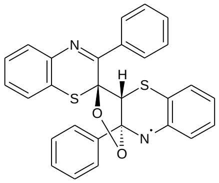<br>S 1.47601 -1.54015 -0.00349<br>C 0.81952 0.13640 -0.28104<br>C 1.81314 1.18718 0.22911<br>N 3.08593 1.07535 0.11551<br>C 5.05913 0.12177 -0.81374<br>C 5.78385 -0.95016 -1.31108<br>C 5.18332 -2.20895 -1.40746<br>C 3.85861 -2.38398 -1.02733<br>C 3.12039 -1.29953 -0.54460<br>C 3.72035 -0.03358 -0.42383<br>C 1.30946 2.44394 0.84434<br>C 1.82755 2.85237 2.07821<br>C 1.40735 4.04535 2.65857<br>C 0.47646 4.85196 2.00601<br>C -0.02925 4.46197 0.76718<br>C 0.38053 3.26356 0.18907<br>H 5.50110 1.10811 -0.70977<br>H 6.81692 -0.81309 -1.61579<br>H 5.74677 -3.05536 -1.78926<br>H 3.38402 -3.35709 -1.11783<br>H 2.56341 2.22481 2.57182	-2132.83813	-2132.40891	-2132.49655	-2134.06012	-2133.71854	-2134.45709	-2134.11551	1.8

H 1.81034 4.34702 3.62125<br>H 0.15057 5.78427 2.45835<br>H -0.74145 5.09375 0.24421<br>H 0.00419 2.98009 -0.79059<br>S -1.02189 -0.48252 1.85889<br>C -0.62780 0.20713 0.24504<br>C -1.46962 -0.33074 -0.94770<br>N -1.93271 -1.68140 -0.95541<br>C -2.40810 -3.80134 -0.08357<br>C -2.49205 -4.70449 0.94906<br>C -2.11056 -4.31801 2.24659<br>C -1.64214 -3.02946 2.48779<br>C -1.55039 -2.09754 1.45551<br>C -1.94487 -2.46062 0.12133<br>C -2.64590 0.59564 -1.23208<br>C -2.60111 1.49068 -2.30067<br>C -3.66572 2.36153 -2.52707<br>C -4.77921 2.34321 -1.69046<br>C -4.82787 1.44390 -0.62636<br>C -3.76748 0.57103 -0.39890<br>H -0.86462 1.26499 0.36104<br>H -2.69062 -4.05985 -1.09930<br>H -2.84761 -5.71359 0.76458<br>H -2.16881 -5.02697 3.06702<br>H -1.33347 -2.74509 3.49041<br>H -1.73648 1.49142 -2.95636<br>H -3.62519 3.05180 -3.36520<br>H -5.60900 3.02094 -1.87008<br>H -5.69589 1.41725 0.02639<br>H -3.81746 -0.13388 0.42668<br>O 0.60380 0.46707 -1.63946<br>O -0.52514 -0.32081 -2.04755								
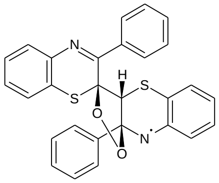<br>S 1.47220 -1.26090 0.79181<br>C 0.87279 0.10949 -0.24436<br>C 1.76628 1.34817 -0.10765<br>N 3.02634 1.32817 0.13600<br>C 5.16262 0.30100 0.32478<br>C 5.98028 -0.79182 0.56783<br>C 5.40990 -2.03266 0.86695<br>C 4.02868 -2.17655 0.90696<br>C 3.20513 -1.07541 0.65447<br>C 3.76518 0.17852 0.36533	-2132.82898	-2132.39961	-2132.48425	-2134.05235	-2133.70762	-2134.44952	-2134.10478	8.6

C 1.17684 2.69347 -0.35380<br>C 0.36828 2.96435 -1.46670<br>C -0.10991 4.25378 -1.68703<br>C 0.20244 5.28222 -0.80037<br>C 1.01040 5.01953 0.30499<br>C 1.50087 3.73625 0.52228<br>H 5.57278 1.28015 0.09666<br>H 7.05982 -0.68224 0.52954<br>H 6.04359 -2.89290 1.06204<br>H 3.58141 -3.14260 1.12519<br>H 0.13500 2.17611 -2.17687<br>H -0.72425 4.45345 -2.56042<br>H -0.17766 6.28521 -0.97277<br>H 1.26255 5.81691 0.99843<br>H 2.14480 3.52583 1.37069<br>S -1.27527 0.13346 1.69026<br>C -0.61027 0.30642 0.03102<br>C -1.28997 -0.60683 -0.99762<br>N -2.62838 -0.18434 -1.25310<br>C -4.73381 0.51597 -0.50498<br>C -5.63480 0.86855 0.47267<br>C -5.20963 0.96540 1.80805<br>C -3.87773 0.73259 2.13949<br>C -2.94185 0.37921 1.16822<br>C -3.36501 0.22407 -0.20769<br>C -1.26669 -2.12662 -0.80621<br>C -0.49247 -2.91444 -1.66323<br>C -0.47950 -4.29981 -1.53150<br>C -1.24242 -4.92034 -0.54552<br>C -2.03275 -4.14366 0.29788<br>C -2.05319 -2.75807 0.16262<br>H -0.86597 1.31993 -0.29004<br>H -5.02716 0.41703 -1.54549<br>H -6.66982 1.06948 0.21403<br>H -5.91283 1.24271 2.58756<br>H -3.55093 0.84710 3.16986<br>H 0.10651 -2.43732 -2.42953<br>H 0.13113 -4.89449 -2.20511<br>H -1.23145 -6.00186 -0.44262<br>H -2.64692 -4.61510 1.05987<br>H -2.69903 -2.18058 0.81378<br>O 0.86422 -0.23216 -1.65434<br>O -0.50502 -0.23673 -2.14061									

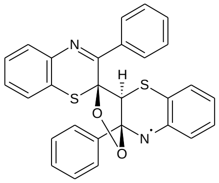<br>S -0.63396 -2.39255 -1.65492<br>C 0.07096 -0.82093 -0.99372<br>C 1.48718 -1.12710 -0.51021<br>N 1.70015 -2.10885 0.28989<br>C 0.92532 -3.79916 1.78965<br>C 0.06500 -4.83538 2.11963<br>C -1.00062 -5.15250 1.27315<br>C -1.21976 -4.40853 0.11936<br>C -0.37657 -3.34009 -0.19866<br>C 0.72722 -3.03734 0.62778<br>C 2.64653 -0.26768 -0.85582<br>C 3.63250 -0.08272 0.12572<br>C 4.76896 0.66932 -0.14226<br>C 4.95016 1.23792 -1.40268<br>C 3.98551 1.04679 -2.38864<br>C 2.83730 0.30671 -2.12033<br>H 1.78208 -3.55689 2.41108<br>H 0.23095 -5.40974 3.02610<br>H -1.66874 -5.97308 1.51743<br>H -2.05912 -4.63915 -0.53093<br>H 3.48692 -0.53979 1.09893<br>H 5.51589 0.81161 0.63381<br>H 5.83991 1.82404 -1.61543<br>H 4.12412 1.47469 -3.37735<br>H 2.10336 0.16453 -2.90396<br>S -0.31652 0.11893 1.75336<br>C -0.89136 -0.18401 0.07250<br>C -1.39613 1.08711 -0.66329<br>N -0.64374 2.29377 -0.52585<br>C 0.94564 3.75144 0.39010<br>C 1.82326 4.12734 1.37798<br>C 2.04292 3.27767 2.47830<br>C 1.39177 2.05042 2.56169<br>C 0.50274 1.64570 1.56779<br>C 0.23107 2.50873 0.44859<br>C -2.88128 1.33510 -0.44911<br>C -3.81273 0.42316 -0.95472<br>C -5.17288 0.62645 -0.74405<br>C -5.61244 1.73451 -0.02041<br>C -4.68502 2.64162 0.48461<br>C -3.32229 2.44701 0.26611<br>H -1.73456 -0.86322 0.20872<br>H 0.74569 4.37996 -0.47211	-2132.84013	-2132.41093	-2132.49703	-2134.06459	-2133.72149	-2134.46153	-2134.11844	0.0

H 2.34753 5.07570 1.31103<br>H 2.73446 3.56972 3.26302<br>H 1.58349 1.39134 3.40449<br>H -3.46990 -0.43692 -1.52374<br>H -5.89120 -0.08137 -1.14789<br>H -6.67463 1.89059 0.14555<br>H -5.02004 3.50897 1.04624<br>H -2.60097 3.16518 0.64233<br>O 0.06523 0.10404 -2.04941<br>O -1.25775 0.66571 -2.02745								
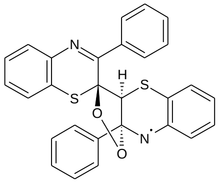<br>S 1.83462 -1.44169 2.10873<br>C 0.40629 -0.89021 1.08716<br>C 0.20888 -1.95331 0.00307<br>N 1.20187 -2.28094 -0.74777<br>C 3.43496 -1.98967 -1.55391<br>C 4.78500 -1.76030 -1.33889<br>C 5.24589 -1.46334 -0.05311<br>C 4.34913 -1.36542 1.00429<br>C 2.98270 -1.55808 0.78510<br>C 2.51057 -1.89653 -0.50163<br>C -1.09465 -2.60193 -0.28205<br>C -1.34053 -3.01117 -1.60287<br>C -2.51917 -3.66398 -1.93697<br>C -3.47002 -3.93857 -0.95351<br>C -3.22707 -3.55459 0.36220<br>C -2.05384 -2.88489 0.69978<br>H 3.05766 -2.25973 -2.53564<br>H 5.48478 -1.83014 -2.16626<br>H 6.30446 -1.29805 0.12433<br>H 4.70098 -1.11466 2.00114<br>H -0.58774 -2.80713 -2.35732<br>H -2.69598 -3.96246 -2.96662<br>H -4.39205 -4.45188 -1.21189<br>H -3.95573 -3.77367 1.13731<br>H -1.88712 -2.59674 1.72981<br>S 0.99032 1.02159 -1.10701<br>C 0.58159 0.56559 0.59128<br>C -0.68615 1.25510 1.09424<br>N -0.45317 2.64195 1.32291<br>C 0.23950 4.72880 0.50515<br>C 0.77420 5.54915 -0.45993<br>C 1.30875 4.98672 -1.63202	-2132.82325	-2132.39389	-2132.47937	-2134.04843	-2133.70454	-2134.44560	-2134.10171	10.5

C 1.33105 3.60738 -1.80163<br>C 0.80316 2.75082 -0.83341<br>C 0.18981 3.30856 0.35020<br>C -2.03426 1.11486 0.39216<br>C -3.12606 1.66238 1.08112<br>C -4.40616 1.59864 0.54541<br>C -4.61750 0.98508 -0.68947<br>C -3.53968 0.43555 -1.37532<br>C -2.25299 0.50256 -0.84122<br>H 1.38071 0.99184 1.20745<br>H -0.20034 5.12618 1.41466<br>H 0.77939 6.62564 -0.31993<br>H 1.72895 5.62589 -2.40273<br>H 1.78870 3.18158 -2.69097<br>H -2.96051 2.13207 2.04557<br>H -5.24095 2.02570 1.09401<br>H -5.61800 0.93065 -1.10951<br>H -3.69303 -0.06001 -2.32966<br>H -1.43118 0.06930 -1.39665<br>O -0.69926 -0.78231 1.97167<br>O -0.76366 0.59792 2.37935								
**Single-bond dimer, dioxane radical**								
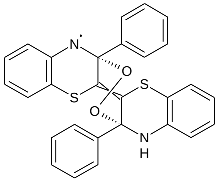<br>S -1.63220 -0.04024 2.03837<br>C -0.71119 -0.00209 0.54479<br>C -1.49728 0.11437 -0.74230<br>N -2.48464 -0.91191 -0.89887<br>C -4.36331 -2.11484 -0.16206<br>C -5.28641 -2.45229 0.79871<br>C -5.13123 -1.98114 2.11592<br>C -4.02925 -1.20740 2.46001<br>C -3.07271 -0.86808 1.50239<br>C -3.24728 -1.26829 0.13297<br>C -2.13053 1.48775 -0.96249<br>C -1.73492 2.61017 -0.23553<br>C -2.30758 3.85368 -0.49670<br>C -3.27698 3.98409 -1.48748<br>C -3.66684 2.86599 -2.22445<br>C -3.09576 1.62446 -1.96572<br>H -4.46180 -2.44155 -1.19265<br>H -6.13766 -3.07531 0.54190<br>H -5.86177 -2.23838 2.87691<br>H -3.89381 -0.88442 3.48899	-2132.83916	-2132.40934	-2132.49572	-2134.06385	-2133.72041	-2134.46310	-2134.11967	0.0

H -0.97015 2.51906 0.52958<br>H -1.99091 4.72136 0.07526<br>H -3.72384 4.95368 -1.68934<br>H -4.41520 2.96205 -3.00622<br>H -3.38737 0.75281 -2.54330<br>S 1.57737 -0.35275 1.98359<br>C 0.63285 -0.04354 0.54564<br>C 1.39329 0.16177 -0.74966<br>N 2.30204 1.26580 -0.55597<br>C 4.51390 1.85881 0.23029<br>C 5.50988 1.80674 1.19539<br>C 5.31987 1.06064 2.35860<br>C 4.11801 0.39073 2.54894<br>C 3.10244 0.45455 1.59167<br>C 3.29915 1.18152 0.40520<br>C 2.04220 -1.12216 -1.26777<br>C 3.24417 -1.06780 -1.97650<br>C 3.80757 -2.22450 -2.50903<br>C 3.17564 -3.45326 -2.33436<br>C 1.97980 -3.51689 -1.62281<br>C 1.41846 -2.35962 -1.09091<br>H 4.66756 2.43063 -0.68241<br>H 6.44084 2.34139 1.03233<br>H 6.09806 1.00251 3.11273<br>H 3.95342 -0.18370 3.45706<br>H 3.75772 -0.12070 -2.11133<br>H 4.74442 -2.16435 -3.05557<br>H 3.61554 -4.35707 -2.74647<br>H 1.47977 -4.47055 -1.47972<br>H 0.48546 -2.41935 -0.53994<br>H 2.56851 1.71446 -1.42240<br>O 0.49290 0.70652 -1.72044<br>O -0.59903 -0.19054 -1.83323								
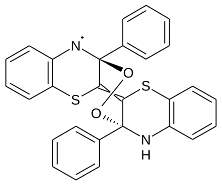<br>S -1.71914 -1.24853 -1.51695<br>C -0.71978 -0.41933 -0.33294<br>C -1.48294 0.44156 0.65047<br>N -2.34032 -0.36123 1.47626<br>C -4.04862 -1.95058 1.75734<br>C -4.95804 -2.85489 1.26027<br>C -4.92481 -3.20417 -0.10186<br>C -3.95176 -2.67131 -0.94139<br>C -3.01014 -1.76784 -0.45158	-2132.83897	-2132.40905	-2132.49509	-2134.06416	-2133.72028	-2134.46338	-2134.11951	0.1

C -3.07238 -1.32689 0.91684<br>C -2.24283 1.58408 -0.03226<br>C -1.57469 2.36961 -0.97517<br>C -2.22882 3.42885 -1.59396<br>C -3.55605 3.71445 -1.27372<br>C -4.21917 2.94133 -0.32514<br>C -3.56412 1.88014 0.29874<br>H -4.05686 -1.64036 2.79782<br>H -5.70381 -3.29475 1.91537<br>H -5.64437 -3.91265 -0.50116<br>H -3.90429 -2.98273 -1.98168<br>H -0.53798 2.15272 -1.21748<br>H -1.70158 4.03289 -2.32697<br>H -4.06838 4.54021 -1.75961<br>H -5.24982 3.16386 -0.06357<br>H -4.07896 1.28751 1.04742<br>S 1.46003 -1.62566 -1.39956<br>C 0.61308 -0.59065 -0.26912<br>C 1.36307 0.09777 0.86198<br>N 2.32073 -0.82187 1.44313<br>C 4.59976 -1.60192 1.17635<br>C 5.59074 -2.20162 0.41186<br>C 5.33152 -2.57031 -0.90850<br>C 4.07024 -2.34306 -1.44461<br>C 3.06136 -1.76413 -0.67086<br>C 3.32016 -1.37628 0.65189<br>C 1.99535 1.43777 0.48872<br>C 2.21552 2.38456 1.49444<br>C 2.86579 3.57960 1.20654<br>C 3.30780 3.84432 -0.08966<br>C 3.08902 2.90676 -1.09435<br>C 2.43483 1.70864 -0.80781<br>H 4.80713 -1.29937 2.20023<br>H 6.57257 -2.36828 0.84467<br>H 6.10521 -3.02612 -1.51797<br>H 3.85476 -2.62487 -2.47216<br>H 1.84735 2.19225 2.49810<br>H 3.01797 4.31183 1.99461<br>H 3.81423 4.77875 -0.31426<br>H 3.42620 3.10390 -2.10816<br>H 2.26655 0.98960 -1.60461<br>H 2.66807 -0.45319 2.32070<br>O 0.44060 0.24998 1.93685<br>O -0.56258 1.15736 1.47590								
**Single-bond dimer, C2-hydroperoxylalkyl radical**								

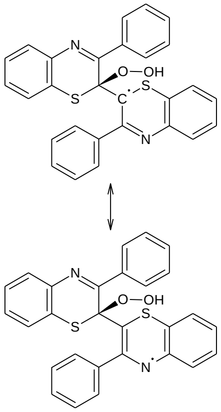<br>S 1.14810 -2.23257 0.28532<br>C 0.42898 -0.86376 -0.72715<br>C 1.32783 0.37573 -0.84669<br>N 2.58447 0.40924 -0.60082<br>C 4.73190 -0.38137 0.00698<br>C 5.59866 -1.34191 0.50536<br>C 5.09589 -2.57735 0.92037<br>C 3.73464 -2.84305 0.82766<br>C 2.86391 -1.87346 0.32172<br>C 3.35180 -0.62302 -0.09172<br>C 0.73389 1.60925 -1.43072<br>C -0.43092 1.61374 -2.21079<br>C -0.92026 2.80393 -2.74363<br>C -0.25887 4.00636 -2.50930<br>C 0.90568 4.01077 -1.74077<br>C 1.39680 2.82605 -1.20995<br>H 5.08920 0.58830 -0.32614<br>H 6.66204 -1.13342 0.57231<br>H 5.76394 -3.33767 1.31435<br>H 3.34218 -3.80525 1.14712<br>H -0.94612 0.68795 -2.43709<br>H -1.81908 2.78490 -3.35346<br>H -0.64447 4.93305 -2.92543<br>H 1.42966 4.94323 -1.54967<br>H 2.29627 2.82011 -0.60415<br>C -0.97164 -0.66062 -0.18026<br>S -2.10540 -1.77599 -0.91203<br>C -3.59861 -1.38023 -0.09820<br>C -3.66529 -0.32839 0.84104<br>N -2.60371 0.41562 1.27323<br>C -1.35908 0.23025 0.83715<br>C -4.73813 -2.11514 -0.43922<br>C -5.96089 -1.80824 0.14144<br>C -6.05236 -0.75964 1.06583	-2132.83944	-2132.41052	-2132.49883	-2134.06829	-2133.72768	-2134.46771	-2134.12710	0.0

C -4.92464 -0.03647 1.40845<br>C -0.38714 1.11705 1.54867<br>C -0.65610 2.49032 1.59614<br>C 0.16816 3.34611 2.31628<br>C 1.26208 2.83941 3.01867<br>C 1.52053 1.47180 2.99793<br>C 0.70099 0.61410 2.26639<br>H -4.66217 -2.92275 -1.16300<br>H -6.84282 -2.38215 -0.12634<br>H -7.00938 -0.51706 1.51816<br>H -4.96301 0.77353 2.13022<br>H -1.51748 2.87660 1.06031<br>H -0.04483 4.41135 2.33254<br>H 1.90392 3.50770 3.58618<br>H 2.35899 1.06476 3.55609<br>H 0.89780 -0.45283 2.27219<br>O 0.20686 -1.35129 -2.06063<br>O 1.44589 -1.70228 -2.64971<br>H 1.54799 -2.62223 -2.35130								
**Single-bond dimer, C3-hydroperoxylalkyl radical**								
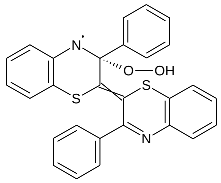<br>S 1.94447 -0.65965 1.47684<br>C 0.88793 0.34600 0.47371<br>C 1.57230 1.56860 -0.03162<br>N 2.75728 1.55179 -0.53943<br>C 4.64507 0.34913 -1.38625<br>C 5.54604 -0.70240 -1.31695<br>C 5.36697 -1.70894 -0.36366<br>C 4.26946 -1.67691 0.48850<br>C 3.33921 -0.63783 0.39700<br>C 3.53302 0.40452 -0.53135<br>C 0.91229 2.88964 0.12418<br>C -0.01937 3.11788 1.14527<br>C -0.57843 4.38078 1.31435<br>C -0.22637 5.42534 0.46166<br>C 0.70116 5.20336 -0.55689<br>C 1.27390 3.94761 -0.71946<br>H 4.78237 1.16644 -2.08807<br>H 6.39894 -0.73167 -1.98842<br>H 6.07813 -2.52656 -0.29049<br>H 4.11368 -2.47288 1.21183<br>H -0.29781 2.30758 1.81387<br>H -1.29060 4.54891 2.11731	-2132.82465	-2132.39596	-2132.48511	-2134.04834	-2133.70881	-2134.44844	-2134.10890	0.0

H -0.66983 6.40871 0.59127<br>H 0.98051 6.01344 -1.22491<br>H 2.00728 3.76487 -1.49827<br>S -1.15845 0.87391 -1.15227<br>C -0.39220 0.01309 0.17316<br>C -1.12910 -1.16021 0.80624<br>N -2.56042 -1.03943 0.92617<br>C -4.73576 -0.48970 0.22360<br>C -5.60853 0.11926 -0.64429<br>C -5.11215 0.91655 -1.69486<br>C -3.74533 1.11093 -1.84422<br>C -2.84279 0.50550 -0.96659<br>C -3.31646 -0.35415 0.07819<br>C -0.82932 -2.50148 0.13778<br>C -1.36224 -3.65805 0.71886<br>C -1.14037 -4.89976 0.13391<br>C -0.39131 -4.99817 -1.03848<br>C 0.13774 -3.84859 -1.61904<br>C -0.07929 -2.60278 -1.03367<br>H -5.08231 -1.12639 1.03180<br>H -6.67917 -0.01633 -0.52594<br>H -5.79871 1.39813 -2.38446<br>H -3.37146 1.75477 -2.63602<br>H -1.94331 -3.57294 1.63075<br>H -1.55164 -5.79328 0.59503<br>H -0.21877 -5.96848 -1.49573<br>H 0.72780 -3.91716 -2.52842<br>H 0.34682 -1.71414 -1.48838<br>O -0.66311 -1.32543 2.15393<br>O -1.14337 -0.23018 2.93327<br>H -2.09075 -0.44833 2.98482								
**Single-bond dimer, C2-hydroperoxide**								
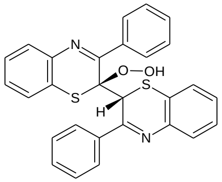<br>S 0.97219 -0.98771 -1.20539<br>C 0.09349 0.48277 -0.56585<br>C 1.02158 1.60405 -0.07163<br>N 2.28660 1.69395 -0.23996<br>C 4.41900 1.13096 -1.11095<br>C 5.29074 0.29659 -1.79414<br>C 4.82339 -0.91546 -2.30806<br>C 3.49348 -1.28137 -2.13839<br>C 2.61721 -0.43865 -1.44812<br>C 3.07287 0.78047 -0.92615	-2133.47129	-2133.02920	-2133.11856	-2134.70194	-2134.34921	-2135.10123	-2134.74850	0.6

<table><tr><td>C 0.38746 2.73367 0.67067
C -0.78315 3.37198 0.23608
C -1.30071 4.45195 0.94817
C -0.66670 4.90509 2.10250
C 0.50171 4.28010 2.53654
C 1.02721 3.20849 1.82346
H 4.74416 2.08254 -0.70131
H 6.32883 0.58463 -1.92831
H 5.49581 -1.57929 -2.84368
H 3.13049 -2.22583 -2.53578
H -1.26740 3.04566 -0.67788
H -2.19857 4.94770 0.58949
H -1.07641 5.74436 2.65737
H 1.00541 4.62799 3.43404
H 1.93968 2.71978 2.14970
C -0.90055 0.04746 0.55674
S -0.08871 -0.29185 2.14231
C 0.28085 -1.99807 1.95370
C -0.50505 -2.79748 1.10344
N -1.60885 -2.33068 0.40490
C -1.84764 -1.08824 0.17898
C 1.31720 -2.56546 2.69512
C 1.59095 -3.92545 2.58535
C 0.83286 -4.72524 1.72837
C -0.21019 -4.16399 1.00434
C -3.13003 -0.75880 -0.49396
C -3.83697 -1.78813 -1.13713
C -5.05142 -1.53725 -1.75984
C -5.59577 -0.25205 -1.74748
C -4.90819 0.77650 -1.11059
C -3.68363 0.52828 -0.49567
H 1.91789 -1.93628 3.34642
H 2.40166 -4.35750 3.16467
H 1.04840 -5.78555 1.63669
H -0.83000 -4.76599 0.34658
H -3.40866 -2.78458 -1.13325
H -5.57745 -2.34670 -2.25825
H -6.54778 -0.05533 -2.23237
H -5.32246 1.78039 -1.09162
H -3.17647 1.35622 -0.01185
O -0.77911 0.98082 -1.57775
O -0.00049 1.52228 -2.63847
H 0.13812 0.73686 -3.19428
H -1.48845 0.93996 0.77497</td><td></td><td></td><td></td><td></td><td></td><td></td><td></td><td></td><td></td></tr></table>

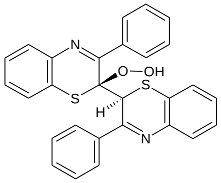<br>S 1.21209 -1.02602 1.60499<br>C -0.18525 -0.43917 0.54727<br>C -0.89688 -1.69819 0.02688<br>N -0.24521 -2.65603 -0.53176<br>C 1.74853 -3.56625 -1.48900<br>C 3.11536 -3.79832 -1.45948<br>C 3.89850 -3.21305 -0.46005<br>C 3.31921 -2.36584 0.47713<br>C 1.94772 -2.09621 0.42785<br>C 1.13981 -2.72351 -0.54551<br>C -2.36242 -1.89002 0.18304<br>C -3.29450 -0.83974 0.18034<br>C -4.65526 -1.10543 0.32475<br>C -5.10825 -2.41398 0.47175<br>C -4.18978 -3.46404 0.47070<br>C -2.83241 -3.20507 0.32928<br>H 1.10944 -4.04118 -2.22743<br>H 3.57095 -4.45008 -2.19888<br>H 4.96635 -3.40719 -0.41832<br>H 3.93078 -1.88459 1.23496<br>H -2.96543 0.18393 0.04609<br>H -5.36126 -0.27981 0.31447<br>H -6.16944 -2.61545 0.58750<br>H -4.53242 -4.48823 0.58777<br>H -2.10965 -4.01436 0.32855<br>C 0.34777 0.43930 -0.62047<br>S -0.87112 0.80314 -1.91743<br>C -1.69698 2.15251 -1.15159<br>C -1.00798 2.95321 -0.21882<br>N 0.34523 2.82079 0.04775<br>C 1.00460 1.74235 -0.17610<br>C -3.01721 2.45078 -1.49780<br>C -3.67386 3.51743 -0.89243<br>C -3.01519 4.29040 0.06665<br>C -1.69296 4.01699 0.38566<br>C 2.47497 1.79825 0.00766<br>C 3.35676 0.96242 -0.68865<br>C 4.73404 1.08907 -0.52440<br>C 5.25224 2.04027 0.34979<br>C 4.38198 2.87511 1.05283<br>C 3.00983 2.76153 0.87738<br>H -3.53086 1.83497 -2.23173<br>H -4.70078 3.74127 -1.16648	-2133.47332	-2133.03098	-2133.11679	-2134.70674	-2134.35020	-2135.10602	-2134.74949	0.0

H -3.52906 5.11676 0.54858<br>H -1.14775 4.62451 1.10163<br>H 2.98732 0.21491 -1.38382<br>H 5.40072 0.43976 -1.08474<br>H 6.32652 2.13273 0.48271<br>H 4.77672 3.61776 1.74053<br>H 2.32433 3.41084 1.41140<br>O -0.97837 0.41462 1.32854<br>O -1.38901 -0.23584 2.52803<br>H -2.25749 -0.59260 2.26937<br>H 1.07988 -0.18614 -1.13562								
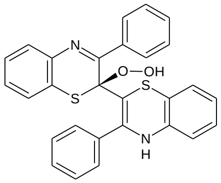<br>S 2.12243 0.85862 -1.31057<br>C 0.91154 0.36437 -0.01405<br>C 1.24310 -1.01923 0.58265<br>N 2.39829 -1.57356 0.60312<br>C 4.75432 -1.70527 0.36099<br>C 5.96626 -1.27450 -0.15866<br>C 5.99795 -0.17738 -1.02246<br>C 4.82088 0.48618 -1.35081<br>C 3.60249 0.05420 -0.82037<br>C 3.55341 -1.05399 0.04059<br>C 0.15277 -1.74199 1.29021<br>C 0.17295 -3.14353 1.29017<br>C -0.80514 -3.86713 1.96066<br>C -1.81616 -3.20223 2.65540<br>C -1.83546 -1.81042 2.67420<br>C -0.86243 -1.08132 1.99406<br>H 4.69387 -2.55670 1.03216<br>H 6.88467 -1.79136 0.10271<br>H 6.94046 0.16498 -1.43992<br>H 4.84303 1.34247 -2.02019<br>H 0.96877 -3.64953 0.75310<br>H -0.77929 -4.95335 1.94244<br>H -2.58091 -3.76679 3.18164<br>H -2.60937 -1.28449 3.22656<br>H -0.87206 0.00179 2.04063<br>C -0.41891 0.39921 -0.72893<br>S -0.66164 -0.97854 -1.83602<br>C -2.36793 -1.28993 -1.47745<br>C -3.20169 -0.18242 -1.28715<br>N -2.63168 1.09472 -1.24007<br>C -1.39163 1.33771 -0.64596	-2133.46708	-2133.02465	-2133.11299	-2134.69880	-2134.34471	-2135.09900	-2134.74491	2.9

C -2.89873 -2.57653 -1.46899<br>C -4.27402 -2.76276 -1.33629<br>C -5.10921 -1.65901 -1.17867<br>C -4.57594 -0.37309 -1.13361<br>C -1.32078 2.65396 0.03930<br>C -0.41273 3.62994 -0.38368<br>C -0.39726 4.88314 0.22117<br>C -1.28711 5.17504 1.25381<br>C -2.20142 4.21087 1.67419<br>C -2.22545 2.96028 1.06275<br>H -2.23339 -3.42669 -1.58675<br>H -4.68808 -3.76605 -1.35696<br>H -6.18164 -1.79584 -1.07547<br>H -5.22607 0.48595 -0.98329<br>H 0.27717 3.40020 -1.19042<br>H 0.30876 5.63604 -0.11783<br>H -1.27082 6.15265 1.72707<br>H -2.89418 4.43080 2.48148<br>H -2.93140 2.20374 1.39777<br>O 0.85387 1.31290 1.04124<br>O 2.10699 1.32953 1.71539<br>H 2.56869 2.03914 1.23984<br>H -3.28595 1.85923 -1.16172								
**Single-bond dimer, C3-hydroperoxide**								
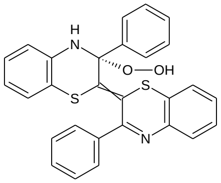<br>S 1.05816 -1.63983 1.40102<br>C 0.92768 -0.12860 0.49296<br>C 2.21933 0.24940 -0.14333<br>N 2.91348 -0.59594 -0.82642<br>C 3.14976 -2.72325 -1.89593<br>C 2.98326 -4.09932 -1.91335<br>C 2.26744 -4.72746 -0.88977<br>C 1.69137 -3.97188 0.12428<br>C 1.82449 -2.58101 0.12336<br>C 2.58220 -1.93911 -0.87879<br>C 2.77847 1.60390 0.05945<br>C 2.46155 2.34644 1.20509<br>C 3.04735 3.59200 1.41651<br>C 3.94006 4.11463 0.48444<br>C 4.25672 3.38002 -0.66048<br>C 3.68842 2.13047 -0.86802<br>H 3.73655 -2.21511 -2.65542<br>H 3.42602 -4.68891 -2.71062	-2133.47116	-2133.02809	-2133.11491	-2134.70234	-2134.34609	-2135.10308	-2134.74683	0.0

H 2.14909 -5.80707 -0.88717<br>H 1.11211 -4.45467 0.90666<br>H 1.76666 1.94368 1.93793<br>H 2.80327 4.15387 2.31352<br>H 4.38963 5.09021 0.64755<br>H 4.95197 3.78436 -1.39096<br>H 3.93526 1.54187 -1.74572<br>S -0.24741 2.14345 -0.41640<br>C -0.22437 0.57208 0.41134<br>C -1.48446 0.10738 1.13798<br>N -2.56357 1.11131 1.06241<br>C -4.25040 1.73643 -0.59803<br>C -4.56583 2.34835 -1.80737<br>C -3.55578 2.85029 -2.62704<br>C -2.22561 2.74073 -2.23005<br>C -1.90969 2.15659 -1.00410<br>C -2.92048 1.65170 -0.17992<br>C -2.00338 -1.24639 0.66728<br>C -2.66512 -2.10198 1.55393<br>C -3.23987 -3.28183 1.08799<br>C -3.17056 -3.61304 -0.26396<br>C -2.52149 -2.75608 -1.14975<br>C -1.94184 -1.57741 -0.68820<br>H -5.03708 1.32830 0.03246<br>H -5.60460 2.41854 -2.11603<br>H -3.79984 3.31513 -3.57717<br>H -1.42630 3.11444 -2.86379<br>H -2.70900 -1.85660 2.61033<br>H -3.73878 -3.94688 1.78748<br>H -3.61844 -4.53462 -0.62466<br>H -2.45998 -3.00520 -2.20519<br>H -1.42861 -0.91985 -1.38386<br>H -3.37322 0.71359 1.52963<br>O -1.22676 -0.02952 2.53044<br>O -0.75762 1.22130 3.02968<br>H -1.45621 1.82677 2.70908								
**Single-bond dimer, dioxolane**								
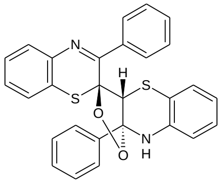<br>S 2.10889 -0.43342 0.30995<br>C 0.53853 0.40744 -0.08206<br>C 0.67197 1.89970 0.25417<br>N 1.71856 2.58889 -0.02331<br>C 3.78653 2.92907 -1.16007	-2133.49066	-2133.04735	-2133.13214	-2134.71939	-2134.36087	-2135.11702	-2134.75850	1.1

C 4.99899 2.47112 -1.65139<br>C 5.32972 1.11751 -1.53306<br>C 4.43778 0.22716 -0.94892<br>C 3.20262 0.68172 -0.47635<br>C 2.87255 2.04568 -0.56601<br>C -0.45020 2.63679 0.89422<br>C -1.73592 2.65678 0.33882<br>C -2.74729 3.40461 0.93657<br>C -2.48942 4.13227 2.09685<br>C -1.20991 4.12228 2.64997<br>C -0.19414 3.38591 2.04796<br>H 3.51046 3.97786 -1.21439<br>H 5.69404 3.16442 -2.11536<br>H 6.28234 0.75339 -1.90682<br>H 4.68426 -0.82831 -0.87291<br>H -1.94179 2.11128 -0.57837<br>H -3.73695 3.41984 0.48903<br>H -3.28161 4.70933 2.56548<br>H -1.00071 4.69226 3.55082<br>H 0.80927 3.38398 2.46284<br>S -0.37622 -1.33680 2.05133<br>C -0.65047 -0.37068 0.54748<br>C -1.21872 -1.18401 -0.66176<br>N -1.31501 -2.59039 -0.43390<br>C 0.16356 -4.53332 -0.33745<br>C 1.12412 -5.27258 0.34719<br>C 1.67521 -4.78685 1.53164<br>C 1.25722 -3.55599 2.03151<br>C 0.27420 -2.82893 1.36452<br>C -0.27872 -3.31126 0.17125<br>C -2.58190 -0.67018 -1.10787<br>C -3.68645 -0.85642 -0.27020<br>C -4.94103 -0.39018 -0.65109<br>C -5.10358 0.26288 -1.87321<br>C -4.00592 0.44405 -2.71046<br>C -2.74640 -0.02056 -2.33079<br>H -1.40080 0.35711 0.85781<br>H -0.25236 -4.90469 -1.27120<br>H 1.45280 -6.22550 -0.05709<br>H 2.43481 -5.35583 2.05867<br>H 1.68777 -3.15389 2.94431<br>H -3.55788 -1.37548 0.67617<br>H -5.79375 -0.54042 0.00507<br>H -6.08349 0.62481 -2.17169<br>H -4.12599 0.94790 -3.66546<br>H -1.88814 0.12071 -2.97900<br>O 0.22288 0.37470 -1.46757<br>O -0.22880 -0.96749 -1.68910<br>H -1.75002 -3.06640 -1.21343								

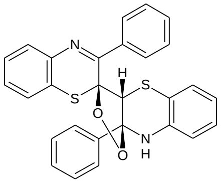<br>S 1.54839 -1.18692 0.81393<br>C 0.87187 0.12486 -0.25032<br>C 1.68327 1.42059 -0.12332<br>N 2.94205 1.48035 0.12040<br>C 5.13736 0.58730 0.31818<br>C 6.01984 -0.45009 0.57678<br>C 5.52589 -1.71802 0.89786<br>C 4.15598 -1.94437 0.94305<br>C 3.26708 -0.89951 0.67362<br>C 3.74988 0.38129 0.36375<br>C 1.01272 2.72444 -0.38228<br>C 0.18930 2.93491 -1.49714<br>C -0.36461 4.19118 -1.73084<br>C -0.11287 5.24581 -0.85581<br>C 0.70961 5.04341 0.25142<br>C 1.27513 3.79393 0.48207<br>H 5.48724 1.58587 0.07426<br>H 7.09073 -0.27605 0.53456<br>H 6.21070 -2.53488 1.10623<br>H 3.76786 -2.93178 1.17826<br>H 0.00243 2.12620 -2.19795<br>H -0.99107 4.34481 -2.60493<br>H -0.55240 6.22242 -1.03814<br>H 0.91363 5.86161 0.93634<br>H 1.92949 3.63095 1.33287<br>S -1.27312 0.06160 1.67976<br>C -0.62432 0.23471 0.01853<br>C -1.22777 -0.74108 -1.00365<br>N -2.58377 -0.33756 -1.21619<br>C -4.75982 0.30214 -0.44304<br>C -5.65300 0.71628 0.53311<br>C -5.20682 0.95480 1.83282<br>C -3.86290 0.77325 2.12874<br>C -2.95372 0.33774 1.15853<br>C -3.40116 0.09300 -0.15821<br>C -1.09432 -2.24539 -0.79587<br>C -0.25193 -3.00703 -1.61103<br>C -0.17490 -4.38797 -1.45135<br>C -0.93394 -5.02652 -0.47365<br>C -1.78558 -4.27567 0.33356<br>C -1.87282 -2.89573 0.16884<br>H -0.94138 1.21992 -0.33087<br>H -5.10586 0.13487 -1.46051	-2133.47442	-2133.03113	-2133.11557	-2134.70294	-2134.34409	-2135.10100	-2134.74214	11.3

H -6.69719 0.86331 0.27381<br>H -5.89319 1.29005 2.60372<br>H -3.49502 0.97020 3.13242<br>H 0.34905 -2.51342 -2.36593<br>H 0.48496 -4.96472 -2.09342<br>H -0.86987 -6.10369 -0.34708<br>H -2.39199 -4.76347 1.09147<br>H -2.55701 -2.33045 0.79263<br>O 0.90275 -0.24160 -1.65250<br>O -0.46341 -0.32266 -2.14631<br>H -3.07267 -0.92157 -1.88152								
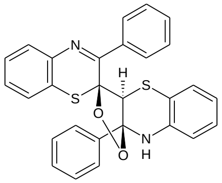<br>S -1.27128 -1.62931 -1.67307<br>C -0.04844 -0.56617 -0.77734<br>C 1.10248 -1.48767 -0.36589<br>N 0.84863 -2.56604 0.28495<br>C -0.62786 -3.96028 1.55042<br>C -1.85141 -4.58860 1.72783<br>C -2.89961 -4.34167 0.83683<br>C -2.72915 -3.43925 -0.20663<br>C -1.50895 -2.77777 -0.36927<br>C -0.43016 -3.05053 0.49970<br>C 2.52934 -1.17427 -0.62277<br>C 3.47598 -1.69808 0.27319<br>C 4.83362 -1.48828 0.07622<br>C 5.27692 -0.75955 -1.02802<br>C 4.34830 -0.24720 -1.92911<br>C 2.98421 -0.44576 -1.73015<br>H 0.21195 -4.16444 2.20762<br>H -1.98764 -5.28471 2.55004<br>H -3.85481 -4.84272 0.96277<br>H -3.54838 -3.22504 -0.88731<br>H 3.11985 -2.27072 1.12298<br>H 5.54953 -1.89467 0.78547<br>H 6.33946 -0.59582 -1.18534<br>H 4.68297 0.31220 -2.79799<br>H 2.28247 -0.04606 -2.45111<br>S 0.24130 0.43105 1.90729<br>C -0.72427 0.21776 0.38711<br>C -1.11601 1.56136 -0.30774<br>N -0.38537 2.69336 0.19022<br>C 1.87049 3.62190 0.09345<br>C 3.19391 3.59052 0.52334	-2133.49090	-2133.04761	-2133.13227	-2134.72143	-2134.36280	-2135.11882	-2134.76019	0.0

C 3.64488 2.56040 1.34665<br>C 2.76217 1.55828 1.74221<br>C 1.43012 1.60339 1.33941<br>C 0.97322 2.63557 0.50934<br>C -2.60680 1.85945 -0.24714<br>C -3.48507 1.36052 -1.21349<br>C -4.84973 1.62151 -1.11714<br>C -5.35148 2.36931 -0.05391<br>C -4.47922 2.85891 0.91545<br>C -3.11209 2.60897 0.81916<br>H -1.62414 -0.30580 0.71430<br>H 1.52451 4.41767 -0.56234<br>H 3.87907 4.36836 0.19882<br>H 4.68128 2.52605 1.66749<br>H 3.10244 0.73738 2.36639<br>H -3.09757 0.76956 -2.03563<br>H -5.52264 1.23750 -1.87867<br>H -6.41687 2.56885 0.01901<br>H -4.86073 3.43876 1.75112<br>H -2.42794 2.98791 1.57182<br>O 0.33921 0.44577 -1.66507<br>O -0.81334 1.30482 -1.69656<br>H -0.63536 3.52532 -0.33026								
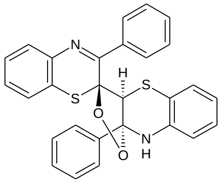<br>S -0.64810 -2.34049 -1.98385<br>C 0.18615 -0.97451 -1.08316<br>C 0.99182 -1.60833 0.05336<br>N 0.44335 -2.42608 0.87685<br>C -1.45678 -3.56018 1.78266<br>C -2.65433 -4.24257 1.63193<br>C -3.22619 -4.37336 0.36376<br>C -2.61407 -3.79323 -0.74135<br>C -1.42711 -3.07311 -0.58738<br>C -0.82125 -2.96704 0.68211<br>C 2.41996 -1.27261 0.27321<br>C 2.84456 -0.97495 1.57304<br>C 4.18757 -0.72881 1.83583<br>C 5.12647 -0.80214 0.80843<br>C 4.71255 -1.11618 -0.48478<br>C 3.36636 -1.33989 -0.75679<br>H -0.97171 -3.47573 2.75041<br>H -3.13596 -4.68969 2.49634<br>H -4.15614 -4.91952 0.23610	-2133.46847	-2133.02519	-2133.10885	-2134.70205	-2134.34243	-2135.10015	-2134.74053	12.3

H -3.06784 -3.87439 -1.72521<br>H 2.10772 -0.93531 2.36900<br>H 4.50191 -0.48347 2.84634<br>H 6.17743 -0.61987 1.01546<br>H 5.44077 -1.19076 -1.28763<br>H 3.05649 -1.58874 -1.76625<br>S -1.48593 0.27070 0.98338<br>C -0.79216 0.14047 -0.66763<br>C -0.07162 1.41525 -1.13633<br>N -1.08606 2.39159 -1.38993<br>C -3.00376 3.66445 -0.72161<br>C -4.09200 3.92424 0.09691<br>C -4.39252 3.06407 1.15306<br>C -3.59172 1.95069 1.36561<br>C -2.47809 1.68648 0.55994<br>C -2.16871 2.55642 -0.50900<br>C 1.09520 1.99075 -0.33904<br>C 0.86718 2.62335 0.89017<br>C 1.91567 3.22114 1.58419<br>C 3.20427 3.21116 1.05631<br>C 3.43471 2.60227 -0.17402<br>C 2.38962 1.99680 -0.86689<br>H -1.64312 0.05100 -1.34986<br>H -2.78112 4.32575 -1.55589<br>H -4.71153 4.79451 -0.09789<br>H -5.24744 3.24961 1.79534<br>H -3.82254 1.26005 2.17264<br>H -0.13145 2.66507 1.30955<br>H 1.71854 3.70320 2.53764<br>H 4.02140 3.68005 1.59749<br>H 4.43399 2.59064 -0.59947<br>H 2.58046 1.52330 -1.82240<br>O 1.02876 -0.32425 -2.02556<br>O 0.38044 0.91128 -2.40700<br>H -0.71065 3.27219 -1.71741								
**Double-bond dimers**								
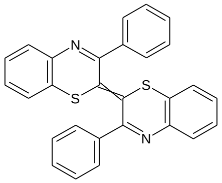<br>C -0.98434 0.29506 4.27998<br>C -0.78837 0.77019 2.98157<br>C -0.04188 1.94568 2.75199<br>C 0.53261 2.59639 3.85561<br>C 0.36411 2.10523 5.14127<br>C -0.41039 0.96173 5.35642	-1982.05731	-1981.64881	-1981.73105	-1983.18691	-1982.86064	-1983.53759	-1983.21132	0.0

N 0.03790 2.56192 1.51437<br>C -0.15152 1.91638 0.41273<br>C -0.32267 0.43894 0.40241<br>S -1.49413 -0.09011 1.61496<br>C 0.32267 -0.43894 -0.40241<br>C 0.15152 -1.91638 -0.41273<br>N -0.03790 -2.56192 -1.51437<br>C 0.04188 -1.94568 -2.75199<br>C 0.78837 -0.77019 -2.98157<br>S 1.49413 0.09011 -1.61496<br>C 0.98434 -0.29506 -4.27998<br>C 0.41039 -0.96173 -5.35642<br>C -0.36411 -2.10523 -5.14127<br>C -0.53261 -2.59639 -3.85561<br>C 0.26997 -2.70456 0.83582<br>C -0.26997 2.70456 -0.83582<br>H 1.09834 3.50392 3.66718<br>H 0.81969 2.62068 5.98147<br>H -0.55970 0.58278 6.36318<br>H -1.56371 -0.61020 4.43871<br>H -1.09834 -3.50392 -3.66718<br>H -0.81969 -2.62068 -5.98147<br>H 0.55970 -0.58278 -6.36318<br>H 1.56371 0.61020 -4.43871<br>C 1.03885 -2.24432 1.91124<br>C 1.19810 -3.02795 3.04944<br>C 0.58124 -4.27506 3.13252<br>C -0.18791 -4.73908 2.06485<br>C -0.33607 -3.96429 0.92061<br>H 1.52168 -1.27294 1.85326<br>H 1.80509 -2.66204 3.87261<br>H 0.70076 -4.88423 4.02416<br>H -0.67210 -5.70983 2.12434<br>H -0.92121 -4.31860 0.07824<br>C 0.33607 3.96429 -0.92061<br>C 0.18791 4.73908 -2.06485<br>C -0.58124 4.27506 -3.13252<br>C -1.19810 3.02795 -3.04944<br>C -1.03885 2.24432 -1.91124<br>H 0.92121 4.31860 -0.07824<br>H 0.67210 5.70983 -2.12434<br>H -0.70076 4.88423 -4.02416<br>H -1.80509 2.66204 -3.87261<br>H -1.52168 1.27294 -1.85326								
**Oxygen species**								
O_2<br>O 0.00000 0.00000 0.60144<br>O 0.00000 0.00000 -0.60144	-150.17434	-150.16711	-150.19038	-150.26391	-150.27995	-150.31368	-150.32972	0.0
H_2O_2<br>O 0.00000 0.71589 -0.05491	-151.39619	-151.36500	-151.39074	-151.48905	-151.48360	-151.54187	-151.53642	0.0

O 0.00000 -0.71589 -0.05491<br>H -0.81094 -0.89788 0.43932<br>H 0.81094 0.89788 0.43932								
H_2O<br>O 0.00000 0.00000 0.11621<br>H 0.00000 0.76535 -0.46485<br>H 0.00000 -0.76535 -0.46485	-76.34912	-76.32371	-76.34512			-151.54187	-151.53787	0.0
HOO·<br>O 0.05526 0.71218 0.00000<br>O 0.05526 -0.60358 0.00000<br>H -0.88411 -0.86876 0.00000	-150.75733	-150.73907	-150.76503	-150.84599	-150.85368	-150.89687	-150.90456	0.0
HO·<br>O 0.00000 0.00000 0.10836<br>H 0.00000 0.00000 -0.86687	-75.65706	-75.64516	-75.66539	-75.70173	-75.71005	-75.72841	-75.73674	0.0

Table S4. Neutral forms in methanol. For each chemical species, values are reported for the most stable conformer / geometric isomer identified. $G_{\mathrm{RRHO,calc}}$ [M062X / 6-311++G(2d,2p) / SMD] = G_{el} [M062X / 6-311++G(2d,2p) / SMD // PBE0 / 6-31+G(d,p) / PCM] - G_{el} [PBE0 / 6-31+G(d,p) / PCM] + G_{RRHO} [PBE0 / 6-31+G(d,p) / PCM]; and likewise for $G_{\mathrm{RRHO,calc}}$ [PBE0 / 6-31+G(d,p) / SMD] and for $G_{\mathrm{RRHO,calc}}$ [M062X / 6-31+G(d,p) / SMD].

Species	PBE0 / 6-31+G(d,p) / PCM			PBE0 / 6-31+G(d,p) / SMD		M062X / 6-31+G(d,p) / SMD		M062X / 6-311++G(2d,2p) / SMD		
**Cartesian coordinates / Å**	**G_{el} / Ha**	**H_{RRHO} / Ha**	**G_{RRHO} / Ha**	**G_{el} / Ha**	**G_{RRHO} / Ha**	**G_{el} / Ha**	**$G_{\mathrm{RRHO,calc}}$ / Ha**	**G_{el} / Ha**	**$G_{\mathrm{RRHO,calc}}$ / Ha**	**$\Delta G_{\mathrm{RRHO,calc}}$ / kcal mol^{-1}**
**Single-bond dimer**										
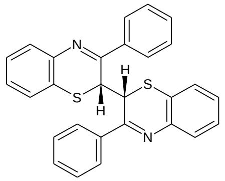<br>S -1.34737 1.03292 -2.39203<br>C -0.76722 0.11693 -0.93950<br>C -1.21798 0.87229 0.30123<br>N -1.02530 2.13869 0.43867<br>C 0.02861 4.17258 -0.25740<br>C 0.43583 5.06094 -1.24298<br>C 0.27026 4.73053 -2.59138<br>C -0.27026 3.49878 -2.94633<br>C -0.65567 2.58986 -1.95709<br>C -0.52805 2.92715 -0.59168<br>C -1.84818 0.13898 1.42394<br>C -1.86008 0.72464 2.70153<br>C -2.45080 0.07387 3.77661<br>C -3.05056 -1.17523 3.59892<br>C -3.05047 -1.76449 2.33710<br>C -2.44955 -1.11709 1.25933<br>H -1.26575 -0.85076 -1.00829<br>H 0.12159 4.42282 0.79556<br>H 0.86823 6.01724 -0.96444<br>H 0.57149 5.42798 -3.36726<br>H -0.38077 3.23072 -3.99341<br>H -1.38960 1.69333 2.83346<br>H -2.44033 0.53823 4.75859<br>H -3.51336 -1.68422 4.43952<br>H -3.51990 -2.73206 2.18547<br>H -2.48078 -1.60099 0.28861<br>S 1.34737 -1.03292 -2.39203<br>C 0.76722 -0.11693 -0.93950<br>C 1.21798 -0.87229 0.30123<br>N 1.02530 -2.13869 0.43867<br>C -0.02861 -4.17258 -0.25740	-1983.29791	-1982.86500	-1982.94787	-1983.31837	-1982.96832	-1984.44865	-1984.09860	-1984.79773	-1984.44769	1.7

C -0.43583 -5.06094 -1.24298<br>C -0.27026 -4.73053 -2.59138<br>C 0.27026 -3.49878 -2.94633<br>C 0.65567 -2.58986 -1.95709<br>C 0.52805 -2.92715 -0.59168<br>C 1.84818 -0.13898 1.42394<br>C 1.86008 -0.72464 2.70153<br>C 2.45080 -0.07387 3.77661<br>C 3.05056 1.17523 3.59892<br>C 3.05047 1.76449 2.33710<br>C 2.44955 1.11709 1.25933<br>H 1.26575 0.85076 -1.00829<br>H -0.12159 -4.42282 0.79556<br>H -0.86823 -6.01724 -0.96444<br>H -0.57149 -5.42798 -3.36726<br>H 0.38077 -3.23072 -3.99341<br>H 1.38960 -1.69333 2.83346<br>H 2.44033 -0.53823 4.75859<br>H 3.51336 1.68422 4.43952<br>H 3.51990 2.73206 2.18547<br>H 2.48078 1.60099 0.28861										
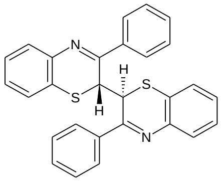<br>S 1.00027 -0.75723 -1.84385<br>C -0.28417 -0.36409 -0.61977<br>C -0.97808 -1.66332 -0.24538<br>N -0.31501 -2.70859 0.11063<br>C 1.74075 -3.75167 0.76503<br>C 3.10284 -3.96606 0.60781<br>C 3.82226 -3.22229 -0.33270<br>C 3.18225 -2.24109 -1.08221<br>C 1.81735 -1.99979 -0.90410<br>C 1.07044 -2.77447 0.01103<br>C -2.45647 -1.74638 -0.25337<br>C -3.09118 -2.73139 0.52202<br>C -4.47459 -2.85275 0.52213<br>C -5.25496 -2.00107 -0.26376<br>C -4.63712 -1.02649 -1.04339<br>C -3.24988 -0.89318 -1.03301<br>H -0.96460 0.30970 -1.14221<br>H 1.15801 -4.34297 1.46566<br>H 3.60353 -4.72340 1.20351<br>H 4.88445 -3.39833 -0.47411<br>H 3.74341 -1.64413 -1.79574<br>H -2.47911 -3.39044 1.12892	-1983.29751	-1982.86470	-1982.94916	-1983.31826	-1982.96991	-1984.44902	-1984.10067	-1984.79871	-1984.45036	0.0

H -4.94843 -3.61177 1.13812<br>H -6.33686 -2.09775 -0.26610<br>H -5.23341 -0.36374 -1.66372<br>H -2.79982 -0.13185 -1.66208<br>S -1.00027 0.75723 1.84385<br>C 0.28417 0.36409 0.61977<br>C 0.97808 1.66332 0.24538<br>N 0.31501 2.70859 -0.11063<br>C -1.74075 3.75167 -0.76503<br>C -3.10284 3.96606 -0.60781<br>C -3.82226 3.22229 0.33270<br>C -3.18225 2.24109 1.08221<br>C -1.81735 1.99979 0.90410<br>C -1.07044 2.77447 -0.01103<br>C 2.45647 1.74638 0.25337<br>C 3.09118 2.73139 -0.52202<br>C 4.47459 2.85275 -0.52213<br>C 5.25496 2.00107 0.26376<br>C 4.63712 1.02649 1.04339<br>C 3.24988 0.89318 1.03301<br>H 0.96460 -0.30970 1.14221<br>H -1.15801 4.34297 -1.46566<br>H -3.60353 4.72340 -1.20351<br>H -4.88445 3.39833 0.47411<br>H -3.74341 1.64413 1.79574<br>H 2.47911 3.39044 -1.12892<br>H 4.94843 3.61177 -1.13812<br>H 6.33686 2.09775 0.26610<br>H 5.23341 0.36374 1.66372<br>H 2.79982 0.13185 1.66208										
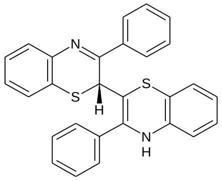<br><br>S -1.66149 -2.01085 -1.34836<br>C -0.46001 -1.13796 -0.26838<br>C -1.07181 -0.92777 1.11193<br>N -2.30503 -0.62886 1.32189<br>C -4.44042 0.15876 0.61134<br>C -5.46391 0.26499 -0.32137<br>C -5.32233 -0.32902 -1.57836<br>C -4.15016 -1.00634 -1.90085<br>C -3.11333 -1.10038 -0.96858<br>C -3.25150 -0.52301 0.30951<br>C -0.17371 -1.00136 2.29388<br>C -0.63928 -0.53127 3.53436<br>C 0.16228 -0.58742 4.66705	-<br>1983.29263	-<br>1982.85921	-<br>1982.94366	-<br>1983.31313	-<br>1982.96416	-<br>1984.44203	-<br>1984.09306	-<br>1984.79221	-<br>1984.44324	4.5

C 1.45292 -1.11572 4.58972<br>C 1.92999 -1.58101 3.36682<br>C 1.12729 -1.52157 2.22900<br>H 0.33813 -1.87672 -0.18689<br>H -4.53547 0.59943 1.59976<br>H -6.37425 0.80032 -0.06874<br>H -6.12153 -0.26078 -2.31056<br>H -4.03177 -1.45907 -2.88167<br>H -1.64147 -0.11975 3.58788<br>H -0.21815 -0.21489 5.61404<br>H 2.08076 -1.16034 5.47501<br>H 2.93206 -1.99341 3.29208<br>H 1.53851 -1.89321 1.29635<br>S -0.89133 1.56696 -0.95107<br>C 0.13687 0.11239 -0.84420<br>C 1.42682 0.20850 -1.25806<br>N 1.97314 1.44292 -1.60387<br>C 2.56731 3.62273 -0.68766<br>C 2.19028 4.78392 -0.01601<br>C 0.88559 4.93785 0.45099<br>C -0.05153 3.92827 0.22936<br>C 0.30919 2.78722 -0.48432<br>C 1.62546 2.61986 -0.93066<br>C 2.36966 -0.93332 -1.35224<br>C 3.61492 -0.87290 -0.70946<br>C 4.51357 -1.93108 -0.81323<br>C 4.18503 -3.05754 -1.56807<br>C 2.95504 -3.12015 -2.22156<br>C 2.05262 -2.06368 -2.11708<br>H 3.59131 3.49282 -1.02929<br>H 2.92632 5.56441 0.15268<br>H 0.59438 5.83789 0.98353<br>H -1.07226 4.03635 0.58517<br>H 3.87075 -0.00309 -0.10971<br>H 5.46959 -1.87754 -0.30054<br>H 4.88831 -3.88128 -1.65090<br>H 2.69984 -3.98787 -2.82276<br>H 1.10527 -2.10259 -2.64804<br>H 2.92685 1.40533 -1.93573										
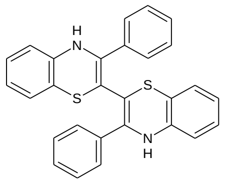<br>S 1.73424 -2.47879 -0.10772<br>C 0.62031 -1.09302 -0.38510<br>C 1.00850 -0.13917 -1.26827<br>N 2.30226 -0.14342 -1.79089	-1983.29245	-1982.85882	-1982.94278	-1983.31390	-1982.96423	-1984.44526	-1984.09559	-1984.79606	-1984.44639	2.5

C 4.64211 0.10761 -1.15274<br>C 5.72637 -0.30814 -0.38276<br>C 5.58092 -1.34203 0.54164<br>C 4.34543 -1.97464 0.68204<br>C 3.27243 -1.59583 -0.12272<br>C 3.40835 -0.53700 -1.02983<br>C 0.14608 0.97201 -1.73087<br>C 0.64498 2.28040 -1.79598<br>C -0.15363 3.32030 -2.26733<br>C -1.45818 3.06550 -2.68707<br>C -1.96021 1.76406 -2.63364<br>C -1.16461 0.72452 -2.16144<br>H 4.74937 0.93197 -1.85336<br>H 6.68427 0.19034 -0.49835<br>H 6.42258 -1.65904 1.14971<br>H 4.21870 -2.78354 1.39604<br>H 1.65442 2.49342 -1.45274<br>H 0.24381 4.33068 -2.30153<br>H -2.07987 3.87521 -3.05837<br>H -2.97196 1.55608 -2.97038<br>H -1.55167 -0.28988 -2.13674<br>S -1.73454 -2.47869 0.10647<br>C -0.62050 -1.09316 0.38455<br>C -1.00857 -0.13976 1.26830<br>N -2.30227 -0.14422 1.79105<br>C -4.64219 0.10721 1.15333<br>C -5.72655 -0.30803 0.38322<br>C -5.58122 -1.34136 -0.54183<br>C -4.34577 -1.97394 -0.68272<br>C -3.27268 -1.59565 0.12215<br>C -3.40846 -0.53735 1.02990<br>C -0.14599 0.97114 1.73129<br>C -0.64480 2.27952 1.79721<br>C 0.15413 3.31920 2.26851<br>C 1.45883 3.06415 2.68764<br>C 1.96069 1.76266 2.63363<br>C 1.16482 0.72337 2.16135<br>H -4.74935 0.93115 1.85447<br>H -6.68442 0.19042 0.49921<br>H -6.42296 -1.65796 -1.15001<br>H -4.21915 -2.78242 -1.39721<br>H -1.65436 2.49278 1.45447<br>H -0.24321 4.32959 2.30330<br>H 2.08075 3.87369 3.05891<br>H 2.97254 1.55446 2.96996<br>H 1.55178 -0.29106 2.13614<br>H 2.48712 0.57649 -2.47491<br>H -2.48703 0.57540 2.47541										
**Single-bond dimer, radical**										

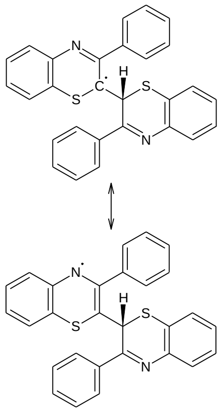<br>S 0.77880 1.55158 -0.55422<br>C -0.31497 0.45740 0.24075<br>C -1.66152 0.79353 0.46135<br>N -2.28451 1.89584 0.04636<br>C -2.40461 4.00511 -1.03349<br>C -1.83747 5.06933 -1.71175<br>C -0.47058 5.05768 -2.01989<br>C 0.31446 3.97400 -1.64914<br>C -0.25943 2.89635 -0.96715<br>C -1.63444 2.88941 -0.63858<br>C -2.55821 -0.14949 1.19925<br>C -2.30237 -0.52133 2.52388<br>C -3.18364 -1.36093 3.20364<br>C -4.32535 -1.84289 2.56515<br>C -4.59001 -1.47118 1.24671<br>C -3.71656 -0.62259 0.57088<br>H -3.46060 3.99656 -0.78026<br>H -2.45268 5.91535 -2.00290<br>H -0.02014 5.89133 -2.54954<br>H 1.37409 3.95834 -1.89011<br>H -1.42359 -0.13518 3.03353<br>H -2.97842 -1.63364 4.23486<br>H -5.00898 -2.50077 3.09402<br>H -5.48018 -1.83918 0.74442<br>H -3.92788 -0.31996 -0.45099<br>S 1.24613 -0.82686 2.20808<br>C 0.28123 -0.86198 0.64360<br>C 1.12573 -1.53357 -0.44012<br>N 2.38832 -1.34046 -0.59471<br>C 4.40327 -0.12259 -0.20298<br>C 5.25765 0.60463 0.61488<br>C 4.88422 0.89091 1.93110<br>C 3.64942 0.46895 2.41358<br>C 2.78020 -0.24735 1.58600	-1982.66679	-1982.24705	-1982.33152	-1982.68568	-1982.35041	-1983.81052	-1983.47525	-1984.16032	-1983.82505	3.0

C 3.15595 -0.56389 0.26482<br>C 0.43987 -2.43785 -1.39317<br>C -0.95808 -2.52712 -1.46417<br>C -1.57097 -3.37626 -2.38312<br>C -0.79948 -4.15663 -3.24086<br>C 0.59396 -4.07945 -3.17712<br>C 1.20628 -3.22858 -2.26643<br>H -0.54129 -1.53092 0.90048<br>H 4.68026 -0.37095 -1.22350<br>H 6.21732 0.94041 0.23381<br>H 5.55130 1.44968 2.58074<br>H 3.35020 0.70252 3.43163<br>H -1.59023 -1.92911 -0.81550<br>H -2.65510 -3.42544 -2.42546<br>H -1.27828 -4.82242 -3.95310<br>H 1.20357 -4.68788 -3.83908<br>H 2.28784 -3.16371 -2.21215										
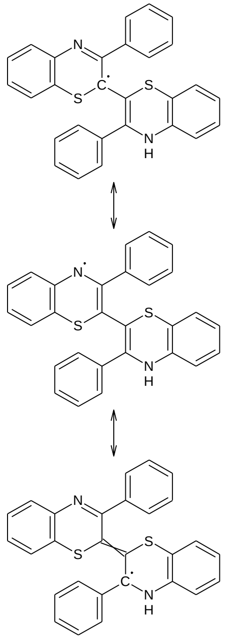<br>S -2.03789 -1.70012 -1.08361<br>C -0.88731 -0.70146 -0.22968<br>C -1.28230 0.12350 0.83886<br>N -2.53133 0.31361 1.26535<br>C -4.86859 -0.08689 1.30939<br>C -6.00837 -0.75082 0.89048<br>C -5.92375 -1.73013 -0.10764<br>C -4.69503 -2.02704 -0.68385	-1982.66840	-1982.24840	-1982.33273	-1982.68839	-1982.35271	-1983.81521	-1983.47953	-1984.16556	-1983.82989	0.0

C -3.54533 -1.34878 -0.26856<br>C -3.60292 -0.36608 0.74775<br>C -0.26122 0.87992 1.62136<br>C 0.90169 0.26559 2.10066<br>C 1.80579 0.97938 2.88446<br>C 1.56344 2.31779 3.19419<br>C 0.40282 2.93437 2.72660<br>C -0.50667 2.21731 1.95333<br>H -4.91116 0.66625 2.09073<br>H -6.96801 -0.51534 1.34079<br>H -6.81349 -2.25860 -0.43567<br>H -4.62465 -2.78140 -1.46307<br>H 1.09342 -0.77943 1.87562<br>H 2.69860 0.48575 3.25840<br>H 2.27179 2.87451 3.80141<br>H 0.20400 3.97553 2.96551<br>H -1.41616 2.69112 1.59631<br>S 1.18399 -2.49752 -0.69472<br>C 0.46727 -0.84644 -0.75673<br>C 1.22675 0.15915 -1.26808<br>N 2.57430 -0.04667 -1.53678<br>C 4.71154 -0.55878 -0.48409<br>C 5.48865 -1.40502 0.30435<br>C 4.93326 -2.55393 0.86605<br>C 3.59560 -2.86589 0.62151<br>C 2.83011 -2.04917 -0.20846<br>C 3.37801 -0.87927 -0.74860<br>C 0.72288 1.52075 -1.55240<br>C -0.49762 1.70674 -2.21690<br>C -0.95099 2.98861 -2.51282<br>C -0.19109 4.10295 -2.15446<br>C 1.02706 3.92767 -1.49941<br>C 1.48325 2.64592 -1.20287<br>H 5.13671 0.35189 -0.89832<br>H 6.52874 -1.15412 0.49015<br>H 5.53514 -3.20787 1.48947<br>H 3.15099 -3.75955 1.05022<br>H -1.08108 0.84205 -2.51855<br>H -1.89444 3.11766 -3.03545<br>H -0.54536 5.10262 -2.38902<br>H 1.62181 4.79002 -1.21236<br>H 2.42177 2.52192 -0.66871<br>H 3.05121 0.73100 -1.97073										
**Single-bond dimer, C2-peroxyl radical**										

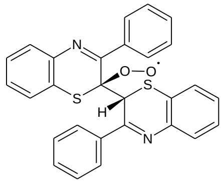<br>S 0.80705 -1.21254 -1.00254<br>C 0.22404 0.40699 -0.49018<br>C 1.33609 1.36571 -0.04539<br>N 2.57620 1.26301 -0.35351<br>C 4.47771 0.40512 -1.49359<br>C 5.12463 -0.56436 -2.24633<br>C 4.43498 -1.71905 -2.62675<br>C 3.10420 -1.89496 -2.26240<br>C 2.45462 -0.91338 -1.50974<br>C 3.13642 0.24619 -1.11227<br>C 0.95817 2.56189 0.75996<br>C 1.75818 2.91170 1.85647<br>C 1.47266 4.04685 2.60787<br>C 0.38961 4.85841 2.26801<br>C -0.40234 4.52792 1.17018<br>C -0.12462 3.38579 0.42157<br>H 4.98826 1.31111 -1.18069<br>H 6.16178 -0.42664 -2.53535<br>H 4.93245 -2.48509 -3.21392<br>H 2.56838 -2.79078 -2.56406<br>H 2.60008 2.27751 2.11664<br>H 2.09557 4.29733 3.46172<br>H 0.16728 5.74560 2.85377<br>H -1.23650 5.16309 0.88694<br>H -0.73498 3.16458 -0.44901<br>S -0.29964 -0.13344 2.22710<br>C -0.90871 0.24534 0.56065<br>C -2.01930 -0.72110 0.15409<br>N -2.04993 -1.96741 0.46522<br>C -1.07080 -3.98614 1.27669<br>C -0.20907 -4.68161 2.11536<br>C 0.61950 -3.98014 2.99404<br>C 0.60076 -2.58792 3.01192<br>C -0.25105 -1.88920 2.15515<br>C -1.10818 -2.58465 1.28235<br>C -3.13490 -0.19954 -0.67322<br>C -3.59151 1.12238 -0.57716<br>C -4.66892 1.55715 -1.34550<br>C -5.29461 0.68547 -2.23448<br>C -4.84289 -0.63100 -2.34376<br>C -3.77827 -1.07077 -1.56638<br>H -1.34360 1.23850 0.67681<br>H -1.73783 -4.50959 0.59783	-2132.84351	-2132.41421	-2132.50282	-2132.86349	-2132.52280	-2134.08919	-2133.74850	-2134.48558	-2134.14489	0.3

H -0.19003 -5.76691 2.09413<br>H 1.28751 -4.51497 3.66251<br>H 1.25611 -2.04118 3.68441<br>H -3.13719 1.82255 0.11748<br>H -5.01877 2.58041 -1.24611<br>H -6.12809 1.02940 -2.84004<br>H -5.31981 -1.31499 -3.03986<br>H -3.42199 -2.09266 -1.64602<br>O -0.48516 1.08164 -1.62885<br>O 0.27147 1.21343 -2.67804										
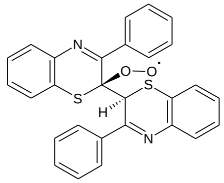<br>S -1.17325 -1.01276 -1.59707<br>C 0.19088 -0.46285 -0.50778<br>C 0.92395 -1.72794 -0.02905<br>N 0.26998 -2.68568 0.52713<br>C -1.73741 -3.58949 1.47853<br>C -3.10806 -3.80024 1.43935<br>C -3.88125 -3.18846 0.44755<br>C -3.28705 -2.33544 -0.47589<br>C -1.91178 -2.09168 -0.41723<br>C -1.11600 -2.74147 0.54830<br>C 2.37476 -1.93639 -0.25668<br>C 2.79897 -3.24887 -0.52337<br>C 4.14465 -3.53576 -0.71996<br>C 5.09452 -2.51610 -0.64702<br>C 4.68547 -1.21078 -0.38016<br>C 3.33694 -0.91819 -0.19351<br>H -1.11343 -4.08478 2.21656<br>H -3.57560 -4.45519 2.16816<br>H -4.95157 -3.36509 0.40173<br>H -3.89033 -1.83849 -1.23007<br>H 2.05536 -4.03716 -0.58112<br>H 4.45209 -4.55492 -0.93554<br>H 6.14679 -2.73765 -0.80066<br>H 5.41810 -0.41156 -0.31552<br>H 3.04780 0.10268 0.02135<br>S 0.91028 0.76756 1.95074<br>C -0.32378 0.41798 0.66829<br>C -0.96402 1.72209 0.21056<br>N -0.28797 2.78949 -0.02890<br>C 1.76485 3.96273 -0.37253<br>C 3.09165 4.22077 -0.05745<br>C 3.74362 3.44124 0.90214	-<br>2132.84353	-<br>2132.41399	-<br>2132.50126	-<br>2132.86278	-<br>2132.52051	-<br>2134.08990	-<br>2133.74763	-<br>2134.48627	-<br>2134.14400	0.8

C 3.07663 2.38403 1.51327<br>C 1.75147 2.10092 1.17090<br>C 1.06940 2.90757 0.23767<br>C -2.43188 1.78551 0.01751<br>C -2.95552 2.68735 -0.92311<br>C -4.32754 2.80261 -1.10650<br>C -5.20541 2.03133 -0.34133<br>C -4.69717 1.14166 0.60226<br>C -3.32068 1.01121 0.77510<br>H -1.06288 -0.19744 1.18653<br>H 1.22942 4.57649 -1.09110<br>H 3.61469 5.03856 -0.54369<br>H 4.77483 3.65063 1.17017<br>H 3.58735 1.76579 2.24671<br>H -2.26781 3.28501 -1.51242<br>H -4.71540 3.49411 -1.84900<br>H -6.27855 2.12419 -0.48121<br>H -5.37143 0.54398 1.20865<br>H -2.95672 0.31973 1.52882<br>O 1.09083 0.33067 -1.31612<br>O 0.59786 0.68155 -2.47311										
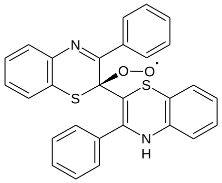<br>S -2.13102 -0.78424 -1.29878<br>C -0.91808 -0.29379 -0.04940<br>C -1.21079 1.09211 0.55749<br>N -2.36882 1.64186 0.61174<br>C -4.72987 1.80263 0.38776<br>C -5.94652 1.38786 -0.13567<br>C -5.98916 0.29868 -1.01024<br>C -4.81996 -0.37682 -1.34617<br>C -3.59905 0.03761 -0.80935<br>C -3.53735 1.13915 0.05875<br>C -0.09943 1.81013 1.23546<br>C 0.91377 1.14370 1.93807<br>C 1.89941 1.86651 2.60816<br>C 1.89658 3.25928 2.57577<br>C 0.89192 3.93091 1.87640<br>C -0.09987 3.21272 1.21869<br>H -4.66683 2.65008 1.06408<br>H -6.85956 1.91123 0.13060<br>H -6.93546 -0.03081 -1.42879<br>H -4.85470 -1.22908 -2.01934<br>H 0.92614 0.06076 1.99072	-2132.84351	-2132.41373	-2132.50175	-2132.86359	-2132.52184	-2134.09018	-2133.74843	-2134.48706	-2134.14531	0.0

H 2.67009 1.33495 3.15905<br>H 2.67092 3.81936 3.09228<br>H 0.88298 5.01671 1.84243<br>H -0.88459 3.72929 0.67494<br>C 0.42361 -0.40797 -0.71807<br>S 0.75218 0.93772 -1.84434<br>C 2.46716 1.17529 -1.46362<br>C 3.23709 0.03156 -1.22322<br>N 2.60841 -1.21830 -1.16372<br>C 1.34567 -1.39829 -0.60903<br>C 3.06035 2.43557 -1.48144<br>C 4.43958 2.55579 -1.31312<br>C 5.21239 1.41509 -1.09706<br>C 4.61536 0.15798 -1.03382<br>C 1.17381 -2.70723 0.07062<br>C 0.18456 -3.60275 -0.35126<br>C 0.06096 -4.84913 0.25712<br>C 0.92361 -5.21278 1.29060<br>C 1.91934 -4.32929 1.70807<br>C 2.05154 -3.08623 1.09486<br>H 2.44589 3.31491 -1.65176<br>H 4.90527 3.53558 -1.35313<br>H 6.28665 1.50124 -0.96371<br>H 5.21525 -0.72745 -0.83957<br>H -0.48259 -3.32436 -1.16168<br>H -0.70744 -5.53860 -0.08020<br>H 0.82441 -6.18430 1.76608<br>H 2.59372 -4.60735 2.51271<br>H 2.82490 -2.39781 1.42635<br>O -0.92243 -1.22637 1.11254<br>O -2.11489 -1.42042 1.59248<br>H 3.22811 -2.01037 -1.06535										
**Single-bond dimer, C3-peroxyl radical**										
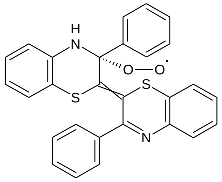<br>S 1.79908 0.17750 1.44716<br>C 0.48599 0.62930 0.35134<br>C 0.49983 2.08997 0.06331<br>N 1.58308 2.70383 -0.27783<br>C 3.85950 2.63390 -1.02661<br>C 5.14628 2.11908 -0.95796<br>C 5.42061 1.03387 -0.12007<br>C 4.39878 0.44535 0.61812<br>C 3.09608 0.94029 0.52235	-2132.84761	-2132.41794	-2132.50691	-2132.86794	-2132.52724	-2134.09301	-2133.75231	-2134.49060	-2134.14990	0.0

C 2.81261 2.06092 -0.28656<br>C -0.72652 2.90078 0.25612<br>C -1.71642 2.50397 1.16451<br>C -2.83264 3.30581 1.38430<br>C -2.98081 4.50696 0.69099<br>C -1.99917 4.90711 -0.21748<br>C -0.87583 4.11447 -0.42826<br>H 3.63350 3.49786 -1.64501<br>H 5.94184 2.57136 -1.54230<br>H 6.42933 0.63834 -0.04828<br>H 4.60492 -0.41174 1.25314<br>H -1.60866 1.56941 1.70767<br>H -3.58836 2.99114 2.09828<br>H -3.85636 5.12816 0.85696<br>H -2.11072 5.83913 -0.76433<br>H -0.10753 4.42047 -1.13120<br>S -1.51877 0.26375 -1.42417<br>C -0.38685 -0.26096 -0.17770<br>C -0.40619 -1.72972 0.19302<br>N -1.46823 -2.45163 -0.37465<br>C -3.88773 -2.71943 -0.13295<br>C -5.16699 -2.20016 -0.31423<br>C -5.34405 -0.89918 -0.78691<br>C -4.23306 -0.11385 -1.08753<br>C -2.95281 -0.64313 -0.93280<br>C -2.77059 -1.94406 -0.45036<br>C 0.89386 -2.48091 -0.06040<br>C 1.33236 -3.49246 0.79650<br>C 2.47345 -4.22916 0.48017<br>C 3.18078 -3.96355 -0.69018<br>C 2.73739 -2.96017 -1.55174<br>C 1.59670 -2.22575 -1.24153<br>H -3.74972 -3.72743 0.24868<br>H -6.02989 -2.81317 -0.07180<br>H -6.34237 -0.49267 -0.91500<br>H -4.35574 0.90198 -1.45187<br>H 0.80031 -3.70528 1.71776<br>H 2.81038 -5.00871 1.15727<br>H 4.07240 -4.53517 -0.93097<br>H 3.27959 -2.74767 -2.46856<br>H 1.25450 -1.44876 -1.91935<br>H -1.41109 -3.44271 -0.17871<br>O -0.58623 -1.63603 1.74993<br>O -1.27410 -2.61718 2.23538											
**Single-bond dimer, dioxolane radical**											

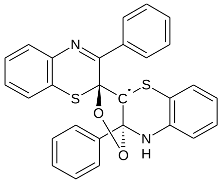<br>S 1.75824 -1.85592 0.45550<br>C 1.02793 -0.26057 -0.12216<br>C 1.91951 0.87372 0.39880<br>N 3.20116 0.83504 0.30088<br>C 5.23152 -0.07637 -0.56746<br>C 6.03136 -1.15134 -0.92565<br>C 5.52899 -2.45392 -0.83632<br>C 4.22140 -2.67233 -0.41843<br>C 3.40290 -1.58829 -0.08410<br>C 3.90860 -0.27391 -0.13977<br>C 1.31352 2.07172 1.02243<br>C 1.93291 2.63312 2.14826<br>C 1.41168 3.77831 2.74204<br>C 0.27406 4.38636 2.20938<br>C -0.33845 3.84251 1.08100<br>C 0.17222 2.68787 0.49334<br>H 5.60895 0.94140 -0.60368<br>H 7.04965 -0.98113 -1.26154<br>H 6.15451 -3.30073 -1.10246<br>H 3.82455 -3.68220 -0.36484<br>H 2.81882 2.15505 2.55518<br>H 1.89224 4.19560 3.62223<br>H -0.13235 5.28130 2.67189<br>H -1.21605 4.31829 0.65297<br>H -0.30633 2.28645 -0.39360<br>S -1.07645 -0.58082 1.74320<br>C -0.42941 -0.30295 0.19075<br>C -1.15310 -0.57761 -1.10360<br>N -1.99693 -1.74254 -1.02398<br>C -3.97382 -2.76259 -0.10004<br>C -4.80278 -3.07283 0.96849<br>C -4.50692 -2.60480 2.25093<br>C -3.36962 -1.83218 2.44314<br>C -2.51896 -1.53502 1.37282<br>C -2.81413 -1.99066 0.07699<br>C -1.88107 0.63845 -1.67461<br>C -1.54512 1.15884 -2.92611<br>C -2.24016 2.25361 -3.44081<br>C -3.27266 2.83765 -2.71043<br>C -3.61064 2.32063 -1.45872<br>C -2.91912 1.22883 -0.94219<br>H -4.21468 -3.12050 -1.09806<br>H -5.68993 -3.67469 0.79589	-<br>2132.85449	-<br>2132.42447	-<br>2132.51148	-<br>2132.87517	-<br>2132.53216	-<br>2134.09916	-<br>2133.75615	-<br>2134.49599	-<br>2134.15298	1.0

H -5.15520 -2.83501 3.09008<br>H -3.12703 -1.45493 3.43327<br>H -0.73910 0.70834 -3.49526<br>H -1.96924 2.65016 -4.41523<br>H -3.81094 3.69180 -3.11104<br>H -4.41294 2.77035 -0.88075<br>H -3.18973 0.84295 0.03701<br>O 1.03127 -0.14185 -1.53914<br>O -0.06384 -0.96602 -1.96514<br>H -2.46628 -1.92658 -1.90326										
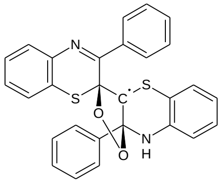<br>S 1.46007 -1.99991 1.48483<br>C 0.95125 -0.29546 0.92057<br>C 2.17882 0.35533 0.28595<br>N 2.90086 -0.26597 -0.57724<br>C 3.26179 -2.05417 -2.12169<br>C 3.21432 -3.39659 -2.47129<br>C 2.64424 -4.32492 -1.59534<br>C 2.09778 -3.90206 -0.38806<br>C 2.11983 -2.54701 -0.04536<br>C 2.72249 -1.60610 -0.90567<br>C 2.54108 1.75127 0.61959<br>C 3.90148 2.09282 0.65201<br>C 4.29580 3.40171 0.90618<br>C 3.33526 4.39175 1.11869<br>C 1.98108 4.06187 1.08004<br>C 1.58059 2.74947 0.84116<br>H 3.72418 -1.32047 -2.77551<br>H 3.63207 -3.72454 -3.41826<br>H 2.61518 -5.37843 -1.85698<br>H 1.63865 -4.61878 0.28695<br>H 4.64170 1.31706 0.48255<br>H 5.35277 3.64971 0.94050<br>H 3.64153 5.41539 1.31484<br>H 1.22919 4.83013 1.23640<br>H 0.52288 2.50798 0.81088<br>S -0.39249 -0.06473 -1.60369<br>C -0.28636 -0.27994 0.08700<br>C -1.42443 0.10150 0.99735<br>N -1.77744 1.47561 0.70713<br>C -3.11324 2.84790 -0.77867<br>C -3.53196 3.20007 -2.05442<br>C -3.05073 2.51228 -3.17117	-2132.85448	-2132.42450	-2132.51041	-2132.87432	-2132.53026	-2134.09887	-2133.75481	-2134.49527	-2134.15121	2.1

C -2.12997 1.48784 -2.99451<br>C -1.68282 1.14632 -1.71383<br>C -2.19063 1.80930 -0.58041<br>C -2.59210 -0.87858 1.04296<br>C -2.32574 -2.25044 1.11054<br>C -3.36978 -3.16715 1.17449<br>C -4.69288 -2.72207 1.16908<br>C -4.96282 -1.35753 1.10148<br>C -3.91600 -0.43716 1.03800<br>H -3.50572 3.37175 0.08966<br>H -4.24878 4.00662 -2.17561<br>H -3.38612 2.77340 -4.16967<br>H -1.73618 0.95336 -3.85515<br>H -1.29490 -2.59549 1.11589<br>H -3.15152 -4.22989 1.22659<br>H -5.50892 -3.43750 1.21510<br>H -5.98938 -1.00309 1.09524<br>H -4.14585 0.62155 0.97770<br>O 0.56878 0.39217 2.09394<br>O -0.82087 0.02778 2.28767<br>H -2.29739 1.92695 1.45050										
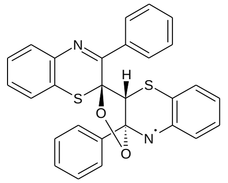<br>S 1.44917 -1.52601 0.03215<br>C 0.83096 0.15454 -0.30826<br>C 1.84410 1.19020 0.19580<br>N 3.11301 1.05470 0.06109<br>C 5.04749 0.04345 -0.90266<br>C 5.74177 -1.05773 -1.38149<br>C 5.11989 -2.30992 -1.41796<br>C 3.80121 -2.45001 -1.00052<br>C 3.09280 -1.33562 -0.54083<br>C 3.71604 -0.07638 -0.47476<br>C 1.36235 2.44145 0.83956<br>C 0.52411 3.33671 0.16130<br>C 0.13050 4.52615 0.76987<br>C 0.55959 4.82805 2.06218<br>C 1.39677 3.94252 2.74001<br>C 1.80271 2.75804 2.12976<br>H 5.51487 1.02207 -0.84454<br>H 6.76891 -0.94832 -1.71546<br>H 5.66114 -3.17866 -1.78052<br>H 3.31257 -3.41940 -1.04154<br>H 0.20553 3.11997 -0.85514	-<br>2132.85417	-<br>2132.42510	-<br>2132.51257	-<br>2132.87505	-<br>2132.53345	-<br>2134.09824	-<br>2133.75664	-<br>2134.49366	-<br>2134.15207	1.6

H -0.50840 5.21932 0.23055<br>H 0.24603 5.75307 2.53723<br>H 1.73691 4.17411 3.74519<br>H 2.45956 2.06786 2.65109<br>S -1.08021 -0.27648 1.81787<br>C -0.62362 0.28848 0.17397<br>C -1.44247 -0.32513 -0.98951<br>N -1.81222 -1.70547 -0.93900<br>C -2.27181 -3.77611 0.06416<br>C -2.40203 -4.59622 1.15938<br>C -2.12474 -4.09404 2.44462<br>C -1.70665 -2.77583 2.61162<br>C -1.56804 -1.92830 1.51441<br>C -1.86800 -2.40608 0.19124<br>C -2.67587 0.51778 -1.29524<br>C -3.85773 0.31210 -0.57817<br>C -4.97178 1.11404 -0.81903<br>C -4.91556 2.12155 -1.78118<br>C -3.73906 2.32348 -2.50152<br>C -2.62179 1.52562 -2.26017<br>H -0.84072 1.35734 0.19985<br>H -2.47904 -4.13102 -0.94075<br>H -2.71536 -5.62794 1.03516<br>H -2.22300 -4.73608 3.31434<br>H -1.47650 -2.40554 3.60693<br>H -3.91740 -0.47757 0.16540<br>H -5.88562 0.94647 -0.25631<br>H -5.78583 2.74292 -1.97179<br>H -3.68906 3.10198 -3.25751<br>H -1.70826 1.67810 -2.82580<br>O 0.66387 0.44707 -1.68227<br>O -0.51039 -0.27455 -2.10411										
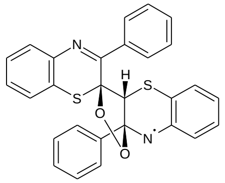<br>S 1.50720 -1.18567 0.84387<br>C 0.87549 0.13592 -0.23239<br>C 1.73470 1.40008 -0.11194<br>N 3.00263 1.40653 0.09330<br>C 5.16224 0.40663 0.21502<br>C 6.00150 -0.67115 0.45606<br>C 5.45795 -1.90979 0.81191<br>C 4.08024 -2.06869 0.90707<br>C 3.23525 -0.98425 0.65159<br>C 3.76821 0.26922 0.31056	-2132.84384	-2132.41453	-2132.49942	-2132.86390	-2132.51948	-2134.08806	-2133.74363	-2134.48364	-2134.13921	9.6

C 1.09852 2.72950 -0.32297<br>C 0.32079 3.00885 -1.45579<br>C -0.20737 4.28395 -1.64574<br>C 0.02344 5.28800 -0.70595<br>C 0.79728 5.01606 0.42209<br>C 1.33774 3.74668 0.60909<br>H 5.55907 1.38180 -0.05171<br>H 7.07730 -0.55039 0.37477<br>H 6.10887 -2.75634 1.00876<br>H 3.65534 -3.03295 1.17193<br>H 0.14932 2.23948 -2.20356<br>H -0.79706 4.49262 -2.53370<br>H -0.39535 6.27919 -0.85404<br>H 0.98232 5.79369 1.15756<br>H 1.94761 3.53195 1.48162<br>S -1.30686 0.15373 1.66223<br>C -0.61642 0.29501 0.01159<br>C -1.25102 -0.67095 -0.99679<br>N -2.59580 -0.29529 -1.29133<br>C -4.73566 0.37737 -0.60591<br>C -5.66187 0.73634 0.34531<br>C -5.26340 0.88219 1.68516<br>C -3.93158 0.69263 2.04788<br>C -2.97199 0.33562 1.10325<br>C -3.36485 0.12845 -0.27541<br>C -1.18670 -2.18286 -0.75962<br>C -0.37853 -2.97682 -1.58018<br>C -0.33136 -4.35788 -1.40639<br>C -1.09413 -4.96811 -0.41258<br>C -1.91891 -4.18655 0.39425<br>C -1.97358 -2.80573 0.21595<br>H -0.89608 1.28870 -0.34889<br>H -5.01483 0.24398 -1.64660<br>H -6.69632 0.90428 0.06314<br>H -5.98715 1.16337 2.44366<br>H -3.62983 0.84279 3.08085<br>H 0.22247 -2.51118 -2.35234<br>H 0.30552 -4.95645 -2.05154<br>H -1.05620 -6.04505 -0.27558<br>H -2.53321 -4.65038 1.16061<br>H -2.64625 -2.22799 0.83944<br>O 0.90246 -0.23691 -1.63696<br>O -0.45806 -0.31273 -2.14135										

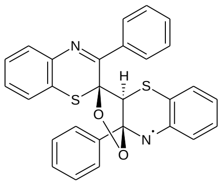<br>S -0.46197 -2.46280 -1.64436<br>C 0.12105 -0.84058 -0.99491<br>C 1.55271 -1.03613 -0.50365<br>N 1.84376 -1.99382 0.30091<br>C 1.19600 -3.73411 1.80972<br>C 0.41302 -4.83018 2.14326<br>C -0.62727 -5.22707 1.29798<br>C -0.90153 -4.50536 0.14080<br>C -0.13709 -3.38009 -0.17995<br>C 0.94102 -2.99372 0.64492<br>C 2.63716 -0.08837 -0.86167<br>C 3.52654 0.30422 0.14951<br>C 4.59518 1.14746 -0.13557<br>C 4.80164 1.59792 -1.43960<br>C 3.93082 1.20111 -2.45358<br>C 2.84960 0.37034 -2.16941<br>H 2.02889 -3.42844 2.43616<br>H 0.62004 -5.38687 3.05212<br>H -1.23386 -6.09294 1.54563<br>H -1.72180 -4.80053 -0.50734<br>H 3.36236 -0.05322 1.16111<br>H 5.26707 1.45345 0.66125<br>H 5.63797 2.25356 -1.66475<br>H 4.09271 1.53729 -3.47361<br>H 2.18934 0.06260 -2.97232<br>S -0.36625 0.07433 1.74868<br>C -0.89017 -0.25662 0.05685<br>C -1.44711 0.98749 -0.68103<br>N -0.73886 2.22182 -0.55126<br>C 0.74044 3.77000 0.40903<br>C 1.56168 4.19581 1.42469<br>C 1.78485 3.36420 2.53922<br>C 1.19539 2.10432 2.60901<br>C 0.36571 1.64963 1.58620<br>C 0.08780 2.49224 0.45261<br>C -2.94196 1.17658 -0.46997<br>C -3.83659 0.22489 -0.97145<br>C -5.20486 0.37515 -0.76394<br>C -5.68935 1.46920 -0.04600<br>C -4.79887 2.41441 0.45814<br>C -3.42817 2.27301 0.24238<br>H -1.69895 -0.97959 0.17635<br>H 0.54049 4.39003 -0.45955	-2132.85572	-2132.42652	-2132.51245	-2132.87643	-2132.53316	-2134.10251	-2133.75924	-2134.49782	-2134.15455	0.0

H 2.03668 5.17035 1.37098<br>H 2.43085 3.69604 3.34612<br>H 1.39214 1.46283 3.46374<br>H -3.46367 -0.63021 -1.52866<br>H -5.89309 -0.36458 -1.16244<br>H -6.75691 1.58314 0.11873<br>H -5.16810 3.26846 1.01843<br>H -2.73939 3.01878 0.62624<br>O 0.06415 0.07231 -2.06482<br>O -1.28809 0.56580 -2.04599										
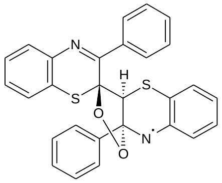<br>S 1.63281 1.69974 -2.09017<br>C 0.29310 0.95198 -1.07645<br>C -0.04651 1.96687 0.01690<br>N 0.88873 2.42826 0.77119<br>C 3.14164 2.41761 1.58454<br>C 4.51139 2.36028 1.37257<br>C 5.00986 2.13362 0.08601<br>C 4.13579 1.93214 -0.97708<br>C 2.75556 1.95044 -0.76002<br>C 2.24024 2.21721 0.52700<br>C -1.43089 2.42069 0.29427<br>C -1.78818 2.65952 1.63075<br>C -3.05285 3.13712 1.95256<br>C -3.97898 3.40102 0.94247<br>C -3.62715 3.18443 -0.38856<br>C -2.36550 2.69214 -0.71491<br>H 2.73486 2.62489 2.57000<br>H 5.19409 2.50820 2.20370<br>H 6.08111 2.10283 -0.08838<br>H 4.52110 1.73709 -1.97374<br>H -1.06313 2.45542 2.41227<br>H -3.31728 3.30172 2.99322<br>H -4.96752 3.77606 1.19171<br>H -4.33642 3.40136 -1.18202<br>H -2.10957 2.54176 -1.75689<br>S 1.14993 -0.89138 1.08475<br>C 0.65628 -0.47519 -0.60066<br>C -0.52111 -1.31478 -1.09353<br>N -0.11725 -2.65836 -1.34187<br>C 0.84079 -4.65302 -0.55575<br>C 1.48631 -5.40802 0.39483<br>C 1.96317 -4.79247 1.56606	-2132.83759	-2132.40829	-2132.49359	-2132.85778	-2132.51378	-2134.08388	-2133.73989	-2134.47958	-2134.13558	11.9

C 1.81720 -3.42128 1.75004<br>C 1.17431 -2.63057 0.79630<br>C 0.61789 -3.25032 -0.38543<br>C -1.87353 -1.35107 -0.38472<br>C -2.91204 -1.95318 -1.11147<br>C -4.18685 -2.06768 -0.56935<br>C -4.44637 -1.58366 0.71388<br>C -3.42187 -0.98334 1.43956<br>C -2.14067 -0.86878 0.89719<br>H 1.49246 -0.79163 -1.23347<br>H 0.44308 -5.10052 -1.46156<br>H 1.62340 -6.47413 0.24474<br>H 2.46971 -5.38059 2.32496<br>H 2.23007 -2.95211 2.63909<br>H -2.71515 -2.32584 -2.11192<br>H -4.97831 -2.53399 -1.14910<br>H -5.44130 -1.67087 1.14111<br>H -3.61351 -0.59323 2.43512<br>H -1.36287 -0.39951 1.48632<br>O -0.79606 0.71452 -1.96081<br>O -0.69335 -0.66207 -2.37488										
**Single-bond dimer, dioxane radical**										
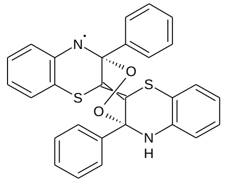<br>S -1.46013 -0.68729 -1.85243<br>C -0.49471 -0.00896 -0.55513<br>C -1.21344 0.19976 0.76233<br>N -2.34734 1.06682 0.65507<br>C -4.46268 1.56892 -0.25174<br>C -5.47647 1.34174 -1.15246<br>C -5.28854 0.42287 -2.20238<br>C -4.06882 -0.22761 -2.36095<br>C -3.02336 0.00210 -1.46701<br>C -3.21573 0.87127 -0.33787<br>C -1.59526 -1.12558 1.43313<br>C -2.63295 -1.13739 2.37149<br>C -2.95684 -2.31067 3.04808<br>C -2.24611 -3.48428 2.79651<br>C -1.20728 -3.47556 1.86767<br>C -0.88263 -2.30176 1.18909<br>H -4.58647 2.25086 0.58402<br>H -6.42334 1.86238 -1.05029<br>H -6.08917 0.23323 -2.91039<br>H -3.91952 -0.89714 -3.20366	-<br>2132.85443	-<br>2132.42486	-<br>2132.51029	-<br>2132.87617	-<br>2132.53202	-<br>2134.10095	-<br>2133.75680	-<br>2134.49778	-<br>2134.15364	2.3

H -3.18427 -0.22423 2.57312<br>H -3.76533 -2.30699 3.77364<br>H -2.49995 -4.39991 3.32311<br>H -0.64565 -4.38356 1.66729<br>H -0.06799 -2.30749 0.47145<br>S 1.71648 -0.15188 -2.17017<br>C 0.82074 0.22082 -0.69285<br>C 1.62557 0.72133 0.48860<br>N 2.89825 0.07541 0.55328<br>C 4.13156 -2.01565 0.48142<br>C 4.56263 -3.13133 -0.22904<br>C 4.09881 -3.37118 -1.52316<br>C 3.20028 -2.48032 -2.10580<br>C 2.79197 -1.34396 -1.40944<br>C 3.24655 -1.10003 -0.10220<br>C 1.81172 2.23858 0.54263<br>C 2.29089 2.80737 1.72748<br>C 2.52304 4.17767 1.80911<br>C 2.27467 4.99746 0.70773<br>C 1.78842 4.43634 -0.47082<br>C 1.55670 3.06308 -0.55289<br>H 4.48470 -1.83896 1.49417<br>H 5.25524 -3.82462 0.23914<br>H 4.42466 -4.24863 -2.07281<br>H 2.82090 -2.65673 -3.10838<br>H 2.46205 2.18175 2.59962<br>H 2.89084 4.60623 2.73710<br>H 2.45270 6.06707 0.77138<br>H 1.58291 5.06652 -1.33146<br>H 1.17286 2.64369 -1.47750<br>H 3.43887 0.30363 1.37625<br>O 0.91408 0.29419 1.67949<br>O -0.35337 0.95253 1.64279										
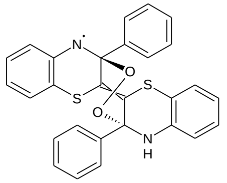<br>S -1.71095 -1.28962 -1.50299<br>C -0.71379 -0.44700 -0.32693<br>C -1.47843 0.42389 0.64292<br>N -2.32938 -0.37487 1.48128<br>C -4.09235 -1.91359 1.75260<br>C -5.02512 -2.78816 1.24528<br>C -4.99422 -3.13272 -0.11889<br>C -4.00015 -2.62670 -0.95155<br>C -3.03586 -1.75388 -0.45037	-<br>2132.85670	-<br>2132.42678	-<br>2132.51244	-<br>2132.87835	-<br>2132.53409	-<br>2134.10427	-<br>2133.76001	-<br>2134.50154	-<br>2134.15728	0.0

C -3.09378 -1.31579 0.91914<br>C -2.24541 1.56195 -0.03750<br>C -1.66507 2.23513 -1.11644<br>C -2.32659 3.30294 -1.71518<br>C -3.57347 3.70945 -1.23815<br>C -4.15014 3.04673 -0.15680<br>C -3.48782 1.97689 0.44543<br>H -4.10769 -1.60787 2.79445<br>H -5.78916 -3.20628 1.89309<br>H -5.73247 -3.81702 -0.52524<br>H -3.95640 -2.93725 -1.99193<br>H -0.69402 1.92196 -1.48976<br>H -1.86945 3.81638 -2.55618<br>H -4.09154 4.54086 -1.70762<br>H -5.11769 3.36150 0.22343<br>H -3.93764 1.46668 1.29109<br>S 1.47107 -1.65974 -1.37649<br>C 0.62011 -0.61550 -0.25819<br>C 1.37383 0.07954 0.86587<br>N 2.31858 -0.84829 1.45885<br>C 4.62560 -1.55592 1.17906<br>C 5.63039 -2.11952 0.40252<br>C 5.37576 -2.48493 -0.92068<br>C 4.10546 -2.29225 -1.45161<br>C 3.08481 -1.75105 -0.66483<br>C 3.33789 -1.36759 0.66004<br>C 2.01173 1.41249 0.48198<br>C 2.39877 2.28784 1.50323<br>C 3.04163 3.48360 1.19972<br>C 3.31155 3.81786 -0.12882<br>C 2.93020 2.94959 -1.14794<br>C 2.28154 1.75141 -0.84398<br>H 4.82904 -1.25550 2.20396<br>H 6.61922 -2.25947 0.82873<br>H 6.16041 -2.91113 -1.53758<br>H 3.89533 -2.57097 -2.48087<br>H 2.17942 2.04063 2.53849<br>H 3.32850 4.15855 2.00108<br>H 3.81323 4.75174 -0.36547<br>H 3.13253 3.20156 -2.18502<br>H 1.98555 1.08755 -1.65138<br>H 2.66818 -0.47749 2.33636<br>O 0.44690 0.25005 1.94036<br>O -0.55741 1.14985 1.46608										
**Single-bond dimer, C2-hydroperoxylalkyl radical**										

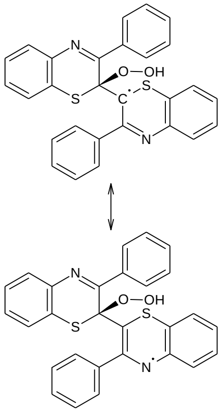<br>S 1.12937 -2.24483 0.02122<br>C 0.39344 -0.79012 -0.83620<br>C 1.28286 0.46160 -0.85771<br>N 2.53938 0.48398 -0.60357<br>C 4.68954 -0.32636 -0.02295<br>C 5.56111 -1.31660 0.40510<br>C 5.06759 -2.59148 0.69676<br>C 3.71199 -2.86686 0.55404<br>C 2.83645 -1.86647 0.12037<br>C 3.31450 -0.57802 -0.17024<br>C 0.68481 1.73193 -1.35980<br>C -0.52279 1.79646 -2.07126<br>C -1.01473 3.01792 -2.52816<br>C -0.31411 4.19687 -2.28593<br>C 0.89164 4.14490 -1.58371<br>C 1.38333 2.92861 -1.12819<br>H 5.04419 0.67314 -0.25708<br>H 6.61956 -1.10113 0.51303<br>H 5.73894 -3.37557 1.03392<br>H 3.33019 -3.85939 0.77823<br>H -1.08031 0.89666 -2.30009<br>H -1.94814 3.04152 -3.08328<br>H -0.70115 5.14741 -2.64205<br>H 1.44701 5.05725 -1.38490<br>H 2.31405 2.88637 -0.57326<br>C -0.99247 -0.63453 -0.23727<br>S -2.17652 -1.62426 -1.05761<br>C -3.63521 -1.27729 -0.15992<br>C -3.63868 -0.36206 0.91561<br>N -2.53496 0.27992 1.41342<br>C -1.31552 0.12313 0.89969<br>C -4.80986 -1.91884 -0.56571<br>C -6.00600 -1.65113 0.08672<br>C -6.03539 -0.73609 1.14762	-2132.85469	-2132.42598	-2132.51477	-2132.87544	-2132.53552	-2134.10488	-2133.76495	-2134.50242	-2134.16249	0.0

C -4.87190 -0.10675 1.55342<br>C -0.27917 0.87103 1.68179<br>C -0.37473 2.26440 1.77377<br>C 0.50481 2.98055 2.57983<br>C 1.48057 2.31077 3.31938<br>C 1.56702 0.92152 3.24995<br>C 0.69224 0.20406 2.43484<br>H -4.78377 -2.62202 -1.39398<br>H -6.91532 -2.15124 -0.23170<br>H -6.97127 -0.52342 1.65538<br>H -4.87060 0.59856 2.37909<br>H -1.14114 2.78390 1.20582<br>H 0.42690 4.06285 2.63339<br>H 2.16471 2.86935 3.95199<br>H 2.31211 0.39047 3.83571<br>H 0.75454 -0.87859 2.39796<br>O 0.13857 -1.14440 -2.20764<br>O 1.36470 -1.42329 -2.86079<br>H 1.44879 -2.38344 -2.72847										
**Single-bond dimer, C3-hydroperoxylalkyl radical**										
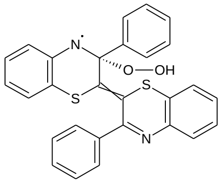<br>S 1.98612 -0.59477 1.48809<br>C 0.89034 0.35096 0.46356<br>C 1.51765 1.60973 -0.02647<br>N 2.71787 1.66313 -0.49717<br>C 4.67806 0.56015 -1.32597<br>C 5.62780 -0.44900 -1.25181<br>C 5.48414 -1.47171 -0.30937<br>C 4.37310 -1.50005 0.52728<br>C 3.39698 -0.50441 0.42961<br>C 3.55271 0.55486 -0.48616<br>C 0.78013 2.89199 0.11317<br>C -0.17014 3.07287 1.12739<br>C -0.80340 4.30257 1.28585<br>C -0.50725 5.36135 0.42774<br>C 0.43585 5.18627 -0.58662<br>C 1.08135 3.96360 -0.73811<br>H 4.78830 1.38409 -2.02526<br>H 6.48903 -0.43249 -1.91280<br>H 6.23246 -2.25485 -0.23235<br>H 4.24913 -2.30782 1.24356<br>H -0.40898 2.25155 1.79752	-2132.83987	-2132.41148	-2132.50132	-2132.86077	-2132.52223	-2134.08529	-2133.74674	-2134.48356	-2134.14501	0.0

H -1.53025 4.43340 2.08246<br>H -1.00861 6.31754 0.54762<br>H 0.66742 6.00490 -1.26217<br>H 1.81813 3.82143 -1.52237<br>S -1.14052 0.74483 -1.21731<br>C -0.36734 -0.04457 0.14637<br>C -1.08097 -1.23249 0.77704<br>N -2.50893 -1.11304 0.92349<br>C -4.69867 -0.51198 0.31111<br>C -5.59051 0.11578 -0.52460<br>C -5.11762 0.89838 -1.59672<br>C -3.75133 1.06295 -1.80160<br>C -2.83264 0.44006 -0.95722<br>C -3.28306 -0.40721 0.10857<br>C -0.77571 -2.56412 0.09008<br>C -1.34885 -3.72598 0.62298<br>C -1.11416 -4.96033 0.02523<br>C -0.31000 -5.04645 -1.11212<br>C 0.26116 -3.89235 -1.64400<br>C 0.03142 -2.65424 -1.04507<br>H -5.03202 -1.13403 1.13615<br>H -6.65833 0.00689 -0.36336<br>H -5.81945 1.39372 -2.26024<br>H -3.39731 1.69514 -2.61127<br>H -1.97555 -3.65536 1.50577<br>H -1.55951 -5.85636 0.44789<br>H -0.12861 -6.00979 -1.58013<br>H 0.89143 -3.95121 -2.52661<br>H 0.48845 -1.76414 -1.46553<br>O -0.59483 -1.41989 2.11837<br>O -1.06002 -0.34182 2.93196<br>H -2.01314 -0.54030 2.98100										
**Single-bond dimer, C2-hydroperoxide**										
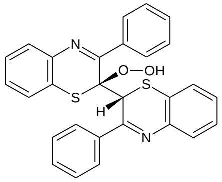<br>S 0.85929 -1.15570 -1.05957<br>C 0.17259 0.44542 -0.52240<br>C 1.24217 1.43586 -0.03478<br>N 2.49805 1.38720 -0.28790<br>C 4.47830 0.62291 -1.35455<br>C 5.19889 -0.30089 -2.09765<br>C 4.57467 -1.47248 -2.53562<br>C 3.23777 -1.70925 -2.23467<br>C 2.51403 -0.77520 -1.48718	-2133.48695	-2133.04505	-2133.13369	-2133.50975	-2133.15649	-2134.74119	-2134.38793	-2135.13877	-2134.78551	0.0

C 3.12858 0.40297 -1.03658<br>C 0.78953 2.61506 0.75897<br>C -0.32135 3.39185 0.39889<br>C -0.66596 4.51698 1.14622<br>C 0.08517 4.87836 2.26303<br>C 1.19455 4.11333 2.62528<br>C 1.54625 2.99519 1.87654<br>H 4.93632 1.54167 -0.99996<br>H 6.24153 -0.11429 -2.33531<br>H 5.12861 -2.20398 -3.11641<br>H 2.75262 -2.61884 -2.57868<br>H -0.89875 3.13782 -0.48446<br>H -1.52125 5.11592 0.84675<br>H -0.18937 5.75282 2.84595<br>H 1.78597 4.38684 3.49446<br>H 2.40892 2.39767 2.15479<br>C -0.91531 0.17913 0.56632<br>S -0.23286 -0.21150 2.20071<br>C -0.10780 -1.96123 2.09227<br>C -0.95279 -2.67710 1.22435<br>N -1.94224 -2.08791 0.44406<br>C -1.98260 -0.83418 0.16379<br>C 0.79394 -2.63782 2.91500<br>C 0.87642 -4.02708 2.86608<br>C 0.06137 -4.74751 1.99012<br>C -0.85069 -4.07485 1.18663<br>C -3.14746 -0.35484 -0.62088<br>C -3.78159 -1.24554 -1.50160<br>C -4.88970 -0.84698 -2.23929<br>C -5.39573 0.44702 -2.10176<br>C -4.78004 1.33728 -1.22442<br>C -3.65950 0.94406 -0.49613<br>H 1.43843 -2.07566 3.58535<br>H 1.58324 -4.54416 3.50816<br>H 0.12981 -5.83009 1.94425<br>H -1.50899 -4.61437 0.51177<br>H -3.38381 -2.25006 -1.60276<br>H -5.35884 -1.54563 -2.92615<br>H -6.26342 0.75869 -2.67602<br>H -5.17115 2.34314 -1.10302<br>H -3.21249 1.65824 0.18850<br>O -0.58456 1.04637 -1.57425<br>O 0.29662 1.41932 -2.62905<br>H 0.22946 0.65275 -3.22287<br>H -1.39893 1.14358 0.72497										

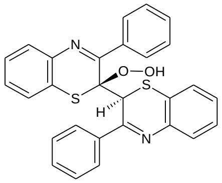<br>S 1.22402 -1.01892 1.58823<br>C -0.18098 -0.44017 0.53622<br>C -0.88179 -1.70551 0.01637<br>N -0.22000 -2.65254 -0.55117<br>C 1.78324 -3.53793 -1.51937<br>C 3.15313 -3.75577 -1.49252<br>C 3.93258 -3.16754 -0.49158<br>C 3.34606 -2.33067 0.45153<br>C 1.97204 -2.07652 0.40347<br>C 1.16735 -2.70590 -0.57060<br>C -2.34204 -1.91617 0.18969<br>C -3.29032 -0.88080 0.15505<br>C -4.64670 -1.16154 0.31210<br>C -5.07851 -2.47168 0.50797<br>C -4.14373 -3.50741 0.54530<br>C -2.79037 -3.23260 0.38893<br>H 1.15327 -4.01600 -2.26381<br>H 3.61419 -4.39779 -2.23681<br>H 5.00239 -3.34967 -0.45332<br>H 3.95477 -1.85239 1.21333<br>H -2.97823 0.14286 -0.01383<br>H -5.36627 -0.34885 0.27477<br>H -6.13595 -2.68504 0.63430<br>H -4.46974 -4.53105 0.70521<br>H -2.05865 -4.03307 0.42597<br>C 0.34191 0.45005 -0.62853<br>S -0.87880 0.80593 -1.92563<br>C -1.71966 2.14761 -1.15953<br>C -1.03672 2.95779 -0.23032<br>N 0.31982 2.83479 0.03980<br>C 0.98513 1.75701 -0.17684<br>C -3.04313 2.43254 -1.50573<br>C -3.70827 3.49657 -0.90337<br>C -3.05506 4.28072 0.05105<br>C -1.72913 4.01993 0.37010<br>C 2.45294 1.81434 0.02532<br>C 3.34485 1.04188 -0.73049<br>C 4.72053 1.16893 -0.54915<br>C 5.22548 2.05274 0.40195<br>C 4.34465 2.82163 1.16580<br>C 2.97329 2.71031 0.97315<br>H -3.55460 1.81071 -2.23570<br>H -4.73836 3.70746 -1.17479	-2133.48803	-2133.04595	-2133.13227	-2133.50998	-2133.15422	-2134.74376	-2134.38799	-2135.14134	-2134.78558	0.0

H -3.57569 5.10416 0.53047<br>H -1.19284 4.63755 1.08484<br>H 2.98337 0.35325 -1.48804<br>H 5.39709 0.57273 -1.15452<br>H 6.29800 2.14243 0.54866<br>H 4.72933 3.50853 1.91430<br>H 2.28350 3.30731 1.56076<br>O -0.98123 0.40497 1.32302<br>O -1.38921 -0.25019 2.52207<br>H -2.26909 -0.58883 2.27551<br>H 1.08438 -0.16166 -1.14588										
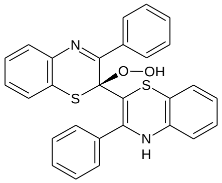<br>S 2.12270 0.83976 -1.31425<br>C 0.91239 0.35841 -0.01647<br>C 1.23512 -1.02598 0.58224<br>N 2.39304 -1.57871 0.61580<br>C 4.75228 -1.71755 0.37450<br>C 5.96482 -1.29238 -0.15031<br>C 5.99642 -0.20128 -1.02316<br>C 4.81981 0.46223 -1.35600<br>C 3.60125 0.03586 -0.82041<br>C 3.55172 -1.06566 0.04975<br>C 0.14018 -1.74800 1.28551<br>C 0.15592 -3.15080 1.28729<br>C -0.82578 -3.87273 1.95652<br>C -1.83804 -3.20444 2.64797<br>C -1.85676 -1.81130 2.66168<br>C -0.87977 -1.08498 1.98244<br>H 4.69835 -2.56557 1.05110<br>H 6.88273 -1.80835 0.11409<br>H 6.93888 0.13756 -1.44314<br>H 4.84451 1.31487 -2.02940<br>H 0.94495 -3.66508 0.74767<br>H -0.80366 -4.95880 1.93648<br>H -2.60516 -3.76689 3.17278<br>H -2.63339 -1.28197 3.20667<br>H -0.89793 -0.00179 2.01984<br>C -0.42089 0.40592 -0.72490<br>S -0.66993 -0.96453 -1.84214<br>C -2.37653 -1.28093 -1.48178<br>C -3.20449 -0.17175 -1.27361<br>N -2.63396 1.10568 -1.22560<br>C -1.39269 1.34814 -0.63935	-2133.48525	-2133.04290	-2133.13042	-2133.50845	-2133.15363	-2134.74069	-2134.38586	-2135.13885	-2134.78403	1.0

C -2.90858 -2.56833 -1.48695<br>C -4.28324 -2.75241 -1.33961<br>C -5.11348 -1.64668 -1.15728<br>C -4.57858 -0.36129 -1.10579<br>C -1.30947 2.66716 0.03918<br>C -0.37655 3.62391 -0.37592<br>C -0.34576 4.87984 0.22388<br>C -1.24484 5.19191 1.24392<br>C -2.18407 4.24649 1.65515<br>C -2.22417 2.99329 1.04827<br>H -2.24957 -3.41963 -1.63167<br>H -4.70060 -3.75411 -1.36988<br>H -6.18463 -1.78228 -1.04084<br>H -5.22457 0.49672 -0.93743<br>H 0.32022 3.38222 -1.17310<br>H 0.37888 5.61762 -0.10847<br>H -1.21755 6.17137 1.71277<br>H -2.88690 4.48385 2.44850<br>H -2.95541 2.25676 1.37206<br>O 0.86546 1.30178 1.04654<br>O 2.12615 1.31792 1.70972<br>H 2.55419 2.08484 1.29440<br>H -3.29134 1.86862 -1.14224										
**Single-bond dimer, C3-hydroperoxide**										
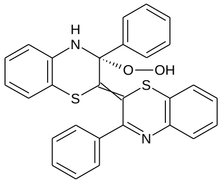<br>S 1.23021 -1.53270 1.42042<br>C 0.94706 -0.04531 0.50268<br>C 2.19551 0.46462 -0.12645<br>N 2.99384 -0.30590 -0.78589<br>C 3.47397 -2.40239 -1.84028<br>C 3.45452 -3.78949 -1.85283<br>C 2.79445 -4.48646 -0.83567<br>C 2.12533 -3.79242 0.16676<br>C 2.11021 -2.39476 0.15911<br>C 2.81087 -1.67996 -0.83469<br>C 2.59138 1.87996 0.05215<br>C 2.22560 2.58345 1.20854<br>C 2.65874 3.89363 1.39979<br>C 3.44339 4.52056 0.43354<br>C 3.80430 3.82735 -0.72480<br>C 3.38993 2.51417 -0.91095<br>H 4.01200 -1.84192 -2.59946<br>H 3.96700 -4.33147 -2.64185	-2133.48673	-2133.04405	-2133.13118	-2133.50926	-2133.15371	-2134.74078	-2134.38523	-2135.13967	-2134.78412	0.0

H 2.79128 -5.57233 -0.82881<br>H 1.59316 -4.33261 0.94503<br>H 1.61132 2.10033 1.96414<br>H 2.37974 4.42498 2.30511<br>H 3.77199 5.54562 0.57932<br>H 4.41058 4.31415 -1.48342<br>H 3.66940 1.96689 -1.80567<br>S -0.45649 2.07910 -0.43734<br>C -0.27173 0.53157 0.41024<br>C -1.48529 -0.06341 1.12446<br>N -2.64179 0.84566 1.09794<br>C -4.41947 1.35097 -0.50786<br>C -4.81377 1.94183 -1.70514<br>C -3.86899 2.52387 -2.55137<br>C -2.52321 2.51827 -2.19198<br>C -2.13168 1.95441 -0.97761<br>C -3.07451 1.36725 -0.12773<br>C -1.88022 -1.44273 0.60716<br>C -2.54528 -2.34555 1.44415<br>C -3.00176 -3.56123 0.93974<br>C -2.81401 -3.88091 -0.40472<br>C -2.16680 -2.97564 -1.24372<br>C -1.70152 -1.76196 -0.74137<br>H -5.15438 0.88476 0.14363<br>H -5.86336 1.93373 -1.98358<br>H -4.17500 2.97173 -3.49165<br>H -1.77530 2.95846 -2.84543<br>H -2.69674 -2.10609 2.49202<br>H -3.50518 -4.26018 1.60158<br>H -3.17154 -4.82900 -0.79601<br>H -2.01837 -3.21287 -2.29327<br>H -1.18934 -1.06894 -1.40264<br>H -3.40568 0.38689 1.58728<br>O -1.19664 -0.23098 2.51101<br>O -0.85700 1.03956 3.06573<br>H -1.62835 1.58028 2.79934										
**Single-bond dimer, dioxolane**										
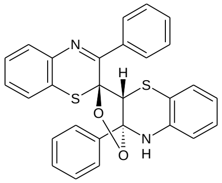<br>S -2.14628 0.25757 0.38775<br>C -0.51503 -0.43767 -0.04791<br>C -0.52544 -1.94088 0.26480<br>N -1.50948 -2.70604 -0.04665<br>C -3.53311 -3.17246 -1.22804	-2133.50577	-2133.06276	-2133.14809	-2133.52715	-2133.16947	-2134.75632	-2134.39863	-2135.15244	-2134.79476	0.0

C -4.77807 -2.79719 -1.71095<br>C -5.22425 -1.48263 -1.53779<br>C -4.41360 -0.54478 -0.90882<br>C -3.14586 -0.91385 -0.44693<br>C -2.70041 -2.24087 -0.58834<br>C 0.64093 -2.59039 0.91946<br>C 1.92542 -2.53416 0.36235<br>C 2.98347 -3.20456 0.97227<br>C 2.77338 -3.92604 2.14708<br>C 1.49642 -3.98752 2.70445<br>C 0.43373 -3.33013 2.08988<br>H -3.17500 -4.19301 -1.32765<br>H -5.40897 -3.52639 -2.20989<br>H -6.20302 -1.18538 -1.90237<br>H -4.75303 0.48015 -0.78776<br>H 2.09658 -1.98942 -0.56268<br>H 3.97244 -3.16479 0.52504<br>H 3.60163 -4.44163 2.62452<br>H 1.32606 -4.55007 3.61787<br>H -0.56433 -3.38062 2.51518<br>S 0.25896 1.39179 2.05970<br>C 0.61778 0.42669 0.57100<br>C 1.13045 1.26584 -0.64628<br>N 1.17083 2.67307 -0.42028<br>C -0.40209 4.54495 -0.35940<br>C -1.41259 5.23649 0.30479<br>C -1.96105 4.72833 1.48247<br>C -1.48995 3.52247 1.99815<br>C -0.45976 2.84341 1.35010<br>C 0.09066 3.34738 0.16422<br>C 2.50009 0.80341 -1.12704<br>C 3.60649 0.97008 -0.28718<br>C 4.86978 0.55668 -0.70038<br>C 5.03948 -0.02416 -1.95830<br>C 3.94021 -0.18484 -2.79837<br>C 2.67236 0.22770 -2.38558<br>H 1.41570 -0.24089 0.89715<br>H 0.01606 4.93612 -1.28354<br>H -1.78015 6.17076 -0.10932<br>H -2.75722 5.26093 1.99331<br>H -1.91332 3.10877 2.90919<br>H 3.47867 1.42933 0.68975<br>H 5.72255 0.69016 -0.04096<br>H 6.02513 -0.34725 -2.28090<br>H 4.06472 -0.63264 -3.78019<br>H 1.81712 0.10043 -3.04044<br>O -0.24172 -0.36128 -1.44119<br>O 0.12029 1.00880 -1.65178<br>H 1.60089 3.16853 -1.19162										

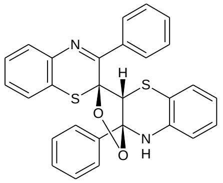<br>S -1.58545 -1.12281 -0.84989<br>C -0.87575 0.14481 0.24430<br>C -1.64872 1.46467 0.12823<br>N -2.91160 1.55713 -0.08660<br>C -5.13174 0.70539 -0.23743<br>C -6.03913 -0.31122 -0.49649<br>C -5.57627 -1.58007 -0.86000<br>C -4.21145 -1.83003 -0.94466<br>C -3.29792 -0.80729 -0.67106<br>C -3.74925 0.47571 -0.32225<br>C -0.92941 2.74747 0.36099<br>C -0.14970 2.96306 1.50627<br>C 0.45669 4.19927 1.71809<br>C 0.30274 5.22672 0.78778<br>C -0.47181 5.01781 -0.35308<br>C -1.09052 3.78810 -0.56186<br>H -5.46462 1.70260 0.03532<br>H -7.10513 -0.11921 -0.42355<br>H -6.28050 -2.37919 -1.07109<br>H -3.84969 -2.81822 -1.21511<br>H -0.03591 2.17456 2.24495<br>H 1.04755 4.35949 2.61531<br>H 0.78236 6.18720 0.95295<br>H -0.59646 5.81386 -1.08151<br>H -1.70105 3.62283 -1.44461<br>S 1.28800 0.06776 -1.65893<br>C 0.62508 0.21794 -0.00191<br>C 1.19348 -0.79407 1.00604<br>N 2.55362 -0.42573 1.24484<br>C 4.75314 0.18002 0.51104<br>C 5.66747 0.59346 -0.44658<br>C 5.24459 0.86599 -1.74875<br>C 3.90082 0.71958 -2.06688<br>C 2.97100 0.28796 -1.11378<br>C 3.39335 0.00741 0.20406<br>C 1.02303 -2.28947 0.76518<br>C 0.15648 -3.04953 1.55685<br>C 0.04373 -4.42458 1.36328<br>C 0.79244 -5.05910 0.37373<br>C 1.67081 -4.31086 -0.40852<br>C 1.79296 -2.93734 -0.20868<br>H 0.96231 1.18660 0.37432<br>H 5.08298 -0.02069 1.52744	-2133.48868	-2133.04559	-2133.13062	-2133.50954	-2133.15148	-2134.73847	-2134.38041	-2135.13492	-2134.77686	11.2

H 6.71130 0.71066 -0.17121<br>H 5.94896 1.19821 -2.50460<br>H 3.55324 0.93937 -3.07302<br>H -0.43463 -2.56314 2.32427<br>H -0.63386 -4.99958 1.98812<br>H 0.70116 -6.13066 0.22023<br>H 2.27222 -4.79620 -1.17187<br>H 2.50201 -2.37802 -0.80972<br>O -0.93583 -0.24341 1.63966<br>O 0.42101 -0.37786 2.14871<br>H 3.02467 -1.03015 1.90643										
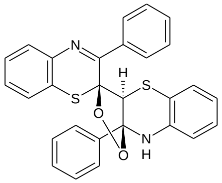<br>S -1.21022 -1.70238 -1.64914<br>C -0.02856 -0.58147 -0.76893<br>C 1.14602 -1.46407 -0.34584<br>N 0.93043 -2.53126 0.33705<br>C -0.51957 -3.91622 1.65217<br>C -1.72918 -4.56873 1.84442<br>C -2.77839 -4.37784 0.93994<br>C -2.62588 -3.50554 -0.13258<br>C -1.42162 -2.81850 -0.30953<br>C -0.34048 -3.03638 0.57278<br>C 2.55662 -1.12943 -0.65839<br>C 3.53879 -1.48310 0.28052<br>C 4.88440 -1.25511 0.01996<br>C 5.27620 -0.68120 -1.19064<br>C 4.31004 -0.33785 -2.13404<br>C 2.95861 -0.55326 -1.87182<br>H 0.31809 -4.07918 2.32401<br>H -1.85294 -5.23977 2.68898<br>H -3.72110 -4.89879 1.07736<br>H -3.44681 -3.33753 -0.82378<br>H 3.22826 -1.93152 1.21863<br>H 5.62940 -1.52309 0.76377<br>H 6.32805 -0.50499 -1.39704<br>H 4.60602 0.09681 -3.08443<br>H 2.22569 -0.29446 -2.62664<br>S 0.17297 0.43826 1.91995<br>C -0.74353 0.20135 0.37434<br>C -1.14274 1.53518 -0.33622<br>N -0.43003 2.67845 0.15166<br>C 1.80305 3.65818 0.15328<br>C 3.10429 3.66492 0.64901	-<br>2133.50482	-<br>2133.06177	-<br>2133.14682	-<br>2133.52549	-<br>2133.16748	-<br>2134.75598	-<br>2134.39797	-<br>2135.15202	-<br>2134.79402	0.5

C 3.54217 2.65361 1.50473<br>C 2.66750 1.62987 1.86437<br>C 1.35672 1.63502 1.39171<br>C 0.91162 2.64910 0.53187<br>C -2.63929 1.81359 -0.30409<br>C -3.50218 1.22712 -1.23589<br>C -4.87296 1.46679 -1.16865<br>C -5.39761 2.28273 -0.16697<br>C -4.54136 2.86148 0.76765<br>C -3.16812 2.63158 0.69874<br>H -1.64232 -0.33468 0.68296<br>H 1.46671 4.44397 -0.51869<br>H 3.78232 4.46065 0.35423<br>H 4.56016 2.65375 1.88194<br>H 2.99643 0.82752 2.51850<br>H -3.10170 0.58339 -2.01096<br>H -5.53195 1.01256 -1.90308<br>H -6.46706 2.46589 -0.11566<br>H -4.93926 3.49661 1.55387<br>H -2.50220 3.08564 1.42529<br>O 0.34776 0.42570 -1.66758<br>O -0.82154 1.26105 -1.72260<br>H -0.67991 3.50807 -0.37346										
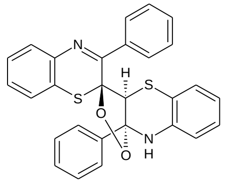<br>S -0.61287 -2.33491 -1.99456<br>C 0.20832 -0.97440 -1.07180<br>C 1.00714 -1.61968 0.06238<br>N 0.45084 -2.45118 0.86889<br>C -1.46581 -3.58206 1.75245<br>C -2.66796 -4.25458 1.58525<br>C -3.23024 -4.37083 0.31063<br>C -2.60488 -3.78583 -0.78573<br>C -1.41400 -3.07546 -0.61396<br>C -0.81718 -2.98450 0.66116<br>C 2.43373 -1.28952 0.30116<br>C 2.84948 -1.02222 1.61224<br>C 4.19386 -0.79909 1.89397<br>C 5.14253 -0.86353 0.87327<br>C 4.73657 -1.14113 -0.43169<br>C 3.38960 -1.34198 -0.72188<br>H -0.99553 -3.50776 2.72862<br>H -3.16096 -4.70350 2.44211<br>H -4.16288 -4.90901 0.17078	-2133.48279	-2133.03970	-2133.12349	-2133.50415	-2133.14485	-2134.73721	-2134.37791	-2135.13376	-2134.77446	12.7

H -3.05079 -3.85872 -1.77360<br>H 2.10801 -0.98551 2.40456<br>H 4.50108 -0.57888 2.91239<br>H 6.19335 -0.69864 1.09388<br>H 5.47095 -1.20293 -1.22969<br>H 3.08957 -1.56415 -1.74042<br>S -1.45624 0.26397 1.00695<br>C -0.78055 0.13113 -0.65117<br>C -0.08942 1.41612 -1.14085<br>N -1.12840 2.36260 -1.40557<br>C -3.04047 3.62763 -0.70381<br>C -4.11517 3.88364 0.13465<br>C -4.38577 3.03134 1.20666<br>C -3.56858 1.92861 1.41597<br>C -2.47063 1.66751 0.58686<br>C -2.19178 2.52766 -0.49837<br>C 1.07259 2.03114 -0.36514<br>C 0.84913 2.65845 0.86820<br>C 1.88972 3.29832 1.53791<br>C 3.16528 3.34127 0.97859<br>C 3.38973 2.74115 -0.25850<br>C 2.35379 2.08775 -0.92294<br>H -1.64026 0.02384 -1.31936<br>H -2.83657 4.28782 -1.54335<br>H -4.74552 4.74729 -0.05483<br>H -5.22786 3.21637 1.86586<br>H -3.77323 1.24924 2.23947<br>H -0.14031 2.66786 1.31038<br>H 1.69574 3.77427 2.49499<br>H 3.97472 3.84621 1.49827<br>H 4.37671 2.77509 -0.71117<br>H 2.54427 1.62075 -1.88189<br>O 1.05217 -0.31060 -2.00347<br>O 0.36956 0.89917 -2.40679<br>H -0.77992 3.24541 -1.75941										
**Double-bond dimers**										
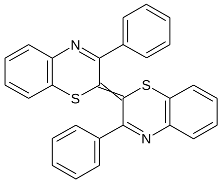<br>C -0.99661 0.28580 4.29094<br>C -0.79523 0.76007 2.99251<br>C -0.06062 1.94340 2.76574<br>C 0.50282 2.60166 3.87113<br>C 0.33242 2.10992 5.15730<br>C -0.43419 0.95991 5.36989	-<br>1982.06926	-<br>1981.66097	-<br>1981.74341	-<br>1982.08734	-<br>1981.76149	-<br>1983.21692	-<br>1982.89108	-<br>1983.56655	-<br>1983.24070	0.0

N 0.01510 2.56088 1.52588<br>C -0.15936 1.91126 0.42329<br>C -0.32068 0.43393 0.40949<br>S -1.48612 -0.10834 1.62205<br>C 0.32068 -0.43393 -0.40949<br>C 0.15936 -1.91126 -0.42329<br>N -0.01510 -2.56088 -1.52588<br>C 0.06062 -1.94340 -2.76574<br>C 0.79523 -0.76007 -2.99251<br>S 1.48612 0.10834 -1.62205<br>C 0.99661 -0.28580 -4.29094<br>C 0.43419 -0.95991 -5.36989<br>C -0.33242 -2.10992 -5.15730<br>C -0.50282 -2.60166 -3.87113<br>C 0.27665 -2.69689 0.82766<br>C -0.27665 2.69689 -0.82766<br>H 1.06536 3.51284 3.68915<br>H 0.78052 2.62952 5.99879<br>H -0.58624 0.58122 6.37615<br>H -1.57356 -0.62066 4.45109<br>H -1.06536 -3.51284 -3.68915<br>H -0.78052 -2.62952 -5.99879<br>H 0.58624 -0.58122 -6.37615<br>H 1.57356 0.62066 -4.45109<br>C 1.09922 -2.26014 1.87392<br>C 1.26266 -3.04220 3.01357<br>C 0.59567 -4.26190 3.12830<br>C -0.22894 -4.70075 2.09086<br>C -0.38115 -3.92846 0.94395<br>H 1.62132 -1.31090 1.79134<br>H 1.91192 -2.69784 3.81342<br>H 0.71739 -4.86778 4.02166<br>H -0.75509 -5.64727 2.17662<br>H -1.01714 -4.26493 0.13126<br>C 0.38115 3.92846 -0.94395<br>C 0.22894 4.70075 -2.09086<br>C -0.59567 4.26190 -3.12830<br>C -1.26266 3.04220 -3.01357<br>C -1.09922 2.26014 -1.87392<br>H 1.01714 4.26493 -0.13126<br>H 0.75509 5.64727 -2.17662<br>H -0.71739 4.86778 -4.02166<br>H -1.91192 2.69784 -3.81342<br>H -1.62132 1.31090 -1.79134										
**Oxygen species**										
O_2<br>O 0.00000 0.00000 0.60130<br>O 0.00000 0.00000 -0.60130	-150.17445	-150.16722	-150.19049	-150.17424	-150.19028	-150.26380	-150.27984	-150.31351	-150.32955	0.0
H_2O_2<br>O 0.00000 0.71450 -0.06520	-151.40433	-151.37324	-151.39898	-151.41318	-151.40783	-151.50645	-151.50109	-151.55778	-151.55243	0.0

O 0.00000 -0.71450 -0.06520<br>H -0.74458 -0.91370 0.52162<br>H 0.74458 0.91370 0.52162										
H_2O<br>O 0.00000 0.00000 0.11750<br>H 0.00000 0.76275 -0.47000<br>H 0.00000 -0.76275 -0.47000	-76.35719	-76.33188	-76.35331	-76.36339	-76.35950					0.0
HOO·<br>O 0.05522 0.71139 0.00000<br>O 0.05522 -0.60193 0.00000<br>H -0.88355 -0.87569 0.00000	-150.76380	-150.74551	-150.77146	-150.76970	-150.77736	-150.85860	-150.86626	-150.90856	-150.91622	0.0
HO·<br>O 0.00000 0.00000 0.10848<br>H 0.00000 0.00000 -0.86783	-75.66227	-75.65038	-75.67062	-75.66470	-75.67305					0.0

Table S5. Monoprotonated forms in methanol. For each chemical species, values are reported for the most stable conformer / geometric isomer identified. $G_{RRHO,calc}$ [M062X / 6-311++G(2d,2p) / SMD] = G_{el} [M062X / 6-311++G(2d,2p) / SMD // PBE0 / 6-31+G(d,p) / PCM] - G_{el} [PBE0 / 6-31+G(d,p) / PCM] + G_{RRHO} [PBE0 / 6-31+G(d,p) / PCM]; and likewise for $G_{RRHO,calc}$ [PBE0 / 6-31+G(d,p) / SMD] and for $G_{RRHO,calc}$ [M062X / 6-31+G(d,p) / SMD].

Species	PBE0 / 6-31+G(d,p) / PCM			PBE0 / 6-31+G(d,p) / SMD		M062X / 6-31+G(d,p) / SMD		M062X / 6-311++G(2d,2p) / SMD		
**Cartesian coordinates / Å**	**G_{el} / Ha**	**H_{RRHO} / Ha**	**G_{RRHO} / Ha**	**G_{el} / Ha**	**$G_{RRHO,calc}$ / Ha**	**G_{el} / Ha**	**$G_{RRHO,calc}$ / Ha**	**G_{el} / Ha**	**$G_{RRHO,calc}$ / Ha**	**$\Delta G_{RRHO,calc}$ / kcal mol^{-1}**
**Single-bond dimer**										
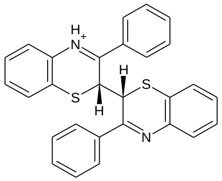<br>S -0.95488 -2.39940 1.36073<br>C -0.07592 -0.94076 0.73764<br>C -0.74463 0.28944 1.27772<br>N -2.04887 0.37814 1.16827<br>C -4.21625 -0.24540 0.30801<br>C -5.12609 -1.22866 -0.05235<br>C -4.75783 -2.57519 0.00360<br>C -3.47862 -2.94394 0.40636<br>C -2.54964 -1.96598 0.76725<br>C -2.93388 -0.61683 0.71892<br>C 0.00878 1.40257 1.83750<br>C -0.48202 2.72003 1.74389<br>C 0.23067 3.77145 2.29882<br>C 1.43572 3.52827 2.96121<br>C 1.93178 2.22916 3.05737<br>C 1.23283 1.17133 2.49078<br>H 0.92981 -1.01639 1.15439<br>H -4.48875 0.80549 0.27112<br>H -6.12176 -0.94600 -0.37699<br>H -5.46992 -3.34454 -0.27726<br>H -3.19265 -3.99044 0.43822<br>H -1.38966 2.94149 1.18943<br>H -0.14569 4.78459 2.20245<br>H 1.99026 4.35397 3.39632<br>H 2.86496 2.03668 3.57650<br>H 1.62518 0.16641 2.60304<br>S 0.86296 -2.36672 -1.47666<br>C 0.04866 -0.89393 -0.81877<br>C 0.82305 0.33315 -1.27668<br>N 2.10253 0.41265 -1.15718<br>C 4.16894 -0.39162 -0.25717	-1983.73473	-1983.28790	-1983.37200	-1983.76701	-1983.40428	-1984.89083	-1984.52810	-1985.24033	-1984.87760	0.0

C 5.03805 -1.42273 0.06968<br>C 4.64103 -2.75209 -0.10361<br>C 3.36406 -3.04418 -0.57125<br>C 2.47633 -2.00856 -0.87488<br>C 2.87789 -0.66172 -0.73977<br>C 0.09178 1.50240 -1.81499<br>C 0.73058 2.75462 -1.83650<br>C 0.08006 3.87481 -2.33510<br>C -1.22227 3.76983 -2.83030<br>C -1.86395 2.53414 -2.82243<br>C -1.21548 1.40951 -2.31562<br>H -0.95115 -0.91453 -1.25365<br>H 4.46824 0.64767 -0.15722<br>H 6.03086 -1.19471 0.44529<br>H 5.32301 -3.56265 0.13460<br>H 3.04597 -4.07632 -0.68824<br>H 1.74010 2.83049 -1.44672<br>H 0.58655 4.83565 -2.33469<br>H -1.73080 4.64661 -3.22048<br>H -2.87155 2.43830 -3.21574<br>H -1.73910 0.45945 -2.35117<br>H -2.49067 1.22649 1.51219										
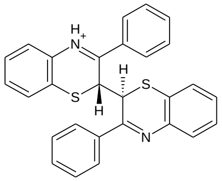<br>S 1.01274 1.00361 1.87295<br>C -0.23174 0.46466 0.66712<br>C -0.96771 1.69378 0.15153<br>N -0.34121 2.73491 -0.26948<br>C 1.67711 3.84334 -0.92896<br>C 3.01630 4.15116 -0.73746<br>C 3.73697 3.52611 0.28469<br>C 3.12465 2.56603 1.08301<br>C 1.78496 2.22692 0.86919<br>C 1.03351 2.88590 -0.12798<br>C -2.44636 1.69751 0.10235<br>C -3.09625 2.51660 -0.83582<br>C -4.48298 2.56583 -0.89177<br>C -5.24947 1.81039 -0.00091<br>C -4.61581 1.00321 0.94045<br>C -3.22469 0.93766 0.98659<br>H -0.90717 -0.17298 1.23911<br>H 1.09013 4.34519 -1.69268<br>H 3.49682 4.89169 -1.36941<br>H 4.77887 3.77962 0.45505<br>H 3.68781 2.06473 1.86559	-1983.73492	-1983.28791	-1983.37071	-1983.76576	-1983.40155	-1984.89051	-1984.52630	-1985.24064	-1984.87643	0.7

H -2.49507 3.10258 -1.52330<br>H -4.96983 3.19365 -1.63247<br>H -6.33401 1.85232 -0.04223<br>H -5.20253 0.41974 1.64377<br>H -2.76185 0.31247 1.74416<br>S -0.83164 -0.85422 -1.75648<br>C 0.38390 -0.34908 -0.49636<br>C 1.04255 -1.63175 -0.06598<br>N 0.25791 -2.56448 0.43428<br>C -1.85851 -3.51580 1.11681<br>C -3.21167 -3.70550 0.88163<br>C -3.84057 -3.03317 -0.17042<br>C -3.12781 -2.15009 -0.97297<br>C -1.76757 -1.93724 -0.73968<br>C -1.13658 -2.64352 0.29757<br>C 2.46431 -1.89208 -0.20014<br>C 2.94018 -3.21693 -0.28480<br>C 4.29970 -3.46602 -0.38970<br>C 5.20498 -2.40309 -0.41801<br>C 4.74456 -1.08804 -0.35554<br>C 3.38456 -0.82957 -0.25679<br>H 1.10129 0.26356 -1.04552<br>H -1.35415 -4.04076 1.92275<br>H -3.77698 -4.38111 1.51463<br>H -4.89883 -3.18606 -0.35648<br>H -3.62481 -1.61045 -1.77284<br>H 2.25182 -4.05598 -0.32355<br>H 4.65428 -4.48849 -0.46782<br>H 6.26905 -2.60123 -0.50329<br>H 5.44638 -0.26087 -0.38381<br>H 3.04873 0.19925 -0.19339<br>H 0.70234 -3.37353 0.85861										
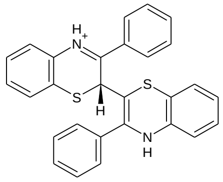<br>S -1.57116 -1.58004 -1.81520<br>C -0.38552 -0.98569 -0.53501<br>C -1.02846 -1.15763 0.81498<br>N -2.26553 -0.75438 0.99385<br>C -4.36371 0.28974 0.43861<br>C -5.32894 0.62760 -0.49751<br>C -5.13405 0.31247 -1.84550<br>C -3.97391 -0.33179 -2.25885<br>C -2.99222 -0.67913 -1.32658<br>C -3.20106 -0.36536 0.02389<br>C -0.30466 -1.72708 1.94320	-<br>1983.73393	-<br>1983.28656	-<br>1983.37001	-<br>1983.76499	-<br>1983.40107	-<br>1984.88643	-<br>1984.52250	-<br>1985.23695	-<br>1984.87302	2.9

C -1.00005 -2.37185 2.98386<br>C -0.30801 -2.90173 4.06408<br>C 1.08210 -2.79856 4.12287<br>C 1.78155 -2.17339 3.08982<br>C 1.09796 -1.64624 2.00326<br>H 0.42103 -1.71784 -0.60530<br>H -4.50167 0.53195 1.48878<br>H -6.23197 1.13716 -0.17863<br>H -5.88777 0.57743 -2.58014<br>H -3.82002 -0.56755 -3.30726<br>H -2.07579 -2.51274 2.93053<br>H -0.85273 -3.41042 4.85282<br>H 1.62097 -3.21439 4.96871<br>H 2.86290 -2.09257 3.13309<br>H 1.65315 -1.14315 1.21896<br>S -0.82887 1.81590 -0.50748<br>C 0.19931 0.37902 -0.73248<br>C 1.50943 0.56879 -1.07523<br>N 2.06985 1.82801 -1.03044<br>C 2.60725 3.70708 0.42002<br>C 2.19714 4.66001 1.34962<br>C 0.87347 4.69786 1.78597<br>C -0.05062 3.78598 1.27621<br>C 0.34616 2.86159 0.31294<br>C 1.67954 2.80564 -0.10594<br>C 2.43795 -0.51097 -1.48840<br>C 3.68484 -0.64601 -0.86026<br>C 4.56753 -1.64529 -1.26047<br>C 4.22197 -2.50931 -2.29956<br>C 2.99140 -2.37015 -2.94023<br>C 2.10304 -1.37601 -2.53869<br>H 3.64480 3.66034 0.09966<br>H 2.92178 5.36688 1.74214<br>H 0.55699 5.43521 2.51700<br>H -1.08611 3.80787 1.60329<br>H 3.95576 0.02029 -0.04504<br>H 5.52502 -1.74989 -0.75903<br>H 4.91357 -3.28561 -2.61331<br>H 2.72528 -3.02946 -3.76094<br>H 1.15747 -1.24982 -3.05897<br>H 3.02105 1.89430 -1.36538<br>H -2.60996 -0.74449 1.95027										
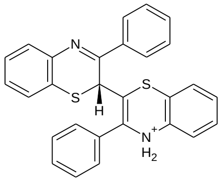	n.d.									

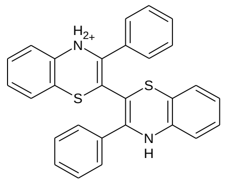	n.d.									
**Single-bond dimer, radical**										
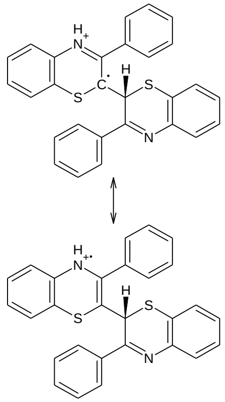<br>S 0.68112 1.46708 -0.71613<br>C -0.35077 0.39636 0.16684<br>C -1.68834 0.66978 0.40547<br>N -2.28857 1.79666 -0.04652<br>C -2.52079 3.91963 -1.14170<br>C -1.96143 4.96664 -1.85190<br>C -0.60403 4.94429 -2.19959<br>C 0.19187 3.86909 -1.83752<br>C -0.36335 2.80396 -1.12010<br>C -1.72415 2.82919 -0.76793<br>C -2.59624 -0.25865 1.12394<br>C -3.68868 -0.80466 0.43602<br>C -4.56802 -1.65827 1.09691<br>C -4.36984 -1.96000 2.44328<br>C -3.29120 -1.40619 3.13223<br>C -2.40278 -0.55764 2.47788<br>H -3.57220 3.93090 -0.87032<br>H -2.58181 5.80898 -2.13910<br>H -0.17066 5.76856 -2.75588<br>H 1.24301 3.84624 -2.10816<br>H -3.83880 -0.57769 -0.61627<br>H -5.40589 -2.08818 0.55688<br>H -5.05781 -2.62455 2.95712<br>H -3.14196 -1.62968 4.18402<br>H -1.57500 -0.11156 3.02083<br>S 1.14911 -0.68734 2.25571	-<br>1983.11486	-<br>1982.68051	-<br>1982.76494	-<br>1983.14295	-<br>1982.79303	-<br>1984.26261	-<br>1983.91269	-<br>1984.61340	-<br>1984.26348	4.8

C 0.29782 -0.87506 0.64538<br>C 1.25956 -1.51750 -0.35773<br>N 2.50496 -1.20900 -0.44476<br>C 4.36550 0.21888 -0.00421<br>C 5.09219 1.06242 0.82432<br>C 4.61149 1.37037 2.10042<br>C 3.39489 0.85316 2.53314<br>C 2.65315 0.01739 1.69372<br>C 3.13981 -0.32153 0.41422<br>C 0.71846 -2.53376 -1.28584<br>C -0.65959 -2.73863 -1.44823<br>C -1.13477 -3.68822 -2.34999<br>C -0.24229 -4.45514 -3.09493<br>C 1.13275 -4.26364 -2.93764<br>C 1.60820 -3.31192 -2.04592<br>H -0.49557 -1.58731 0.87707<br>H 4.72821 -0.04761 -0.99255<br>H 6.03757 1.47331 0.48373<br>H 5.18107 2.02036 2.75765<br>H 3.01331 1.10101 3.51954<br>H -1.38355 -2.15559 -0.88777<br>H -2.20540 -3.82722 -2.46638<br>H -0.61367 -5.20011 -3.79257<br>H 1.83469 -4.86168 -3.51144<br>H 2.67441 -3.15706 -1.91932<br>H -3.26497 1.90717 0.20545										
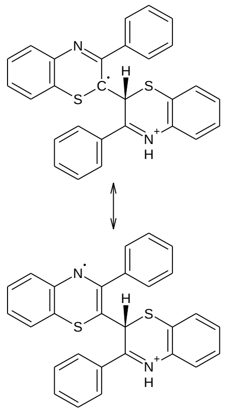<br>S -1.22412 1.42628 0.06639<br>C 0.12237 0.47012 -0.46520<br>C 1.40369 1.03544 -0.65451<br>N 1.77180 2.26308 -0.31815<br>C 1.44615 4.41062 0.63504<br>C 0.65034 5.39156 1.19761<br>C -0.71757 5.15890 1.39841<br>C -1.27606 3.93983 1.04130	-<br>1983.10335	-<br>1982.66948	-<br>1982.75370	-<br>1983.13332	-<br>1982.78368	-<br>1984.25136	-<br>1983.90171	-<br>1984.60140	-<br>1984.25175	12.2

C -0.46861 2.94486 0.48119										
C 0.91037 3.16062 0.25731										
C 2.49169 0.22223 -1.27517										
C 3.70140 0.05042 -0.59334										
C 4.73870 -0.67350 -1.17570										
C 4.58330 -1.21998 -2.45007										
C 3.38665 -1.03780 -3.14163										
C 2.34336 -0.32250 -2.55625										
H 2.50585 4.57224 0.46268										
H 1.08514 6.34538 1.47984										
H -1.34552 5.92901 1.83514										
H -2.33476 3.75382 1.19938										
H 3.82172 0.48465 0.39490										
H 5.67063 -0.80929 -0.63448										
H 5.39462 -1.78101 -2.90469										
H 3.26478 -1.44626 -4.14054										
H 1.41983 -0.16558 -3.10863										
S -1.22353 -1.46182 -2.08244										
C -0.14188 -0.99628 -0.67446										
C -0.61333 -1.63759 0.60066										
N -1.89602 -1.72046 0.85284										
C -4.23271 -1.18969 0.61476										
C -5.31738 -0.85075 -0.18055										
C -5.14287 -0.66321 -1.55403										
C -3.88688 -0.80720 -2.13281										
C -2.78558 -1.15393 -1.34579										
C -2.97299 -1.34343 0.03085										
C 0.34220 -2.06626 1.61594										
C 1.57060 -2.63900 1.24308										
C 2.46086 -3.07018 2.21798										
C 2.15170 -2.91541 3.56893										
C 0.94285 -2.32994 3.94805										
C 0.03929 -1.91111 2.98195										
H 0.81491 -1.45128 -0.93489										
H -4.35347 -1.32752 1.68559										
H -6.29653 -0.72877 0.26998										
H -5.98979 -0.39586 -2.17788										
H -3.75239 -0.65398 -3.19902										
H 1.82014 -2.79169 0.19826										
H 3.39782 -3.52991 1.92065										
H 2.85552 -3.24452 4.32718										
H 0.70953 -2.18721 4.99818										
H -0.87415 -1.41297 3.29525										
H -2.15990 -2.13016 1.74586										

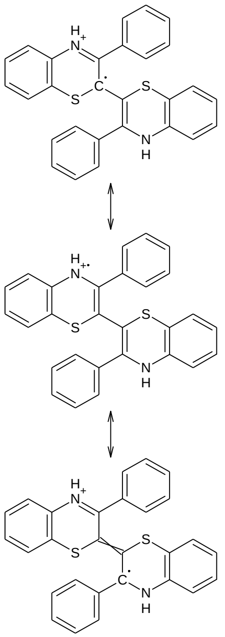<br>S 0.57917 1.40250 -2.45516<br>C 0.14075 0.69588 -0.87902<br>C -0.04302 1.56078 0.20821<br>N 0.49387 2.79990 0.20989<br>C 2.22544 4.40791 -0.29719<br>C 3.17055 4.91918 -1.17922<br>C 3.36151 4.32332 -2.42609<br>C 2.59967 3.21758 -2.79617<br>C 1.63562 2.71451 -1.92403<br>C 1.45448 3.30414 -0.66907<br>C -0.88734 1.25510 1.37990<br>C -2.13536 0.63679 1.21075<br>C -2.96561 0.43058 2.30619<br>C -2.56471 0.83907 3.57942<br>C -1.32879 1.45954 3.75299<br>C -0.49387 1.67052 2.66013<br>H 2.08204 4.85885 0.68068<br>H 3.76321 5.78017 -0.88755<br>H 4.10405 4.71627 -3.11311<br>H 2.74333 2.75070 -3.76584<br>H -2.46252 0.34143 0.21865<br>H -3.93467 -0.03807 2.16371<br>H -3.21633 0.67615 4.43272<br>H -1.00761 1.77190 4.74198<br>H 0.48184 2.12471 2.81248	-<br>1983.12175	-<br>1982.68722	-<br>1982.77091	-<br>1983.15053	-<br>1982.79970	-<br>1984.27181	-<br>1983.92097	-<br>1984.62204	-<br>1984.27120	0.0

S -0.57917 -1.40250 -2.45516<br>C -0.14075 -0.69588 -0.87902<br>C 0.04302 -1.56078 0.20821<br>N -0.49387 -2.79990 0.20989<br>C -2.22544 -4.40791 -0.29719<br>C -3.17055 -4.91918 -1.17922<br>C -3.36151 -4.32332 -2.42609<br>C -2.59967 -3.21758 -2.79617<br>C -1.63562 -2.71451 -1.92403<br>C -1.45448 -3.30414 -0.66907<br>C 0.88734 -1.25510 1.37990<br>C 2.13536 -0.63679 1.21075<br>C 2.96561 -0.43058 2.30619<br>C 2.56471 -0.83907 3.57942<br>C 1.32879 -1.45954 3.75299<br>C 0.49387 -1.67052 2.66013<br>H -2.08204 -4.85885 0.68068<br>H -3.76321 -5.78017 -0.88755<br>H -4.10405 -4.71627 -3.11311<br>H -2.74333 -2.75070 -3.76584<br>H 2.46252 -0.34143 0.21865<br>H 3.93467 0.03807 2.16371<br>H 3.21633 -0.67615 4.43272<br>H 1.00761 -1.77190 4.74198<br>H -0.48184 -2.12471 2.81248<br>H -0.21418 -3.40858 0.96883<br>H 0.21418 3.40858 0.96883										

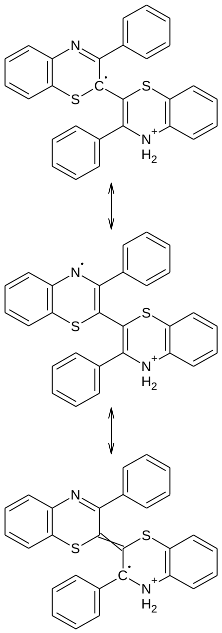	n.d.									
**Single-bond dimer, C2-peroxyl radical**										
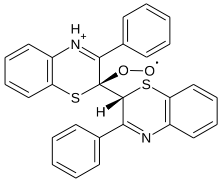<br>S 0.56412 -1.64020 -0.32311<br>C 0.35550 0.14411 -0.42588<br>C 1.69448 0.83128 -0.17399<br>N 2.73921 0.36707 -0.81219<br>C 3.95978 -0.94880 -2.42173<br>C 4.12136 -2.13779 -3.11299<br>C 3.19093 -3.17079 -2.95700<br>C 2.08919 -3.01647 -2.12429<br>C 1.90771 -1.81673 -1.43407<br>C 2.85374 -0.79546 -1.57939<br>C 1.92469 1.95798 0.73228<br>C 2.95621 1.84659 1.68086<br>C 3.23024 2.90834 2.53368<br>C 2.49801 4.09075 2.43404<br>C 1.49063 4.21386 1.47631<br>C 1.19406 3.15294 0.63014<br>H 4.67507 -0.13859 -2.52827	-2133.27400	-2132.83028	-2132.91766	-2133.30523	-2132.94888	-2134.52505	-2134.16870	-2134.92199	-2134.56564	2.7

H 4.97252 -2.26430 -3.77306<br>H 3.32005 -4.10199 -3.49902<br>H 1.36309 -3.81594 -2.01746<br>H 3.50411 0.91406 1.78239<br>H 4.01181 2.80878 3.27999<br>H 2.71513 4.92010 3.10010<br>H 0.93862 5.14326 1.38040<br>H 0.44968 3.27582 -0.14893<br>S -0.51371 0.46052 2.25685<br>C -0.80871 0.62542 0.47735<br>C -2.16066 0.01265 0.09928<br>N -2.62351 -1.07001 0.60934<br>C -2.37443 -3.10558 1.83354<br>C -1.83165 -3.85120 2.87119<br>C -0.89664 -3.26970 3.73154<br>C -0.48605 -1.95510 3.53246<br>C -1.01477 -1.21233 2.47498<br>C -1.97994 -1.77689 1.61922<br>C -2.98200 0.70049 -0.92412<br>C -3.79881 -0.07187 -1.76469<br>C -4.59511 0.53429 -2.72894<br>C -4.60084 1.92380 -2.86224<br>C -3.80282 2.70072 -2.02475<br>C -2.99260 2.09467 -1.06778<br>H -0.86869 1.70229 0.31603<br>H -3.11817 -3.52986 1.16555<br>H -2.14452 -4.88001 3.01996<br>H -0.47934 -3.84204 4.55438<br>H 0.25336 -1.50672 4.19003<br>H -3.78811 -1.15162 -1.65704<br>H -5.21011 -0.07851 -3.38155<br>H -5.22419 2.39768 -3.61480<br>H -3.81095 3.78308 -2.11139<br>H -2.40426 2.73066 -0.41315<br>O -0.02426 0.42062 -1.83250<br>O 0.12247 1.68697 -2.11746<br>H 3.59380 0.91345 -0.71454										
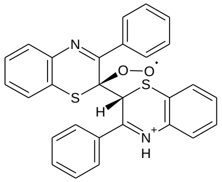<br>S 0.61111 -1.25397 -1.03444<br>C 0.22209 0.41968 -0.52035<br>C 1.44124 1.25833 -0.11345<br>N 2.64940 1.01579 -0.46426<br>C 4.41334 -0.04387 -1.65385	-<br>2133.27668	-<br>2132.83336	-<br>2132.92275	-<br>2133.30793	-<br>2132.95400	-<br>2134.52676	-<br>2134.17283	-<br>2134.92358	-<br>2134.56965	0.2

C 4.92802 -1.08146 -2.41715<br>C 4.10873 -2.15917 -2.76456<br>C 2.77909 -2.19276 -2.35679<br>C 2.26577 -1.14286 -1.59337<br>C 3.07600 -0.05834 -1.22848<br>C 1.22325 2.48967 0.69617<br>C 2.10096 2.75022 1.75811<br>C 1.97184 3.91032 2.51306<br>C 0.97055 4.83480 2.21172<br>C 0.10325 4.59213 1.14875<br>C 0.22412 3.42577 0.39509<br>H 5.02605 0.80571 -1.36776<br>H 5.96354 -1.05610 -2.74116<br>H 4.50335 -2.97634 -3.36056<br>H 2.14228 -3.02824 -2.63364<br>H 2.87762 2.02747 1.98797<br>H 2.65246 4.09297 3.33948<br>H 0.87052 5.74193 2.80043<br>H -0.66603 5.31525 0.89472<br>H -0.43512 3.27498 -0.45442<br>S -0.26303 0.03206 2.25262<br>C -0.88374 0.38260 0.58146<br>C -2.06209 -0.48615 0.22218<br>N -2.12794 -1.72026 0.65021<br>C -1.34026 -3.80971 1.54942<br>C -0.49734 -4.51313 2.39882<br>C 0.43895 -3.82664 3.17384<br>C 0.54277 -2.44020 3.09909<br>C -0.29879 -1.72275 2.24985<br>C -1.23651 -2.42001 1.48112<br>C -3.12733 0.00771 -0.64207<br>C -3.72355 -0.85827 -1.57835<br>C -4.75155 -0.40146 -2.39015<br>C -5.20605 0.91257 -2.26936<br>C -4.62183 1.77515 -1.34188<br>C -3.57789 1.33503 -0.53946<br>H -1.25407 1.40778 0.65694<br>H -2.07029 -4.32872 0.93486<br>H -0.56991 -5.59384 2.45429<br>H 1.09970 -4.37383 3.83849<br>H 1.27643 -1.91178 3.69977<br>H -3.34714 -1.86833 -1.71622<br>H -5.18952 -1.06695 -3.12680<br>H -6.01268 1.26637 -2.90392<br>H -4.98058 2.79422 -1.24168<br>H -3.15564 2.01309 0.19495<br>O -0.46165 1.16653 -1.62608<br>O 0.22519 1.15439 -2.72922<br>H -2.95716 -2.24436 0.37773											

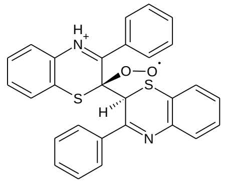<br>S -1.15884 -1.01830 -1.58247<br>C 0.17966 -0.42551 -0.48082<br>C 0.93007 -1.67024 -0.02813<br>N 0.21854 -2.59449 0.57776<br>C -1.78451 -3.56112 1.52670<br>C -3.15357 -3.76732 1.45865<br>C -3.90932 -3.13389 0.46777<br>C -3.30665 -2.27589 -0.44498<br>C -1.92976 -2.04963 -0.38340<br>C -1.17748 -2.71131 0.59809<br>C 2.33615 -1.94031 -0.28355<br>C 2.71010 -3.26258 -0.60204<br>C 4.04273 -3.57655 -0.82373<br>C 5.01807 -2.58375 -0.72360<br>C 4.65636 -1.27193 -0.41419<br>C 3.32512 -0.94177 -0.20645<br>H -1.18280 -4.05281 2.28511<br>H -3.63359 -4.42439 2.17578<br>H -4.98068 -3.29833 0.41496<br>H -3.90065 -1.76882 -1.19840<br>H 1.95368 -4.03132 -0.73245<br>H 4.31864 -4.59189 -1.08873<br>H 6.06105 -2.83080 -0.89677<br>H 5.41567 -0.50080 -0.33431<br>H 3.06292 0.07744 0.04696<br>S 0.92395 0.77480 1.96758<br>C -0.32880 0.45323 0.69812<br>C -0.94846 1.76327 0.22197<br>N -0.25694 2.82071 -0.00843<br>C 1.80583 3.97654 -0.33993<br>C 3.13804 4.21310 -0.03062<br>C 3.78617 3.41418 0.91480<br>C 3.10942 2.35953 1.52035<br>C 1.77859 2.10016 1.18380<br>C 1.10109 2.92387 0.26289<br>C -2.41022 1.82809 -0.00290<br>C -3.31825 1.07733 0.75598<br>C -4.68961 1.20525 0.54611<br>C -5.17171 2.06852 -0.43506<br>C -4.27419 2.81672 -1.20014<br>C -2.90726 2.70439 -0.98121<br>H -1.08267 -0.14126 1.21931<br>H 1.27387 4.60434 -1.04863	-2133.27514	-2132.83148	-2132.91898	-2133.30516	-2132.94900	-2134.52553	-2134.16937	-2134.92237	-2134.56621	2.4

H 3.66849 5.02954 -0.51074<br>H 4.82167 3.60696 1.17809<br>H 3.61587 1.72989 2.24696<br>H -2.97494 0.41325 1.54369<br>H -5.38012 0.62834 1.15409<br>H -6.24079 2.15934 -0.60357<br>H -4.64255 3.48763 -1.97073<br>H -2.20404 3.28322 -1.57105<br>O 1.08406 0.36091 -1.28500<br>O 0.62538 0.62696 -2.47731<br>H 0.73664 -3.36435 0.99502										
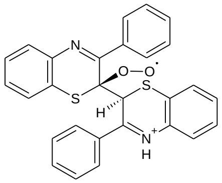<br>S 1.39712 -0.90659 1.65333<br>C -0.04818 -0.46065 0.63591<br>C -0.81992 -1.73380 0.26343<br>N -0.20853 -2.73820 -0.24701<br>C 1.73025 -3.76023 -1.21150<br>C 3.09924 -3.98327 -1.24263<br>C 3.93940 -3.28899 -0.36758<br>C 3.41286 -2.34780 0.51072<br>C 2.03769 -2.09829 0.52283<br>C 1.17693 -2.82074 -0.32823<br>C -2.29567 -1.82141 0.40870<br>C -3.02661 -2.33949 -0.67073<br>C -4.40446 -2.50082 -0.57839<br>C -5.07091 -2.16319 0.60000<br>C -4.34920 -1.66685 1.68383<br>C -2.97038 -1.48927 1.59143<br>H 1.05521 -4.31241 -1.85838<br>H 3.51351 -4.70898 -1.93546<br>H 5.00957 -3.47128 -0.37673<br>H 4.06808 -1.79231 1.17552<br>H -2.50274 -2.60198 -1.58433<br>H -4.95833 -2.89087 -1.42736<br>H -6.14696 -2.29086 0.67424<br>H -4.85780 -1.41872 2.61071<br>H -2.42669 -1.12180 2.45406<br>S -0.92562 0.45530 -1.93170<br>C 0.36354 0.32062 -0.66183<br>C 0.93238 1.67902 -0.36123<br>N 0.10670 2.68734 -0.22095<br>C -2.02540 3.75855 0.12936<br>C -3.39026 3.82256 -0.11217	-<br>2133.27657	-<br>2132.83300	-<br>2132.92114	-<br>2133.30743	-<br>2132.95200	-<br>2134.52854	-<br>2134.17310	-<br>2134.92541	-<br>2134.56998	0.0

C -4.00882 2.84284 -0.89234<br>C -3.27073 1.79078 -1.42533<br>C -1.89872 1.71172 -1.18581<br>C -1.28478 2.70525 -0.41028<br>C 2.36033 1.89766 -0.18028<br>C 3.29234 1.14666 -0.91765<br>C 4.65161 1.39205 -0.77402<br>C 5.09790 2.36434 0.11997<br>C 4.17992 3.10248 0.86991<br>C 2.81997 2.87974 0.71900<br>H 1.13780 -0.28998 -1.13339<br>H -1.53341 4.51192 0.73800<br>H -3.97201 4.63544 0.30884<br>H -5.07633 2.89339 -1.08110<br>H -3.75576 1.02560 -2.02310<br>H 2.96605 0.40545 -1.63940<br>H 5.36302 0.82242 -1.36291<br>H 6.16200 2.54409 0.23807<br>H 4.52623 3.84356 1.58279<br>H 2.12373 3.42720 1.34841<br>O -0.90095 0.40237 1.41973<br>O -0.27803 0.98992 2.40820<br>H 0.52358 3.59152 -0.01263										
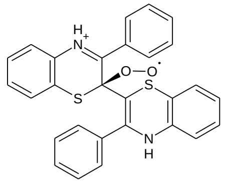<br>S -2.10883 -0.73723 -1.35147<br>C -0.88997 -0.30485 -0.07852<br>C -1.11733 1.07876 0.52258<br>N -2.32938 1.57036 0.60579<br>C -4.71232 1.79757 0.43571<br>C -5.92513 1.39615 -0.10068<br>C -5.96849 0.32984 -1.00329<br>C -4.80543 -0.34077 -1.36444<br>C -3.57884 0.05607 -0.82912<br>C -3.54103 1.12993 0.06327<br>C -0.04589 1.82292 1.18020<br>C 0.95711 1.15914 1.90708<br>C 1.91990 1.89644 2.58682<br>C 1.90672 3.28929 2.53620<br>C 0.92107 3.95402 1.80414<br>C -0.05326 3.22933 1.13312<br>H -4.66461 2.62159 1.14220<br>H -6.83610 1.91110 0.18424<br>H -6.91723 0.01281 -1.42422	-2133.27770	-2132.83376	-2132.92209	-2133.30805	-2132.95244	-2134.52695	-2134.17134	-2134.92435	-2134.56875	0.8

H -4.84568 -1.17492 -2.05799<br>H 0.96079 0.07812 1.97993<br>H 2.68020 1.37801 3.16226<br>H 2.66688 3.85851 3.06247<br>H 0.91935 5.03771 1.74592<br>H -0.78582 3.75586 0.52680<br>C 0.45528 -0.43815 -0.71675<br>S 0.78898 0.90172 -1.84163<br>C 2.50106 1.13413 -1.44575<br>C 3.26450 -0.00675 -1.17156<br>N 2.64100 -1.25799 -1.08177<br>C 1.36754 -1.43920 -0.58086<br>C 3.09794 2.39233 -1.48834<br>C 4.47409 2.51219 -1.30324<br>C 5.24090 1.37443 -1.05188<br>C 4.64076 0.12073 -0.97021<br>C 1.15440 -2.74719 0.08510<br>C 0.14958 -3.61426 -0.35922<br>C -0.01680 -4.85835 0.24270<br>C 0.81656 -5.24547 1.29162<br>C 1.82645 -4.38946 1.73143<br>C 2.00307 -3.14896 1.12509<br>H 2.48890 3.26882 -1.68913<br>H 4.94319 3.48937 -1.35943<br>H 6.31336 1.46091 -0.90640<br>H 5.23584 -0.76137 -0.74898<br>H -0.49177 -3.32001 -1.18470<br>H -0.79474 -5.52779 -0.11203<br>H 0.68343 -6.21526 1.76208<br>H 2.47717 -4.68696 2.54827<br>H 2.78599 -2.48102 1.47531<br>O -0.95582 -1.22351 1.08949<br>O -2.16724 -1.36707 1.54114<br>H 3.25814 -2.05274 -0.98775<br>H -2.41129 2.41434 1.17016										
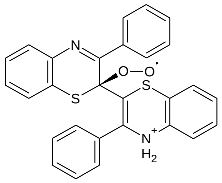	n.d.									
**Single-bond dimer, C3-peroxyl radical**										

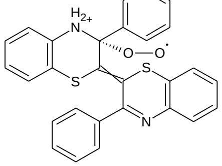<br>S 0.84173 -1.74644 1.44341<br>C 0.93420 -0.22921 0.54895<br>C 2.25263 -0.02750 -0.11285<br>N 2.82105 -0.97043 -0.78398<br>C 2.80351 -3.12853 -1.81763<br>C 2.46371 -4.47278 -1.81637<br>C 1.66437 -4.99113 -0.79218<br>C 1.17845 -4.15620 0.20775<br>C 1.48892 -2.79442 0.18362<br>C 2.32908 -2.26464 -0.81667<br>C 2.96781 1.25823 0.03482<br>C 2.85356 2.00939 1.21329<br>C 3.57610 3.19039 1.36600<br>C 4.40554 3.64026 0.34062<br>C 4.52207 2.89643 -0.83634<br>C 3.81671 1.70877 -0.98640<br>H 3.45245 -2.71317 -2.58295<br>H 2.83405 -5.12516 -2.60102<br>H 1.41026 -6.04654 -0.77714<br>H 0.54058 -4.55293 0.99243<br>H 2.21900 1.65983 2.02351<br>H 3.49030 3.75718 2.28830<br>H 4.96130 4.56620 0.45630<br>H 5.16522 3.24573 -1.63888<br>H 3.90315 1.12423 -1.89681<br>S 0.06414 2.23370 -0.27165<br>C -0.10715 0.63433 0.49734<br>C -1.42552 0.29643 1.16790<br>N -2.33446 1.51898 1.15262<br>C -3.95522 2.07256 -0.64480<br>C -4.16952 2.56353 -1.93110<br>C -3.09029 2.94751 -2.72629<br>C -1.78160 2.83613 -2.25939<br>C -1.55709 2.35328 -0.97199<br>C -2.64962 1.99636 -0.18936<br>C -2.17701 -0.89197 0.62506<br>C -3.08784 -1.57012 1.44326<br>C -3.83198 -2.62373 0.92059<br>C -3.68057 -2.99488 -0.41469<br>C -2.78185 -2.31018 -1.23079<br>C -2.02934 -1.25933 -0.71515<br>H -4.78777 1.77184 -0.01635<br>H -5.18282 2.64186 -2.30991	-<br>2133.25900			-<br>2133.29152				-<br>2134.91265		

H -3.26602 3.32574 -3.72822<br>H -0.94232 3.11518 -2.88793<br>H -3.20661 -1.29934 2.48798<br>H -4.52823 -3.15578 1.56117<br>H -4.26262 -3.81772 -0.81861<br>H -2.66150 -2.59410 -2.27153<br>H -1.32580 -0.73508 -1.35451<br>H -3.19625 1.28460 1.65834<br>O -1.21356 0.05710 2.60400<br>O -0.81852 1.13348 3.22903<br>H -1.85201 2.25301 1.69983										
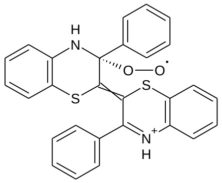<br>S 1.85695 0.10977 1.41054<br>C 0.51650 0.59701 0.35585<br>C 0.50144 2.04265 0.11895<br>N 1.64161 2.63009 -0.17374<br>C 3.91830 2.64142 -0.99040<br>C 5.18675 2.07789 -1.00444<br>C 5.43759 0.90082 -0.29585<br>C 4.42221 0.27474 0.42081<br>C 3.14239 0.82913 0.43882<br>C 2.90079 2.01618 -0.26758<br>C -0.68991 2.87460 0.29368<br>C -1.63609 2.51430 1.26654<br>C -2.73789 3.32746 1.49366<br>C -2.91495 4.49251 0.74611<br>C -1.98185 4.84944 -0.22832<br>C -0.86843 4.05028 -0.45303<br>H 3.70995 3.55597 -1.53779<br>H 5.98012 2.55647 -1.56855<br>H 6.43015 0.46187 -0.30599<br>H 4.61719 -0.64451 0.96394<br>H -1.49351 1.61229 1.85334<br>H -3.45982 3.05259 2.25603<br>H -3.78305 5.12113 0.91965<br>H -2.12752 5.74597 -0.82231<br>H -0.17450 4.31836 -1.24519<br>S -1.51416 0.30213 -1.39231<br>C -0.40579 -0.26065 -0.16349<br>C -0.46413 -1.72611 0.22204<br>N -1.53199 -2.42771 -0.35070<br>C -3.96156 -2.65840 -0.19492<br>C -5.22393 -2.11632 -0.41873	-2133.28525	-2132.84164	-2132.93092	-2133.31535	-2132.96102	-2134.53307	-2134.17874	-2134.93112	-2134.57678	0.0

C -5.36475 -0.81150 -0.89367<br>C -4.23289 -0.04363 -1.15486<br>C -2.96959 -0.59753 -0.95430<br>C -2.82142 -1.90084 -0.47029<br>C 0.82447 -2.50162 -0.01734<br>C 1.30210 -3.43581 0.90351<br>C 2.43163 -4.19482 0.60005<br>C 3.08815 -4.02537 -0.61680<br>C 2.60605 -3.09716 -1.53966<br>C 1.47468 -2.34252 -1.24486<br>H -3.85441 -3.66923 0.18849<br>H -6.10446 -2.71547 -0.20780<br>H -6.35135 -0.38896 -1.05448<br>H -4.32609 0.97469 -1.52044<br>H 0.81145 -3.57024 1.86130<br>H 2.79953 -4.91626 1.32361<br>H 3.97117 -4.61442 -0.84659<br>H 3.10854 -2.96146 -2.49276<br>H 1.09807 -1.62905 -1.97288<br>H -1.48871 -3.42258 -0.17257<br>H 1.63260 3.64210 -0.26611<br>O -0.64863 -1.59650 1.77337<br>O -1.36719 -2.55017 2.26633										
**Single-bond dimer, dioxolane radical**										
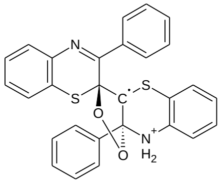<br>S -1.86074 -1.67129 -0.73770<br>C -1.13510 -0.20988 0.10870<br>C -2.04890 0.99797 -0.13690<br>N -3.32518 0.91466 -0.02294<br>C -5.32342 -0.17916 0.69095<br>C -6.09712 -1.31733 0.86281<br>C -5.57810 -2.57192 0.52743<br>C -4.27810 -2.68712 0.04821<br>C -3.48887 -1.54344 -0.10044<br>C -4.00924 -0.27036 0.20594<br>C -1.45758 2.30356 -0.49986<br>C -2.09324 3.08203 -1.47689<br>C -1.58381 4.32946 -1.82233<br>C -0.44255 4.81943 -1.18618<br>C 0.18625 4.05682 -0.20234<br>C -0.31318 2.80206 0.13637<br>H -5.71368 0.80725 0.92294<br>H -7.10940 -1.23110 1.24523	-<br>2133.27135	-<br>2132.82676	-<br>2132.91260	-<br>2133.30425	-<br>2132.94549	-<br>2134.52562	-<br>2134.16687	-<br>2134.92276	-<br>2134.56401	15.0

H -6.18466 -3.46431 0.64792<br>H -3.86833 -3.66198 -0.19946<br>H -2.98087 2.69297 -1.96627<br>H -2.07617 4.91900 -2.59010<br>H -0.04576 5.79421 -1.45462<br>H 1.06475 4.44033 0.30815<br>H 0.17042 2.23133 0.92306<br>S 0.89455 -0.13396 -1.86725<br>C 0.31265 -0.16308 -0.25821<br>C 1.06544 -0.74832 0.89592<br>N 1.71674 -2.06117 0.42475<br>C 3.91040 -2.55770 -0.56135<br>C 4.83874 -2.41717 -1.58810<br>C 4.55191 -1.59247 -2.67458<br>C 3.34883 -0.89837 -2.73764<br>C 2.40418 -1.03896 -1.71709<br>C 2.70294 -1.87956 -0.64314<br>C 2.09784 0.10294 1.59947<br>C 2.34569 -0.13442 2.95629<br>C 3.31491 0.60818 3.62399<br>C 4.03714 1.58831 2.94418<br>C 3.78514 1.82962 1.59507<br>C 2.81620 1.09096 0.92071<br>H 4.12108 -3.19904 0.28995<br>H 5.78264 -2.94821 -1.53323<br>H 5.27461 -1.47607 -3.47551<br>H 3.14013 -0.23995 -3.57533<br>H 1.77158 -0.88259 3.49459<br>H 3.49952 0.42451 4.67783<br>H 4.79047 2.16914 3.46787<br>H 4.33743 2.59880 1.06423<br>H 2.61847 1.30360 -0.12518<br>O -1.07438 -0.35515 1.52443<br>O 0.04808 -1.21734 1.76582<br>H 0.95411 -2.68709 0.12938<br>H 2.16803 -2.51387 1.22691										
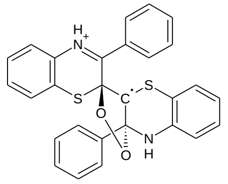<br>S -1.69709 -1.84289 -0.66339<br>C -0.96045 -0.30932 0.07640<br>C -1.83639 0.83670 -0.39086<br>N -3.12654 0.73429 -0.15910<br>C -5.14513 -0.24371 0.72623<br>C -5.90301 -1.36832 1.00768	-2133.28881	-2132.84465	-2132.93168	-2133.31948	-2132.96236	-2134.53613	-2134.17901	-2134.93348	-2134.57636	7.3

C -5.37450 -2.64371 0.78035<br>C -4.08525 -2.80004 0.28754<br>C -3.30401 -1.67518 0.00634<br>C -3.85044 -0.40053 0.22086<br>C -1.34508 2.03200 -1.05475<br>C -2.10799 2.59916 -2.09449<br>C -1.67835 3.76441 -2.71359<br>C -0.49715 4.38048 -2.29754<br>C 0.26062 3.82582 -1.26531<br>C -0.14986 2.65165 -0.64902<br>H -5.54379 0.75265 0.89383<br>H -6.90665 -1.25502 1.40310<br>H -5.96979 -3.52393 1.00104<br>H -3.67426 -3.79167 0.12750<br>H -3.00475 2.10066 -2.45210<br>H -2.25780 4.18539 -3.52870<br>H -0.16365 5.29262 -2.78299<br>H 1.17359 4.31140 -0.93599<br>H 0.43260 2.24022 0.16748<br>S 1.16265 -0.57083 -1.75296<br>C 0.49987 -0.30711 -0.20843<br>C 1.19076 -0.59721 1.10286<br>N 2.11533 -1.69043 1.01880<br>C 4.15446 -2.59225 0.11075<br>C 5.01451 -2.83952 -0.94928<br>C 4.71256 -2.37187 -2.23062<br>C 3.53679 -1.66294 -2.43174<br>C 2.65637 -1.43126 -1.36906<br>C 2.95603 -1.88511 -0.07494<br>C 1.79311 0.64592 1.75350<br>C 1.31907 1.13511 2.97228<br>C 1.90638 2.26107 3.54978<br>C 2.96536 2.90591 2.91491<br>C 3.44067 2.41961 1.69600<br>C 2.85778 1.29667 1.11659<br>H 4.40137 -2.94860 1.10756<br>H 5.93178 -3.39222 -0.77055<br>H 5.38583 -2.55361 -3.06172<br>H 3.28711 -1.28676 -3.42038<br>H 0.49479 0.63631 3.47034<br>H 1.53165 2.63283 4.49908<br>H 3.41985 3.78326 3.36577<br>H 4.26671 2.91538 1.19451<br>H 3.23786 0.93054 0.16633<br>O -1.02292 -0.29641 1.49016<br>O 0.08849 -1.10206 1.89418<br>H 2.58175 -1.85865 1.90264<br>H -3.67966 1.57087 -0.33254										

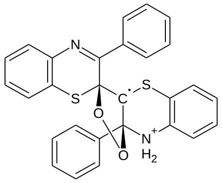<br>S 1.21067 -1.98723 1.65200<br>C 1.04307 -0.28421 0.95210<br>C 2.34614 0.07651 0.24084<br>N 2.85313 -0.72899 -0.61848<br>C 2.74491 -2.65845 -2.02683<br>C 2.41669 -3.98718 -2.25442<br>C 1.74150 -4.71601 -1.27142<br>C 1.36758 -4.10477 -0.07917<br>C 1.66880 -2.75826 0.14068<br>C 2.38241 -2.02252 -0.82929<br>C 2.97515 1.40471 0.42366<br>C 3.42790 2.08011 -0.71945<br>C 4.06309 3.31202 -0.60493<br>C 4.26863 3.87835 0.65335<br>C 3.83376 3.20618 1.79496<br>C 3.18333 1.97984 1.68514<br>H 3.29691 -2.08230 -2.76348<br>H 2.69906 -4.46198 -3.18887<br>H 1.49449 -5.76016 -1.43732<br>H 0.82552 -4.66459 0.67744<br>H 3.26811 1.63099 -1.69471<br>H 4.39726 3.83040 -1.49890<br>H 4.76826 4.83848 0.74409<br>H 4.00407 3.63485 2.77811<br>H 2.86672 1.46290 2.58467<br>S -0.21180 0.10097 -1.58931<br>C -0.18583 -0.13143 0.10490<br>C -1.25243 0.36590 1.02151<br>N -1.54316 1.83976 0.60300<br>C -3.21757 2.78242 -0.93067<br>C -3.78114 2.88076 -2.19931<br>C -3.26346 2.12492 -3.24950<br>C -2.18987 1.26653 -3.03964<br>C -1.60765 1.16830 -1.77295<br>C -2.13414 1.93882 -0.73279<br>C -2.55964 -0.37589 1.13944<br>C -2.79624 -1.52316 0.38155<br>C -3.99818 -2.21188 0.52765<br>C -4.95705 -1.76306 1.43324<br>C -4.71478 -0.62340 2.20001<br>C -3.51856 0.07142 2.05591<br>H -3.61630 3.36313 -0.10360<br>H -4.62584 3.54164 -2.36004	-2133.27033	-2132.82582	-2132.91278	-2133.30482	-2132.94727	-2134.52567	-2134.16811	-2134.92295	-2134.56539	14.2

H -3.70336 2.19360 -4.23901<br>H -1.80056 0.66742 -3.85691<br>H -2.04745 -1.88152 -0.31785<br>H -4.18145 -3.10114 -0.06720<br>H -5.89259 -2.30261 1.54551<br>H -5.45440 -0.27581 2.91429<br>H -3.33955 0.94554 2.67763<br>O 0.77037 0.59487 2.02939<br>O -0.64476 0.39757 2.28157<br>H -2.17343 2.26255 1.29144<br>H -0.66058 2.36433 0.66508										
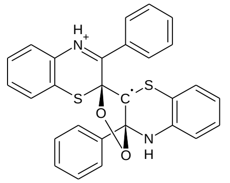<br>S -1.49315 1.98456 1.51785<br>C -0.92263 0.29683 0.94906<br>C -2.10927 -0.38806 0.32370<br>N -2.77352 0.27918 -0.59261<br>C -3.20616 2.07071 -2.14611<br>C -3.18414 3.42132 -2.45688<br>C -2.63069 4.33671 -1.55690<br>C -2.08740 3.90481 -0.35263<br>C -2.09907 2.54628 -0.02573<br>C -2.67051 1.63777 -0.92917<br>C -2.52062 -1.74640 0.63734<br>C -3.89446 -2.05718 0.63984<br>C -4.31090 -3.35849 0.88192<br>C -3.36727 -4.36098 1.11016<br>C -2.00485 -4.05844 1.10803<br>C -1.57379 -2.75791 0.88552<br>H -3.63311 1.34946 -2.83697<br>H -3.59713 3.76191 -3.40029<br>H -2.61205 5.39409 -1.80078<br>H -1.64629 4.61598 0.33842<br>H -4.63525 -1.27348 0.50723<br>H -5.37114 -3.58842 0.90419<br>H -3.69511 -5.37916 1.29672<br>H -1.27316 -4.84136 1.28005<br>H -0.51304 -2.53034 0.87083<br>S 0.42342 0.16041 -1.58520<br>C 0.30685 0.29205 0.10686<br>C 1.44115 -0.13523 1.00425<br>N 1.75209 -1.50747 0.67281<br>C 3.04460 -2.86580 -0.86372<br>C 3.46255 -3.17253 -2.15065	-2133.29006	-2132.84595	-2132.93167	-2133.31955	-2132.96116	-2134.53719	-2134.17880	-2134.93405	-2134.57566	7.7

C 3.01757 -2.41839 -3.23992<br>C 2.13058 -1.37271 -3.02700<br>C 1.68341 -1.07696 -1.73456<br>C 2.15816 -1.80449 -0.62655<br>C 2.62647 0.81974 1.06345<br>C 2.38684 2.19129 1.20100<br>C 3.44996 3.08408 1.28058<br>C 4.76309 2.61401 1.22124<br>C 5.00513 1.24947 1.08444<br>C 3.93993 0.35229 1.00538<br>H 3.41303 -3.43987 -0.01706<br>H 4.15300 -3.99649 -2.30268<br>H 3.35503 -2.64618 -4.24565<br>H 1.76361 -0.78715 -3.86573<br>H 1.36462 2.55774 1.24799<br>H 3.25451 4.14695 1.38756<br>H 5.59388 3.31115 1.27991<br>H 6.02404 0.87703 1.03702<br>H 4.14691 -0.70677 0.89254<br>O -0.54846 -0.39949 2.11442<br>O 0.84564 -0.06995 2.29826<br>H 2.25293 -2.00226 1.40207<br>H -3.46420 -0.25161 -1.11811										
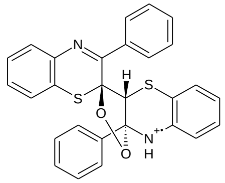<br>S 1.66818 -0.92468 1.40540<br>C -0.01732 -0.75034 0.74792<br>C -0.55836 -2.09554 0.25527<br>N 0.15963 -2.91742 -0.41933<br>C 2.11565 -3.54843 -1.63166<br>C 3.48800 -3.52545 -1.83882<br>C 4.30420 -2.72910 -1.02984<br>C 3.74210 -1.93513 -0.03708<br>C 2.35606 -1.93189 0.14689<br>C 1.52415 -2.75719 -0.63673<br>C -2.00233 -2.40295 0.40321<br>C -2.70438 -2.25853 1.60935<br>C -4.04867 -2.61536 1.68195<br>C -4.71317 -3.10123 0.55706<br>C -4.02252 -3.24609 -0.64646<br>C -2.67600 -2.90788 -0.72096<br>H 1.46805 -4.18523 -2.22663<br>H 3.92736 -4.14011 -2.61817<br>H 5.37963 -2.72006 -1.17743	-2133.29740	-2132.85434	-2132.94151	-2133.32688	-2132.97099	-2134.54813	-2134.19224	-2134.94386	-2134.58796	0.0

H 4.37167 -1.30430 0.58400<br>H -2.20012 -1.89307 2.49520<br>H -4.57707 -2.51456 2.62542<br>H -5.76413 -3.36876 0.61818<br>H -4.53268 -3.62326 -1.52796<br>H -2.13104 -3.02476 -1.65261<br>S 1.11160 0.77007 -1.43951<br>C -0.25549 0.33852 -0.36730<br>C -0.85942 1.50070 0.42975<br>N 0.17423 2.28729 1.07869<br>C 2.33307 3.09260 1.67458<br>C 3.65942 3.24431 1.37664<br>C 4.21442 2.64780 0.21452<br>C 3.42787 1.88580 -0.62338<br>C 2.06781 1.69213 -0.33593<br>C 1.48944 2.33392 0.81557<br>C -1.86216 2.35387 -0.32036<br>C -1.54188 3.65569 -0.70768<br>C -2.46515 4.41349 -1.42628<br>C -3.70653 3.87588 -1.75624<br>C -4.02576 2.57379 -1.36943<br>C -3.10793 1.81045 -0.65528<br>H -1.03353 -0.02931 -1.04211<br>H 1.90097 3.55158 2.55831<br>H 4.29464 3.82712 2.03516<br>H 5.26824 2.77893 -0.00656<br>H 3.85565 1.40968 -1.49987<br>H -0.57678 4.08453 -0.45730<br>H -2.20936 5.42509 -1.72605<br>H -4.42531 4.46867 -2.31409<br>H -4.99299 2.15040 -1.62237<br>H -3.36469 0.79978 -0.35247<br>O -0.69515 -0.22414 1.88770<br>O -1.58536 0.80827 1.42686<br>H -0.15848 2.83626 1.86547										
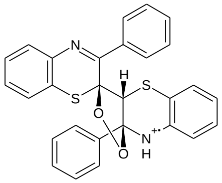<br>S 1.57488 -1.16652 0.78100<br>C 0.88913 0.14267 -0.26770<br>C 1.68446 1.44893 -0.14384<br>N 2.94350 1.51659 0.09129<br>C 5.15005 0.64162 0.28415<br>C 6.03992 -0.38970 0.54471<br>C 5.55606 -1.66060 0.87099	-2133.27747	-2132.83413	-2132.92007	-2133.30741	-2132.95001	-2134.52693	-2134.16953	-2134.92316	-2134.56577	13.9

C 4.18735 -1.89918 0.91899<br>C 3.29348 -0.86025 0.64585<br>C 3.76395 0.42432 0.33212<br>C 0.97863 2.73946 -0.37188<br>C 0.22418 2.97996 -1.52931<br>C -0.37843 4.22061 -1.72610<br>C -0.24430 5.22667 -0.76995<br>C 0.50656 4.99306 0.38186<br>C 1.12003 3.75877 0.57775<br>H 5.49893 1.63993 0.03736<br>H 7.10914 -0.20786 0.50095<br>H 6.24708 -2.47091 1.08231<br>H 3.80981 -2.88856 1.16142<br>H 0.13299 2.21197 -2.29221<br>H -0.94786 4.40180 -2.63297<br>H -0.71941 6.19102 -0.92452<br>H 0.61689 5.77345 1.12911<br>H 1.71120 3.57383 1.46966<br>S -1.27170 0.16470 1.65035<br>C -0.60729 0.24581 -0.01176<br>C -1.18990 -0.77713 -0.99269<br>N -2.57858 -0.40295 -1.18132<br>C -4.76429 0.23298 -0.51864<br>C -5.64990 0.61188 0.44961<br>C -5.20702 0.83543 1.77782<br>C -3.87274 0.69378 2.10984<br>C -2.92813 0.32815 1.14335<br>C -3.38375 0.05766 -0.20669<br>C -1.07794 -2.27662 -0.75348<br>C -0.24972 -3.04274 -1.58013<br>C -0.14823 -4.41770 -1.38840<br>C -0.87261 -5.04100 -0.37447<br>C -1.71416 -4.28404 0.43946<br>C -1.82449 -2.91014 0.24691<br>H -0.92456 1.21582 -0.40764<br>H -5.09259 0.04979 -1.53701<br>H -6.69808 0.73954 0.20244<br>H -5.91862 1.13685 2.53897<br>H -3.53993 0.88859 3.12430<br>H 0.32083 -2.56330 -2.36687<br>H 0.50027 -5.00094 -2.03511<br>H -0.79155 -6.11362 -0.22533<br>H -2.29524 -4.76219 1.22195<br>H -2.50484 -2.35189 0.88077<br>O 0.89805 -0.20259 -1.68428<br>O -0.47126 -0.37890 -2.15151<br>H -3.01967 -0.71367 -2.04291										

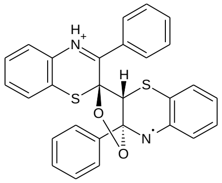<br>S -1.29324 -1.48746 -1.41753<br>C 0.22379 -0.72556 -0.75396<br>C 1.20620 -1.78487 -0.28221<br>N 0.75496 -2.82253 0.36877<br>C -0.82349 -4.18091 1.58096<br>C -2.12177 -4.63025 1.77494<br>C -3.16006 -4.11252 0.99785<br>C -2.91061 -3.13611 0.03863<br>C -1.61098 -2.66745 -0.15751<br>C -0.57569 -3.20465 0.61448<br>C 2.65681 -1.63561 -0.40791<br>C 3.45567 -1.90366 0.72108<br>C 4.83776 -1.80654 0.63431<br>C 5.43831 -1.46400 -0.57701<br>C 4.65215 -1.20707 -1.69994<br>C 3.26688 -1.27726 -1.62252<br>H -0.00365 -4.57893 2.17223<br>H -2.32294 -5.38447 2.52790<br>H -4.17615 -4.46396 1.14573<br>H -3.72351 -2.72828 -0.55384<br>H 2.99733 -2.13777 1.67824<br>H 5.44406 -1.99131 1.51517<br>H 6.51968 -1.39446 -0.64467<br>H 5.11942 -0.95096 -2.64542<br>H 2.66433 -1.08923 -2.50115<br>S -1.39237 0.40730 1.38388<br>C 0.07060 0.38153 0.34505<br>C 0.28783 1.67483 -0.47525<br>N -0.83092 2.16132 -1.21328<br>C -3.10309 2.53237 -1.66475<br>C -4.43639 2.43985 -1.34611<br>C -4.83145 1.74341 -0.18829<br>C -3.88184 1.12778 0.62545<br>C -2.52670 1.20504 0.31745<br>C -2.08801 1.94656 -0.83699<br>C 0.97166 2.77592 0.32228<br>C 0.27891 3.93809 0.66316<br>C 0.90347 4.92593 1.42348<br>C 2.22336 4.76294 1.83752<br>C 2.91948 3.60471 1.49128<br>C 2.29720 2.61171 0.73984<br>H 0.90270 0.28549 1.04820<br>H -2.76330 3.06988 -2.54466	-2133.28874	-2132.84569	-2132.93266	-2133.32040	-2132.96432	-2134.53974	-2134.18366	-2134.93534	-2134.57926	5.5

H -5.18359 2.90268 -1.98274<br>H -5.88300 1.66796 0.07017<br>H -4.20313 0.57090 1.50130<br>H -0.74549 4.07925 0.33467<br>H 0.35494 5.82527 1.68738<br>H 2.70979 5.53528 2.42612<br>H 3.95050 3.47228 1.80571<br>H 2.85004 1.71645 0.47041<br>O 0.71249 -0.03392 -1.89079<br>O 1.26442 1.20698 -1.41469<br>H 1.45815 -3.48482 0.69114										
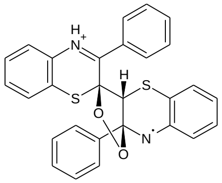<br>S 1.43207 -1.18400 0.91914<br>C 0.83659 0.11547 -0.20068<br>C 1.70341 1.35839 -0.11917<br>N 2.99685 1.24117 0.04044<br>C 5.15994 0.19495 0.15022<br>C 5.93996 -0.91797 0.41572<br>C 5.33657 -2.11754 0.80851<br>C 3.95593 -2.21118 0.92810<br>C 3.15808 -1.09497 0.66261<br>C 3.77007 0.10242 0.28057<br>C 1.18082 2.71476 -0.30072<br>C 0.34900 3.02791 -1.38967<br>C -0.07787 4.33711 -1.57072<br>C 0.29965 5.33233 -0.66788<br>C 1.12100 5.02314 0.41513<br>C 1.57214 3.72073 0.59772<br>H 5.61422 1.13102 -0.16144<br>H 7.01798 -0.85511 0.31513<br>H 5.94840 -2.98974 1.01490<br>H 3.49029 -3.14599 1.22399<br>H 0.07706 2.26506 -2.11323<br>H -0.70328 4.58175 -2.42324<br>H -0.04767 6.35102 -0.81027<br>H 1.40837 5.79405 1.12277<br>H 2.19158 3.47658 1.45647<br>S -1.36505 0.18527 1.67276<br>C -0.65145 0.32214 0.03160<br>C -1.28823 -0.62774 -0.99468<br>N -2.61638 -0.22524 -1.30720<br>C -4.75742 0.46877 -0.64817<br>C -5.69212 0.83158 0.29321	-<br>2133.27750	-<br>2132.83388	-<br>2132.91852	-<br>2133.30840	-<br>2132.94942	-<br>2134.52541	-<br>2134.16643	-<br>2134.92144	-<br>2134.56246	16.0

C -5.31095 0.96158 1.63929<br>C -3.98649 0.75234 2.02026<br>C -3.02047 0.38904 1.08637<br>C -3.39535 0.19889 -0.29935<br>C -1.24524 -2.14149 -0.76946<br>C -0.45670 -2.94458 -1.60063<br>C -0.43292 -4.32699 -1.43377<br>C -1.19954 -4.92859 -0.43794<br>C -2.00594 -4.13739 0.37781<br>C -2.03790 -2.75520 0.20745<br>H -0.90214 1.32335 -0.32791<br>H -5.02284 0.34747 -1.69379<br>H -6.72021 1.01479 -0.00190<br>H -6.04165 1.24576 2.38981<br>H -3.69841 0.89096 3.05858<br>H 0.14542 -2.48952 -2.37799<br>H 0.18804 -4.93304 -2.08713<br>H -1.17964 -6.00663 -0.30689<br>H -2.62416 -4.59440 1.14489<br>H -2.69834 -2.17049 0.83713<br>O 0.88204 -0.27883 -1.59039<br>O -0.46347 -0.26663 -2.12268<br>H 3.52524 2.10759 -0.04878										
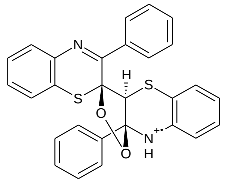<br>S 0.85855 2.29919 -1.63638<br>C -0.01408 0.82983 -0.96715<br>C -1.39243 1.28605 -0.49097<br>N -1.49659 2.28338 0.31023<br>C -0.52271 3.86502 1.82185<br>C 0.45994 4.78682 2.15290<br>C 1.55594 4.97472 1.30560<br>C 1.68350 4.21556 0.14742<br>C 0.71352 3.26165 -0.17127<br>C -0.41812 3.09042 0.65583<br>C -2.62611 0.55295 -0.86212<br>C -2.88725 0.11228 -2.16775<br>C -4.09339 -0.51596 -2.46615<br>C -5.04491 -0.72648 -1.46903<br>C -4.79260 -0.29001 -0.16827<br>C -3.59676 0.35316 0.13152<br>H -1.39773 3.72673 2.44986<br>H 0.36738 5.37238 3.06230<br>H 2.31954 5.70572 1.55302	-2133.29667	-2132.85321	-2132.94012	-2133.32677	-2132.97021	-2134.54763	-2134.19108	-2134.94367	-2134.58711	0.5

H 2.54400 4.34601 -0.50223<br>H -2.16722 0.28186 -2.96040<br>H -4.28995 -0.83841 -3.48435<br>H -5.98041 -1.22519 -1.70534<br>H -5.52844 -0.44983 0.61444<br>H -3.39838 0.70199 1.14013<br>S 0.19779 -0.16490 1.76624<br>C 0.87491 0.10216 0.11554<br>C 1.32759 -1.16461 -0.63896<br>N 0.45392 -2.30955 -0.42524<br>C -1.29347 -3.69985 0.39029<br>C -2.21848 -3.97857 1.35558<br>C -2.41059 -3.09210 2.44922<br>C -1.68012 -1.92508 2.54857<br>C -0.73243 -1.59988 1.56847<br>C -0.50786 -2.51260 0.47923<br>C 2.78566 -1.53435 -0.45328<br>C 3.77045 -0.75441 -1.06951<br>C 5.11365 -1.06226 -0.87909<br>C 5.48022 -2.13909 -0.07117<br>C 4.49843 -2.90983 0.54613<br>C 3.15011 -2.61157 0.35521<br>H 1.75913 0.71380 0.30404<br>H -1.13278 -4.37121 -0.44754<br>H -2.81263 -4.88348 1.28866<br>H -3.15058 -3.32749 3.20664<br>H -1.84505 -1.24222 3.37580<br>H 3.49059 0.08606 -1.69737<br>H 5.87502 -0.45875 -1.36364<br>H 6.52982 -2.37512 0.07638<br>H 4.77656 -3.74739 1.17832<br>H 2.39414 -3.21990 0.84174<br>O -0.11465 -0.11873 -2.00055<br>O 1.16078 -0.78273 -1.99530<br>H 0.57939 -3.03313 -1.12800										
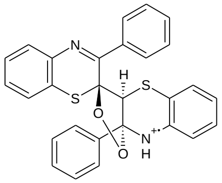<br><br>S -0.52250 -2.36475 -1.99714<br>C 0.25181 -0.97843 -1.08469<br>C 1.06929 -1.57867 0.06119<br>N 0.51254 -2.39504 0.88336<br>C -1.40753 -3.51248 1.77816<br>C -2.60040 -4.19939 1.61389<br>C -3.14636 -4.35611 0.33554	-2133.27222	-2132.82877	-2132.91361	-2133.30226	-2132.94365	-2134.52574	-2134.16713	-2134.92221	-2134.56360	15.3

C -2.51527 -3.79650 -0.76967<br>C -1.33240 -3.07118 -0.60333<br>C -0.74967 -2.93993 0.67716<br>C 2.49066 -1.22793 0.28398<br>C 2.91992 -0.98713 1.59660<br>C 4.26173 -0.73616 1.86332<br>C 5.19463 -0.74450 0.82606<br>C 4.77619 -0.99802 -0.47962<br>C 3.43122 -1.22870 -0.75508<br>H -0.94920 -3.40784 2.75703<br>H -3.10018 -4.62966 2.47613<br>H -4.07198 -4.90712 0.20046<br>H -2.94864 -3.90186 -1.75996<br>H 2.19053 -0.99307 2.40072<br>H 4.57960 -0.53659 2.88257<br>H 6.24377 -0.55596 1.03493<br>H 5.49915 -1.01861 -1.28984<br>H 3.12478 -1.43536 -1.77489<br>S -1.54439 0.16503 0.94724<br>C -0.78041 0.08982 -0.67503<br>C -0.10507 1.39406 -1.11402<br>N -1.17744 2.34151 -1.32966<br>C -3.14246 3.54191 -0.75229<br>C -4.20150 3.75847 0.08377<br>C -4.43690 2.89780 1.18626<br>C -3.61521 1.81510 1.41810<br>C -2.52826 1.54446 0.57149<br>C -2.25902 2.44801 -0.53007<br>C 1.02026 2.05015 -0.32242<br>C 0.78556 2.62491 0.93347<br>C 1.81267 3.27109 1.61406<br>C 3.08052 3.37018 1.04290<br>C 3.31216 2.82296 -0.21639<br>C 2.29012 2.16636 -0.89680<br>H -1.61331 -0.03109 -1.37670<br>H -2.95021 4.20133 -1.59303<br>H -4.86527 4.59750 -0.09476<br>H -5.27976 3.08279 1.84346<br>H -3.81325 1.14645 2.24986<br>H -0.19421 2.59191 1.39604<br>H 1.61515 3.70635 2.58888<br>H 3.87933 3.88010 1.57325<br>H 4.29250 2.90310 -0.67622<br>H 2.48338 1.74372 -1.87526<br>O 1.06323 -0.26640 -2.01674<br>O 0.35089 0.93654 -2.38652<br>H -0.96831 3.13208 -1.93321										

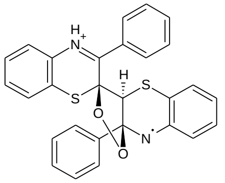<br>S 0.50516 2.36928 1.61360<br>C 0.43637 0.65430 0.93007<br>C 1.82757 0.40135 0.40007<br>N 2.26824 1.25417 -0.49940<br>C 2.08192 3.11264 -2.03407<br>C 1.63475 4.39720 -2.30233<br>C 0.83595 5.07029 -1.37337<br>C 0.46504 4.45782 -0.18145<br>C 0.89814 3.16012 0.09786<br>C 1.72017 2.50118 -0.83038<br>C 2.71120 -0.66949 0.83253<br>C 2.23114 -1.94905 1.17163<br>C 3.12566 -2.95426 1.51178<br>C 4.49630 -2.69809 1.54518<br>C 4.98039 -1.42571 1.23674<br>C 4.09858 -0.41735 0.87969<br>H 2.70615 2.58182 -2.74671<br>H 1.90893 4.87647 -3.23595<br>H 0.48825 6.07620 -1.58534<br>H -0.16869 4.97647 0.53054<br>H 1.17073 -2.16134 1.15078<br>H 2.74928 -3.94271 1.75447<br>H 5.18807 -3.48713 1.82383<br>H 6.04359 -1.21582 1.28981<br>H 4.48349 0.58158 0.69595<br>S -0.51005 0.02310 -1.77252<br>C -0.77680 0.46652 -0.04732<br>C -1.66391 -0.53422 0.74212<br>N -1.43697 -1.92960 0.55401<br>C -0.74178 -3.87416 -0.56088<br>C -0.21238 -4.52857 -1.64654<br>C 0.22722 -3.79056 -2.76211<br>C 0.14157 -2.39965 -2.76821<br>C -0.39422 -1.71771 -1.67913<br>C -0.87600 -2.44526 -0.53535<br>C -3.14524 -0.19331 0.66419<br>C -3.61381 0.99201 1.24120<br>C -4.96389 1.32063 1.15874<br>C -5.85187 0.47571 0.49223<br>C -5.38429 -0.70188 -0.08646<br>C -4.03472 -1.04076 0.00338<br>H -1.30124 1.42194 -0.10344<br>H -1.09241 -4.41243 0.31432	-2133.28973	-2132.84629	-2132.93188	-2133.32068	-2132.96283	-2134.53918	-2134.18133	-2134.93507	-2134.57721	6.7

H -0.13133 -5.61079 -1.64553<br>H 0.64910 -4.30164 -3.62168<br>H 0.50259 -1.84083 -3.62718<br>H -2.92785 1.65400 1.76231<br>H -5.32157 2.23869 1.61547<br>H -6.90437 0.73542 0.42585<br>H -6.06945 -1.36457 -0.60676<br>H -3.67936 -1.96582 -0.43859<br>O 0.17378 -0.20425 2.00627<br>O -1.26083 -0.22390 2.08982<br>H 3.14344 1.01729 -0.95936										
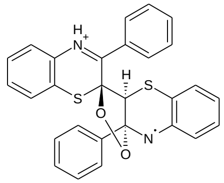<br>S -0.54243 -2.39447 -1.99763<br>C 0.20258 -0.96487 -1.11359<br>C 1.06009 -1.53578 -0.00085<br>N 0.47892 -2.38250 0.81235<br>C -1.38349 -3.57627 1.78552<br>C -2.57027 -4.27538 1.62499<br>C -3.14473 -4.39497 0.35700<br>C -2.54685 -3.80699 -0.75276<br>C -1.35862 -3.08934 -0.60526<br>C -0.78186 -2.98963 0.67030<br>C 2.45310 -1.18168 0.25362<br>C 2.86471 -0.96206 1.58094<br>C 4.20105 -0.70876 1.85863<br>C 5.13862 -0.69864 0.82579<br>C 4.73503 -0.92455 -0.49008<br>C 3.39717 -1.14929 -0.78657<br>H -0.92406 -3.47540 2.76442<br>H -3.04779 -4.72809 2.48722<br>H -4.07364 -4.94206 0.23219<br>H -3.00463 -3.89225 -1.73300<br>H 2.13501 -0.93682 2.38608<br>H 4.50867 -0.51376 2.88090<br>H 6.18475 -0.50943 1.04643<br>H 5.46556 -0.92412 -1.29269<br>H 3.09638 -1.33441 -1.81055<br>S -1.54027 0.20920 0.95938<br>C -0.82637 0.09866 -0.68888<br>C -0.16688 1.41104 -1.14098<br>N -1.15178 2.40040 -1.41339<br>C -3.06272 3.61050 -0.79177<br>C -4.10530 3.90129 0.05656	-<br>2133.27030	-<br>2132.82707	-<br>2132.91119	-<br>2133.30095	-<br>2132.94184	-<br>2134.52348	-<br>2134.16437	-<br>2134.91946	-<br>2134.56035	17.3

C -4.33183 3.09389 1.18401<br>C -3.52681 1.98291 1.42966<br>C -2.46857 1.66457 0.58285<br>C -2.17807 2.51078 -0.55481<br>C 0.97425 2.04156 -0.33555<br>C 0.75522 2.61735 0.92246<br>C 1.79331 3.24893 1.60307<br>C 3.06050 3.33691 1.03062<br>C 3.27853 2.79250 -0.23267<br>C 2.24443 2.15216 -0.91128<br>H -1.66661 -0.03807 -1.37905<br>H -2.85227 4.22270 -1.66325<br>H -4.74878 4.75223 -0.14207<br>H -5.15040 3.31717 1.86085<br>H -3.73738 1.34502 2.28367<br>H -0.22545 2.59890 1.38206<br>H 1.60209 3.68471 2.57937<br>H 3.86712 3.83611 1.55976<br>H 4.25725 2.86411 -0.69815<br>H 2.42890 1.73872 -1.89557<br>O 1.00652 -0.27842 -2.05723<br>O 0.32191 0.93888 -2.41384<br>H 1.05370 -2.73882 1.57365										
**Single-bond dimer, dioxane radical**										
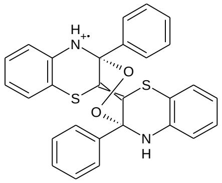<br>S -1.98512 -0.28447 1.52140<br>C -0.59992 0.39520 0.84854<br>C -0.63123 1.38400 -0.29458<br>N -1.85482 2.06807 -0.44135<br>C -4.24193 2.16249 -0.76609<br>C -5.51858 1.71702 -0.45418<br>C -5.71204 0.66346 0.44531<br>C -4.61077 0.06087 1.03267<br>C -3.32112 0.50730 0.71757<br>C -3.11794 1.56502 -0.18138<br>C 0.50126 2.38860 -0.35904<br>C 1.34855 2.47050 -1.46311<br>C 2.33370 3.45709 -1.50643<br>C 2.47172 4.36079 -0.45525<br>C 1.61507 4.28533 0.64382<br>C 0.62962 3.30456 0.69111<br>H -4.10193 2.98093 -1.46664<br>H -6.37383 2.19469 -0.92203	-<br>2133.25477			-<br>2133.28083		-<br>2134.49633		-<br>2134.89450		

H -6.71163 0.31483 0.68122<br>H -4.73848 -0.75986 1.73280<br>H 1.24272 1.77049 -2.28350<br>H 2.99266 3.51724 -2.36750<br>H 3.24146 5.12609 -0.49288<br>H 1.71215 4.99121 1.46319<br>H -0.04211 3.24840 1.54391<br>S 1.98514 0.28422 1.52131<br>C 0.60001 -0.39494 0.84818<br>C 0.63114 -1.38375 -0.29494<br>N 1.85477 -2.06777 -0.44197<br>C 4.24189 -2.16242 -0.76648<br>C 5.51853 -1.71719 -0.45424<br>C 5.71203 -0.66386 0.44554<br>C 4.61078 -0.06126 1.03290<br>C 3.32112 -0.50749 0.71750<br>C 3.11789 -1.56496 -0.18176<br>C -0.50123 -2.38847 -0.35926<br>C -1.34904 -2.47009 -1.46294<br>C -2.33414 -3.45674 -1.50608<br>C -2.47159 -4.36077 -0.45510<br>C -1.61441 -4.28559 0.64357<br>C -0.62901 -3.30477 0.69068<br>H 4.10189 -2.98068 -1.46723<br>H 6.37380 -2.19484 -0.92207<br>H 6.71165 -0.31544 0.68162<br>H 4.73848 0.75929 1.73325<br>H -1.24366 -1.76984 -2.28317<br>H -2.99350 -3.51668 -2.36685<br>H -3.24133 -5.12608 -0.49259<br>H -1.71103 -4.99175 1.46277<br>H 0.04317 -3.24885 1.54314<br>H 1.81405 -2.85863 -1.07043<br>O 0.59018 -0.39897 -1.43234<br>O -0.59048 0.39932 -1.43224<br>H -1.81418 2.85903 -1.06969										
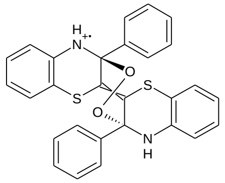<br>S -1.65135 -1.47044 -1.36946<br>C -0.66834 -0.54348 -0.28798<br>C -1.41426 0.38437 0.64805<br>N -2.26167 -0.46918 1.46554<br>C -4.26281 -1.78247 1.70722<br>C -5.25143 -2.53557 1.11924	-<br>2133.30064	-<br>2132.85694	-<br>2132.94224	-<br>2133.32908	-<br>2132.97068	-<br>2134.54846	-<br>2134.19006	-<br>2134.94676	-<br>2134.58836	0.0

C -5.20541 -2.84741 -0.25784<br>C -4.13926 -2.43276 -1.02687<br>C -3.09940 -1.69012 -0.43907<br>C -3.18619 -1.29815 0.92913<br>C -2.18235 1.49585 -0.06033<br>C -1.61264 2.11577 -1.17587<br>C -2.26877 3.17361 -1.79664<br>C -3.49580 3.61995 -1.30502<br>C -4.06106 3.00793 -0.18860<br>C -3.40640 1.94706 0.43543<br>H -4.31564 -1.50840 2.75645<br>H -6.08799 -2.87842 1.71945<br>H -5.99949 -3.43319 -0.70802<br>H -4.07536 -2.70633 -2.07570<br>H -0.65600 1.77557 -1.56182<br>H -1.82242 3.64784 -2.66539<br>H -4.00952 4.44329 -1.79247<br>H -5.01443 3.35252 0.20014<br>H -3.85744 1.47915 1.30474<br>S 1.53952 -1.74213 -1.29154<br>C 0.69001 -0.68530 -0.23036<br>C 1.42662 0.06614 0.87463<br>N 2.40761 -0.81547 1.46972<br>C 4.72895 -1.46918 1.17695<br>C 5.73217 -2.01181 0.38609<br>C 5.46928 -2.40425 -0.93012<br>C 4.18813 -2.26342 -1.44339<br>C 3.17044 -1.73624 -0.64054<br>C 3.42976 -1.32086 0.67484<br>C 1.99348 1.41744 0.45892<br>C 2.34682 2.32870 1.46098<br>C 2.93184 3.54483 1.12607<br>C 3.17561 3.86206 -0.21177<br>C 2.82810 2.95707 -1.21034<br>C 2.23775 1.73734 -0.87660<br>H 4.94214 -1.15010 2.19361<br>H 6.73236 -2.11619 0.79506<br>H 6.25840 -2.81464 -1.55123<br>H 3.96649 -2.56865 -2.46210<br>H 2.14917 2.09380 2.50329<br>H 3.19441 4.24872 1.91030<br>H 3.63189 4.81265 -0.47180<br>H 3.01174 3.19579 -2.25367<br>H 1.96989 1.04572 -1.67028<br>H 2.73830 -0.44252 2.35363<br>O 0.49598 0.20398 1.95488<br>O -0.52735 1.08896 1.48935<br>H -2.30679 -0.24910 2.45407										
**Single-bond dimer, C2-hydroperoxylalkyl radical**										

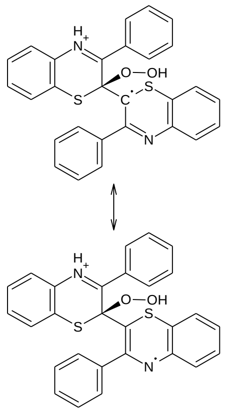<br>S 1.88905 0.68927 -1.47678<br>C 0.84202 0.32413 -0.01105<br>C 1.21643 -1.00930 0.64802<br>N 2.43944 -1.46899 0.59219<br>C 4.79429 -1.60906 0.16443<br>C 5.91976 -1.19217 -0.52655<br>C 5.81185 -0.18230 -1.48785<br>C 4.58579 0.41480 -1.75440<br>C 3.44362 0.00167 -1.06346<br>C 3.55800 -1.01470 -0.11109<br>C 0.25749 -1.74012 1.47535<br>C -0.67731 -1.05538 2.27057<br>C -1.54699 -1.77221 3.08301<br>C -1.50772 -3.16639 3.10125<br>C -0.58914 -3.85142 2.30535<br>C 0.29284 -3.14583 1.49780<br>H 4.86369 -2.38782 0.91884<br>H 6.88045 -1.64966 -0.31687<br>H 6.69237 0.14690 -2.03019<br>H 4.50890 1.20337 -2.49654<br>H -0.69117 0.02824 2.28834<br>H -2.25383 -1.23812 3.71000<br>H -2.19600 -3.71981 3.73271<br>H -0.56849 -4.93641 2.30235<br>H 0.96740 -3.69207 0.84341<br>C -0.58335 0.30403 -0.53187<br>S -0.85463 -1.09444 -1.53362<br>C -2.59675 -1.19934 -1.52847<br>C -3.37952 -0.13868 -1.01542<br>N -2.89026 1.04302 -0.52874<br>C -1.59697 1.25700 -0.30779<br>C -3.19084 -2.34320 -2.07177<br>C -4.57370 -2.45559 -2.08980<br>C -5.37090 -1.42645 -1.56781	-<br>2133.28862	-<br>2132.84576	-<br>2132.93500	-<br>2133.32039	-<br>2132.96676	-<br>2134.53955	-<br>2134.18592	-<br>2134.93758	-<br>2134.58396	9.5

C -4.78351 -0.28932 -1.04555<br>C -1.30582 2.62895 0.20006<br>C -0.43493 3.47746 -0.49109<br>C -0.23111 4.78306 -0.05127<br>C -0.88798 5.24836 1.08787<br>C -1.76330 4.40772 1.77605<br>C -1.98154 3.10785 1.32675<br>H -2.57028 -3.14142 -2.46921<br>H -5.03376 -3.34564 -2.50725<br>H -6.45242 -1.51938 -1.58116<br>H -5.38246 0.52486 -0.64916<br>H 0.07221 3.12372 -1.38449<br>H 0.43834 5.43821 -0.60127<br>H -0.72390 6.26490 1.43379<br>H -2.28152 4.76657 2.66071<br>H -2.67312 2.45445 1.85069<br>O 0.92443 1.30382 1.00372<br>O 2.26140 1.32905 1.49653<br>H 2.57715 2.17916 1.14696<br>H 2.62073 -2.28021 1.18118										
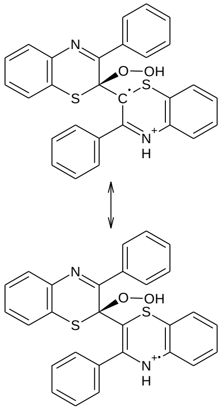<br>S 1.14639 -2.19511 0.34012<br>C 0.46463 -0.87148 -0.72908<br>C 1.35671 0.37208 -0.85997<br>N 2.61394 0.39836 -0.61221<br>C 4.75900 -0.41169 -0.00661<br>C 5.61083 -1.37901 0.50526<br>C 5.08824 -2.59727 0.94829<br>C 3.72151 -2.84301 0.87199<br>C 2.86783 -1.86689 0.35136<br>C 3.37414 -0.63400 -0.09082<br>C 0.76339 1.60077 -1.45255<br>C -0.40117 1.59806 -2.23480<br>C -0.89430 2.78380 -2.77622<br>C -0.23545 3.98961 -2.54919	-2133.30164	-2132.85838	-2132.94677	-2133.33090	-2132.97603	-2134.55554	-2134.20067	-2134.95405	-2134.59917	0.0

C 0.92902 4.00244 -1.77865<br>C 1.42214 2.82242 -1.23788<br>H 5.13707 0.54371 -0.35811<br>H 6.67797 -1.18891 0.56190<br>H 5.74566 -3.36126 1.35199<br>H 3.31698 -3.79233 1.21234<br>H -0.91420 0.67164 -2.46397<br>H -1.79183 2.75793 -3.38724<br>H -0.62249 4.91241 -2.97167<br>H 1.44973 4.93750 -1.59289<br>H 2.32012 2.82959 -0.62949<br>C -0.94700 -0.66232 -0.19543<br>S -2.08440 -1.74133 -0.94496<br>C -3.58492 -1.36760 -0.14359<br>C -3.68413 -0.38158 0.85159<br>N -2.56712 0.31189 1.26829<br>C -1.28882 0.20679 0.82986<br>C -4.72276 -2.08152 -0.53574<br>C -5.94344 -1.80905 0.06062<br>C -6.04191 -0.82134 1.05052<br>C -4.92335 -0.11061 1.44671<br>C -0.36735 1.12684 1.54467<br>C -0.65539 2.49927 1.52311<br>C 0.12744 3.38583 2.25443<br>C 1.19113 2.91054 3.02053<br>C 1.46774 1.54480 3.05741<br>C 0.69255 0.65075 2.32435<br>H -4.64302 -2.84292 -1.30555<br>H -6.82456 -2.36337 -0.24440<br>H -7.00029 -0.60988 1.51269<br>H -4.99460 0.65441 2.21396<br>H -1.47280 2.87387 0.91275<br>H -0.09162 4.44855 2.22063<br>H 1.80083 3.60372 3.59234<br>H 2.28467 1.16875 3.66561<br>H 0.89647 -0.41309 2.37412<br>O 0.23908 -1.37606 -2.05533<br>O 1.47831 -1.75016 -2.62715<br>H 1.49568 -2.70789 -2.45901<br>H -2.70608 0.95894 2.03756										
**Single-bond dimer, C3-hydroperoxylalkyl radical**										
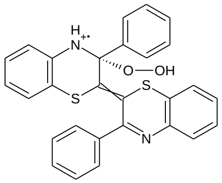	-2133.28616	-2132.84361	-2132.93291	-2133.31398	-2132.96073	-2134.53273	-2134.17947	-2134.93316	-2134.57991	0.0

S 1.76714 -1.14799 1.01775<br>C 0.92268 0.22849 0.37361<br>C 1.74093 1.35385 -0.09493<br>N 2.93191 1.22558 -0.59641<br>C 4.85672 0.01683 -1.30787<br>C 5.69761 -1.07271 -1.19427<br>C 5.36801 -2.13782 -0.33673<br>C 4.18190 -2.12712 0.37491<br>C 3.30064 -1.04523 0.22136<br>C 3.64622 0.06125 -0.58919<br>C 1.24624 2.74283 0.05010<br>C 0.59227 3.14304 1.22400<br>C 0.19996 4.46772 1.38393<br>C 0.44365 5.39775 0.37234<br>C 1.09912 5.00359 -0.79497<br>C 1.51020 3.68434 -0.95267<br>H 5.11163 0.87007 -1.92828<br>H 6.62880 -1.09812 -1.75101<br>H 6.04310 -2.98153 -0.23617<br>H 3.91703 -2.95899 1.02074<br>H 0.39849 2.42024 2.01253<br>H -0.29535 4.77527 2.29997<br>H 0.12671 6.42921 0.49558<br>H 1.29098 5.72578 -1.58290<br>H 2.02499 3.36879 -1.85469<br>S -1.29181 1.35798 -0.63958<br>C -0.47380 0.17036 0.28292<br>C -1.23581 -0.99290 0.92569<br>N -2.63573 -0.67682 1.15624<br>C -4.82280 -0.42265 0.14274<br>C -5.63966 0.19522 -0.79141<br>C -5.11982 1.12731 -1.70040<br>C -3.77326 1.44305 -1.65891<br>C -2.94511 0.82305 -0.71044<br>C -3.45598 -0.11671 0.19725<br>C -1.06706 -2.31037 0.17274<br>C -1.23323 -3.52120 0.85531<br>C -1.17417 -4.72507 0.15910<br>C -0.95973 -4.73105 -1.21914<br>C -0.80412 -3.52585 -1.90029<br>C -0.85749 -2.31758 -1.20815<br>H -5.23273 -1.14817 0.83978<br>H -6.69510 -0.05701 -0.82052<br>H -5.76472 1.59899 -2.43385<br>H -3.35179 2.16615 -2.35123<br>H -1.39845 -3.52708 1.92822<br>H -1.29485 -5.66068 0.69696<br>H -0.91504 -5.67194 -1.75948<br>H -0.63820 -3.52113 -2.97331<br>H -0.72486 -1.38740 -1.75371										

O -0.69257 -1.22649 2.22377<br>O -0.75568 -0.02788 2.98948<br>H -1.70952 0.06215 3.16522<br>H -3.07843 -1.44526 1.65080										
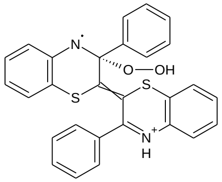<br>S 1.98428 -0.71521 1.38087<br>C 0.88663 0.31879 0.42513<br>C 1.45160 1.61033 0.06038<br>N 2.72207 1.69594 -0.27255<br>C 4.79877 0.80411 -1.13057<br>C 5.74025 -0.21609 -1.16653<br>C 5.52081 -1.39220 -0.44731<br>C 4.35913 -1.55811 0.30187<br>C 3.40608 -0.53980 0.34735<br>C 3.63898 0.63736 -0.37251<br>C 0.69043 2.86099 0.15561<br>C -0.24837 3.01681 1.18839<br>C -0.92042 4.22361 1.33089<br>C -0.67683 5.27269 0.44317<br>C 0.24823 5.11717 -0.58983<br>C 0.93708 3.91933 -0.73298<br>H 4.95456 1.72358 -1.68749<br>H 6.64246 -0.09381 -1.75628<br>H 6.25508 -2.19106 -0.47451<br>H 4.18786 -2.47779 0.85271<br>H -0.43337 2.20015 1.88027<br>H -1.63517 4.34739 2.13836<br>H -1.21152 6.21121 0.55367<br>H 0.42525 5.92570 -1.29181<br>H 1.62264 3.79495 -1.56728<br>S -1.16332 0.73167 -1.23869<br>C -0.38809 -0.05523 0.09728<br>C -1.08558 -1.24087 0.75563<br>N -2.51331 -1.14805 0.89425<br>C -4.70924 -0.56683 0.28851<br>C -5.60717 0.06599 -0.53902<br>C -5.14310 0.86522 -1.59986<br>C -3.77551 1.04296 -1.80718<br>C -2.85716 0.41377 -0.97394<br>C -3.29600 -0.44623 0.08514<br>C -0.75526 -2.58146 0.10134<br>C -1.24702 -3.74172 0.71251<br>C -0.99530 -4.98779 0.14713	-2133.27929	-2132.83687	-2132.92611	-2133.30956	-2132.95638	-2134.52711	-2134.17392	-2134.92579	-2134.57261	4.6

C -0.25843 -5.08565 -1.03372<br>C 0.22920 -3.93173 -1.64358<br>C -0.01674 -2.68120 -1.07898<br>H -5.03598 -1.20172 1.10607<br>H -6.67365 -0.05376 -0.37808<br>H -5.84925 1.36338 -2.25627<br>H -3.42891 1.68698 -2.61047<br>H -1.82281 -3.66176 1.62839<br>H -1.37630 -5.88339 0.62896<br>H -0.06457 -6.05849 -1.47608<br>H 0.80612 -4.00021 -2.56106<br>H 0.37474 -1.79341 -1.56530<br>O -0.58615 -1.36738 2.09713<br>O -1.04175 -0.24971 2.86021<br>H -1.96899 -0.49403 3.02853<br>H 3.09650 2.62863 -0.42374										
**Single-bond dimer, C2-hydroperoxide**										
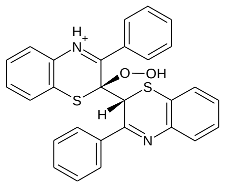<br>S 0.64489 -1.27858 -0.99965<br>C 0.17244 0.41356 -0.51420<br>C 1.34494 1.26506 -0.03120<br>N 2.57235 1.00596 -0.39021<br>C 4.44579 0.03458 -1.52845<br>C 4.99470 -0.98226 -2.29108<br>C 4.19975 -2.06554 -2.67971<br>C 2.86206 -2.13347 -2.31121<br>C 2.29749 -1.11377 -1.53981<br>C 3.09954 -0.03781 -1.15383<br>C 1.13819 2.48391 0.76081<br>C 0.14438 3.41443 0.41402<br>C 0.01918 4.59120 1.14266<br>C 0.86599 4.84630 2.22107<br>C 1.85121 3.92315 2.57098<br>C 1.99449 2.74850 1.84351<br>H 5.04685 0.88642 -1.22242<br>H 6.03713 -0.93269 -2.58590<br>H 4.62587 -2.86314 -3.27975<br>H 2.24989 -2.97548 -2.61905<br>H -0.49072 3.23884 -0.44753<br>H -0.73818 5.31524 0.85961<br>H 0.75736 5.76457 2.78997<br>H 2.50289 4.11179 3.41805<br>H 2.73524 2.01326 2.14636	-2133.92148	-2133.46528	-2133.55350	-2133.95502	-2133.58704	-2135.17929	-2134.81131	-2135.57731	-2135.20933	2.0

C -0.93763 0.32032 0.57910<br>S -0.29198 -0.07124 2.22698<br>C -0.34378 -1.82820 2.18126<br>C -1.26650 -2.48607 1.34801<br>N -2.19032 -1.83040 0.54042<br>C -2.10276 -0.59441 0.20266<br>C 0.49303 -2.55816 3.02596<br>C 0.42900 -3.94910 3.03567<br>C -0.46655 -4.61598 2.19700<br>C -1.31141 -3.88712 1.36949<br>C -3.19762 -0.02940 -0.62132<br>C -3.90925 -0.88745 -1.47511<br>C -4.95779 -0.40720 -2.24977<br>C -5.32448 0.93806 -2.17760<br>C -4.63060 1.79719 -1.32811<br>C -3.56929 1.32103 -0.56196<br>H 1.19858 -2.03868 3.66847<br>H 1.08428 -4.51027 3.69493<br>H -0.51232 -5.70059 2.19808<br>H -2.02777 -4.38286 0.72098<br>H -3.61865 -1.93168 -1.52576<br>H -5.48893 -1.08213 -2.91464<br>H -6.14576 1.31363 -2.78094<br>H -4.91495 2.84279 -1.25722<br>H -3.06350 2.01390 0.10358<br>O -0.45576 1.11874 -1.57262<br>O 0.49400 1.31208 -2.61851<br>H 0.18897 0.66997 -3.28137<br>H -1.31886 1.33624 0.68952<br>H 3.25876 1.70444 -0.10774										
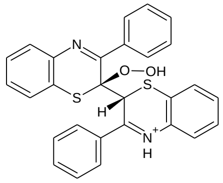<br>S 0.67182 -2.18165 -1.69338<br>C 0.01553 -0.66237 -0.89995<br>C 1.09594 0.42235 -1.01902<br>N 2.31702 0.18860 -0.68444<br>C 4.00043 -1.15773 0.35242<br>C 4.59176 -2.38718 0.60368<br>C 3.99658 -3.55851 0.12521<br>C 2.79478 -3.50023 -0.57224<br>C 2.17947 -2.26562 -0.79814<br>C 2.79068 -1.07294 -0.35604<br>C 0.78309 1.78036 -1.53188<br>C -0.46693 2.40800 -1.39306	-2133.92288	-2133.46689	-2133.55369	-2133.95732	-2133.58812	-2135.18402	-2134.81482	-2135.58165	-2135.21246	0.1

C -0.66872 3.69922 -1.87783<br>C 0.36554 4.38281 -2.51199<br>C 1.61008 3.76788 -2.65927<br>C 1.81653 2.48274 -2.17519<br>H 4.46107 -0.23295 0.68683<br>H 5.52421 -2.43639 1.15729<br>H 4.46330 -4.52201 0.30567<br>H 2.32129 -4.41108 -0.92692<br>H -1.29148 1.90337 -0.90293<br>H -1.64007 4.16853 -1.75460<br>H 0.20300 5.38638 -2.89366<br>H 2.42005 4.28955 -3.16044<br>H 2.77998 1.99826 -2.29322<br>C -0.33951 -0.96576 0.60159<br>S -1.47574 -2.35355 0.85871<br>C -2.99837 -1.54192 0.54303<br>C -3.14794 -0.17942 0.83394<br>N -2.07687 0.54134 1.38518<br>C -0.80287 0.23248 1.38082<br>C -4.10443 -2.25835 0.08482<br>C -5.32737 -1.61705 -0.08545<br>C -5.46245 -0.25491 0.19326<br>C -4.37288 0.46724 0.65747<br>C 0.13824 1.08736 2.09502<br>C 1.29428 0.53788 2.67832<br>C 2.16746 1.34800 3.39154<br>C 1.91689 2.71412 3.51197<br>C 0.78034 3.27243 2.92421<br>C -0.10820 2.46832 2.22699<br>H -4.00211 -3.31455 -0.14332<br>H -6.17955 -2.18428 -0.44592<br>H -6.41549 0.24262 0.04965<br>H -4.46203 1.52678 0.87899<br>H 1.49903 -0.52610 2.62271<br>H 3.04547 0.90964 3.85451<br>H 2.60941 3.34621 4.05912<br>H 0.59324 4.33859 2.99863<br>H -0.96074 2.93391 1.74044<br>O -1.21597 -0.35372 -1.49954<br>O -1.07237 -0.22714 -2.91030<br>H -0.96302 0.73572 -3.01697<br>H 0.60067 -1.30716 1.04122<br>H -2.34561 1.36027 1.92563										

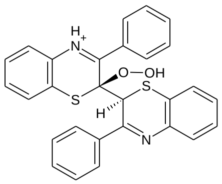<br>S 1.21626 -1.04251 1.58502<br>C -0.16693 -0.40122 0.53437<br>C -0.90839 -1.64595 0.05023<br>N -0.19537 -2.55974 -0.57202<br>C 1.78485 -3.52016 -1.56676<br>C 3.15233 -3.74253 -1.52829<br>C 3.93129 -3.13909 -0.53649<br>C 3.35331 -2.29553 0.40482<br>C 1.97782 -2.05153 0.37176<br>C 1.20038 -2.68446 -0.61055<br>C -2.32441 -1.90699 0.25454<br>C -3.28543 -0.88060 0.21454<br>C -4.63025 -1.18848 0.36206<br>C -5.03366 -2.50754 0.57341<br>C -4.08545 -3.52917 0.64040<br>C -2.73922 -3.23550 0.48018<br>H 1.16547 -3.98937 -2.32544<br>H 3.61253 -4.38839 -2.26826<br>H 5.00161 -3.31591 -0.50520<br>H 3.96507 -1.81217 1.15952<br>H -2.98396 0.14498 0.04608<br>H -5.36802 -0.39402 0.31268<br>H -6.08730 -2.73839 0.69805<br>H -4.39356 -4.55179 0.83217<br>H -2.00695 -4.03076 0.58869<br>C 0.34739 0.46851 -0.65141<br>S -0.89339 0.78261 -1.93754<br>C -1.74368 2.12707 -1.18307<br>C -1.06106 2.96576 -0.28013<br>N 0.29658 2.85823 -0.00674<br>C 0.97448 1.78596 -0.20144<br>C -3.07345 2.38599 -1.52293<br>C -3.74440 3.45720 -0.93970<br>C -3.09059 4.27303 -0.01323<br>C -1.75887 4.03536 0.29973<br>C 2.43633 1.84407 0.03048<br>C 3.34492 1.07894 -0.71327<br>C 4.71576 1.20298 -0.49792<br>C 5.19752 2.07701 0.47409<br>C 4.29974 2.83952 1.22425<br>C 2.93321 2.73093 0.99907<br>H -3.58433 1.74238 -2.23402<br>H -4.77939 3.64952 -1.20566	-2133.92238	-2133.46613	-2133.55294	-2133.95564	-2133.58620	-2135.18267	-2134.81323	-2135.58041	-2135.21097	1.0

H -3.61567 5.10291 0.44960<br>H -1.22270 4.67608 0.99369<br>H 3.00227 0.40438 -1.49235<br>H 5.40651 0.61442 -1.09443<br>H 6.26621 2.16480 0.64683<br>H 4.66725 3.51922 1.98758<br>H 2.23002 3.32249 1.57624<br>O -0.95486 0.44403 1.30881<br>O -1.36083 -0.22674 2.50592<br>H -1.03122 0.39451 3.17609<br>H 1.10095 -0.13196 -1.16509<br>H -0.71497 -3.31355 -1.01554										
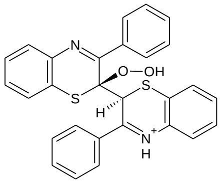<br>S 1.22683 -1.01732 1.58519<br>C -0.17739 -0.45503 0.53314<br>C -0.86619 -1.72576 0.00944<br>N -0.18893 -2.65642 -0.56620<br>C 1.82813 -3.49527 -1.54944<br>C 3.20231 -3.68274 -1.52825<br>C 3.97315 -3.08489 -0.52640<br>C 3.37344 -2.26807 0.42592<br>C 1.99410 -2.04538 0.38411<br>C 1.19851 -2.68337 -0.59211<br>C -2.31859 -1.95959 0.20240<br>C -3.28606 -0.94145 0.18754<br>C -4.63445 -1.24740 0.36256<br>C -5.03876 -2.56635 0.55708<br>C -4.08501 -3.58519 0.57471<br>C -2.73950 -3.28536 0.40060<br>H 1.20603 -3.98101 -2.29528<br>H 3.67453 -4.30934 -2.27846<br>H 5.04662 -3.24435 -0.49388<br>H 3.97445 -1.78458 1.19047<br>H -2.99859 0.08952 0.02083<br>H -5.36909 -0.44797 0.34036<br>H -6.09005 -2.79963 0.69772<br>H -4.39001 -4.61534 0.73342<br>H -1.99298 -4.07249 0.42328<br>C 0.33064 0.43291 -0.64749<br>S -0.89342 0.79358 -1.94209<br>C -1.76338 2.10428 -1.16087<br>C -1.11057 2.94821 -0.25262<br>N 0.25660 2.76911 0.01324	-<br>2133.92422	-<br>2133.46797	-<br>2133.55456	-<br>2133.95684	-<br>2133.58718	-<br>2135.18428	-<br>2134.81461	-<br>2135.58224	-<br>2135.21257	0.0

C 0.99106 1.70623 -0.19041<br>C -3.09073 2.37453 -1.49814<br>C -3.74890 3.45765 -0.92340<br>C -3.09498 4.28087 -0.00458<br>C -1.77118 4.03150 0.32864<br>C 2.42650 1.78952 0.04944<br>C 3.32350 1.08205 -0.76851<br>C 4.69259 1.22069 -0.57846<br>C 5.18062 2.03580 0.44149<br>C 4.29502 2.72454 1.27291<br>C 2.92705 2.61296 1.07588<br>H -3.60533 1.73336 -2.20727<br>H -4.78195 3.65514 -1.19118<br>H -3.61466 5.11805 0.44873<br>H -1.24765 4.66708 1.03689<br>H 2.96513 0.46131 -1.58274<br>H 5.37897 0.68896 -1.22935<br>H 6.25130 2.13022 0.59424<br>H 4.67172 3.34136 2.08229<br>H 2.25189 3.11945 1.75978<br>O -0.98289 0.40404 1.29843<br>O -1.36577 -0.21236 2.52243<br>H -2.23966 -0.58212 2.29740<br>H 1.07981 -0.17293 -1.16391<br>H 0.74506 3.60751 0.31972										
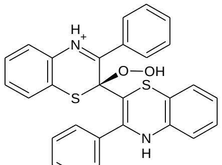<br>S 2.10731 0.78907 -1.35608<br>C 0.88732 0.36617 -0.04755<br>C 1.13614 -1.02477 0.54432<br>N 2.34392 -1.52516 0.60073<br>C 4.72646 -1.73643 0.42523<br>C 5.93835 -1.32453 -0.10519<br>C 5.97681 -0.25115 -0.99990<br>C 4.81007 0.41354 -1.35828<br>C 3.58238 0.00619 -0.83003<br>C 3.55124 -1.07369 0.05618<br>C 0.07293 -1.76860 1.21994<br>C 0.07098 -3.17415 1.16627<br>C -0.90057 -3.89674 1.84495<br>C -1.87512 -3.22973 2.58883<br>C -1.87977 -1.83676 2.64392<br>C -0.91861 -1.10200 1.95889<br>H 4.68232 -2.56553 1.12611	-<br>2133.92388	-<br>2133.46724	-<br>2133.55477	-<br>2133.95699	-<br>2133.58788	-<br>2135.18208	-<br>2134.81297	-<br>2135.58059	-<br>2135.21148	0.7

H 6.85174 -1.83619 0.17814<br>H 6.92431 0.07464 -1.41714<br>H 4.84581 1.25188 -2.04714<br>H 0.79356 -3.70283 0.54982<br>H -0.90516 -4.98026 1.78241<br>H -2.63320 -3.79665 3.12067<br>H -2.63212 -1.31644 3.22830<br>H -0.90924 -0.02113 2.03288<br>C -0.44779 0.44067 -0.73530<br>S -0.71302 -0.92399 -1.84998<br>C -2.41554 -1.23033 -1.46063<br>C -3.23182 -0.11825 -1.22285<br>N -2.66010 1.15921 -1.15826<br>C -1.40634 1.39722 -0.62135<br>C -2.95465 -2.51476 -1.47540<br>C -4.32652 -2.69184 -1.30258<br>C -5.14615 -1.58345 -1.09014<br>C -4.60351 -0.30210 -1.03343<br>C -1.27445 2.71557 0.04759<br>C -0.31325 3.63688 -0.38326<br>C -0.22960 4.89122 0.21410<br>C -1.10211 5.23490 1.24685<br>C -2.06834 4.32425 1.67361<br>C -2.16255 3.07279 1.06988<br>H -2.30404 -3.36746 -1.64608<br>H -4.75092 -3.69023 -1.33838<br>H -6.21554 -1.71425 -0.95503<br>H -5.24099 0.55711 -0.84163<br>H 0.36012 3.37319 -1.19343<br>H 0.51455 5.60314 -0.13070<br>H -1.03330 6.21309 1.71388<br>H -2.75014 4.58736 2.47689<br>H -2.91369 2.36290 1.40705<br>O 0.89235 1.27336 1.03812<br>O 2.17877 1.25177 1.65247<br>H 2.55455 2.09316 1.34371<br>H -3.31154 1.92775 -1.07881<br>H 2.43267 -2.37485 1.15534											
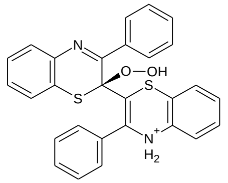	n.d.										
**Single-bond dimer, C3-hydroperoxide**											

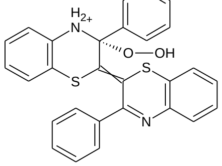<br>S 0.94811 -1.71063 1.42228<br>C 0.94733 -0.19613 0.51293<br>C 2.25630 0.08118 -0.14176<br>N 2.89474 -0.82435 -0.80098<br>C 3.01966 -2.98763 -1.81927<br>C 2.76895 -4.35147 -1.80741<br>C 2.00169 -4.91138 -0.78064<br>C 1.45788 -4.09997 0.20888<br>C 1.67754 -2.72043 0.17506<br>C 2.48615 -2.14802 -0.82753<br>C 2.88907 1.40950 0.01544<br>C 2.68311 2.16612 1.17821<br>C 3.33175 3.38759 1.34493<br>C 4.17716 3.87298 0.34929<br>C 4.38328 3.12511 -0.81281<br>C 3.75235 1.89789 -0.97619<br>H 3.64334 -2.53763 -2.58613<br>H 3.18418 -4.98510 -2.58500<br>H 1.81755 -5.98109 -0.75580<br>H 0.84372 -4.53078 0.99468<br>H 2.02907 1.79285 1.96208<br>H 3.17516 3.95912 2.25501<br>H 4.67492 4.83002 0.47594<br>H 5.03824 3.50203 -1.59304<br>H 3.90966 1.30926 -1.87442<br>S -0.06066 2.17596 -0.39075<br>C -0.13876 0.60580 0.45090<br>C -1.42008 0.22620 1.18708<br>N -2.39207 1.42696 1.13064<br>C -4.08198 1.86353 -0.63317<br>C -4.35072 2.30617 -1.92666<br>C -3.30906 2.70404 -2.76448<br>C -1.98504 2.65069 -2.33301<br>C -1.70663 2.21454 -1.03883<br>C -2.76233 1.84682 -0.21099<br>C -2.14965 -0.99125 0.65691<br>C -3.04316 -1.67493 1.48906<br>C -3.76248 -2.75706 0.98954<br>C -3.60331 -3.15228 -0.33789<br>C -2.72139 -2.46279 -1.16817<br>C -1.99375 -1.38364 -0.67446<br>H -4.88582 1.55147 0.02719<br>H -5.37652 2.33695 -2.27793	-<br>2133.90833	-<br>2133.45183	-<br>2133.53970	-<br>2133.94297	-<br>2133.57434	-<br>2135.17180	-<br>2134.80317	-<br>2135.57092	-<br>2135.20229	6.4

H -3.52649 3.04571 -3.77145<br>H -1.17367 2.93806 -2.99380<br>H -3.16819 -1.37959 2.52620<br>H -4.44656 -3.29154 1.64136<br>H -4.16619 -3.99652 -0.72465<br>H -2.59426 -2.76517 -2.20300<br>H -1.30024 -0.85852 -1.32401<br>H -3.22119 1.16914 1.67465<br>O -1.19888 0.01072 2.55147<br>O -0.56433 1.19747 3.06177<br>H -0.96778 1.24129 3.94459<br>H -1.92461 2.19403 1.63993										
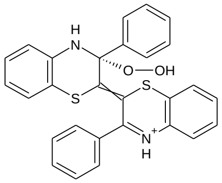<br>S 1.57107 -1.20517 1.41997<br>C 0.91761 0.19016 0.52687<br>C 1.95480 1.05798 -0.02157<br>N 3.01385 0.51131 -0.57827<br>C 4.23245 -1.28113 -1.64351<br>C 4.57381 -2.62491 -1.70578<br>C 3.98573 -3.53577 -0.82515<br>C 3.04761 -3.11190 0.11106<br>C 2.69077 -1.76425 0.17960<br>C 3.29440 -0.85740 -0.70037<br>C 1.89686 2.51481 0.06129<br>C 1.37669 3.10799 1.22438<br>C 1.38011 4.49081 1.35178<br>C 1.87405 5.28771 0.31865<br>C 2.37549 4.70248 -0.84591<br>C 2.39811 3.32084 -0.97556<br>H 4.68299 -0.56002 -2.31942<br>H 5.29807 -2.96180 -2.43970<br>H 4.25367 -4.58652 -0.87171<br>H 2.58673 -3.82408 0.78840<br>H 0.99386 2.48314 2.02638<br>H 0.99442 4.94784 2.25737<br>H 1.86299 6.36893 0.41727<br>H 2.74168 5.32470 -1.65611<br>H 2.75456 2.87542 -1.90049<br>S -1.03630 1.85027 -0.41416<br>C -0.42033 0.42661 0.40855<br>C -1.41147 -0.52670 1.09118<br>N -2.76914 0.02153 1.13752<br>C -4.68966 0.13926 -0.37535	-2133.92514	-2133.46843	-2133.55589	-2133.95743	-2133.58817	-2135.18240	-2134.81314	-2135.58174	-2135.21248	0.0

C -5.27096 0.66726 -1.52413<br>C -4.54175 1.50829 -2.36606<br>C -3.22393 1.82750 -2.05037<br>C -2.65349 1.32098 -0.88267<br>C -3.37533 0.47492 -0.03829<br>C -1.39881 -1.92684 0.49048<br>C -1.75237 -3.03089 1.27353<br>C -1.83964 -4.29500 0.69556<br>C -1.58922 -4.46607 -0.66586<br>C -1.25198 -3.36435 -1.44946<br>C -1.15761 -2.09830 -0.87489<br>H -5.25444 -0.52757 0.27106<br>H -6.29629 0.40749 -1.76934<br>H -4.99190 1.90755 -3.26926<br>H -2.64074 2.47387 -2.69998<br>H -1.94743 -2.90783 2.33420<br>H -2.10319 -5.14864 1.31323<br>H -1.65860 -5.45319 -1.11339<br>H -1.05883 -3.48660 -2.51116<br>H -0.88605 -1.24906 -1.49601<br>H -3.35897 -0.65092 1.62026<br>O -1.03695 -0.67198 2.45810<br>O -1.03327 0.61387 3.07544<br>H -1.95123 0.91302 2.92030<br>H 3.73937 1.14309 -0.90577										
**Single-bond dimer, dioxolane**										
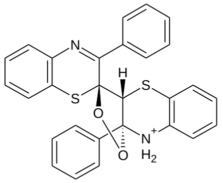<br>S -1.69362 -1.64736 -0.73646<br>C -1.12437 -0.13311 0.10596<br>C -2.15390 0.98468 -0.10907<br>N -3.41376 0.77343 0.00563<br>C -5.27731 -0.52624 0.73788<br>C -5.91814 -1.74090 0.93500<br>C -5.26411 -2.93456 0.61625<br>C -3.96315 -2.91176 0.12485<br>C -3.30788 -1.69048 -0.04517<br>C -3.96438 -0.47996 0.24584<br>C -1.70448 2.36296 -0.42801<br>C -2.19035 2.98572 -1.58349<br>C -1.81402 4.29161 -1.88663<br>C -0.96169 4.99069 -1.03246<br>C -0.48817 4.38139 0.12922<br>C -0.85212 3.07106 0.43035	-2133.91789	-2133.46025	-2133.54566	-2133.95136	-2133.57913	-2135.17601	-2134.80378	-2135.57288	-2135.20065	11.2

H -5.77346 0.41449 0.95698<br>H -6.93146 -1.76148 1.32367<br>H -5.76573 -3.88711 0.75686<br>H -3.45016 -3.83865 -0.11461<br>H -2.85819 2.43811 -2.24187<br>H -2.18858 4.76372 -2.79009<br>H -0.67141 6.01037 -1.26805<br>H 0.16025 4.92770 0.80773<br>H -0.50508 2.61656 1.35513<br>S 0.76947 -0.28165 -1.99221<br>C 0.35028 0.13648 -0.26109<br>C 1.10141 -0.63471 0.84650<br>N 1.65038 -1.95397 0.28561<br>C 3.93964 -2.36795 -0.55644<br>C 4.89348 -2.18006 -1.55279<br>C 4.59295 -1.39770 -2.66762<br>C 3.34926 -0.78540 -2.79656<br>C 2.38117 -0.97374 -1.80826<br>C 2.69583 -1.77710 -0.71477<br>C 2.20566 0.11987 1.54606<br>C 2.99112 1.05459 0.86455<br>C 4.02515 1.70919 1.52852<br>C 4.27899 1.43516 2.87107<br>C 3.49489 0.50566 3.55255<br>C 2.45968 -0.15207 2.89443<br>H 0.54843 1.19904 -0.12026<br>H 4.15853 -2.97195 0.31970<br>H 5.87013 -2.64094 -1.45291<br>H 5.34035 -1.24742 -3.44006<br>H 3.12897 -0.15793 -3.65407<br>H 2.80912 1.28479 -0.18138<br>H 4.62803 2.43686 0.99442<br>H 5.08325 1.95043 3.38756<br>H 3.68210 0.29589 4.60104<br>H 1.83870 -0.85996 3.43438<br>O -1.04063 -0.26790 1.51639<br>O 0.10613 -1.09692 1.74087<br>H 2.00277 -2.51141 1.07093<br>H 0.84710 -2.47444 -0.10309										
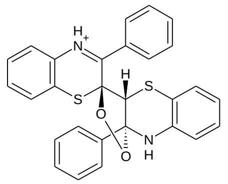<br>S 1.56336 -1.41965 0.44661<br>C 0.81208 0.10073 -0.18755<br>C 1.74435 1.26788 0.10932	-2133.93467	-2133.47780	-2133.56471	-2133.96709	-2133.59713	-2135.18854	-2134.81858	-2135.58530	-2135.21534	2.0

N 3.02639 1.12648 -0.12283<br>C 5.03845 0.10007 -0.96186<br>C 5.76450 -1.04361 -1.24916<br>C 5.19449 -2.30481 -1.04306<br>C 3.89788 -2.42835 -0.56001<br>C 3.15192 -1.28199 -0.27348<br>C 3.73476 -0.02555 -0.47094<br>C 1.30772 2.58211 0.58756<br>C 0.29353 3.28946 -0.07784<br>C -0.06020 4.55961 0.35795<br>C 0.57390 5.12284 1.46640<br>C 1.57854 4.42208 2.13170<br>C 1.95740 3.15862 1.69093<br>H 5.46722 1.08597 -1.11546<br>H 6.77490 -0.95669 -1.63368<br>H 5.76461 -3.19993 -1.27001<br>H 3.45689 -3.40873 -0.41071<br>H -0.18004 2.86649 -0.95809<br>H -0.83049 5.11169 -0.17092<br>H 0.28350 6.11048 1.81120<br>H 2.06717 4.85597 2.99813<br>H 2.72476 2.60438 2.22411<br>S -1.12274 -0.61965 1.85540<br>C -0.64194 0.23958 0.35543<br>C -1.48346 -0.09562 -0.89544<br>N -1.58735 -1.50738 -1.10060<br>C -2.02028 -3.79795 -0.51851<br>C -2.11534 -4.82273 0.41193<br>C -1.88230 -4.57170 1.76595<br>C -1.55208 -3.28333 2.16760<br>C -1.46625 -2.24326 1.23711<br>C -1.69861 -2.48783 -0.12589<br>C -2.81316 0.64351 -0.95598<br>C -3.92546 0.06305 -0.33880<br>C -5.15330 0.72037 -0.34251<br>C -5.28615 1.95470 -0.97645<br>C -4.18346 2.52914 -1.60551<br>C -2.94909 1.88185 -1.59189<br>H -0.82337 1.28874 0.59239<br>H -2.19659 -3.99972 -1.57213<br>H -2.37011 -5.82342 0.07584<br>H -1.95118 -5.36873 2.49914<br>H -1.35928 -3.07181 3.21622<br>H -3.83944 -0.90626 0.14278<br>H -6.00804 0.26136 0.14542<br>H -6.24587 2.46296 -0.98572<br>H -4.27999 3.48448 -2.11293<br>H -2.10395 2.33651 -2.09688<br>O 0.66863 0.02115 -1.60704<br>O -0.66654 0.45925 -1.92444										

H -1.98751 -1.74797 -1.99790<br>H 3.58616 1.97295 -0.03921										
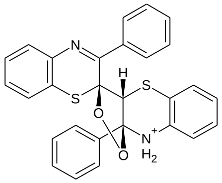<br>S -1.60971 -1.24340 -0.71006<br>C -0.96462 0.11479 0.30551<br>C -1.78970 1.40017 0.12298<br>N -3.04943 1.42205 -0.10938<br>C -5.23153 0.47436 -0.22208<br>C -6.09347 -0.59353 -0.42212<br>C -5.57630 -1.86191 -0.70400<br>C -4.20192 -2.06128 -0.76702<br>C -3.33497 -0.98647 -0.55237<br>C -3.84069 0.29570 -0.28487<br>C -1.10515 2.71194 0.28089<br>C -0.40674 3.04345 1.45087<br>C 0.18444 4.29843 1.58031<br>C 0.09199 5.22802 0.54516<br>C -0.60642 4.90463 -0.61784<br>C -1.20694 3.65519 -0.74838<br>H -5.60676 1.47163 -0.01269<br>H -7.16688 -0.44207 -0.36693<br>H -6.24569 -2.70057 -0.86934<br>H -3.79840 -3.04794 -0.97601<br>H -0.35675 2.33544 2.27387<br>H 0.71081 4.55139 2.49594<br>H 0.55755 6.20390 0.64738<br>H -0.68519 5.62635 -1.42557<br>H -1.75532 3.40024 -1.65037<br>S 1.22115 0.21291 -1.57625<br>C 0.53081 0.24990 0.07063<br>C 1.11149 -0.80898 1.02136<br>N 2.47463 -0.25089 1.39964<br>C 4.68247 0.41430 0.63097<br>C 5.58710 0.85211 -0.32597<br>C 5.14769 1.09443 -1.62685<br>C 3.81371 0.91014 -1.96106<br>C 2.88087 0.47366 -1.01020<br>C 3.34709 0.22279 0.28828<br>C 1.24018 -2.27199 0.63594<br>C 0.40787 -3.19747 1.27685<br>C 0.49799 -4.55260 0.97513<br>C 1.42160 -5.00219 0.03404<br>C 2.26111 -4.08826 -0.59801	-<br>2133.90068	-<br>2133.44291	-<br>2133.52768	-<br>2133.93597	-<br>2133.56297	-<br>2135.16116	-<br>2134.78816	-<br>2135.55787	-<br>2135.18487	21.1

C 2.17576 -2.73019 -0.30142<br>H 0.80359 1.22106 0.49744<br>H 5.01422 0.21906 1.64704<br>H 6.62647 1.00022 -0.05439<br>H 5.84468 1.43546 -2.38536<br>H 3.47455 1.10952 -2.97325<br>H -0.31143 -2.85721 2.01245<br>H -0.15653 -5.25620 1.48026<br>H 1.49152 -6.05995 -0.20146<br>H 2.99042 -4.42711 -1.32718<br>H 2.85327 -2.05212 -0.80612<br>O -0.98668 -0.10942 1.72615<br>O 0.30252 -0.65631 2.16070<br>H 2.30437 0.51910 2.06047<br>H 2.98271 -0.96939 1.92770										
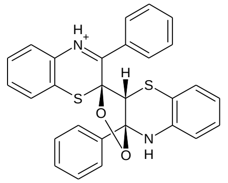<br>S -1.52438 -1.10262 -0.93183<br>C -0.83624 0.12856 0.21349<br>C -1.59891 1.43913 0.13583<br>N -2.89675 1.42710 -0.03100<br>C -5.13549 0.55468 -0.15304<br>C -5.99943 -0.49101 -0.43145<br>C -5.49074 -1.72915 -0.83856<br>C -4.12146 -1.92863 -0.95913<br>C -3.23921 -0.88154 -0.67961<br>C -3.75679 0.35534 -0.28408<br>C -0.97074 2.74791 0.33229<br>C -0.12462 2.98493 1.42945<br>C 0.40296 4.25472 1.62541<br>C 0.11159 5.28470 0.72964<br>C -0.72286 5.05044 -0.36199<br>C -1.27375 3.78934 -0.55978<br>H -5.51577 1.52028 0.16855<br>H -7.06944 -0.34565 -0.33048<br>H -6.16857 -2.54836 -1.05546<br>H -3.72932 -2.89311 -1.26601<br>H 0.08325 2.19623 2.14664<br>H 1.04007 4.44146 2.48393<br>H 0.53702 6.27156 0.88387<br>H -0.94247 5.84750 -1.06492<br>H -1.90138 3.60173 -1.42663<br>S 1.35319 0.08557 -1.66302<br>C 0.66564 0.22783 -0.01594	-2133.92271	-2133.46534	-2133.54978	-2133.95448	-2133.58155	-2135.17638	-2134.80346	-2135.57325	-2135.20033	11.4

C 1.22062 -0.78289 1.00375<br>N 2.56961 -0.40718 1.26733<br>C 4.77517 0.22525 0.57419<br>C 5.70245 0.64718 -0.36685<br>C 5.30142 0.91485 -1.67687<br>C 3.96596 0.75571 -2.02086<br>C 3.02458 0.31420 -1.08385<br>C 3.42411 0.03830 0.24101<br>C 1.05099 -2.27767 0.76377<br>C 0.18597 -3.04206 1.55322<br>C 0.07827 -4.41676 1.35497<br>C 0.83014 -5.04551 0.36445<br>C 1.70799 -4.29277 -0.41387<br>C 1.82572 -2.91974 -0.21032<br>H 0.98754 1.19864 0.36719<br>H 5.08780 0.02841 1.59658<br>H 6.73964 0.77512 -0.07229<br>H 6.01660 1.25373 -2.41935<br>H 3.63524 0.97265 -3.03312<br>H -0.40600 -2.56388 2.32498<br>H -0.59714 -4.99576 1.97822<br>H 0.74253 -6.11681 0.20786<br>H 2.31289 -4.77406 -1.17680<br>H 2.53570 -2.35762 -0.80743<br>O -0.92560 -0.28380 1.59083<br>O 0.41828 -0.35713 2.13089<br>H 3.03368 -1.01574 1.93037<br>H -3.35440 2.33243 0.06214										
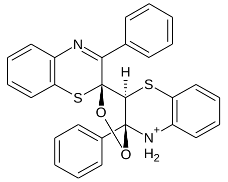<br>S -1.33190 -1.34508 -1.67771<br>C -0.00762 -0.53385 -0.68553<br>C 1.03296 -1.60572 -0.35149<br>N 0.65671 -2.70475 0.19392<br>C -0.98627 -4.05004 1.30143<br>C -2.27626 -4.54893 1.41061<br>C -3.27797 -4.09218 0.54883<br>C -2.99458 -3.11227 -0.39655<br>C -1.70423 -2.58370 -0.48706<br>C -0.67504 -3.06513 0.35070<br>C 2.48220 -1.39748 -0.57302<br>C 3.37115 -1.92662 0.37596<br>C 4.74453 -1.80330 0.20336<br>C 5.25300 -1.16284 -0.92775	-2133.92161	-2133.46413	-2133.54815	-2133.95572	-2133.58226	-2135.18415	-2134.81069	-2135.58100	-2135.20754	6.9

C 4.37756 -0.64840 -1.88239<br>C 2.99995 -0.75717 -1.70739<br>H -0.18689 -4.41546 1.93913<br>H -2.50184 -5.30553 2.15572<br>H -4.28526 -4.49074 0.62099<br>H -3.77596 -2.73962 -1.05245<br>H 2.96765 -2.42306 1.25266<br>H 5.41953 -2.20562 0.95321<br>H 6.32630 -1.06806 -1.06502<br>H 4.76574 -0.16205 -2.77241<br>H 2.33732 -0.36642 -2.47121<br>S 0.51934 0.38470 1.97491<br>C -0.60180 0.19985 0.54274<br>C -1.04313 1.53112 -0.11927<br>N -0.39110 2.73176 0.61534<br>C 1.83268 3.71585 0.12598<br>C 3.21664 3.63975 0.27390<br>C 3.79318 2.56705 0.95291<br>C 3.00463 1.54164 1.47246<br>C 1.62054 1.60899 1.33259<br>C 1.06407 2.70664 0.68061<br>C -2.52961 1.80205 -0.15902<br>C -3.13403 2.25161 -1.33574<br>C -4.49629 2.54112 -1.34322<br>C -5.25368 2.39121 -0.18314<br>C -4.64808 1.94786 0.99165<br>C -3.28803 1.65276 1.00688<br>H -1.46150 -0.33947 0.94356<br>H 1.37104 4.54902 -0.39583<br>H 3.84181 4.42219 -0.14283<br>H 4.87159 2.51638 1.06488<br>H 3.45789 0.69071 1.96958<br>H -2.55108 2.36138 -2.24395<br>H -4.96467 2.88201 -2.26130<br>H -6.31581 2.61626 -0.19442<br>H -5.23324 1.82393 1.89736<br>H -2.83523 1.29751 1.92988<br>O 0.53089 0.52107 -1.43668<br>O -0.51420 1.52108 -1.41134<br>H -0.77757 2.75199 1.56976<br>H -0.72040 3.58503 0.15250										
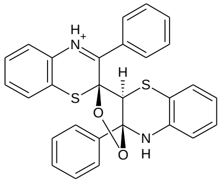<br>S -1.32623 -1.55316 -1.65445	-<br>2133.93805	-<br>2133.48105	-<br>2133.56612	-<br>2133.96917	-<br>2133.59723	-<br>2135.19382	-<br>2134.82189	-<br>2135.59045	-<br>2135.21852	0.0

C -0.06110 -0.55241 -0.74866										
C 1.04243 -1.50940 -0.34802										
N 0.66831 -2.58026 0.30684										
C -0.87093 -3.94063 1.58093										
C -2.13842 -4.48191 1.73740										
C -3.16490 -4.12553 0.85908										
C -2.93614 -3.21619 -0.16810										
C -1.66817 -2.65494 -0.33330										
C -0.64009 -3.03298 0.54472										
C 2.46415 -1.30921 -0.60569										
C 3.39718 -1.72159 0.36580										
C 4.75676 -1.59757 0.12266										
C 5.20462 -1.07743 -1.09250										
C 4.28678 -0.67110 -2.05992										
C 2.92191 -0.77150 -1.82139										
H -0.06292 -4.21259 2.25357										
H -2.32682 -5.18228 2.54391										
H -4.15607 -4.54989 0.98254										
H -3.73941 -2.93024 -0.83935										
H 3.06549 -2.08446 1.33499										
H 5.46721 -1.89573 0.88679										
H 6.26959 -0.98433 -1.28208										
H 4.63323 -0.27377 -3.00850										
H 2.22076 -0.47041 -2.58949										
S 0.40007 0.42927 1.88928										
C -0.66667 0.26266 0.42725										
C -1.02542 1.61702 -0.27072										
N -0.31537 2.73468 0.26746										
C 1.92485 3.70339 0.12870										
C 3.26391 3.67050 0.51048										
C 3.75735 2.61399 1.27598										
C 2.90238 1.58364 1.66335										
C 1.55717 1.62853 1.30384										
C 1.05623 2.68747 0.53541										
C -2.51749 1.91857 -0.26205										
C -3.35027 1.54475 -1.32049										
C -4.71643 1.81596 -1.26229										
C -5.26295 2.44860 -0.14747										
C -4.43463 2.81647 0.91188										
C -3.06715 2.55785 0.85439										
H -1.57153 -0.21707 0.80304										
H 1.54448 4.52392 -0.47439										
H 3.92663 4.47192 0.19761										
H 4.80304 2.58590 1.56588										
H 3.27344 0.75343 2.25698										
H -2.93593 1.04684 -2.18946										
H -5.35330 1.53012 -2.09437										
H -6.32837 2.65538 -0.10476										
H -4.85062 3.30933 1.78571										
H -2.42169 2.85024 1.67670										

O 0.41946 0.43153 -1.61533<br>O -0.66949 1.36169 -1.65937<br>H -0.60858 3.59642 -0.17769<br>H 1.41000 -3.20903 0.60489										
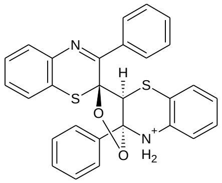<br>S 1.65041 1.90071 -1.98451<br>C 0.30801 1.04339 -1.08383<br>C -0.12460 1.93444 0.08458<br>N 0.74975 2.40308 0.89958<br>C 2.96949 2.53299 1.78408<br>C 4.34554 2.59189 1.61847<br>C 4.90019 2.47022 0.34111<br>C 4.07949 2.25781 -0.76189<br>C 2.69647 2.16127 -0.59195<br>C 2.12191 2.31966 0.68631<br>C -1.55117 2.23750 0.35108<br>C -2.01930 2.12573 1.66741<br>C -3.32810 2.47748 1.98160<br>C -4.17828 2.96535 0.98914<br>C -3.71418 3.09197 -0.31966<br>C -2.41181 2.72117 -0.64338<br>H 2.51544 2.65654 2.76279<br>H 4.98792 2.74898 2.47933<br>H 5.97545 2.53029 0.20358<br>H 4.51073 2.14447 -1.75225<br>H -1.34922 1.75640 2.43768<br>H -3.68293 2.37359 3.00291<br>H -5.19804 3.24737 1.23501<br>H -4.36732 3.48308 -1.09430<br>H -2.06221 2.83646 -1.66383<br>S 1.21852 -0.85618 0.95254<br>C 0.68170 -0.40644 -0.69885<br>C -0.54185 -1.19045 -1.19629<br>N -0.01631 -2.55127 -1.56188<br>C 0.88028 -4.62479 -0.66548<br>C 1.59912 -5.37351 0.25527<br>C 2.19689 -4.73456 1.34108<br>C 2.07958 -3.36120 1.49768<br>C 1.36021 -2.58223 0.57966<br>C 0.76345 -3.24819 -0.50047<br>C -1.83021 -1.31661 -0.40395<br>C -1.89989 -1.96780 0.83514<br>C -3.12448 -2.11248 1.48260	-<br>2133.89451	-<br>2133.43671	-<br>2133.52046	-<br>2133.93049	-<br>2133.55644	-<br>2135.16075	-<br>2134.78670	-<br>2135.55783	-<br>2135.18378	21.8

C -4.29030 -1.61504 0.90695<br>C -4.22762 -0.97477 -0.32833<br>C -3.00816 -0.82709 -0.98148<br>H 1.50441 -0.67537 -1.37098<br>H 0.40860 -5.11011 -1.51537<br>H 1.68878 -6.44608 0.12331<br>H 2.76147 -5.30704 2.06976<br>H 2.55049 -2.86907 2.34345<br>H -1.01866 -2.37594 1.31151<br>H -3.15956 -2.61997 2.44165<br>H -5.24260 -1.72940 1.41624<br>H -5.12939 -0.58484 -0.79029<br>H -2.97432 -0.32770 -1.94189<br>O -0.75133 0.85486 -2.02544<br>O -0.73696 -0.52798 -2.43384<br>H 0.56421 -2.44889 -2.40583<br>H -0.80803 -3.14729 -1.83038										
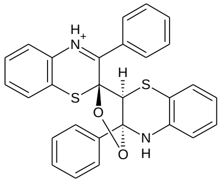<br>S -0.57343 -2.34359 -2.00941<br>C 0.21125 -0.94321 -1.11223<br>C 1.05533 -1.54371 -0.00407<br>N 0.46236 -2.39408 0.79622<br>C -1.40327 -3.59323 1.75372<br>C -2.59756 -4.27793 1.58616<br>C -3.18080 -4.36524 0.31962<br>C -2.58371 -3.75979 -0.78125<br>C -1.38826 -3.05614 -0.62580<br>C -0.80351 -2.98833 0.64763<br>C 2.45240 -1.21131 0.25975<br>C 2.86005 -1.00063 1.58949<br>C 4.19911 -0.77012 1.87461<br>C 5.14220 -0.77426 0.84685<br>C 4.74187 -0.99100 -0.47156<br>C 3.40199 -1.19268 -0.77543<br>H -0.93689 -3.51734 2.73159<br>H -3.07387 -4.74452 2.44165<br>H -4.11554 -4.90092 0.18899<br>H -3.04782 -3.82058 -1.76035<br>H 2.12636 -0.96479 2.39067<br>H 4.50449 -0.58192 2.89884<br>H 6.19019 -0.60314 1.07335<br>H 5.47656 -1.00136 -1.27033<br>H 3.10261 -1.36994 -1.80130	-2133.91502	-2133.45798	-2133.54199	-2133.94649	-2133.57346	-2135.17377	-2134.80075	-2135.57067	-2135.19764	13.1

S -1.49421 0.23434 0.98136<br>C -0.79439 0.14007 -0.66962<br>C -0.10571 1.43942 -1.12912<br>N -1.14532 2.38544 -1.37668<br>C -3.08003 3.61883 -0.67907<br>C -4.17148 3.84336 0.14640<br>C -4.45472 2.96197 1.19129<br>C -3.63207 1.86169 1.38924<br>C -2.51713 1.63526 0.57343<br>C -2.22570 2.52162 -0.48496<br>C 1.05628 2.03137 -0.33860<br>C 0.83174 2.61864 0.91410<br>C 1.87274 3.23739 1.60220<br>C 3.14794 3.29751 1.04327<br>C 3.37243 2.73772 -0.21238<br>C 2.33584 2.10768 -0.89772<br>H -1.63878 0.02712 -1.35721<br>H -2.86757 4.30127 -1.49825<br>H -4.80618 4.70587 -0.03277<br>H -5.31081 3.12302 1.83841<br>H -3.84539 1.15982 2.19114<br>H -0.15779 2.61519 1.35642<br>H 1.67944 3.68400 2.57327<br>H 3.95743 3.78599 1.57812<br>H 4.35863 2.78747 -0.66484<br>H 2.52517 1.67929 -1.87507<br>O 1.03046 -0.27208 -2.04911<br>O 0.35110 0.94988 -2.40962<br>H -0.79917 3.27691 -1.71044<br>H 1.03229 -2.76517 1.55418										
**Double-bond dimers**										
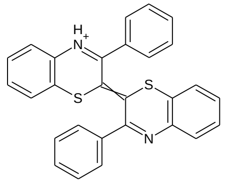<br>S -1.30306 2.41219 -0.91938<br>C -0.57651 0.84003 -0.48839<br>C -1.36681 -0.31109 -0.82649<br>N -2.68895 -0.23152 -0.77386<br>C -4.75303 0.59928 0.17334<br>C -5.51996 1.66300 0.63387<br>C -4.98551 2.95141 0.65872<br>C -3.68195 3.18359 0.22605<br>C -2.90963 2.12318 -0.24393<br>C -3.44996 0.83347 -0.26628<br>C -0.80041 -1.55367 -1.35954	-<br>1982.50999	-<br>1982.08785	-<br>1982.17029	-<br>1982.53709	-<br>1982.19739	-<br>1983.66106	-<br>1983.32136	-<br>1984.01083	-<br>1983.67113	0.0

C 0.32511 -1.50023 -2.19675<br>C 0.81712 -2.66540 -2.77241<br>C 0.19679 -3.88816 -2.51757<br>C -0.92063 -3.94746 -1.68290<br>C -1.42105 -2.78838 -1.10629<br>H -5.15618 -0.40916 0.15568<br>H -6.53368 1.48392 0.97623<br>H -5.58267 3.78229 1.02049<br>H -3.26478 4.18531 0.24771<br>H 0.78829 -0.54533 -2.42333<br>H 1.67960 -2.61662 -3.42954<br>H 0.58507 -4.79697 -2.96739<br>H -1.39209 -4.90121 -1.46830<br>H -2.25633 -2.85368 -0.41417<br>S 1.58853 2.31970 -0.06906<br>C 0.72193 0.81244 -0.00992<br>C 1.38285 -0.33622 0.63789<br>N 2.66601 -0.47385 0.74512<br>C 4.94800 0.11957 0.40676<br>C 5.93217 1.06796 0.19036<br>C 5.57966 2.40632 -0.02945<br>C 4.24503 2.78579 -0.06471<br>C 3.25111 1.81696 0.10785<br>C 3.58718 0.47641 0.37526<br>C 0.57060 -1.37106 1.33382<br>C -0.57327 -1.03113 2.06890<br>C -1.25887 -2.00488 2.79135<br>C -0.81635 -3.32671 2.78279<br>C 0.32881 -3.66799 2.06146<br>C 1.02342 -2.69617 1.35013<br>H 5.19509 -0.91552 0.62173<br>H 6.97814 0.77909 0.21235<br>H 6.35057 3.15548 -0.18007<br>H 3.97303 3.82068 -0.25165<br>H -0.91658 -0.00141 2.10423<br>H -2.13597 -1.72558 3.36775<br>H -1.35722 -4.08635 3.33961<br>H 0.67969 -4.69585 2.05082<br>H 1.91578 -2.95609 0.78978<br>H -3.21186 -1.02089 -1.13904										

Table S6. Diprotonated forms in methanol. For each chemical species, values are reported for the most stable conformer / geometric isomer identified. $G_{RRHO,calc}$ [M062X / 6-311++G(2d,2p) / SMD] = G_{el} [M062X / 6-311++G(2d,2p) / SMD // PBE0 / 6-31+G(d,p) / PCM] - G_{el} [PBE0 / 6-31+G(d,p) / PCM] + G_{RRHO} [PBE0 / 6-31+G(d,p) / PCM]; and likewise for $G_{RRHO,calc}$ [PBE0 / 6-31+G(d,p) / SMD] and for $G_{RRHO,calc}$ [M062X / 6-31+G(d,p) / SMD].

**Species**	**PBE0 / 6-31+G(d,p) / PCM**			**PBE0 / 6-31+G(d,p) / SMD**		**M062X / 6-31+G(d,p) / SMD**		**M062X / 6-311++G(2d,2p) / SMD**		
**Cartesian coordinates / Å**	**G_{el} / Ha**	**H_{RRHO} / Ha**	**G_{RRHO} / Ha**	**G_{el} / Ha**	**$G_{RRHO,calc}$ / Ha**	**G_{el} / Ha**	**$G_{RRHO,calc}$ / Ha**	**G_{el} / Ha**	**$G_{RRHO,calc}$ / Ha**	**$\Delta G_{RRHO,calc}$ / kcal mol^{-1}**
**Single-bond dimer**										
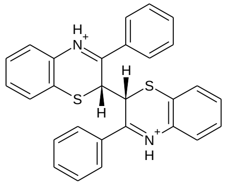<br>S 1.48772 0.74668 -2.37006<br>C 0.46346 0.63469 -0.88292<br>C 1.35000 0.69047 0.32880<br>N 2.40712 -0.08488 0.35642<br>C 3.80887 -1.90788 -0.38000<br>C 4.36660 -2.64977 -1.41071<br>C 4.02550 -2.37217 -2.73673<br>C 3.12090 -1.35970 -3.03861<br>C 2.54937 -0.60594 -2.01218<br>C 2.90324 -0.88836 -0.68357<br>C 1.03019 1.51832 1.48196<br>C 1.42333 1.12345 2.77675<br>C 1.13518 1.92775 3.86768<br>C 0.46346 3.13905 3.68720<br>C 0.06991 3.53886 2.41108<br>C 0.33912 2.73227 1.31326<br>H -0.16877 1.52365 -0.90681<br>H 4.06809 -2.11260 0.65465<br>H 5.06726 -3.44509 -1.18063<br>H 4.46191 -2.95350 -3.54244<br>H 2.85206 -1.15125 -4.06916<br>H 1.90030 0.16213 2.94668<br>H 1.42239 1.60472 4.86290<br>H 0.24265 3.76775 4.54421<br>H -0.44422 4.48336 2.26730<br>H 0.05372 3.08158 0.32670<br>S -1.48772 -0.74668 -2.37006<br>C -0.46346 -0.63469 -0.88292<br>C -1.35000 -0.69047 0.32880<br>N -2.40712 0.08488 0.35642<br>C -3.80887 1.90788 -0.38000	-1984.16444	-1983.70324	-1983.78656	-1984.21051	-1983.83263	-1985.32719	-1984.94930	-1985.67717	-1985.29929	0.0

C -4.36660 2.64977 -1.41071<br>C -4.02550 2.37217 -2.73673<br>C -3.12090 1.35970 -3.03861<br>C -2.54937 0.60594 -2.01218<br>C -2.90324 0.88836 -0.68357<br>C -1.03019 -1.51832 1.48196<br>C -1.42333 -1.12345 2.77675<br>C -1.13518 -1.92775 3.86768<br>C -0.46346 -3.13905 3.68720<br>C -0.06991 -3.53886 2.41108<br>C -0.33912 -2.73227 1.31326<br>H 0.16877 -1.52365 -0.90681<br>H -4.06809 2.11260 0.65465<br>H -5.06726 3.44509 -1.18063<br>H -4.46191 2.95350 -3.54244<br>H -2.85206 1.15125 -4.06916<br>H -1.90030 -0.16213 2.94668<br>H -1.42239 -1.60472 4.86290<br>H -0.24265 -3.76775 4.54421<br>H 0.44422 -4.48336 2.26730<br>H -0.05372 -3.08158 0.32670<br>H 2.98748 -0.04771 1.19062<br>H -2.98748 0.04771 1.19062										
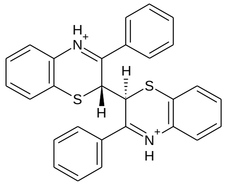<br>S 1.06336 1.00452 1.87155<br>C -0.18875 0.45585 0.67580<br>C -0.96179 1.64932 0.18016<br>N -0.28073 2.69143 -0.22335<br>C 1.69503 3.90670 -0.88759<br>C 3.03510 4.21156 -0.69982<br>C 3.77682 3.53208 0.26928<br>C 3.18944 2.53453 1.04051<br>C 1.84434 2.20793 0.85484<br>C 1.10356 2.91012 -0.10817<br>C -2.41394 1.66177 0.10169<br>C -3.06608 2.40354 -0.90374<br>C -4.45047 2.44108 -0.95146<br>C -5.20362 1.75837 0.00685<br>C -4.56701 1.02237 1.00430<br>C -3.18016 0.95659 1.04650<br>H -0.86228 -0.17830 1.25443<br>H 1.10265 4.43330 -1.63033<br>H 3.50042 4.98146 -1.30561	-1984.16481	-1983.70346	-1983.78586	-1984.20846	-1983.82950	-1985.32702	-1984.94806	-1985.67748	-1985.29853	0.5

H 4.82353 3.77470 0.42144<br>H 3.77158 2.00335 1.78721<br>H -2.50007 2.90198 -1.68613<br>H -4.94491 2.99440 -1.74308<br>H -6.28793 1.79559 -0.03058<br>H -5.15013 0.49518 1.75214<br>H -2.70661 0.39824 1.84718<br>S -0.83243 -0.83208 -1.75953<br>C 0.40057 -0.36484 -0.50453<br>C 1.02523 -1.66333 -0.06588<br>N 0.21144 -2.56895 0.43736<br>C -1.93033 -3.46160 1.11964<br>C -3.28907 -3.60956 0.88707<br>C -3.90062 -2.91643 -0.16126<br>C -3.16409 -2.05368 -0.96514<br>C -1.79794 -1.88365 -0.73440<br>C -1.18471 -2.60902 0.30058<br>C 2.43602 -1.96250 -0.20493<br>C 2.87277 -3.30258 -0.27103<br>C 4.22354 -3.59251 -0.37525<br>C 5.15937 -2.55677 -0.42345<br>C 4.73853 -1.22755 -0.38280<br>C 3.38760 -0.92839 -0.28390<br>H 1.13606 0.22675 -1.05231<br>H -1.44050 -4.00364 1.92313<br>H -3.87259 -4.26970 1.51972<br>H -4.96337 -3.03730 -0.34466<br>H -3.64652 -1.50040 -1.76465<br>H 2.16059 -4.12208 -0.29680<br>H 4.54794 -4.62587 -0.43841<br>H 6.21682 -2.78726 -0.50874<br>H 5.46413 -0.42223 -0.42911<br>H 3.08464 0.11134 -0.24307<br>H 0.63284 -3.38690 0.86850<br>H -0.81228 3.48454 -0.57396										
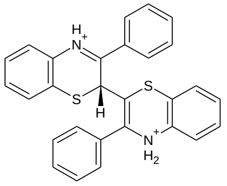	n.d.									
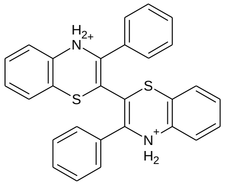	n.d.									

**Single-bond dimer, radical**										
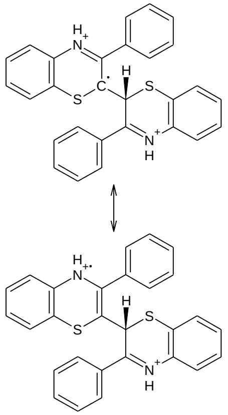<br>S -1.09722 1.34996 0.45623<br>C 0.19334 0.47089 -0.28420<br>C 1.44536 1.02230 -0.51726<br>N 1.75842 2.27662 -0.12580<br>C 1.49425 4.43431 0.89117<br>C 0.69992 5.35586 1.54827<br>C -0.63126 5.05073 1.86637<br>C -1.16770 3.81990 1.52573<br>C -0.37228 2.87975 0.86217<br>C 0.96352 3.18555 0.54214<br>C 2.54943 0.29189 -1.18373<br>C 2.43057 -0.11833 -2.51759<br>C 3.49369 -0.76976 -3.13615<br>C 4.67162 -1.01440 -2.43094<br>C 4.79234 -0.60112 -1.10499<br>C 3.73701 0.05598 -0.47875<br>H 2.52514 4.66612 0.64104<br>H 1.11387 6.32174 1.81726<br>H -1.24788 5.77919 2.38174<br>H -2.19784 3.58026 1.77007<br>H 1.52402 0.09497 -3.07698<br>H 3.40245 -1.07876 -4.17267<br>H 5.49748 -1.52517 -2.91667<br>H 5.70757 -0.79226 -0.55351<br>H 3.82709 0.36426 0.55935<br>S -1.06542 -1.16084 -2.22147<br>C -0.11008 -0.95698 -0.67472<br>C -0.73308 -1.74951 0.44849<br>N -2.03604 -1.80034 0.55368<br>C -4.30209 -1.05773 0.20551<br>C -5.27801 -0.51848 -0.62072<br>C -4.95682 -0.14006 -1.92609<br>C -3.66090 -0.29064 -2.40920	-<br>1983.54274	-<br>1983.09441	-<br>1983.17911	-<br>1983.58334	-<br>1983.21971	-<br>1984.69628	-<br>1984.33264	-<br>1985.04719	-<br>1984.68356	0.0

C -2.66992 -0.83557 -1.59112<br>C -3.00445 -1.21707 -0.28415<br>C 0.09714 -2.39131 1.45613<br>C 1.33898 -2.95332 1.10838<br>C 2.10821 -3.58822 2.07396<br>C 1.66519 -3.64964 3.39483<br>C 0.44240 -3.07899 3.75274<br>C -0.34295 -2.45865 2.79325<br>H 0.84895 -1.42369 -0.90738<br>H -4.53640 -1.34650 1.22622<br>H -6.28753 -0.39034 -0.24556<br>H -5.71962 0.28256 -2.57199<br>H -3.41344 0.00910 -3.42254<br>H 1.68815 -2.94751 0.08123<br>H 3.05417 -4.03911 1.79278<br>H 2.27578 -4.13722 4.14842<br>H 0.10729 -3.10553 4.78435<br>H -1.26660 -1.97660 3.10060<br>H 2.69284 2.59134 -0.36771<br>H -2.41065 -2.35107 1.32345										
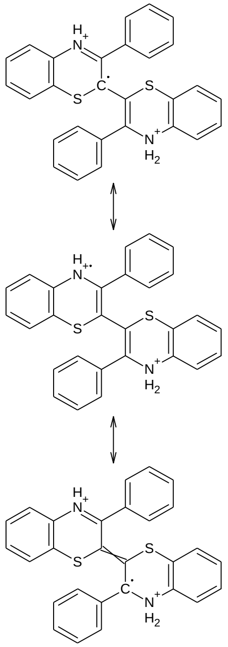<br>S -1.12157 -2.25883 0.00683<br>C -0.66618 -0.64844 -0.44761<br>C -1.58804 0.35991 -0.71276<br>N -2.91082 0.16928 -0.52535<br>C -4.94964 -0.92981 0.09675	-1983.52436	-1983.07529	-1983.15901	-1983.56676	-1983.20140	-1984.68444	-1984.31908	-1985.03644	-1984.67109	7.8

C -5.61461 -2.06462 0.52413<br>C -4.90783 -3.24642 0.78739<br>C -3.53300 -3.29064 0.62332<br>C -2.84886 -2.14748 0.19461<br>C -3.55920 -0.96207 -0.07072<br>C -1.22294 1.69795 -1.22101<br>C -0.40292 1.83431 -2.34878<br>C -0.11878 3.10038 -2.84797<br>C -0.64514 4.23451 -2.22785<br>C -1.46379 4.10145 -1.10817<br>C -1.75783 2.83767 -0.60498<br>H -5.49320 -0.01289 -0.11043<br>H -6.69112 -2.03572 0.65410<br>H -5.43589 -4.13300 1.12163<br>H -2.98290 -4.20408 0.82659<br>H -0.01240 0.95458 -2.85129<br>H 0.50619 3.20068 -3.72991<br>H -0.41894 5.22119 -2.62061<br>H -1.87025 4.98110 -0.61904<br>H -2.37503 2.74330 0.28476<br>S 1.63901 -1.58152 -1.61889<br>C 0.78674 -0.45416 -0.54843<br>C 1.46278 0.48760 0.14526<br>N 2.88266 0.65228 -0.24873<br>C 4.81873 -0.61317 0.68049<br>C 5.52523 -1.81159 0.74537<br>C 5.06252 -2.93985 0.06760<br>C 3.87920 -2.89938 -0.66525<br>C 3.16239 -1.70482 -0.73280<br>C 3.65903 -0.58499 -0.07673<br>C 1.00382 1.33137 1.25129<br>C 0.22713 0.77823 2.28119<br>C -0.20503 1.57596 3.33432<br>C 0.14062 2.92670 3.37922<br>C 0.92160 3.48040 2.36542<br>C 1.35168 2.69073 1.30451<br>H 5.17412 0.27451 1.19491<br>H 6.44148 -1.85937 1.32384<br>H 5.61931 -3.86958 0.12386<br>H 3.50647 -3.78722 -1.16542<br>H -0.01167 -0.28059 2.27108<br>H -0.79828 1.13720 4.13057<br>H -0.19310 3.54553 4.20657<br>H 1.18902 4.53200 2.39322<br>H 1.92327 3.15394 0.50319<br>H 3.30638 1.40630 0.30045<br>H -3.50736 0.94430 -0.79661<br>H 2.93583 0.94424 -1.23692										
**Single-bond dimer, C2-peroxyl radical**										

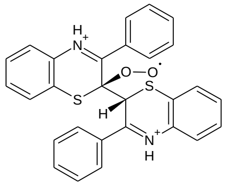<br>S 0.45579 -1.68125 -0.16188<br>C 0.36761 0.08940 -0.45534<br>C 1.74584 0.71766 -0.27039<br>N 2.75248 0.11455 -0.85111<br>C 3.87395 -1.43643 -2.31602<br>C 3.94667 -2.69421 -2.89121<br>C 2.94557 -3.63847 -2.64299<br>C 1.86062 -3.32921 -1.83045<br>C 1.77167 -2.06104 -1.25674<br>C 2.78508 -1.12515 -1.49515<br>C 2.05013 1.91543 0.51129<br>C 1.37575 3.13166 0.30808<br>C 1.74111 4.25052 1.04494<br>C 2.76242 4.16761 1.99246<br>C 3.44031 2.96540 2.19074<br>C 3.09700 1.84334 1.44811<br>H 4.64536 -0.69422 -2.49878<br>H 4.78421 -2.94265 -3.53371<br>H 3.00515 -4.62270 -3.09581<br>H 1.08059 -4.06181 -1.65100<br>H 0.62513 3.22441 -0.46959<br>H 1.23379 5.19354 0.86957<br>H 3.03322 5.04399 2.57305<br>H 4.23299 2.89791 2.92872<br>H 3.60137 0.89822 1.62847<br>S -0.46728 0.80003 2.18916<br>C -0.75838 0.74117 0.39990<br>C -2.12992 0.18844 0.07951<br>N -2.62034 -0.78707 0.79833<br>C -2.56014 -2.66854 2.30580<br>C -2.03589 -3.28939 3.43091<br>C -1.01376 -2.67376 4.15595<br>C -0.50342 -1.44210 3.75638<br>C -1.02150 -0.80864 2.62759<br>C -2.05174 -1.42976 1.91139<br>C -2.90652 0.70642 -1.03662<br>C -3.70952 -0.16853 -1.79420<br>C -4.45982 0.31995 -2.85313<br>C -4.43196 1.68095 -3.16139<br>C -3.64149 2.55407 -2.41422<br>C -2.87026 2.07334 -1.36520<br>H -0.76958 1.79022 0.09854	-<br>2133.70062	-<br>2133.24303	-<br>2133.33065	-<br>2133.74493	-<br>2133.37495	-<br>2134.95844	-<br>2134.58847	-<br>2135.35570	-<br>2134.98572	0.5

H -3.35003 -3.13926 1.72799<br>H -2.42360 -4.25366 3.74099<br>H -0.60379 -3.15893 5.03578<br>H 0.29614 -0.96955 4.31798<br>H -3.70190 -1.23714 -1.59922<br>H -5.05630 -0.36384 -3.44789<br>H -5.02271 2.06019 -3.98934<br>H -3.62699 3.61367 -2.64674<br>H -2.28993 2.77712 -0.77775<br>O -0.00522 0.24622 -1.87979<br>O 0.18813 1.47431 -2.28232<br>H -3.55631 -1.10337 0.55082<br>H 3.64222 0.60977 -0.81207										
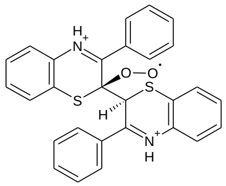<br>S 1.15006 -1.03758 1.58687<br>C -0.17830 -0.43824 0.48298<br>C -0.90855 -1.68987 0.01151<br>N -0.17646 -2.58714 -0.61309<br>C 1.85220 -3.48894 -1.57613<br>C 3.22584 -3.66125 -1.50729<br>C 3.96396 -3.02764 -0.50348<br>C 3.33881 -2.20239 0.42459<br>C 1.95721 -2.01131 0.36217<br>C 1.22155 -2.67234 -0.63331<br>C -2.30366 -1.99042 0.27594<br>C -2.64673 -3.32708 0.57183<br>C -3.96984 -3.67322 0.79822<br>C -4.96669 -2.69914 0.72633<br>C -4.63624 -1.37355 0.44107<br>C -3.31467 -1.01130 0.22856<br>H 1.26591 -3.98073 -2.34621<br>H 3.72379 -4.29274 -2.23498<br>H 5.03890 -3.16623 -0.45134<br>H 3.91794 -1.69713 1.19067<br>H -1.87254 -4.08098 0.68172<br>H -4.22202 -4.69905 1.04552<br>H -6.00258 -2.97173 0.90327<br>H -5.41259 -0.61776 0.38359<br>H -3.07931 0.01886 -0.00466<br>S -0.94338 0.78495 -1.97046<br>C 0.31300 0.45154 -0.70297<br>C 0.95747 1.72839 -0.22880<br>N 0.20502 2.77745 -0.01804	-2133.70225	-2133.24444	-2133.33259	-2133.74530	-2133.37564	-2134.95889	-2134.58923	-2135.35612	-2134.98646	0.0

C -1.83349 4.01865 0.30656<br>C -3.16574 4.24490 -0.00846<br>C -3.82178 3.40494 -0.90982<br>C -3.15669 2.32734 -1.48808<br>C -1.82079 2.08221 -1.16970<br>C -1.16549 2.94133 -0.27767<br>C 2.38587 1.80980 0.03389<br>C 2.86823 2.61527 1.08435<br>C 4.23225 2.72087 1.30651<br>C 5.13128 2.04447 0.47902<br>C 4.66184 1.24803 -0.56443<br>C 3.29681 1.11455 -0.78115<br>H 1.07035 -0.13423 -1.23063<br>H -1.31021 4.66658 1.00358<br>H -3.69074 5.07759 0.44665<br>H -4.86171 3.58459 -1.16237<br>H -3.67235 1.67558 -2.18664<br>H 2.18171 3.11009 1.76530<br>H 4.59574 3.32250 2.13298<br>H 6.19903 2.13483 0.65264<br>H 5.35948 0.72920 -1.21352<br>H 2.95410 0.51755 -1.61974<br>O -1.08145 0.36590 1.27026<br>O -0.76845 0.42661 2.53624<br>H 0.68189 3.61917 0.29991<br>H -0.67826 -3.35981 -1.04569										
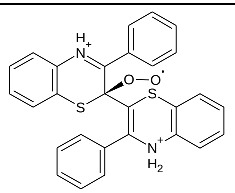	n.d.									
**Single-bond dimer, C3-peroxyl radical**										
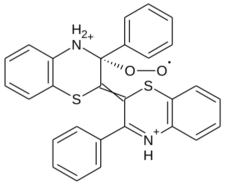<br>S -0.96547 1.68992 1.48735<br>C -0.94642 0.16157 0.59295<br>C -2.23631 -0.16623 -0.02936<br>N -2.88591 0.79658 -0.64214<br>C -3.10648 2.94767 -1.72020<br>C -2.81181 4.30274 -1.75451<br>C -1.94440 4.85568 -0.80949<br>C -1.35703 4.05789 0.16715	-2133.68733	-2133.22926	-2133.31674	-2133.73224	-2133.36165	-2134.94842	-2134.57783	-2135.34718	-2134.97659	0.0

C -1.63890 2.69264 0.20490<br>C -2.51940 2.14616 -0.73857<br>C -2.83413 -1.49269 0.03774<br>C -2.70432 -2.24249 1.22043<br>C -3.31859 -3.48375 1.31875<br>C -4.04043 -3.99399 0.23966<br>C -4.16044 -3.25785 -0.94125<br>C -3.57003 -2.00682 -1.04511<br>H -3.78163 2.50739 -2.44792<br>H -3.25979 4.92945 -2.51799<br>H -1.71622 5.91622 -0.83668<br>H -0.67615 4.48924 0.89395<br>H -2.15812 -1.83820 2.06736<br>H -3.23422 -4.05308 2.23858<br>H -4.50795 -4.97083 0.31569<br>H -4.70759 -3.66511 -1.78514<br>H -3.63304 -1.46049 -1.98230<br>S 0.11775 -2.23987 -0.21772<br>C 0.15526 -0.63088 0.52087<br>C 1.45466 -0.15552 1.16252<br>N 2.46433 -1.29284 1.19316<br>C 4.12366 -1.81777 -0.57608<br>C 4.37391 -2.34669 -1.84089<br>C 3.32552 -2.82986 -2.62228<br>C 2.00998 -2.78478 -2.16302<br>C 1.75342 -2.26701 -0.89629<br>C 2.81427 -1.80591 -0.12684<br>C 2.08460 1.06899 0.55383<br>C 1.87993 1.36562 -0.79668<br>C 2.52827 2.45551 -1.36948<br>C 3.37832 3.24764 -0.60029<br>C 3.58491 2.94801 0.74563<br>C 2.94543 1.85761 1.32687<br>H 4.93232 -1.43888 0.04114<br>H 5.39183 -2.37612 -2.21390<br>H 3.52908 -3.23619 -3.60766<br>H 1.19333 -3.14345 -2.78062<br>H 1.21696 0.75724 -1.40433<br>H 2.36579 2.68386 -2.41798<br>H 3.87985 4.09956 -1.04916<br>H 4.24294 3.56493 1.34919<br>H 3.10640 1.64374 2.37922<br>H 3.30636 -0.95343 1.67269<br>H -3.79197 0.55517 -1.03727<br>O 1.22122 0.12529 2.58386<br>O 0.95546 -0.95682 3.26600<br>H 2.06204 -2.03871 1.78742										
**Single-bond dimer, dioxolane radical**										

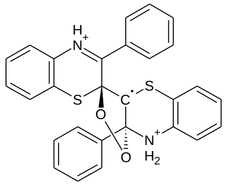<br>S -1.85693 -1.63894 -0.85811<br>C -1.09092 -0.25120 0.07779<br>C -1.96091 0.97550 -0.12790<br>N -3.25007 0.83817 0.07771<br>C -5.28278 -0.24713 0.77854<br>C -6.04899 -1.39628 0.88485<br>C -5.52988 -2.62638 0.46829<br>C -4.24050 -2.71679 -0.04214<br>C -3.45446 -1.56743 -0.14711<br>C -3.98834 -0.33572 0.25770<br>C -1.44761 2.27520 -0.52018<br>C -2.17562 3.04860 -1.44437<br>C -1.72089 4.31120 -1.79786<br>C -0.55335 4.81754 -1.22549<br>C 0.16740 4.05796 -0.30218<br>C -0.26597 2.78649 0.04517<br>H -5.67248 0.71479 1.09774<br>H -7.05252 -1.33659 1.29176<br>H -6.13225 -3.52506 0.55225<br>H -3.83681 -3.67479 -0.35313<br>H -3.06055 2.64080 -1.92498<br>H -2.27087 4.89559 -2.52808<br>H -0.20142 5.80660 -1.50224<br>H 1.06697 4.46003 0.15240<br>H 0.28162 2.21734 0.78836<br>S 0.94490 -0.16852 -1.88505<br>C 0.35877 -0.20084 -0.28129<br>C 1.09645 -0.79272 0.88222<br>N 1.80773 -2.06946 0.41550<br>C 4.03772 -2.46579 -0.53695<br>C 4.97517 -2.28021 -1.54894<br>C 4.67137 -1.46496 -2.63717<br>C 3.44012 -0.82323 -2.71814<br>C 2.48811 -1.01074 -1.71317<br>C 2.80408 -1.84081 -0.63644<br>C 2.06391 0.08957 1.63667<br>C 2.21528 -0.10570 3.01402<br>C 3.12561 0.66970 3.72604<br>C 3.88423 1.63834 3.07052<br>C 3.72972 1.83564 1.69955<br>C 2.81997 1.06566 0.98016<br>H 4.26357 -3.09941 0.31611	-2133.69785	-2133.23905	-2133.32524	-2133.74320	-2133.37059	-2134.95769	-2134.58508	-2135.35533	-2134.98272	12.4

H 5.94045 -2.76937 -1.47966<br>H 5.40167 -1.31345 -3.42505<br>H 3.21659 -0.17025 -3.55615<br>H 1.61465 -0.84825 3.53014<br>H 3.23585 0.51928 4.79534<br>H 4.59104 2.24424 3.62927<br>H 4.31238 2.59411 1.18638<br>H 2.70228 1.24326 -0.08435<br>O -1.05767 -0.45697 1.48338<br>O 0.06088 -1.31743 1.70364<br>H 1.08434 -2.73394 0.10608<br>H -3.79479 1.69893 0.07226<br>H 2.26723 -2.50314 1.22439										
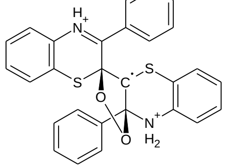<br>S 1.17957 -1.70590 1.63896<br>C 1.04495 -0.05574 0.78791<br>C 2.35068 0.10407 0.05033<br>N 2.63016 -0.84367 -0.81925<br>C 2.20322 -2.84367 -2.10643<br>C 1.70531 -4.13520 -2.17452<br>C 1.03957 -4.68863 -1.07685<br>C 0.85299 -3.95101 0.08688<br>C 1.34043 -2.64520 0.16797<br>C 2.02577 -2.10534 -0.93279<br>C 3.30646 1.17679 0.25310<br>C 4.68035 0.86817 0.16449<br>C 5.62931 1.86893 0.30429<br>C 5.22526 3.18652 0.52434<br>C 3.86881 3.50024 0.61838<br>C 2.90970 2.50559 0.49669<br>H 2.72784 -2.40287 -2.94867<br>H 1.83699 -4.71374 -3.08242<br>H 0.65091 -5.70030 -1.13240<br>H 0.32193 -4.37897 0.93087<br>H 5.00570 -0.16117 0.04517<br>H 6.68413 1.61963 0.25636<br>H 5.96980 3.96919 0.63252<br>H 3.55640 4.52553 0.78646<br>H 1.86276 2.76719 0.56103<br>S -0.37088 0.22364 -1.67997<br>C -0.22209 0.09703 0.01448<br>C -1.21116 0.63828 0.99287<br>N -1.65907 2.03655 0.48387	-2133.69566	-2133.23678	-2133.32141	-2133.74228	-2133.36803	-2134.95648	-2134.58223	-2135.35432	-2134.98007	14.0

C -3.52099 2.71456 -0.97264<br>C -4.17970 2.67429 -2.19837<br>C -3.67756 1.88805 -3.23294<br>C -2.52404 1.13337 -3.04941<br>C -1.85015 1.17523 -1.82647<br>C -2.36006 1.97582 -0.80196<br>C -2.44527 -0.16262 1.32893<br>C -2.77320 -1.30784 0.60311<br>C -3.91378 -2.03360 0.93886<br>C -4.71757 -1.62348 1.99987<br>C -4.38147 -0.48523 2.73290<br>C -3.24762 0.24841 2.40035<br>H -3.90650 3.32391 -0.16057<br>H -5.08575 3.25376 -2.33712<br>H -4.19088 1.84994 -4.18797<br>H -2.14562 0.50772 -3.85165<br>H -2.14502 -1.64132 -0.21697<br>H -4.16959 -2.92181 0.36991<br>H -5.60496 -2.19242 2.26000<br>H -4.99909 -0.16858 3.56727<br>H -2.98760 1.12105 2.99499<br>O 0.92075 0.92537 1.79518<br>O -0.46883 0.82141 2.17559<br>H -2.27697 2.45587 1.18723<br>H 3.43221 -0.67849 -1.42374<br>H -0.83761 2.65083 0.42708										
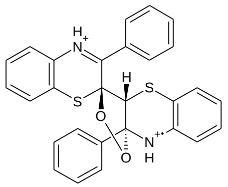<br>S 1.76083 -1.25084 0.70944<br>C 0.80983 0.07590 -0.06050<br>C 1.60008 1.37513 0.09008<br>N 2.86433 1.35405 -0.25312<br>C 4.89160 0.45787 -1.20679<br>C 5.72138 -0.62760 -1.43664<br>C 5.33601 -1.90614 -1.02146<br>C 4.11470 -2.11103 -0.38968<br>C 3.26536 -1.02692 -0.16586<br>C 3.66674 0.25324 -0.56443<br>C 1.06939 2.64665 0.57885<br>C 1.78262 3.33140 1.57841<br>C 1.32221 4.55974 2.03732<br>C 0.16947 5.12222 1.49127<br>C -0.52997 4.45511 0.48438<br>C -0.09305 3.21706 0.03312	-2133.72169	-2133.26445	-2133.35065	-2133.76427	-2133.39322	-2134.97674	-2134.60570	-2135.37329	-2135.00224	0.1

H 5.17975 1.45667 -1.52168<br>H 6.67148 -0.47992 -1.93833<br>H 5.98895 -2.75388 -1.20171<br>H 3.81401 -3.10693 -0.08056<br>H 2.66730 2.88328 2.02166<br>H 1.86276 5.07424 2.82508<br>H -0.18571 6.08339 1.84975<br>H -1.41584 4.90183 0.04484<br>H -0.62155 2.72551 -0.77720<br>S -1.05010 -0.90218 1.92175<br>C -0.65457 0.11088 0.48334<br>C -1.48096 -0.16012 -0.79131<br>N -1.68384 -1.58443 -1.03640<br>C -1.83408 -3.93198 -0.71998<br>C -1.81400 -5.01128 0.11632<br>C -1.55486 -4.83914 1.49978<br>C -1.30455 -3.58065 2.02607<br>C -1.30585 -2.45823 1.19825<br>C -1.59638 -2.62230 -0.20143<br>C -2.80134 0.57932 -0.89841<br>C -3.85576 0.21032 -0.05699<br>C -5.07016 0.88433 -0.13262<br>C -5.24256 1.91610 -1.05498<br>C -4.19761 2.26952 -1.90552<br>C -2.97576 1.60386 -1.83314<br>H -0.89141 1.12132 0.82045<br>H -2.03310 -4.04368 -1.78130<br>H -1.99598 -6.00502 -0.27755<br>H -1.53930 -5.70370 2.15467<br>H -1.09267 -3.46429 3.08388<br>H -3.74172 -0.59931 0.65770<br>H -5.88297 0.59806 0.52731<br>H -6.19274 2.43800 -1.11492<br>H -4.32916 3.06403 -2.63344<br>H -2.17384 1.88042 -2.50934<br>O 0.65766 -0.22496 -1.44928<br>O -0.62325 0.31997 -1.80391<br>H 3.33454 2.25727 -0.26643<br>H -1.96086 -1.78592 -1.99462										
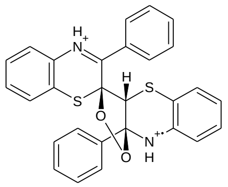<br>S 1.50691 -1.15570 0.87474<br>C 0.85186 0.12143 -0.22984<br>C 1.64780 1.41243 -0.14686	-2133.70430	-2133.24672	-2133.33212	-2133.74689	-2133.37471	-2134.95953	-2134.58735	-2135.35612	-2134.98394	11.6

N 2.94097 1.36732 0.04008<br>C 5.15991 0.45333 0.19715<br>C 5.99812 -0.61303 0.47485<br>C 5.45922 -1.84852 0.84900<br>C 4.08422 -2.02614 0.93773<br>C 3.22965 -0.95737 0.65957<br>C 3.77536 0.27741 0.29713<br>C 1.04487 2.73265 -0.34175<br>C 0.22881 2.99669 -1.45587<br>C -0.28049 4.27499 -1.64313<br>C 0.00051 5.28671 -0.72338<br>C 0.80596 5.02572 0.38373<br>C 1.33760 3.75545 0.57508<br>H 5.56422 1.41610 -0.10214<br>H 7.07234 -0.48594 0.39807<br>H 6.11750 -2.68358 1.06520<br>H 3.66917 -2.98859 1.21926<br>H 0.03628 2.22603 -2.19635<br>H -0.89178 4.48374 -2.51515<br>H -0.40918 6.28090 -0.87235<br>H 1.01846 5.80928 1.10369<br>H 1.94235 3.54654 1.45303<br>S -1.33886 0.18152 1.66287<br>C -0.64563 0.26306 0.01109<br>C -1.22363 -0.74717 -0.98925<br>N -2.59983 -0.35321 -1.20361<br>C -4.79001 0.30291 -0.57915<br>C -5.68917 0.69114 0.37263<br>C -5.26762 0.90741 1.70845<br>C -3.93981 0.74841 2.06647<br>C -2.98450 0.37154 1.11888<br>C -3.41772 0.11116 -0.24064<br>C -1.12650 -2.24778 -0.75815<br>C -0.30973 -3.02247 -1.58888<br>C -0.22535 -4.39865 -1.39862<br>C -0.95569 -5.01406 -0.38431<br>C -1.78742 -4.24839 0.43145<br>C -1.88100 -2.87325 0.24169<br>H -0.93799 1.23990 -0.38439<br>H -5.10071 0.12558 -1.60394<br>H -6.73089 0.83192 0.10651<br>H -5.98891 1.21653 2.45726<br>H -3.62612 0.93648 3.08817<br>H 0.26416 -2.55291 -2.37893<br>H 0.41387 -4.98889 -2.04796<br>H -0.88772 -6.08772 -0.23695<br>H -2.37426 -4.72043 1.21316<br>H -2.55492 -2.30880 0.87648<br>O 0.88778 -0.26298 -1.62771<br>O -0.47028 -0.34043 -2.12946										

H 3.41938 2.26391 -0.03932<br>H -3.02952 -0.65846 -2.07341										
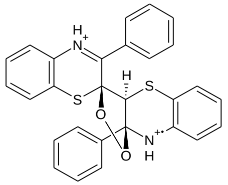<br>S -1.02168 -2.12676 -1.63486<br>C -0.02026 -0.76106 -0.91790<br>C 1.29981 -1.35394 -0.47120<br>N 1.21824 -2.39456 0.31919<br>C 0.09710 -3.92351 1.81605<br>C -0.99518 -4.72858 2.10308<br>C -2.10293 -4.73602 1.25191<br>C -2.13498 -3.92805 0.11984<br>C -1.04817 -3.10452 -0.17659<br>C 0.06757 -3.11776 0.67571<br>C 2.61242 -0.81436 -0.80225<br>C 3.62356 -0.83690 0.18005<br>C 4.89453 -0.37075 -0.11910<br>C 5.18105 0.10328 -1.39999<br>C 4.18848 0.11968 -2.37951<br>C 2.90502 -0.32201 -2.08769<br>H 0.96688 -3.91038 2.46608<br>H -0.98306 -5.35242 2.99017<br>H -2.95584 -5.36765 1.47784<br>H -3.00251 -3.92700 -0.53193<br>H 3.40703 -1.16613 1.19255<br>H 5.65934 -0.36896 0.65054<br>H 6.17835 0.46342 -1.63291<br>H 4.41364 0.47671 -3.37910<br>H 2.15229 -0.32444 -2.86605<br>S -0.04966 0.30009 1.79767<br>C -0.82412 0.02836 0.19004<br>C -1.25012 1.29895 -0.58649<br>N -0.38546 2.44792 -0.36832<br>C 1.47243 3.77387 0.29191<br>C 2.50127 4.00437 1.15989<br>C 2.77158 3.09096 2.21185<br>C 2.00812 1.94595 2.37385<br>C 0.95577 1.67530 1.49789<br>C 0.66204 2.60895 0.44361<br>C -2.70965 1.67371 -0.43297<br>C -3.68377 0.93012 -1.10782<br>C -5.02813 1.24707 -0.94222<br>C -5.40518 2.29393 -0.10086<br>C -4.43331 3.02672 0.57598	-2133.72133	-2133.26362	-2133.35026	-2133.76319	-2133.39212	-2134.97707	-2134.60601	-2135.37348	-2135.00242	0.0

C -3.08379 2.72089 0.41077<br>H -1.72559 -0.53609 0.43473<br>H 1.25430 4.45980 -0.52036<br>H 3.11911 4.88806 1.04437<br>H 3.59415 3.28485 2.89200<br>H 2.22877 1.25038 3.17695<br>H -3.39719 0.11247 -1.76171<br>H -5.78188 0.67366 -1.47285<br>H -6.45584 2.53650 0.02657<br>H -4.72049 3.84015 1.23482<br>H -2.33415 3.29668 0.94481<br>O 0.18489 0.19576 -1.92087<br>O -1.05616 0.90992 -1.94246<br>H 2.09878 -2.78576 0.64730<br>H -0.56074 3.19599 -1.03514										
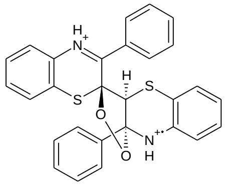<br>S -0.45036 -2.37990 -2.00733<br>C 0.25081 -0.94049 -1.11667<br>C 1.14314 -1.48233 -0.01352<br>N 0.59765 -2.35461 0.79755<br>C -1.21173 -3.62502 1.77546<br>C -2.37568 -4.36139 1.61736<br>C -2.95949 -4.48486 0.35391<br>C -2.39382 -3.86483 -0.75523<br>C -1.22876 -3.11015 -0.61005<br>C -0.64196 -3.00441 0.66119<br>C 2.52575 -1.08368 0.22276<br>C 2.95153 -0.87429 1.54809<br>C 4.28257 -0.58100 1.80875<br>C 5.20224 -0.52063 0.76151<br>C 4.78575 -0.73751 -0.55183<br>C 3.45206 -1.00249 -0.83176<br>H -0.74460 -3.52321 2.75061<br>H -2.82818 -4.84114 2.47835<br>H -3.87075 -5.06132 0.23211<br>H -2.85836 -3.95517 -1.73175<br>H 2.23575 -0.88692 2.36587<br>H 4.60018 -0.39384 2.82933<br>H 6.24478 -0.30001 0.96925<br>H 5.50309 -0.70036 -1.36528<br>H 3.14520 -1.18592 -1.85430<br>S -1.62784 0.10772 0.92016<br>C -0.81781 0.08726 -0.68465	-2133.69680	-2133.23934	-2133.32534	-2133.73885	-2133.36739	-2134.95665	-2134.58520	-2135.35326	-2134.98180	12.9

C -0.16032 1.41475 -1.08320<br>N -1.24036 2.35605 -1.28391<br>C -3.22943 3.52116 -0.72515<br>C -4.31408 3.69827 0.08572<br>C -4.56791 2.79651 1.14974<br>C -3.73770 1.71269 1.37211<br>C -2.62635 1.48622 0.55390<br>C -2.33949 2.42824 -0.51006<br>C 0.95418 2.05835 -0.27025<br>C 0.72509 2.54633 1.02280<br>C 1.74289 3.19067 1.71850<br>C 2.99286 3.37150 1.12793<br>C 3.21762 2.90846 -0.16590<br>C 2.20465 2.25835 -0.86494<br>H -1.62319 -0.04883 -1.41563<br>H -3.02008 4.20779 -1.53954<br>H -4.98390 4.53419 -0.08304<br>H -5.43215 2.94680 1.78780<br>H -3.95551 1.01611 2.17539<br>H -0.24156 2.44614 1.50244<br>H 1.55204 3.56024 2.72119<br>H 3.78373 3.87976 1.67141<br>H 4.18371 3.05320 -0.63946<br>H 2.38921 1.90969 -1.87402<br>O 1.01972 -0.20473 -2.05802<br>O 0.29990 1.00433 -2.37059<br>H 1.19190 -2.69625 1.55106<br>H -1.03771 3.15276 -1.88278										
**Single-bond dimer, dioxane radical**										
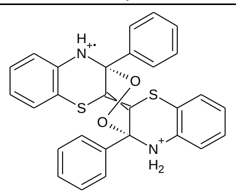<br>S -1.50857 -0.47385 -1.86598<br>C -0.47297 0.07425 -0.57228<br>C -1.09572 0.18613 0.80263<br>N -2.22601 1.09188 0.72661<br>C -4.40982 1.71288 -0.00870<br>C -5.44210 1.53601 -0.88777<br>C -5.30689 0.66123 -1.99745<br>C -4.11827 0.00231 -2.23531<br>C -3.03009 0.18588 -1.37038<br>C -3.19237 1.00389 -0.19778<br>C -1.46399 -1.16423 1.40392<br>C -2.44343 -1.22654 2.40084<br>C -2.73352 -2.43949 3.01688	-<br>2133.69369	-<br>2133.23512	-<br>2133.32059	-<br>2133.73956	-<br>2133.36646	-<br>2134.95833	-<br>2134.58523	-<br>2135.35649	-<br>2134.98339	6.3

C -2.05051 -3.59692 2.64327<br>C -1.07301 -3.53659 1.65317<br>C -0.77893 -2.32424 1.03347<br>H -4.51466 2.35259 0.86172<br>H -6.38009 2.05633 -0.72716<br>H -6.13996 0.52543 -2.67856<br>H -4.00582 -0.63034 -3.10985<br>H -2.98396 -0.33677 2.70903<br>H -3.49459 -2.47884 3.78999<br>H -2.28121 -4.54303 3.12352<br>H -0.53700 -4.43278 1.35658<br>H -0.01245 -2.29651 0.26544<br>S 1.71302 -0.04808 -2.24853<br>C 0.83928 0.29700 -0.74849<br>C 1.65344 0.75502 0.43537<br>N 2.99725 0.01351 0.42523<br>C 3.67117 -2.36533 0.44470<br>C 3.81362 -3.57375 -0.23378<br>C 3.35026 -3.70233 -1.54140<br>C 2.71535 -2.63918 -2.18116<br>C 2.57197 -1.42562 -1.51379<br>C 3.06448 -1.30737 -0.21326<br>C 1.93521 2.24087 0.51426<br>C 2.48417 2.74819 1.69819<br>C 2.78055 4.10304 1.79862<br>C 2.52712 4.95579 0.72411<br>C 1.97022 4.45251 -0.44905<br>C 1.67135 3.09614 -0.55694<br>H 4.03935 -2.25341 1.46015<br>H 4.28906 -4.41172 0.26426<br>H 3.46428 -4.64531 -2.06623<br>H 2.32340 -2.75151 -3.18665<br>H 2.65874 2.09665 2.55015<br>H 3.20240 4.49293 2.71956<br>H 2.75773 6.01365 0.80518<br>H 1.76141 5.11410 -1.28374<br>H 1.22697 2.72260 -1.47333<br>H 3.32753 -0.04555 1.39681<br>O 1.04854 0.26562 1.62669<br>O -0.20903 0.91531 1.66737<br>H -2.36522 1.71620 1.51663<br>H 3.65859 0.64358 -0.04799										
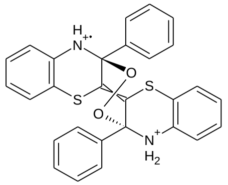	-2133.70596	-2133.24733	-2133.33164	-2133.75110	-2133.37679	-2134.96969	-2134.59537	-2135.36782	-2134.99350	0.0

S -1.77636 -1.31075 -1.45934<br>C -0.75023 -0.46843 -0.32302<br>C -1.46065 0.47968 0.61363<br>N -2.36345 -0.33411 1.42249<br>C -4.27825 -1.73171 1.73121<br>C -5.24606 -2.52608 1.17947<br>C -5.21306 -2.85652 -0.19954<br>C -4.18308 -2.41630 -1.00519<br>C -3.15713 -1.62974 -0.46135<br>C -3.23586 -1.21712 0.91353<br>C -2.16882 1.63243 -0.08633<br>C -1.53119 2.26015 -1.16108<br>C -2.13129 3.34835 -1.78451<br>C -3.36761 3.81516 -1.33735<br>C -3.99935 3.19394 -0.26260<br>C -3.40256 2.10287 0.36619<br>H -4.31471 -1.44111 2.77619<br>H -6.05698 -2.89366 1.79899<br>H -5.99228 -3.48238 -0.62082<br>H -4.13777 -2.71078 -2.04878<br>H -0.56840 1.90174 -1.51370<br>H -1.63469 3.82876 -2.62175<br>H -3.83735 4.66245 -1.82777<br>H -4.95993 3.55539 0.09078<br>H -3.90932 1.63220 1.20228<br>S 1.37755 -1.86365 -1.27001<br>C 0.57789 -0.70347 -0.23869<br>C 1.32633 0.00274 0.87871<br>N 2.26269 -1.01306 1.51607<br>C 4.65825 -1.42146 1.10035<br>C 5.67711 -1.83455 0.24635<br>C 5.37734 -2.22849 -1.05605<br>C 4.06587 -2.20743 -1.52158<br>C 3.03582 -1.80650 -0.66957<br>C 3.35458 -1.42727 0.63152<br>C 2.08580 1.25414 0.50942<br>C 2.50117 2.10653 1.53912<br>C 3.22360 3.25491 1.23533<br>C 3.52989 3.55880 -0.09150<br>C 3.10943 2.71407 -1.11584<br>C 2.38673 1.56091 -0.81852<br>H 4.87920 -1.10639 2.11611<br>H 6.70190 -1.84045 0.60078<br>H 6.17174 -2.54392 -1.72434<br>H 3.84049 -2.49692 -2.54299<br>H 2.24264 1.89189 2.57250<br>H 3.53990 3.91669 2.03536<br>H 4.09146 4.45801 -0.32585<br>H 3.33948 2.95010 -2.14992<br>H 2.06239 0.91395 -1.62822										

H 2.65266 -0.58710 2.36540<br>O 0.42324 0.19911 1.95488<br>O -0.54954 1.13380 1.47470<br>H -2.43350 -0.08414 2.40496<br>H 1.71178 -1.82461 1.83129										
**Single-bond dimer, C2-hydroperoxylalkyl radical**										
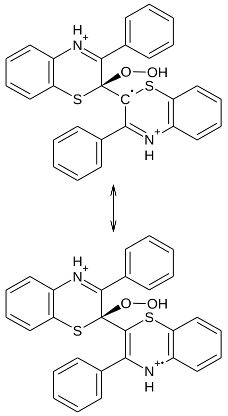<br>S -1.15520 -2.09766 -0.59066<br>C -0.48420 -0.88824 0.60781<br>C -1.33645 0.35888 0.80659<br>N -2.62769 0.30117 0.60328<br>C -4.82230 -0.50169 0.07815<br>C -5.65635 -1.46835 -0.45789<br>C -5.11010 -2.63088 -1.00950<br>C -3.73488 -2.83199 -1.02158<br>C -2.88605 -1.86329 -0.48243<br>C -3.43678 -0.69886 0.05844<br>C -0.79537 1.57527 1.40375<br>C 0.29489 1.53046 2.29180<br>C 0.75205 2.69919 2.88569<br>C 0.13985 3.92007 2.60243<br>C -0.93683 3.97426 1.71652<br>C -1.40280 2.81321 1.11840<br>H -5.23290 0.40479 0.51434<br>H -6.73037 -1.31923 -0.44542<br>H -5.76114 -3.39089 -1.42906<br>H -3.31724 -3.74030 -1.44457<br>H 0.75138 0.58549 2.56038<br>H 1.58211 2.65324 3.58316<br>H 0.50435 4.83028 3.06854<br>H -1.40174 4.92476 1.47626<br>H -2.19725 2.88536 0.37988<br>C 0.95589 -0.67133 0.15558	-2133.72927	-2133.27153	-2133.35890	-2133.77068	-2133.40031	-2134.98871	-2134.61834	-2135.38753	-2135.01716	0.0

S 2.01639 -1.84559 0.87392<br>C 3.56140 -1.46923 0.17155<br>C 3.74087 -0.41779 -0.74436<br>N 2.67068 0.34744 -1.15541<br>C 1.37086 0.25851 -0.78636<br>C 4.65336 -2.25372 0.56046<br>C 5.90767 -1.98644 0.03775<br>C 6.08631 -0.93562 -0.87396<br>C 5.01447 -0.15485 -1.26547<br>C 0.51477 1.26286 -1.46658<br>C 0.86946 2.61622 -1.35504<br>C 0.14672 3.58277 -2.04424<br>C -0.92354 3.20951 -2.85757<br>C -1.26519 1.86490 -2.98551<br>C -0.54934 0.88901 -2.29621<br>H 4.51210 -3.06445 1.26843<br>H 6.75394 -2.59456 0.33857<br>H 7.07174 -0.73090 -1.27850<br>H 5.14818 0.65821 -1.97222<br>H 1.68870 2.91470 -0.70644<br>H 0.41706 4.62893 -1.94118<br>H -1.48548 3.96632 -3.39638<br>H -2.08256 1.56717 -3.63478<br>H -0.79601 -0.15768 -2.43221<br>O -0.34226 -1.45522 1.91320<br>O -1.61726 -1.85765 2.38305<br>H -1.56148 -2.82555 2.30436<br>H 2.86931 1.04577 -1.86508<br>H -3.14687 1.11618 0.92544										
**Single-bond dimer, C3-hydroperoxylalkyl radical**										
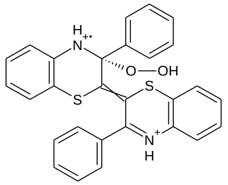<br>S 1.94682 -0.27484 1.34971<br>C 0.68278 0.51164 0.39888<br>C 1.00860 1.89021 0.01729<br>N 2.22591 2.16409 -0.39315<br>C 4.42758 1.63656 -1.23788<br>C 5.54743 0.81912 -1.21689<br>C 5.56287 -0.32767 -0.41817<br>C 4.45711 -0.67206 0.35159<br>C 3.31894 0.13514 0.32704<br>C 3.31755 1.29142 -0.46340<br>C 0.05748 2.99363 0.14931	-2133.71053	-2133.25395	-2133.34462	-2133.75264	-2133.38673	-2134.96576	-2134.59985	-2135.36526	-2134.99935	0.0

C -0.81375 3.01227 1.25238<br>C -1.66909 4.08972 1.43350<br>C -1.68094 5.13784 0.51186<br>C -0.82762 5.11479 -0.59240<br>C 0.04788 4.05244 -0.77401<br>H 4.40348 2.53360 -1.84926<br>H 6.41086 1.07646 -1.82070<br>H 6.44121 -0.96467 -0.40050<br>H 4.46911 -1.56896 0.96259<br>H -0.80249 2.19799 1.97088<br>H -2.32825 4.11236 2.29523<br>H -2.36080 5.97269 0.65134<br>H -0.85110 5.92094 -1.31836<br>H 0.67846 4.02388 -1.65869<br>S -1.62553 0.70390 -0.95091<br>C -0.50901 -0.10187 0.10883<br>C -0.78167 -1.49066 0.68144<br>N -2.14101 -1.96723 0.48373<br>C -4.44725 -2.09616 -0.09800<br>C -5.52457 -1.56391 -0.75084<br>C -5.41089 -0.33953 -1.45857<br>C -4.21090 0.33964 -1.49845<br>C -3.09369 -0.17882 -0.82680<br>C -3.19753 -1.41565 -0.12098<br>C 0.15510 -2.56635 0.13382<br>C 0.53849 -3.64154 0.94164<br>C 1.32134 -4.66019 0.40498<br>C 1.71506 -4.61557 -0.93177<br>C 1.32025 -3.54855 -1.73742<br>C 0.53745 -2.52602 -1.20959<br>H -4.52427 -3.03349 0.44338<br>H -6.47677 -2.08282 -0.72801<br>H -6.27618 0.07003 -1.96822<br>H -4.12644 1.27852 -2.03632<br>H 0.24517 -3.67917 1.98567<br>H 1.62620 -5.48872 1.03689<br>H 2.32628 -5.41195 -1.34550<br>H 1.62004 -3.50961 -2.78008<br>H 0.23579 -1.70139 -1.84909<br>O -0.55708 -1.44233 2.08263<br>O -1.39979 -0.42858 2.62831<br>H -2.02702 -0.95328 3.15253<br>H 2.42578 3.13925 -0.60389<br>H -2.28302 -2.88662 0.89351										
**Single-bond dimer, C2-hydroperoxide**										

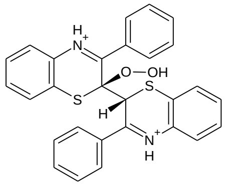<br>S 0.59730 -2.38348 -1.20966<br>C 0.13189 -0.66476 -0.75050<br>C 1.39285 0.16598 -0.96514<br>N 2.49652 -0.24687 -0.37369<br>C 3.83107 -1.62230 1.09795<br>C 4.11817 -2.87003 1.62846<br>C 3.32085 -3.97133 1.30218<br>C 2.22487 -3.83075 0.45844<br>C 1.91917 -2.57797 -0.07707<br>C 2.73624 -1.48287 0.23905<br>C 1.45509 1.38551 -1.75007<br>C 0.37921 2.29233 -1.80145<br>C 0.51636 3.49016 -2.48749<br>C 1.70577 3.78811 -3.15283<br>C 2.76814 2.88266 -3.13362<br>C 2.65008 1.69123 -2.43516<br>H 4.44158 -0.75806 1.34329<br>H 4.96379 -2.98686 2.29742<br>H 3.54738 -4.94694 1.71983<br>H 1.59913 -4.68475 0.22061<br>H -0.55338 2.07424 -1.29804<br>H -0.31020 4.19273 -2.50642<br>H 1.80118 4.72192 -3.69827<br>H 3.68297 3.10003 -3.67487<br>H 3.46500 0.97349 -2.47090<br>C -0.41711 -0.58378 0.72837<br>S -1.56984 -1.90708 1.17257<br>C -2.99436 -1.34276 0.32016<br>C -3.20789 0.03155 0.14363<br>N -2.30341 0.94459 0.69459<br>C -1.08110 0.71095 1.13037<br>C -3.96249 -2.24470 -0.12157<br>C -5.11168 -1.77251 -0.74732<br>C -5.30514 -0.40210 -0.94268<br>C -4.35602 0.50475 -0.49598<br>C -0.44244 1.68352 1.99996<br>C 0.95501 1.69808 2.16644<br>C 1.56055 2.65302 2.96980<br>C 0.78277 3.59762 3.63785<br>C -0.60787 3.57657 3.50975<br>C -1.21830 2.62908 2.70469<br>H -3.80842 -3.31039 0.01426	-2134.34771	-2133.87753	-2133.96824	-2134.39581	-2134.01634	-2135.61306	-2135.23359	-2136.01115	-2135.63168	1.1

H -5.85667 -2.48063 -1.09541<br>H -6.19842 -0.04186 -1.44142<br>H -4.49688 1.57237 -0.63697<br>H 1.58534 0.97611 1.66280<br>H 2.63991 2.65670 3.07742<br>H 1.25689 4.33900 4.27334<br>H -1.21772 4.28897 4.05517<br>H -2.30329 2.60143 2.67691<br>O -0.93100 -0.26081 -1.55154<br>O -0.59405 -0.43003 -2.92973<br>H -1.33402 -0.98619 -3.22697<br>H 0.43019 -0.75502 1.39572<br>H 3.29464 0.38306 -0.42046<br>H -2.68205 1.87059 0.87290										
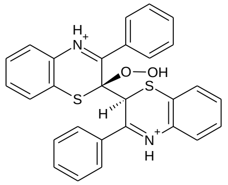<br>S 1.21893 -1.03079 1.58143<br>C -0.16606 -0.41619 0.52683<br>C -0.88599 -1.67355 0.04158<br>N -0.15254 -2.56777 -0.58715<br>C 1.85176 -3.47996 -1.58220<br>C 3.22402 -3.66889 -1.54055<br>C 3.98682 -3.04947 -0.54618<br>C 3.38756 -2.22248 0.39679<br>C 2.00688 -2.01350 0.36090<br>C 1.24531 -2.66129 -0.62460<br>C -2.29129 -1.96531 0.25993<br>C -3.27780 -0.96159 0.24085<br>C -4.61308 -1.30259 0.39666<br>C -4.98257 -2.63334 0.59657<br>C -4.00977 -3.63306 0.64487<br>C -2.67298 -3.30630 0.47632<br>H 1.24621 -3.96153 -2.34405<br>H 3.70113 -4.30176 -2.28091<br>H 5.06088 -3.20079 -0.51344<br>H 3.98588 -1.72847 1.15536<br>H -3.00568 0.07370 0.08471<br>H -5.36990 -0.52563 0.36318<br>H -6.02921 -2.89040 0.72800<br>H -4.29185 -4.66435 0.82950<br>H -1.92143 -4.08479 0.57288<br>C 0.32770 0.45904 -0.67242<br>S -0.92083 0.78264 -1.95127<br>C -1.79814 2.09197 -1.17180	-<br>2134.35192	-<br>2133.88156	-<br>2133.96934	-<br>2134.39741	-<br>2134.01483	-<br>2135.61800	-<br>2135.23542	-<br>2136.01606	-<br>2135.63348	0.0

C -1.14130 2.96298 -0.29276<br>N 0.22917 2.80038 -0.03265<br>C 0.97398 1.74194 -0.21648<br>C -3.13375 2.33502 -1.49303<br>C -3.79692 3.42232 -0.93086<br>C -3.13890 4.27479 -0.04286<br>C -1.80664 4.05063 0.27465<br>C 2.40326 1.82594 0.04674<br>C 3.31587 1.11988 -0.75643<br>C 4.68054 1.25696 -0.53906<br>C 5.14814 2.06905 0.49316<br>C 4.24724 2.75657 1.30904<br>C 2.88342 2.64698 1.08549<br>H -3.65096 1.67301 -2.18069<br>H -4.83676 3.59993 -1.18543<br>H -3.66204 5.11548 0.39963<br>H -1.28133 4.70855 0.96079<br>H 2.97454 0.50789 -1.58471<br>H 5.37957 0.72866 -1.17903<br>H 6.21560 2.16257 0.66704<br>H 4.60882 3.37087 2.12703<br>H 2.19483 3.15166 1.75716<br>O -0.96528 0.43529 1.28184<br>O -1.34991 -0.20796 2.49837<br>H -1.04718 0.44742 3.14920<br>H 1.08597 -0.13207 -1.19203<br>H -0.65592 -3.33214 -1.03204<br>H 0.71069 3.64646 0.26533										
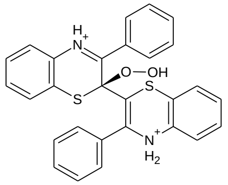	n.d.									
**Single-bond dimer, C3-hydroperoxide**										
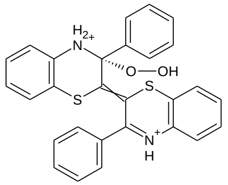<br>S 1.17817 -1.58495 1.42435<br>C 0.96023 -0.06767 0.52885<br>C 2.19962 0.44617 -0.06677<br>N 3.01410 -0.40040 -0.65213<br>C 3.63643 -2.49573 -1.67836<br>C 3.57904 -3.88137 -1.69573	-<br>2134.33888	-<br>2133.86790	-<br>2133.95589	-<br>2134.38555	-<br>2134.00256	-<br>2135.60808	-<br>2135.22509	-<br>2136.00750	-<br>2135.62451	0.0

C 2.77933 -4.55948 -0.77258<br>C 2.02514 -3.85861 0.16285<br>C 2.06763 -2.46407 0.18418<br>C 2.88110 -1.79246 -0.73723<br>C 2.58513 1.84976 0.01374<br>C 2.29073 2.57087 1.18502<br>C 2.71235 3.88845 1.30563<br>C 3.40241 4.50093 0.25969<br>C 3.68381 3.79260 -0.91089<br>C 3.28847 2.46892 -1.03539<br>H 4.25772 -1.95591 -2.38670<br>H 4.15903 -4.43349 -2.42725<br>H 2.73633 -5.64375 -0.78465<br>H 1.39938 -4.38938 0.87333<br>H 1.76343 2.09050 2.00428<br>H 2.50123 4.43817 2.21708<br>H 3.71806 5.53550 0.35290<br>H 4.20451 4.27698 -1.73044<br>H 3.47765 1.93990 -1.96570<br>S -0.41855 2.13292 -0.36926<br>C -0.23507 0.57210 0.44892<br>C -1.44375 -0.03782 1.16809<br>N -2.57899 1.00579 1.20325<br>C -4.37335 1.28695 -0.48649<br>C -4.74043 1.72766 -1.75647<br>C -3.78789 2.27806 -2.61287<br>C -2.45408 2.38666 -2.22330<br>C -2.08203 1.95688 -0.95149<br>C -3.04955 1.42913 -0.10502<br>C -1.97765 -1.31697 0.56127<br>C -2.75258 -2.17658 1.34857<br>C -3.29438 -3.32681 0.78278<br>C -3.07573 -3.61686 -0.56330<br>C -2.31164 -2.75450 -1.34707<br>C -1.76068 -1.60478 -0.78846<br>H -5.10563 0.85299 0.18766<br>H -5.77309 1.63518 -2.07510<br>H -4.08066 2.61506 -3.60201<br>H -1.71010 2.79580 -2.89888<br>H -2.92125 -1.96563 2.40009<br>H -3.88645 -3.99714 1.39783<br>H -3.50088 -4.51502 -1.00092<br>H -2.13979 -2.97474 -2.39590<br>H -1.15999 -0.94467 -1.40689<br>H -3.34312 0.59439 1.74874<br>O -1.15656 -0.29680 2.50929<br>O -0.70547 0.94402 3.08297<br>H -1.07228 0.86383 3.97973<br>H 3.87887 -0.01571 -1.02458<br>H -2.22226 1.80565 1.75092										

Single-bond dimer, dioxolane										
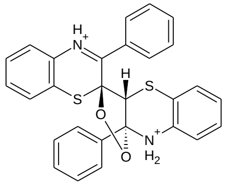<br>S 1.68455 -1.71512 0.69880<br>C 1.05639 -0.19962 -0.05308<br>C 2.07432 0.92433 0.11676<br>N 3.32902 0.65626 -0.14310<br>C 5.21557 -0.60267 -0.95587<br>C 5.83409 -1.82409 -1.16656<br>C 5.17520 -3.01081 -0.83034<br>C 3.89370 -2.98468 -0.29193<br>C 3.25677 -1.76046 -0.08646<br>C 3.92717 -0.57703 -0.41267<br>C 1.76031 2.29347 0.52487<br>C 2.52563 2.88301 1.54592<br>C 2.28313 4.19999 1.91735<br>C 1.29990 4.94048 1.26254<br>C 0.54945 4.36316 0.23713<br>C 0.76499 3.04117 -0.12829<br>H 5.71657 0.32733 -1.20866<br>H 6.83139 -1.85471 -1.59146<br>H 5.66167 -3.96639 -0.99675<br>H 3.38315 -3.90839 -0.03915<br>H 3.27326 2.29721 2.07306<br>H 2.86039 4.64535 2.72098<br>H 1.11678 5.97086 1.55107<br>H -0.20258 4.94575 -0.28488<br>H 0.20459 2.61343 -0.95390<br>S -0.94635 -0.85221 1.90524<br>C -0.38336 0.11413 0.46652<br>C -1.20904 0.00800 -0.82085<br>N -1.58888 -1.46111 -1.06340<br>C -3.61535 -2.74978 -0.54329<br>C -4.49027 -3.29995 0.38552<br>C -4.27060 -3.09503 1.74769<br>C -3.19195 -2.33696 2.18693<br>C -2.29604 -1.78840 1.26272<br>C -2.52592 -2.01757 -0.09078<br>C -2.41460 0.89024 -0.97415<br>C -3.05170 1.45021 0.13582<br>C -4.17568 2.25099 -0.04698<br>C -4.66131 2.49498 -1.32985<br>C -4.02431 1.93654 -2.43744<br>C -2.90335 1.13194 -2.26436	-2134.34571	-2133.87403	-2133.95917	-2134.39217	-2134.00563	-2135.60936	-2135.22282	-2136.00679	-2135.62026	3.4

H -0.42027 1.15727 0.78510<br>H -3.77326 -2.89504 -1.60823<br>H -5.34132 -3.87933 0.04505<br>H -4.95372 -3.51640 2.47804<br>H -3.04386 -2.16134 3.24796<br>H -2.68975 1.26626 1.14321<br>H -4.66989 2.68336 0.81704<br>H -5.53714 3.12165 -1.46752<br>H -4.39645 2.12882 -3.43857<br>H -2.40591 0.71223 -3.13431<br>O 0.87502 -0.44906 -1.45929<br>O -0.28396 0.32988 -1.82235<br>H -1.99538 -1.51857 -2.00356<br>H -0.71862 -2.01328 -1.10319<br>H 3.96087 1.45520 -0.13861										
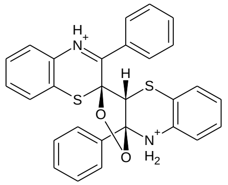<br>S -1.42897 -1.23672 -0.86505<br>C -0.89541 0.08728 0.25135<br>C -1.79181 1.30834 0.14569<br>N -3.07493 1.15677 -0.05048<br>C -5.20922 0.06166 -0.21363<br>C -5.95314 -1.07268 -0.48984<br>C -5.30967 -2.26141 -0.85059<br>C -3.92407 -2.32478 -0.92884<br>C -3.16315 -1.18698 -0.65158<br>C -3.81417 -0.00023 -0.30124<br>C -1.29824 2.67708 0.31685<br>C -0.55662 3.03831 1.45463<br>C -0.14522 4.35531 1.61334<br>C -0.45113 5.30963 0.64174<br>C -1.18665 4.95259 -0.48697<br>C -1.62090 3.64173 -0.65078<br>H -5.69464 0.98952 0.07450<br>H -7.03484 -1.03506 -0.42263<br>H -5.89444 -3.14990 -1.06531<br>H -3.42939 -3.25153 -1.20143<br>H -0.35150 2.31093 2.23451<br>H 0.40834 4.63873 2.50265<br>H -0.11727 6.33485 0.76794<br>H -1.42038 5.69267 -1.24534<br>H -2.17249 3.35840 -1.54279<br>S 1.27475 0.29984 -1.62210<br>C 0.59030 0.35442 0.03016	-2134.32683	-2133.85473	-2133.94015	-2134.37537	-2133.98869	-2135.59397	-2135.20729	-2135.99130	-2135.60461	13.2

C 1.21868 -0.62566 1.03363<br>N 2.56732 -0.04712 1.36879<br>C 4.77960 0.49016 0.52488<br>C 5.67649 0.86089 -0.46682<br>C 5.21616 1.06895 -1.76616<br>C 3.87037 0.91401 -2.06638<br>C 2.94600 0.54172 -1.08063<br>C 3.43305 0.32758 0.21504<br>C 1.31882 -2.11086 0.75202<br>C 0.61306 -2.98782 1.58487<br>C 0.68740 -4.36151 1.37948<br>C 1.46860 -4.87564 0.34676<br>C 2.18457 -4.00830 -0.47447<br>C 2.11512 -2.63168 -0.27677<br>H 0.78894 1.35657 0.42342<br>H 5.12420 0.33008 1.54277<br>H 6.72548 0.98599 -0.22244<br>H 5.90637 1.35881 -2.55150<br>H 3.51673 1.08416 -3.07888<br>H 0.00331 -2.59879 2.39166<br>H 0.13203 -5.02890 2.03103<br>H 1.52464 -5.94812 0.18660<br>H 2.80442 -4.39821 -1.27565<br>H 2.69233 -1.98814 -0.92844<br>O -0.93500 -0.29714 1.64808<br>O 0.41592 -0.34539 2.16589<br>H 2.41675 0.78013 1.96271<br>H 3.07587 -0.72546 1.94853<br>H -3.62398 2.01357 0.00825										
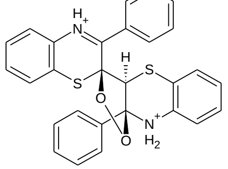<br>S -1.29834 -1.35137 -1.69444<br>C -0.00902 -0.50928 -0.68195<br>C 1.05210 -1.53904 -0.35156<br>N 0.61617 -2.64130 0.20492<br>C -1.00785 -4.04535 1.31599<br>C -2.30132 -4.53883 1.39993<br>C -3.29335 -4.05385 0.54416<br>C -3.00447 -3.06369 -0.38896<br>C -1.70938 -2.55133 -0.48021<br>C -0.71565 -3.05709 0.37320<br>C 2.48245 -1.36806 -0.56477<br>C 3.37866 -1.90565 0.38127<br>C 4.74620 -1.80497 0.17791	-<br>2134.34680	-<br>2133.87518	-<br>2133.95956	-<br>2134.39334	-<br>2134.00610	-<br>2135.61557	-<br>2135.22833	-<br>2136.01288	-<br>2135.62564	0.0

C 5.23781 -1.18590 -0.97273<br>C 4.35664 -0.65667 -1.91473<br>C 2.98454 -0.73211 -1.71428<br>H -0.22740 -4.41523 1.97407<br>H -2.53698 -5.30220 2.13341<br>H -4.30487 -4.44081 0.61203<br>H -3.78095 -2.67816 -1.04156<br>H 3.01276 -2.34517 1.30534<br>H 5.42927 -2.19939 0.92297<br>H 6.30922 -1.11214 -1.13140<br>H 4.73782 -0.18361 -2.81376<br>H 2.31295 -0.33822 -2.46706<br>S 0.52823 0.39055 1.97758<br>C -0.60565 0.21426 0.55516<br>C -1.04809 1.54483 -0.10928<br>N -0.40157 2.74360 0.62401<br>C 1.81763 3.73926 0.13894<br>C 3.20211 3.66964 0.28570<br>C 3.78531 2.59841 0.96056<br>C 3.00285 1.56653 1.47681<br>C 1.61893 1.62676 1.33550<br>C 1.05469 2.72403 0.68969<br>C -2.53568 1.80268 -0.16250<br>C -3.13461 2.23628 -1.34814<br>C -4.49897 2.51425 -1.36668<br>C -5.26250 2.36834 -0.21025<br>C -4.66185 1.94002 0.97260<br>C -3.29988 1.65625 1.00018<br>H -1.46662 -0.32386 0.95441<br>H 1.35144 4.57228 -0.37893<br>H 3.82244 4.45743 -0.12788<br>H 4.86372 2.55445 1.07397<br>H 3.46160 0.72145 1.97894<br>H -2.54754 2.34463 -2.25382<br>H -4.96381 2.84355 -2.29062<br>H -6.32615 2.58502 -0.23054<br>H -5.25207 1.81935 1.87531<br>H -2.85228 1.31335 1.93031<br>O 0.53618 0.54639 -1.42231<br>O -0.51270 1.52977 -1.40310<br>H -0.79177 2.76795 1.57716<br>H -0.73258 3.59618 0.15976<br>H 1.32353 -3.32754 0.45913										

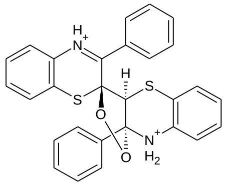<br>S 0.82942 2.38937 -1.98269<br>C -0.08827 1.05839 -1.12227<br>C -0.86482 1.69034 0.02132<br>N -0.18256 2.44851 0.84128<br>C 1.80322 3.41737 1.81753<br>C 3.06120 3.97890 1.65888<br>C 3.64218 4.04577 0.39014<br>C 2.97897 3.54117 -0.72381<br>C 1.71784 2.96287 -0.57776<br>C 1.13648 2.91450 0.69826<br>C -2.28305 1.47353 0.28604<br>C -2.69408 1.23926 1.61134<br>C -4.04471 1.11161 1.90447<br>C -4.99416 1.24102 0.89090<br>C -4.59044 1.48260 -0.42227<br>C -3.24187 1.58414 -0.73610<br>H 1.33765 3.35923 2.79707<br>H 3.58921 4.36585 2.52354<br>H 4.62675 4.48516 0.26724<br>H 3.43982 3.58515 -1.70516<br>H -1.95861 1.10620 2.40010<br>H -4.35461 0.90553 2.92378<br>H -6.05054 1.14948 1.12414<br>H -5.32969 1.59355 -1.20897<br>H -2.94301 1.78881 -1.75696<br>S 1.54625 -0.34118 0.88135<br>C 0.80023 -0.14831 -0.73901<br>C -0.08106 -1.32729 -1.18252<br>N 0.88085 -2.42000 -1.55868<br>C 2.46196 -4.04070 -0.66854<br>C 3.42927 -4.46760 0.23018<br>C 3.81453 -3.62907 1.27506<br>C 3.23893 -2.37456 1.41550<br>C 2.25930 -1.92291 0.52067<br>C 1.88506 -2.78350 -0.51983<br>C -1.21540 -1.87452 -0.33890<br>C -2.50237 -1.87335 -0.89165<br>C -3.57006 -2.42117 -0.18807<br>C -3.36767 -2.98106 1.07113<br>C -2.08907 -2.99316 1.62196<br>C -1.01475 -2.44582 0.92544<br>H 1.62646 -0.10373 -1.45754	-2134.31965	-2133.84791	-2133.93232	-2134.36763	-2133.98030	-2135.59218	-2135.20485	-2135.98953	-2135.60220	14.7

H 2.15918 -4.68456 -1.48945<br>H 3.87698 -5.44796 0.11114<br>H 4.57034 -3.94997 1.98430<br>H 3.54595 -1.72410 2.22897<br>H -2.67290 -1.44655 -1.87282<br>H -4.56146 -2.40817 -0.62996<br>H -4.20157 -3.40940 1.61905<br>H -1.91729 -3.43297 2.59940<br>H -0.03499 -2.48501 1.38223<br>O -0.99114 0.50686 -2.07259<br>O -0.53042 -0.80901 -2.42345<br>H 1.36416 -2.13615 -2.42333<br>H 0.33870 -3.25903 -1.79731<br>H -0.70146 2.84475 1.62347										
**Double-bond dimers**										
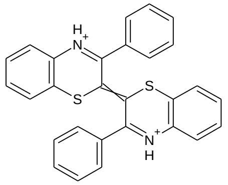<br>S 1.46649 -0.10793 -2.40504<br>C 0.69413 0.00107 -0.82824<br>C 1.52053 0.29110 0.32227<br>N 2.80079 -0.03008 0.32226<br>C 4.81332 -1.16368 -0.36316<br>C 5.58899 -1.71872 -1.36915<br>C 5.10409 -1.77253 -2.67877<br>C 3.84015 -1.28282 -2.98727<br>C 3.04843 -0.73573 -1.97716<br>C 3.54262 -0.67146 -0.67104<br>C 1.04838 1.07519 1.46985<br>C 0.20282 2.17794 1.26261<br>C -0.16382 2.97811 2.33800<br>C 0.29766 2.68434 3.62060<br>C 1.13805 1.58972 3.82998<br>C 1.52053 0.78970 2.76193<br>H 5.17941 -1.10935 0.65787<br>H 6.57424 -2.10672 -1.13481<br>H 5.71204 -2.20501 -3.46650<br>H 3.46406 -1.33362 -4.00403<br>H -0.12809 2.43605 0.26179<br>H -0.80237 3.83942 2.17042<br>H 0.00308 3.30850 4.45857<br>H 1.48459 1.35013 4.83005<br>H 2.13476 -0.08789 2.94692<br>S -1.46649 0.10793 -2.40504<br>C -0.69413 -0.00107 -0.82824	-1982.93615	-1982.49979	-1982.58217	-1982.97583	-1982.62186	-1984.09187	-1983.73789	-1984.44227	-1984.08829	0.0

C -1.52053 -0.29110 0.32227<br>N -2.80079 0.03008 0.32226<br>C -4.81332 1.16368 -0.36316<br>C -5.58899 1.71872 -1.36915<br>C -5.10409 1.77253 -2.67877<br>C -3.84015 1.28282 -2.98727<br>C -3.04843 0.73573 -1.97716<br>C -3.54262 0.67146 -0.67104<br>C -1.04838 -1.07519 1.46985<br>C -0.20282 -2.17794 1.26261<br>C 0.16382 -2.97811 2.33800<br>C -0.29766 -2.68434 3.62060<br>C -1.13805 -1.58972 3.82998<br>C -1.52053 -0.78970 2.76193<br>H -5.17941 1.10935 0.65787<br>H -6.57424 2.10672 -1.13481<br>H -5.71204 2.20501 -3.46650<br>H -3.46406 1.33362 -4.00403<br>H 0.12809 -2.43605 0.26179<br>H 0.80237 -3.83942 2.17042<br>H -0.00308 -3.30850 4.45857<br>H -1.48459 -1.35013 4.83005<br>H -2.13476 0.08789 2.94692<br>H -3.33823 -0.29419 1.12187<br>H 3.33823 0.29419 1.12187										

Table S7. pK_a values computed in methanol at different theory levels for all bibenzothiazine derivatives examined. For each chemical species and for each protonation state, the $G_{RRHO,calc}$ value (see Tables S4-S6) of the most stable stereoisomer identified was used in the calculation.

	**PBE0 / 6-31+G(d,p) / SMD**		**M062X / 6-31+G(d,p) / SMD**		**M062X / 6-311++G(2d,2p) / SMD**	
	**pK_{a1}**	**pK_{a2}**	**pK_{a1}**	**pK_{a2}**	**pK_{a1}**	**pK_{a2}**
**Single-bond dimer**	4.3	3.2	3.6	2.3	3.6	2.4
**Single-bond dimer, radical**	6.6	1.6	6.6	0.3	6.6	0.4
**Single-bond dimer, C2-peroxyl radical**	3.7	1.9	3.0	1.2	3.0	1.3
**Single-bond dimer, C3-peroxyl radical**	4.2	-2.0	3.4	-2.4	3.5	-2.3
**Single-bond dimer, dioxolane radical**	4.9	2.0	4.8	0.7	4.9	0.8
**Single-bond dimer, dioxane radical**	4.7	-1.0	4.2	-1.1	4.4	-1.2
**Single-bond dimer, C2-hydroperoxylalkyl radical**	5.4	2.4	5.4	1.5	5.6	1.6
**Single-bond dimer, C3-hydroperoxylalkyl radical**	5.0	2.7	4.8	2.1	5.2	1.9
**Single-bond dimer, C2-hydroperoxide**	3.8	3.1	3.5	2.2	3.5	2.2
**Single-bond dimer, C3-hydroperoxide**	4.3	0.6	3.7	0.3	3.8	0.3
**Single-bond dimer, dioxolane**	3.1	-0.4	2.7	-0.9	2.8	-0.7
**Double-bond dimers**	4.6	2.4	4.2	1.3	4.3	1.4

Table S8. pK_a values computed in methanol at different theory levels for some reference nitrogen bases and corresponding experimental values. For each chemical species and for each protonation state, the $G_{RRHO,calc}$ value of the most stable stereoisomer identified was used in the calculation. At each theory level, a linear fitting with respect to the experimental values was used to correct the raw pK_a values.

	PBE0 / 6-31+G(d,p) / SMD	M062X / 6-31+G(d,p) / SMD	M062X / 6-311++G(2d,2p) / SMD	Experimental[a]
**pyridine**	5.87	5.90	5.85	5.44
**aniline**	4.23	4.42	4.30	6.05
**2-chloroaniline**	2.95	2.88	2.81	3.71
**4-chloroaniline**	3.77	3.87	3.76	4.95
**2-nitroaniline**	0.73	0.39	0.51	0.20
**4-nitroaniline**	1.30	1.46	1.46	1.55
**4-chloro-2-nitroaniline**	0.69	0.73	0.86	-0.67
**2-methylpyridine**	6.39	6.42	6.43	6.18
**quinoline**	5.84	5.73	5.74	5.16
**2-methylquinoline**	6.86	6.83	6.90	6.06

[a] Rived, F.; Rosés, M.; Bosch, E. *Anal. Chim. Acta* **1998**, *374*, 309-324.
